# Additive and Emergent Catalytic Properties of Dimeric Unnatural Amino Acid Derivatives: Aldol and Conjugate Additions

**DOI:** 10.1002/chem.202102394

**Published:** 2021-10-13

**Authors:** María de Gracia Retamosa, Andrea Ruiz‐Olalla, Maddalen Agirre, Abel de Cózar, Tamara Bello, Fernando P. Cossío

**Affiliations:** ^1^ Donostia International Physics Center (DIPC) P° Manuel Lardizabal 4 20018 Donostia/San Sebastián Spain; ^2^ Departamento de Química Orgánica I and Instituto de Innovación en Química Avanzada (ORFEO-CINQA) University of the Basque Country (UPV/EHU) P° Manuel Lardizabal 3 20018 Donostia/San Sebastián Spain; ^3^ Ikerbasque, Basque Foundation for Science Plaza Euskadi 5 48009 Bilbao Spain; ^4^ Present address: Departamento de Química Orgánica and Centro de Innovación en Químca Avanzada (ORFEO-CINQA) Instituto de Síntesis Orgánica Universidad de Alicante 03080 Alicante Spain; ^5^ Present address: CIC Energigune, Parque Tecnológico de Álava 01510 Vitoria/Gasteiz Spain.

**Keywords:** aldol reaction, asymmetric catalysis, conjugate additions, DFT calculations, 1,3-dipolar reactions

## Abstract

Different densely substituted L‐ and D‐proline esters were prepared by asymmetric (3+2) cycloaddition reactions catalyzed by conveniently selected EhuPhos chiral ligands. The γ‐nitro‐2‐alkoxycarbonyl pyrrolidines thus obtained in either their *endo* or *exo* forms were functionalized and coupled to yield the corresponding γ‐dipeptides. The catalytic properties of these latter dimers were examined using aldol and conjugate additions as case studies. When aldol reactions were analyzed, an additive behavior in terms of stereocontrol was observed on going from the monomers to the dimers. In contrast, in the case of the conjugate additions between ketones and nitroalkenes, the monomers did not catalyze this reaction, whereas the different γ‐dipeptides promoted the formation of the corresponding Michael adducts. Therefore, in this latter case emergent catalytic properties were observed for these novel γ‐dipeptides based on unnatural proline derivatives. Under certain conditions stoichiometric amounts of ketone, acid and nitroalkene), formation of *N‐*acyloxy‐2‐oxooctahydro‐1*H*‐indoles was observed.

## Graphical abstract

**Monomeric and dimeric organocatalysts behave differently**: Aldol and Michael reactions catalysed by γ‐dipeptides derived from densely substituted unnatural proline derivatives show different behaviours. In aldol reactions an additive scheme is observed, whereas in conjugate reactions the catalytic activity of these dipeptides constitutes an emergent property. Experimental, kinetic and computational studies are reported to understand these phenomena.

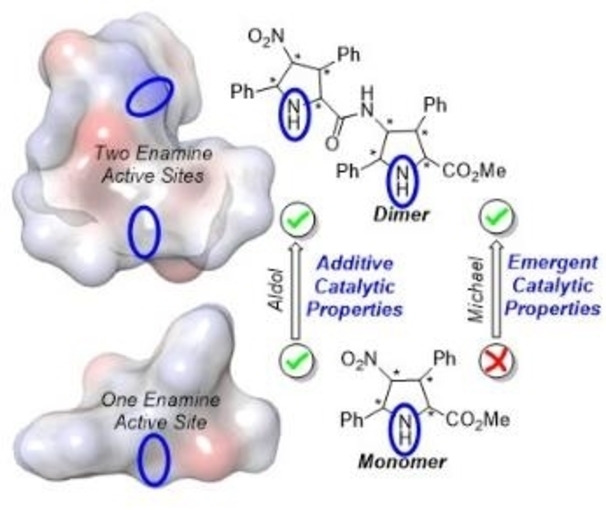

## Introduction

**Scheme 1:**
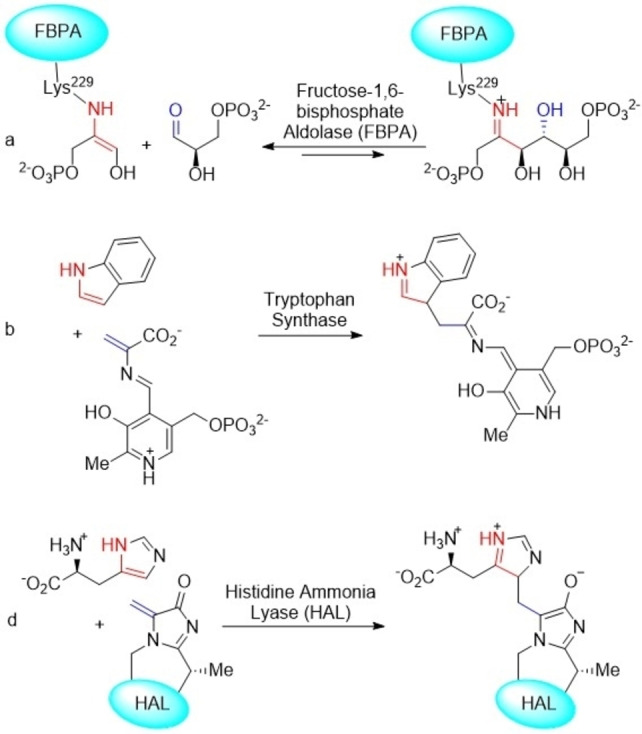
Examples of aldol and conjugate additions catalyzed by enamine‐based enzymes.

Organocatalyzed aldol^[1]^ and conjugate^[2]^ additions in which enamine reactants or intermediates act as HOMO‐activated nucleophilic species^[3]^ can be considered as analogs of naturally occurring enzymatic^[4]^ processes catalyzed by type I aldolases such as fructose‐1,6‐bisphosphate aldolase^[5]^ (FBPA, Scheme 1a) or synthases or lyases like, for example, tryptophan synthase^[6]^ or histidine ammonia lyase^[7]^ (Scheme 1b,c). These additions of enamines to electrophilic C=O and C=C bonds[^8]^ can be catalyzed by simple natural amino acids. In particular, L‐proline and its derivatives^[9]^ constitute one of the most widely used families of organocatalysts.^[10]^

**Figure 1:**
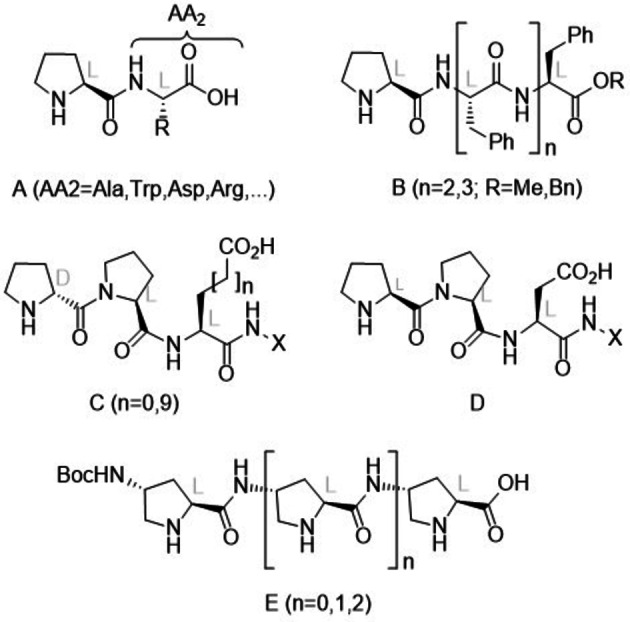
Previously described proline containing α‐ and γ‐oligopeptides that catalyze aldol and conjugate additions.

From these monomeric species, the next step in modular complexity consists of using di‐, tri‐ or oligopeptides^[11]^ to promote these addition reactions. This transition from monomeric amino acids to oligopeptides is considered as a fundamental step in prebiotic chemistry.^[12]^ Although dendritic oligopeptides have been tested as organocatalysts,^[13]^ most peptidic organocatalysts consist of a linear sequence of natural amino acids. Thus, as far as aldol reactions are concerned, different oligopeptides have been reported to be able to catalyze this reaction.[^11a^, ^14^] It is interesting to note that most of these di‐ and tripeptides start with a L‐proline unit^[15]^ (Figure 1, dipeptides **A** and oligopeptides **B**). In the case of conjugate reactions between nitroalkenes and ketones^[16]^ or aldehydes,^[17]^ H−L(D)‐Pro‐L‐Pro‐X tri‐ and oligopeptides^[18]^ (Figure 1, Compounds **C** and **D**) have been described as suitable organocatalysts involving aldehydes as nucleophiles. It is interesting to note that in catalytic oligopeptides **C**, two consecutive D‐ and L‐Pro units are more efficient in terms of stereocontrol.

In all the preceding cases, α‐peptides based on natural or modified α‐amino acids were used to catalyze aldol and conjugate additions. In addition to these results, γ‐peptides formed from *trans*‐4‐amino‐L‐proline^[19]^ (Figure 1, compounds **E**) were synthetized and used in conjugate additions of in situ formed nitronates on α,β‐unsaturated ketones.^[20]^ In these conjugated Henry reactions, the observed chemical yields were from moderate to excellent and the stereocontrol was found to depend on the nature of the nitroalkane, but not on the length of the γ‐oligopeptide **E**.

**Figure 2:**
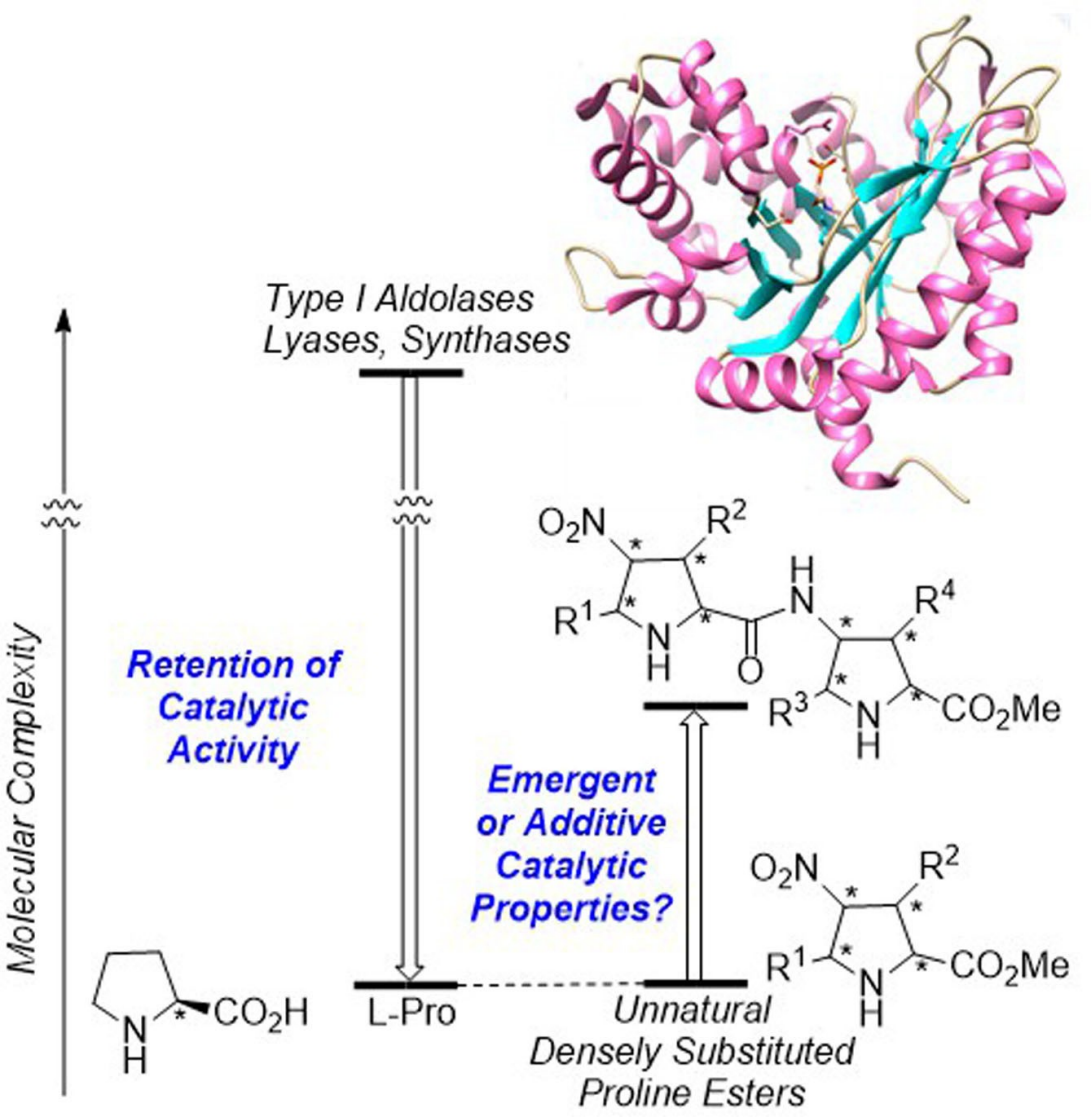
Top‐down and bottom‐up approaches to catalysis from natural and unnatural amino acids. The possibilities of preserving original catalytic properties in the first approach and of observing emergent catalytic properties in the latter are highlighted.

Recently, some of us reported on the chemical synthesis of densely substituted unnatural proline derivatives and its use as organocatalysts.^[21]^ Thus, using EhuPhos catalytic ligands, we synthesized with complete regio‐, distereo‐ and enantiocontrol *exo* (X) or *endo* (N) γ‐nitroproline esters (Figure 2) via formal (3+2) cycloaddition reactions between nitroalkenes and in situ formed *N‐*metallated azomethine ylides.[^22^, ^23^] We observed that, in turn, these latter unnatural proline derivatives catalyze aldol reactions,[^22^, ^24^] ring‐opening polymerizations^[25]^ and Michael additions.^[26]^ Moreover,in one case we observed an unprecedented three‐component cyclization reaction that permitted a concise synthesis of the unnatural enantiomer of naturally occurring alkaloid pancracine.^[27]^ It is interesting to note that, whereas γ‐nitroproline esters catalyzed efficiently aldol reactions,[^22^, ^24^] conversion to their corresponding γ‐aminoproline ester derivatives was required to permit the Michael addition between ketones and nitroalkenes.^[26]^ This suggests a relevant role of the substitution pattern of these organocatalysts. Indeed, the configuration of their distal substituents determines the stereochemical outcome of the organocatalyzed reactions.[^24^, ^25^, ^26^]

Based on these precedents, we wondered whether an increase in complexity on going from the monomeric organocatalysts to the dimeric γ‐dipeptides (Figure 2) would lead to the emergence of novel catalytic properties not present in the lower level of organization. This would suppose a small evolution step towards the complexity reached by proteins along very large time spans.^[28]^ With this idea in mind, we synthesized unnatural γ‐peptides based on our previously developed densely substituted proline analogues in order to assess the aldolase‐like and the synthase or lyase‐like properties of these novel dimeric organocatalysts. These results are presented and discussed in the following sections of this article.

## Results and Discussion

### Synthesis of unnatural γ‐dipeptides

**Scheme 2:**
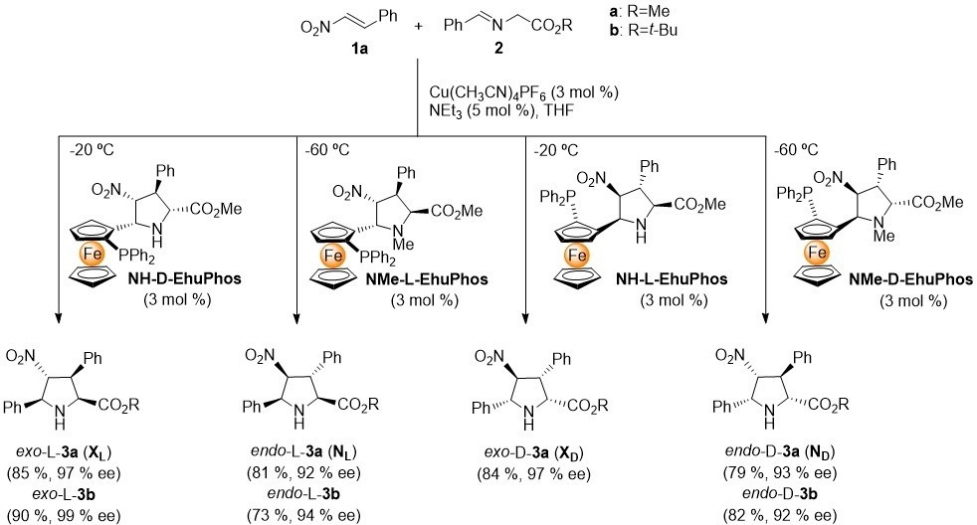
Synthesis of monomeric densely substituted proline esters **3** by (3+2) cycloaddition catalyzed by **EhuPhos−Cu(I)** complexes.

The monomeric building blocks required to build the γ‐dipeptides were synthesized first. Thus, ligands **NH−D−EhuPhos** and **NMe−L−EhuPhos**, already reported by our group, were employed in the (3+2) cycloaddition between *trans*‐β‐nitrostyrene **1** and imines **2 a**,**b** to produce the densely substituted pyrrolidines *exo‐*L‐**3 a**,**b** and *endo*‐L‐**3 a**,**b** (where R=Me or ^t^Bu for the a and b series, respectively, Scheme 2). Both the chemical yields and enantiomeric excesses were excellent (Scheme 2). In order to obtain the D‐series of these cycloadducts, the necessary enantiomeric catalytic ligands **NH−L−EhuPhos** and **NMe−D−EhuPhos** were synthesized through a procedure similar to that described for **NH−D−EhuPhos** and **NMe−L−EhuPhos** (see Supporting Information). In all cases reported in Scheme 2, the required four basic building blocks **3 a**,**b** were purified to >99 % and <−99 % ee by recrystallization from ethyl acetate/hexane mixtures.

**Scheme 3:**
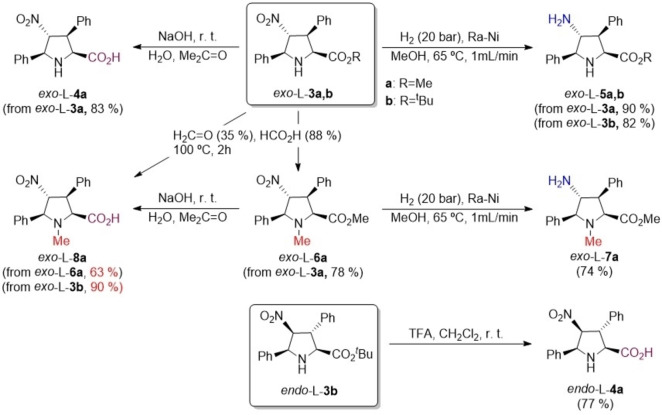
Synthesis of γ‐nitro acids **4 a** and **8 a**, and γ‐amino esters **5 a** and **7 a**.

The nitro and ester group installed in cycloadducts **3 a**,**b** were transformed into the amino and acid groups, respectively, necessary to proceed with the γ‐coupling reactions. The main transformations for the L‐series are gathered in Scheme 3 (see the Supporting Information for similar reactions for the D‐series). Thus, basic hydrolysis of ester *exo‐*L‐**3 a** yielded carboxylic acid *exo‐*L‐**4 a** in good yield. In parallel, hydrogenolysis of *exo‐*L‐**3 a** resulted in the formation of γ‐amino ester *exo‐*L‐**5 a**. A third transformation of *exo‐*L‐**3 a** consisted of its *N‐*methylation to give rise to compound *exo‐*L‐**6 a** (Scheme 3). Similar reactions on this latter compound permitted to prepare carboxylic acid *exo‐*L‐**8 a** and amine *exo‐*L‐**7 a**, respectively. Alternatively, *N‐*methylated amino acid *exo‐*L‐**8 a** was obtained by *N‐*formylation and acid hydrolysis of *tert‐*butyl ester *exo‐*L‐**3 b** in formic acid as solvent. Synthesis of γ‐nitro amino acid *endo‐*L‐**4 a** required deprotection of *tert‐*butyl ester with trifluoroacetic acid (TFA). In this latter case, alternative hydrolysis under basic conditions, similar to those used for the *exo*‐series, resulted in partial isomerization of the 4‐nitro position. Similar transformations were carried out for the D‐series of cycloadducts **3 a**,**b** (see Supporting Information). In summary, (3+2) cycloadditions gathered in Scheme 2 and convenient functionalization reactions shown in Scheme 3 permitted to obtain at will the L‐ and D‐ series of *exo* (X) and *endo* (N) building blocks, which could be condensed to obtain the corresponding γ‐dipeptides.

In order to determine the best reaction conditions for the synthesis of the γ‐dipeptides **9**, different coupling methods were screened in the reaction between *exo‐*L‐**4 a** and *exo*‐L‐**5 b** to yield γ‐dipeptide X_L_X_L_‐**9 a** at room temperature (Table 1).^[29]^ Coupling agents such as diethyl cyanophosphonate (DEPC), propanephosphonic acid anhydride (T3P)^[30]^ or ZrCl_4_ ^[31]^ did not give any trace of product (entries 1, 3 and 4). 1‐Ethyl‐3‐(3‐dimethylamino‐propyl)carbodiimide (EDC)^[32]^ combined with 1‐hydroxybenzotriazole (HOBt) provided low yield due to uncomplete conversion (entry 2). Yields of the γ‐peptidic coupling were improved by employing 2‐(1*H*‐Benzotriazole‐1‐yl)‐1,1,3,3‐tetramethylaminium tetrafluoro‐borate (TBTU)^[33]^ and (benzotriazole‐1‐yl‐oxy)‐tris‐(pyrrolidinophosphonium) hexafluoro‐phosphate (PyBOP)^[34]^ coupling agents (entries 5 and 6). When TBTU was used, full conversion was achieved in a moderate yield of 60 % (entry 5). This result was improved employing PyBOP instead, for which the yield was raised in a shorter reaction time proving to be the most suitable method for the chemical synthesis of these γ‐dipeptides (entry 6). The structure and stereochemical integrity of X_L_X_L_‐**9 a** dimer were also secured by X‐ray diffraction analysis (see Supporting Information). In this crystal structure the pyrrolidinic units are pointing to opposite directions, which suggests that both possible active sites of the pyrrolidine rings are not equivalent.

**Table 1:** Screening of coupling reaction conditions between *exo*‐L‐**4 a** and *exo‐*L‐**5 b** to yield γ‐dipeptide X_L_X_L_‐**9 a**.

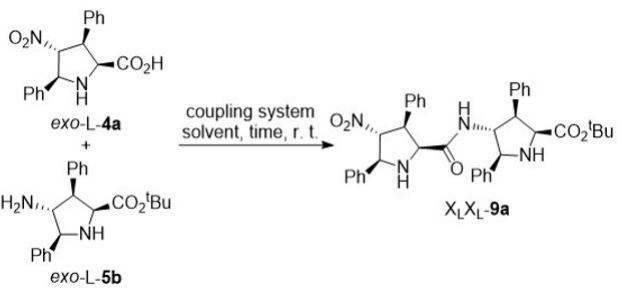					
Entry	Coupling System	Solvent	Time [h]	Conv.^[a]^ [%]	Yield^[b]^ [%]
1	DEPC, NEt_3_	DMF	16	0	–
2	EDC, HOBt ⋅ 2H_2_O, NMM, NEt_3_	DMF	48	80	31
3	T3P (50 % H_2_O)	THF	48	0	–
4	ZrCl_4_ (10 %), 4 Å MS	THF	24	0	–
5	TBTU, DIPEA	DCM	16	>99	60
6	PyBOP, DIPEA	DCM	1	>99	74

[a] Conversions were monitored by ^1^H NMR. [b] Yields of pure X_L_X_L_‐**9 a**.

**Scheme 4:**
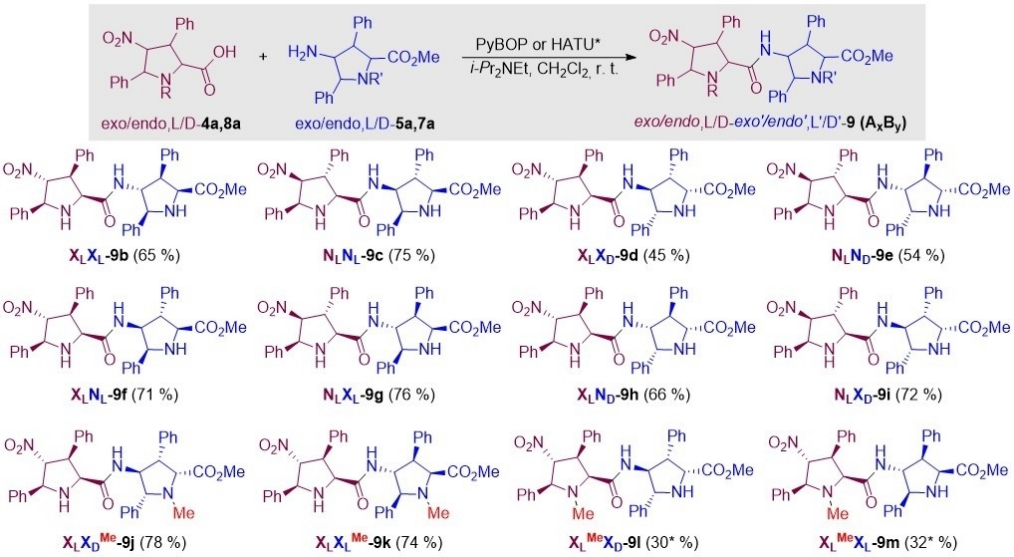
Synthesis of γ‐peptides **9 b**–**m** by coupling between unnatural proline derivatives **4 a**,**8 a** and **5 a**,**7 a** with γ‐dipeptides **9 b**–**m** in hand, we explored their behaviour as organocatalysts in two model addition reactions. The aim was to assess the effect of the combination of *endo/exo* diastereomers, L/D enantiomers and *NH*/*NMe* substitution patterns and, eventually, to search for additive or emergent catalytic properties with respect to the monomeric series. The structural and stereochemical features of the unnatural proline building blocks permitted a flexible enough exploration of the chemical space in order to understand the structure‐activity relationship of dimers **9**.

Under these optimized conditions, γ‐dipeptides **9 b**–**i** were synthesized by combination of diverse X/L and L/D components (Scheme 4). These dipeptides incorporate two *NH*‐pyrrolidine rings and therefore possess two potential active sites for enamine catalysis. Coupling between *NH*‐ and *NMe*‐pyrrolidines should yield γ‐dipeptides with only one active site available for the formation of the intermediate enamine (HOMO activation), the remaining *N*‐Me moiety being available for interaction with the electrophile under acidic conditions (LUMO activation, see below). Reaction between carboxylic acid *exo‐*L‐**4 a** and *N‐*methyl 4‐amino esters *exo‐*D‐**7 a** and *exo*‐L‐**7 a** under the PyBOP/DIPEA system led to the formation of γ‐dipeptides X_L_X_D_^Me^‐**9 j** and X_L_X_L_^Me^‐**9 k** in good yields (Scheme 4). However, when *N‐*methyl carboxylic acid *exo‐*L‐**8 a** was coupled with amines *exo* **‐**D‐**5 a** or *exo*‐L‐**5 a** under the previously optimized conditions, formation of the corresponding condensation products was found to be very low. The best alternative coupling system consisted of using HATU in the presence of DIPEA. However, even under these conditions the desired dipeptides X_L_^Me^X_D_‐**9 l** and X_L_^Me^X_L_‐**9 m** (Scheme 4 and Supporting Information) were obtained in modest yields because of a transesterification reaction leading to the formation of methyl ester derivative *exo‐*L‐**3 a** in 1 : 1 ratio with respect to the respective dipeptide. Compound X_L_^Me^X_L_‐**9 m** was crystalized and its structure was confirmed by X‐ray diffraction analysis (see Supporting Information).

### Aldol reactions

**Scheme 5:**
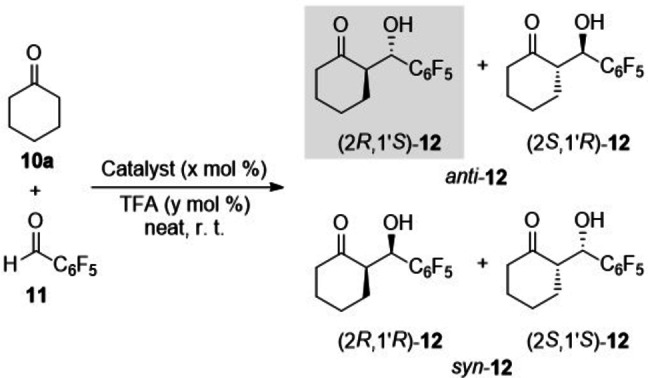
Catalysed aldol reaction between cyclohexanone **10 a** and penta‐fluorobenzaldehyde **11** to yield adducts **12**. *Syn* aldol (2*R*,1’*S*)‐**12** (highlighted in grey) is the major adduct under catalysis of *exo‐*L‐**3 a** (X_L_) and X_L_X_L_ **‐9 b** (see Table 2).

Once synthesized this new generation of organocatalysts and knowing the efficiency of our organocatalysts *exo‐*L‐**3 a** (X_L_) and *endo‐*L‐**3 a** (N_L_) in the aldol reaction,[^22^, ^24^] we proceed to evaluate the outcome of these γ‐dipeptides in this process. Firstly, the reaction conditions between cyclohexanone **10 a** and pentafluoro‐benzaldehyde **11** were optimized employing X_L_X_L_‐**9 b** as catalyst and TFA as acidic additive under the same conditions studied in previous experiments[^22^, ^24^] (Scheme 5, Table 2). The catalytic load could be lowered to 3 mol%, in the presence of 15 mol% of TFA, getting the aldol (2*R*,1’*S*)‐**12** as major product in good yield with no significant loss in the diastereo‐ and enantioselectivity when compared to the monomer *exo‐*L‐**3 a** (entries 1, 2 and 5).

**Table 2:** Catalytic aldol reaction between cyclohexanone **10 a** and pentafluorobenzaldehyde **11** catalysed by *exo‐*L‐**3 a** and X_L_X_L_ **‐9 b**.^[a]^

Entry	Catalyst	Catalytic load [mol %]	TFA [mol %]	*anti:syn* ^*[*b]^	Yield^[c]^ [%]	ee^[d]^ [%]
1	*exo‐*L‐**3 a** (X_L_)	30	30	95 : 5	75	89
2	*exo‐*L‐**3 a** (X_L_)	5	30	95 : 5	81	89
3	X_L_X_L_ **‐9 b**	30	30	89 : 11	66	85
4	X_L_X_L_ **‐9 b**	15	15	94 : 6	66	83
5	X_L_X_L_ **‐9 b**	3	15	93 : 7	86	85

[a] Reactions were monitored by ^19^F NMR and were stirred at room temperature until total consumption of aldehyde **11**. [b] *Anti*:*syn* ratios were measured by ^19^F NMR of crude reaction mixtures. [c] Yields refer to pure aldol adducts. [d] Enantiomeric excesses measured by HPLC correspond to the major *anti*‐diastereomer (2*R*,1’*S*)‐**12**.

Once found the proper reaction conditions, we evaluated the catalytic performance of selected dimers **9**. The obtained results are gathered in Table 3. When the catalytic aldol reaction between cyclohexanone **10 a** and pentafluorobenzaldehyde **11** was carried out in the presence of the different dimers, all obtained *anti*:*syn* relationship were excellent providing good to high yields. On the other hand, the enantioselectivity of the process was affected by the configuration of the monomeric units that form the corresponding dipeptides. For each dimer, we compared the observed enantiomeric excess (ee) with the would be expected by averaging the contributions of both units, denoted in Table 3 as ee_av_. In the case of X_L_X_L_‐**9 b**, both values were almost coincident (Table 3, entry 3), whereas the behaviour of N_L_N_L_‐**9 c**, was in line with (but somewhat lower to) the ee value expected for monomeric *endo‐*L‐**3 a** (N_L_), in which the major aldol adduct was found to be (2*S*,1’*R*)‐**12** (entry 4). When the configurations of the components of the organocatalytic dimers were opposite to each other, the observed ee's were found to be negligible. This was the case of X_L_X_D_ **‐9 d** and N_L_N_D_ **‐9 e** (entries 5 and 6). Dimers of mixed configuration showed an enantioselectivity close to that it could be expected from the trend determined by the respective components. Thus, organocatalysts X_L_N_L_ **‐9 f** and N_L_X_L_ **‐9 g**, in which the monomers point to opposite configurations, showed low enantioselectivities, although higher than those expected from the estimated ee_av_ values (Table 3, entries 7 and 8). When the configuration of the components pointed to the preferential formation of the same enantiomer, the observed enantiocontrol was close to the expected ee_av_ value. This is the case of organocatalysts X_L_N_D_ **‐9 h** and N_L_X_D_ **‐9 i** (entries 9 and 10). Interestingly, blockade by *N‐*methylation of one of the possible catalytic sites responsible for the formation of the corresponding enamine transferred all the enantiocontrol to the remaining active site. This behaviour was observed in *N‐*methylated organocatalysts X_L_X_D_^Me^‐**9 j** and X_L_^Me^X_D_‐**9 l** (Table 3, entries 11–14), which were shown to be efficient aldol catalysts with enantiocontrols identical to those observed for the respective monomers, instead of the low to negligible enantiocontrol observed for X_L_X_D_ **‐9 d** and N_L_N_D_ **‐9 e** (entries 5 and 6, see above).

**Table 3:** Catalytic aldol reaction between cyclohexanone **10 a** and pentafluoro‐benzaldehyde **11** catalysed by dipeptides **9**.^[a]^

Entry	Catalyst	Time [h]	*anti:syn* ^[b]^	Yield^[c]^ [%]	ee^[d]^ [%]	ee_av_^[e]^ [%]
1^[f]^	*exo‐*L‐**3 a** (X_L_)	<1	95 : 5	75	89	–
2^[f]^	*endo‐*L‐**3 a** (N_L_)	<1	96 : 4	83	−81	–
3^[g]^	X_L_X_L_ **‐9 b**	8	93 : 7	86	85	89
4^[g]^	N_L_N_L_ **‐9 c**	24	96 : 4	91	−65	−81
5^[g]^	X_L_X_D_ **‐9 d**	16	92 : 8	84	9	0
6^[g]^	N_L_N_D_ **‐9 e**	32	87 : 13	52	0	0
7^[g]^	X_L_N_L_ **‐9 f**	8	94 : 6	84	31	4
8^[g]^	N_L_X_L_ **‐9 g**	27	94 : 6	80	35	4
9^[g]^	X_L_N_D_ **‐9 h**	36	94 : 6	72	84	85
10^[g]^	N_L_X_D_ **‐9 i**	16	98 : 2	82	−88	−85
11^[g]^	X_L_X_D_^Me^‐**9 j**	48	88 : 12	93	90	89
12^[h]^		16	92 : 8	85	88	89
13^[g]^	X_L_^Me^X_D_‐**9 l**	64	91 : 9	68^i^	−82	−81
14^[h]^		24	97 : 3	84	−85	−85

[a] Reactions were monitored by ^19^F NMR and were stirred at room temperature until total consumption of aldehyde **11**. [b] *Anti*:*syn* ratios were measured by ^19^F NMR of crude reaction mixtures. [c] Yields refer to pure aldol adducts. [d] Enantiomeric excesses measured by HPLC correspond to the major *anti*‐diastereomer (2*R*,1’*S*)‐**12**. [e] Average enantiomeric excess from the data obtained for the corresponding monomers (entries 1 and 2). [f] Reaction carried out in 30 mol% of monomeric catalyst and TFA. [g] 3 mol% of catalyst load was employed. [h] 6 mol% of catalyst load was employed. [i] 98 % conversion was achieved.

**Scheme 6:**
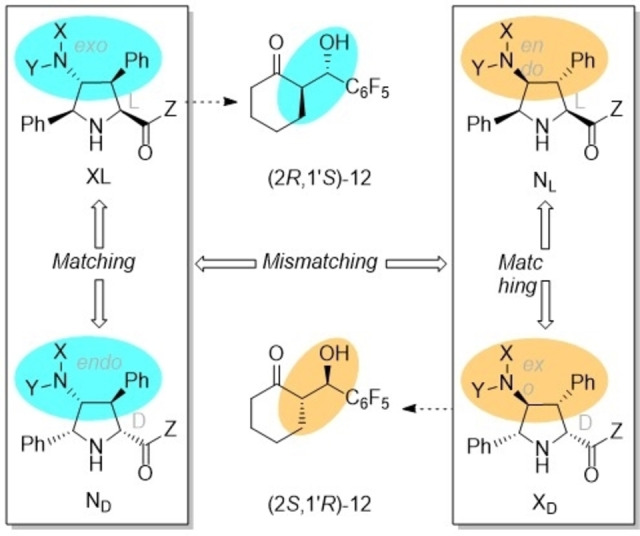
Matching and mismatching between the e*xo/endo* diastereomers and L/D enantiomers of dimeric catalysts **9** in aldol reactions.

These results indicate that both active sites of dimers **9** do participate in the aldol reaction, although in some cases the first unit is somewhat more active than the second one. The additive character of enantiocontrol associated with the *NH*‐active sites present in the catalyst correlates approximately with the matching or mismatching of the X/L diastereoselectivities and the L/D configurations of the catalytic units of these γ‐dimers **9** (Scheme 6). Finally, it is interesting to note that the observed stereocontrol is determined by the chiral centres of the distal parts of the organocatalytic units (Scheme 6) instead of the chiral environment proximal to the catalytic sites.

**Scheme 7:**
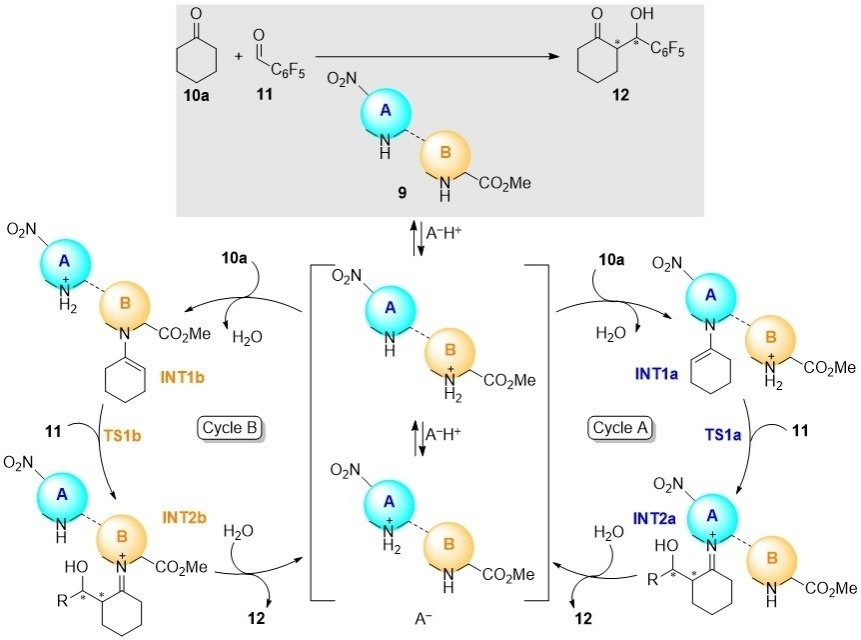
Catalytic cycles associated with dimeric organocatalysts **9** with two active sites in aldol reactions.

After establishing the main aspects that determine the stereocontrol of γ‐dimers **9**, we analysed the kinetic activity of these organocatalysts in the **10 a**+**11→12** reaction. In principle, for organocatalysts **9** possessing two comparable active sites, two independent catalytic cycles can be envisaged, as it is shown in Scheme 7. The combined catalytic activities of γ‐homodimers X_L_X_L_‐**9 b** and N_L_N_L_‐**9 c**, as well as those associated with heterodimers X_L_N_L_‐**9 f**, and N_L_X_L_‐**9 g**, were studied by ^19^F NMR at room temperature and using a catalytic load of 30 mol% in the presence of TFA (30 mol%). For instance, in the case of X_L_X_L_‐**9 b**, the reaction was monitored by ^19^F NMR, as it is gathered in Figure 2.

In order to simplify the kinetic analyses, the reactions were conducted under pseudo‐first order conditions, with a **10 a**:**11** ratio of 60 : 1. Under this large excess of cyclohexanone **10 a**, the reaction rate can be estimated as

(1) $$rate=-\frac{d\left[11\right]}{dt}\approx k_{obs}\left[11\right]$$

**Figure 3:**
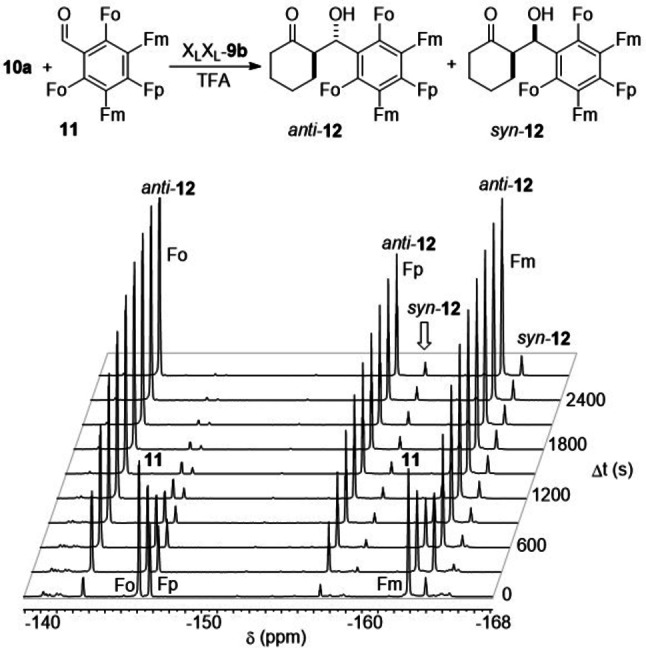
Evolution in time of ^19^F NMR spectra of the aldol reaction between cyclohexanone **10 a** and pentafluorobenzaldehyde **11** to give adducts *anti‐* **12** and *syn‐* **12**, catalysed by X_L_X_L_‐**9 b**. *Ortho‐*, *meta‐* and *para*‐fluoro substituents are denoted as Fo, Fm and Fp, respectively. The signal associated with the acidic additive (TFA), also used as internal reference, does not appear in the spectral window gathered in the figure.

Where $k_{obs}$ is the observed pseudo‐first order rate constant. The solution of Equation (1) was expressed in terms of the fluorine integral signals on ^19^F NMR. As internal reference, the signal provided by the additive TFA was used. For each fluorine atom signal (*ortho*, *meta* and *para*, see Figure 3) in the pentafluorophenyl moiety of **11**, the progress of the reaction was monitored by means of Equation 2:

(2) $$ln\left(\frac{I_{t}^{i}}{I_{t}^{TFA}}\right)-ln\left(\frac{I_{0}^{i}}{I_{0}^{TFA}}\right)=-k_{obs}t$$

**Figure 4:**
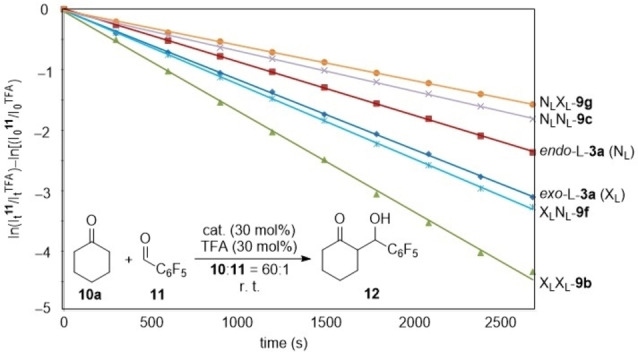
Pseudo‐first order linear plots of the **10 a**+**11→12** reaction in the presence of monomeric and dimeric organocatalysts.

In Equation (2), *I*_0_^i^ and *I*_t_^i^ refer to the integrals of the different fluorine atoms in **11** (where *i*=*ortho*, *meta* or *para*) at initial and instant times, t_0_ and t, respectively. In each experiment, the measurements were averaged for the three signal systems associated to *ortho*, *meta* and *para* positions of fluorine atoms in the pentafluorophenyl groups (standard deviations and error calculations are reported in the Supporting Information). Figure 4 shows the linear plots obtained by means of equation 2 for **10 a**+**11→12** reaction in the presence of selected monomeric and dimeric catalysts.

As previously found by our group,^[24]^ monomeric catalyst *exo‐*L‐**3 a** (X_L_) is more active than its *endo* congener *endo‐*L‐**3 a** (N_L_) (Table 4, entries 1 and 2). γ‐Homodimeric organocatalyst X_L_X_L_ **‐9 b** shows a larger *k*_obs_ value, but does not reach a twice catalytic activity (Table 4, entry 3). In contrast, the other γ‐homodimeric analogue, N_L_N_L_ **‐9 c** has a lower pseudo‐first kinetic constant with respect to its monomeric congener *endo‐*L‐**3 a** (N_L_) (Table 4, entry 4). Concerning γ‐heterodimeric catalyst, X_L_N_L_ **‐9 f**, its measured *k*_obs_ value is still larger than found for *exo‐*L‐**3 a** (X_L_), but lower than that of its γ‐homodimeric congener X_L_X_L_ **‐9 b** (Table 4, entry 5). Finally, it is interesting to note that the alternative γ‐heterodimeric catalyst N_L_X_L_ **‐9 g** is the slowest one, its *k*_obs_ value being close to that found for its homodimeric analogue N_L_N_L_ **‐9 c** and lower than that measured for the monomeric parent congener *endo‐*L‐**3 a** (N_L_) (Table 4, entry 6).

**Table 4:** Measured pseudo‐first order kinetic constants^[a,b]^ for the **10 a**+**11→12** aldol reaction catalysed by selected monomeric and dimeric organocatalysts.

Entry	Catalyst	$k_{obs}$ (10^−4^ s^−1^)^[c]^
1	*exo‐*L‐**3 a** (X_L_)	11.29 (±0.30)
2	*endo‐*L‐**3 a** (N_L_)	8.83 (±0.16)
3	X_L_X_L_ **‐9 b**	16.68 (±0.58)
4	N_L_N_L_ **‐9 c**	6.65 (±0.24)
5	X_L_N_L_ **‐9 f**	13.36 (±0.54)
6	N_L_X_L_ **‐9 g**	5.72 (±0.06)

[a] Calculated by means of Equation (2) with a **10 a**:**11** ratio of 60 : 1. [b] All reactions were monitored by ^19^F NMR at 25 °C. [c] Errors were calculated from the standard deviations of the kinetic constants (see Ref. [35]).

We have performed computational studies in order to provide a rationale of the origins of our experimental results. Since we performed DFT analyses of the origins of stereocontrol in the monomers[^22^, ^24^] and the main features of organocatalyzed aldol and related reactions have been established based on the Houk‐List model,^[36]^ comparison with our results on organocatalytic γ‐dipeptides **9** would permit to assess the emergence of distinct features in the dipeptide level of complexity. According to the Houk‐List model, the main features of a proline‐catalysed aldol reaction are the following:

(i) Only one proline unit catalyses the reaction.^[36b]^

(ii) The critical step in terms of stereochemistry and kinetics is the C−C bond formation between the *ipso* carbon atom of the electrophilic aldehyde and the α‐carbon atom of the previously formed enamine intermediate. The enamine moiety adopts a conformation in which the formal double bond is close (proximal orientation) to the carboxy group of the proline unit.[^36a^, ^36c^, ^36d^]

(iii) The transition state (TS) associated with this step adopts a chair conformation around the C⋅⋅⋅C bond being formed, according to a Zimmerman‐Traxler‐like topology.[^36a^, ^36c^, ^36d^]

(iv) In this TS, the stabilization factors that determine the stereochemical outcome of the reaction are[^36a^, ^36c^, ^36d^, ^36f^] (a) the extent of planarity preservation of the enamine moiety, and (b) the electrostatic stabilization of the alkoxide moiety generated from the carbonyl group. Most of this electrostatic stability stems from degree of advancement of the proton transfer from the carboxylic acid of proline to the oxygen atom of the aldehyde, although an additional stabilisation involving this latter hydrogen atom and the nitrogen of the pyrrolidine ring (which is acquiring a partial positive charge) is not negligible. The remaining stabilization factors are related to the minimisation of other non‐covalent interactions.^[36f]^

Our reported calculations involving organocatalysts possessing densely substituted proline esters showed two additional features:

(v) The presence of 5‐aryl (or alkyl) groups in the pyrrolidine moiety favours enamine conformations in which the double bond adopts a distal geometry with respect to the carboxy group.[^22^, ^24^]

(vi) The preferred conformation of these unnatural proline units is that maximizes the number of equatorial substituents in the half‐chair pyrrolidine unit.[^24^, ^25^, ^26^]

In this research, our γ‐peptides **9** involve two proline units. Therefore, within a two proline‐one reaction scheme, two independent catalytic cycles involving enamine formation can be envisaged (Scheme 7). Since organocatalysts **9** include non‐protic ester groups, addition of an acidic component, TFA in our case, is required. Assuming similar basicities for the two pyrrolidine units, protonation of prolines A or B gives rise to the corresponding A and B‐catalytic cycles, in which enamine intermediates **INT1a**,**b** can be formed independently. From these intermediates, aldehyde **11** yields the corresponding iminium intermediates **INT2a**,**b** via transition structures **TS1a**,**b** (Scheme 7). Hydrolysis of these latter intermediates yields aldol adducts **12** together with the release of the catalyst.

**Figure 5:**
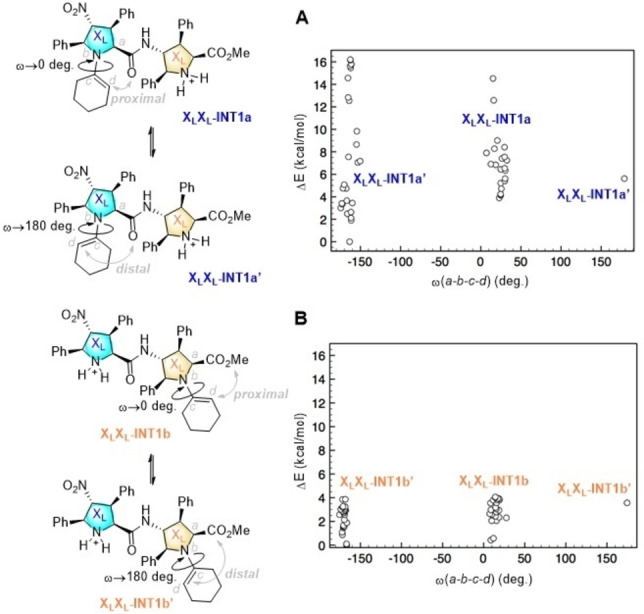
Molecular mechanics Monte Carlo simulations (OPLS 2004 force field) of enamine intermediates X_L_X_L_‐**INT1a** and X_L_X_L_‐**INT1b**, in which the relative energies of distal and proximal conformers, defined by the w(*a‐b‐c‐d*) dihedral angles (in absolute value) are shown.

The enamine‐based catalytic cycles gathered in Scheme 7 involve an additional factor, namely the distal or proximal orientation of the enamine double bond with respect to the carboxiamido group. To explore this feature, we analysed computationally the enamine intermediates **X_L_X_L_‐INT1a** and **X_L_X_L_‐INT1b** (Figure 5). Molecular Mechanics Monte Carlo (MM/MC) simulations (see the Computational Methods section) showed that, in effect, both intermediates adopt preferentially conformations in which the enamine moiety preserve the planar geometry of the N−C=C bonds. In the case of X_L_X_L_‐**INT1a**, there is a wide spectrum of possible proximal conformations, with ω(*a‐b‐c‐d*) angles close to 0 deg. (Figure 5A), although many distal conformations (ω≈180 deg.) are less energetic than their proximal congeners. When the enamine unit is formed in the B‐proline unit, the energy distribution of distal and proximal conformations in X_L_X_L_‐**INT1b** is much shorter and both conformers are very close in energy (Figure 5B). As a consequence of this initial computational analysis, both A and B cycles were explored taking into account the possible distal and proximal orientations of the reacting enamine moieties.

The **INT1a**,**b→TS1a**,**b→INT2a**,**b** elementary processes associated with the **10 a**+**11→12** reaction catalysed by X_L_X_L_‐**9 b** were analysed by means of the B3LYP DFT hybrid functional (see the Computational Methods section). Formation of the four possible enantiomers and diastereomers shown in Scheme 5 were evaluated, as well as the proximal and distal conformations of the intermediate enamines. Since the kinetic scenario was quite complex, instead of a computational Curtin‐Hammett analysis, we decided to calculate numerically the possible reaction paths associated with the stereocontrol of the reaction assuming an irreversible C−C bond formation. All the possible interaction pathways, kinetic constants and rate equations are reported in the Supporting Information. Only the structures and paths of lowest energy are discussed below.

**Figure 6:**
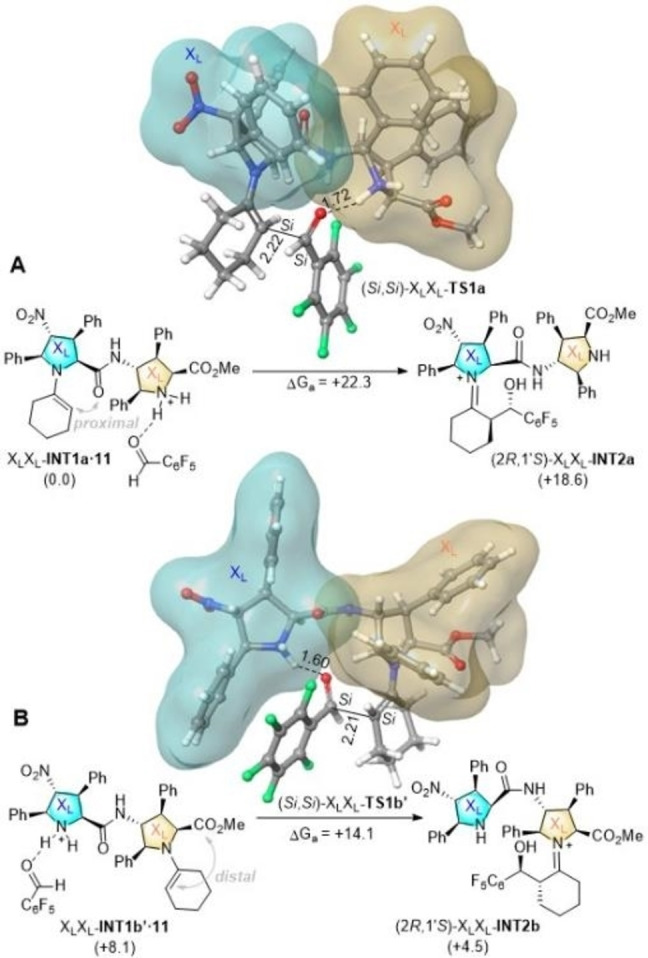
DFT free energy profiles (B3LYP‐D3/6‐31G* level of theory, at 298 K) for the C−C bond formation step associated with the **10 a**+**11→12** aldol reaction catalysed by X_L_X_L_ **‐9 b** γ‐dipeptide. Numbers in parentheses are relative Gibbs energies, in kcal/mol. Bond distances are given in Å.

In the case of the A cycle, the conformation of lowest energy resulting from the interaction between X_L_X_L_ **‐INT1a** and aldehyde **11** showed a proximal conformation (Figure 6A). The corresponding transition structure X_L_X_L_‐**TS1a** is associated with a (*Si*,*Si*) interaction between the enamine and aldehyde moieties, which results in the formation of intermediate X_L_X_L_‐**INT2a**, from which the catalytic A cycle proceeds towards the formation of *anti*‐aldol adduct (2*R*,1’*S*)‐**12**. This latter compound is the major product according to our experimental results. Transition structure X_L_X_L_‐**TS1a** is associated with a *like* (*lk*) topology, according to the Seebach‐Prelog nomenclature,^[37]^ in which both the enamine and aldehyde moieties interact along their respective *Si* prochiral faces. In contrast with the Houk‐List model, X_L_X_L_‐**TS1a** shows a geometry about the C⋅⋅⋅C bond being formed that corresponds to an extended boat geometry (Figure 6A). In addition, the electrophilicity of aldehyde **11** is enhanced via electrostatic interaction with the protonated X_L_ unit adjacent to the enamine‐X_L_ ensemble. This extended interaction pattern, together with the conformational restrictions imposed by the two densely substituted X_L_ units, result in the relative stabilization of X_L_X_L_‐**TS1a**.

As far as cycle B is concerned, the lowest energy path corresponds to the **X_L_X_L_‐INT1b’** ⋅ **11→** X_L_X_L_ **‐TS1b→** X_L_X_L_‐**INT2’b** elementary step (Figure 6B). In this case, the complex formed by enamine intermediate X_L_X_L_‐**INT1b’** and aldehyde **11** is more energetic (and kinetically less abundant) than the X_L_X_L_ **‐INT1a** ⋅ **11** reactive complex. It is interesting to note that this latter reactive complex is associated with a distal conformation of the enamine moiety, thus confirming that both distal and proximal conformations are kinetically accessible in these γ‐peptide catalysed reactions. Interestingly, both **X_L_X_L_‐INT1a** and **X_L_X_L_‐INT1b’** enamine complexes are practically isoenergetic. According to our calculations, **X_L_X_L_‐INT1b’** is 0.5 kcal/mol more stable that **X_L_X_L_‐INT1a**. We interpret this result concluding that the lower stability (and higher reactivity) of **X_L_X_L_‐INT1b’.11** reactive complex is due to the less than optimal interaction of this latter enamine intermediate with the electrophile. Also in this case, the transition structure that connects X_L_X_L_ **‐INT1b’** ⋅ **11** with iminium intermediate X_L_X_L_ **‐INT2’b** corresponds to a *lk* addition pattern via the *Si* prochiral faces of the enamine and aldehyde moieties. This results again in the preferential formation of aldol adduct (2*R*,1’*S*)‐**12**, thus providing another access to the major isomer via catalytic cycle B. Also in this case, X_L_X_L_ **‐INT1b’** shows an extended boat geometry, as well as a similar enhancement of the electrophilicity of **11**, which results in a quite low activation energy (Figure 6B).

**Figure 7:**
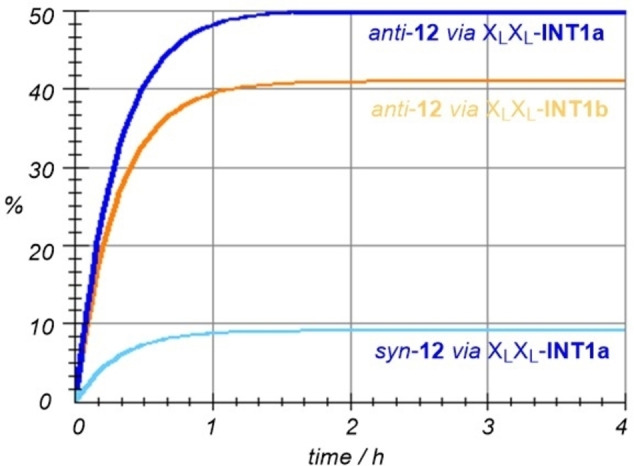
Numerical simulation of the three most relevant kinetic pathways involving *syn‐* and *anti‐*intermediates and catalytic cycles A and B gathered in Scheme 7.

Although the elementary steps discussed above correspond to the faster pathways, the whole scenario is very complex and therefore numerical simulations were performed including all intermediates and transition structures reported in the Supporting Information. The most relevant routes are shown in Figure 7. If the *anti* and *syn* routes are compared, our numerical simulations lead to an *anti:syn* ratio of 92 : 8, in excellent agreement with our experimental value of 93 : 7 (Table 3, entry 3). In addition, our calculations suggest that the relevance of cycles A and B is in an approximate ratio A : B=58 : 42. This means that the 50 : 50 ratio assumed in our estimate of the average ee's could be refined in favour of cycle A. This is in agreement with the very low, but not zero ee obtained with X_L_X_D_ **‐9 d** (Table 3, entry 5). The ee≈0 value obtained for N_L_N_D_ **‐9 e** (Table 3, entry 6) would require an analogous calculation to assess the contributions of A and B cycles, but this experimental result suggests that, in this latter case, the contribution of both cycles would be similar.

In summary, our experimental and computational studies on the aldol reaction with γ‐dipeptides **9** possessing one or two active sites indicate that the catalytic activities of both *NH‐*proline units are approximately additive. Of course, in the case of *N*‐methylated pyrrolidine components, their only possible contribution consists of electrophile activation via *N‐*protonation, the remaining *NH‐*unit being the only one able to form the corresponding enamine intermediates. On the basis of this additive scheme, the behavior of catalysts **9** in conjugate additions was studied.

### Conjugate Additions

**Scheme 8:**
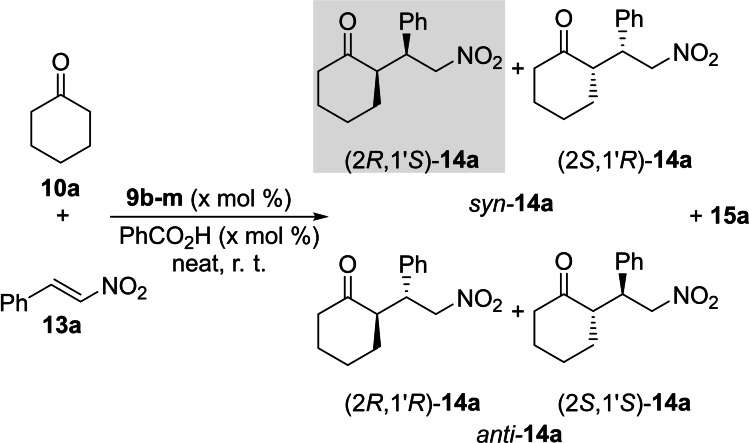
Catalysed Michael reaction between cyclohexanone **10 a** and (*E*)‐β‐nitrostyrene **13 a** to yield adducts **14 a**. *Syn* γ‐nitroketone (2*R*,1’*S*)‐**14 a** (highlighted in grey) is the major adduct under catalysis of X_L_X_L_ **‐9 b** (see Table 5). One lactam by‐product **15 a** was observed, in general with <20 % conversion under these conditions (see Scheme 10).

After studying the outcome of dimeric organocatalysts **9** in the aldol reaction, we also tested these dipeptide catalysts in another enamine mediated organocatalyzed reaction such as the conjugate addition reaction between cyclohexanone **10 a** and (*E*)‐β‐nitrostyrene **13 a** as model Michael acceptor to yield Michael adducts **14 a** (Scheme 8). In contrast with the aldol reaction, there is no monomeric reference catalysts with respect to γ‐dipeptides **9** since monomeric nitroprolines of type **3** do not catalyse this reaction. In contrast, monomeric 4‐aminoproline esters **5** catalyse this conjugate addition by means of the primary amino group of these latter compounds,^[26]^ which is not present in dimers **9**. Therefore, since these latter γ‐dipeptides do catalyse this Michael addition reaction (see below), this ability can be considered as an emergent property, which is presented and discussed below in this section.

The reaction between cyclohexanone **10 a** and nitrostyrene **13 a** was carried out in the presence of benzoic acid as additive to evaluate the catalytic behaviour of dimers **9**. In all the studied cases, the *syn* adducts predominate, with high to excellent diastereomeric ratios, whereas the reaction times, conversions and yields vary considerably depending on the dimer used. In all cases, the evolution of conversion with time resulted to be considerably slower than in aldol reactions. For instance, the **10 a**+**13 a→14 a** reaction catalysed by X_L_X_L_ **‐9 b** required ca. two days for complete reaction when a catalytic loading of 20 % mol was used (Table 5, entry 1). Total conversion in several hours required a considerable 40 % catalytic loading and using benzoic or trifluoroacetic acid (Table 5, entries 3 and 4, respectively). Our results also indicate that *endo*‐units are less active in terms of catalytic power. Thus, when this Michael addition was carried out in the presence of N_L_N_L_ **‐9 c** under a catalytic loading of 20 %, the conversion was not complete after seven days of reaction (entry 5). Mixed *exo‐endo* γ‐dipeptides **9** resulted in even longer reaction times (Table 5, entries 8–11). Finally, it is interesting to note that, when the first pyrrolidine unit was blocked for enamine catalysis via *N‐*methylation (X_L_^Me^X_L_‐**9 m** organocatalysts**)**, a 40 % catalytic loading was required to achieve high conversion, although with low ee Table 5, entry 18). In contrast, *N‐*methylation of the second pyrrolidine unit (X_L_X_L_^Me^‐**9 k** catalyst) resulted in a faster reaction with a catalytic loading of 20 % and in the presence of salicylic acid (Table 5, entry 17). Closely related X_L_X_D_^Me^‐**9 j** dipeptide (Table 5, entry 15) shows lower catalytic activity since it requires a 40 % catalytic loading for complete conversion, but after two days of reaction, to yield a lower ee. However, 40 % catalytic loadings (Table 5, entries 15 and 16) also resulted in higher conversions for adduct **15 a** (see below). These results with partially enamine‐blocked organocatalysts show the relevance of remote effects in this conjugate reaction.

**Table 5:** Catalytic aldol reaction between cyclohexanone **10 a** and (*E*)‐β‐nitrostyrene **13 a** catalysed by dipeptides **9**.^[a]^

Entry	Catalyst	*X* ^[b]^ [mol %]	Time [d]	Conv. [%]	*syn:anti* ^[c]^	Yield^[d]^ [%]	ee^[e]^ [%]
1	X_L_X_L_ **‐9 b**	20	2	>99	85 : 15	79	65
2		30	1	>99	89 : 11	82	78
3		40	4 h	>99	91 : 9	75	82
4^[f]^		40	16 h	>99	89 : 11	75	93
5	N_L_N_L_ **‐9 c**	20	7	76	93 : 7	59	‐17
6	X_L_X_D_ **‐9 d**	20	4	>99	94 : 6	71	‐46
7	N_L_N_D_ **‐9 e**	20	7	71	95 : 5	56	6
8	X_L_N_L_ **‐9 f**	20	6	84	90 : 10	78	55
9	N_L_X_L_ **‐9 g**	20	3	>99	84 : 16	89	46
10	X_L_N_D_ **‐9 h**	20	13	72	82 : 18	57	24
11	N_L_X_D_ **‐9 i**	20	3	86	90 : 10	78	‐70
12		30	19 h	>99	94 : 6	71	‐75
13		40	16 h	>99	97 : 3	76	‐81
14^[f]^		40	16	>99	98 : 2	89	‐88
15	X_L_X_D_^Me^‐**9 j**	40	2	>99	89 : 11^[g]^	61	75
16	X_L_X_L_^Me^‐**9 k**	40	1	>99	90 : 10^[h]^	60	82
17^[i]^		20	16 h	>99	98 : 2	91	96
18	X_L_^Me^X_L_‐**9 m**	40	16 h	>99	87 : 13	91	65

**Scheme 9:**
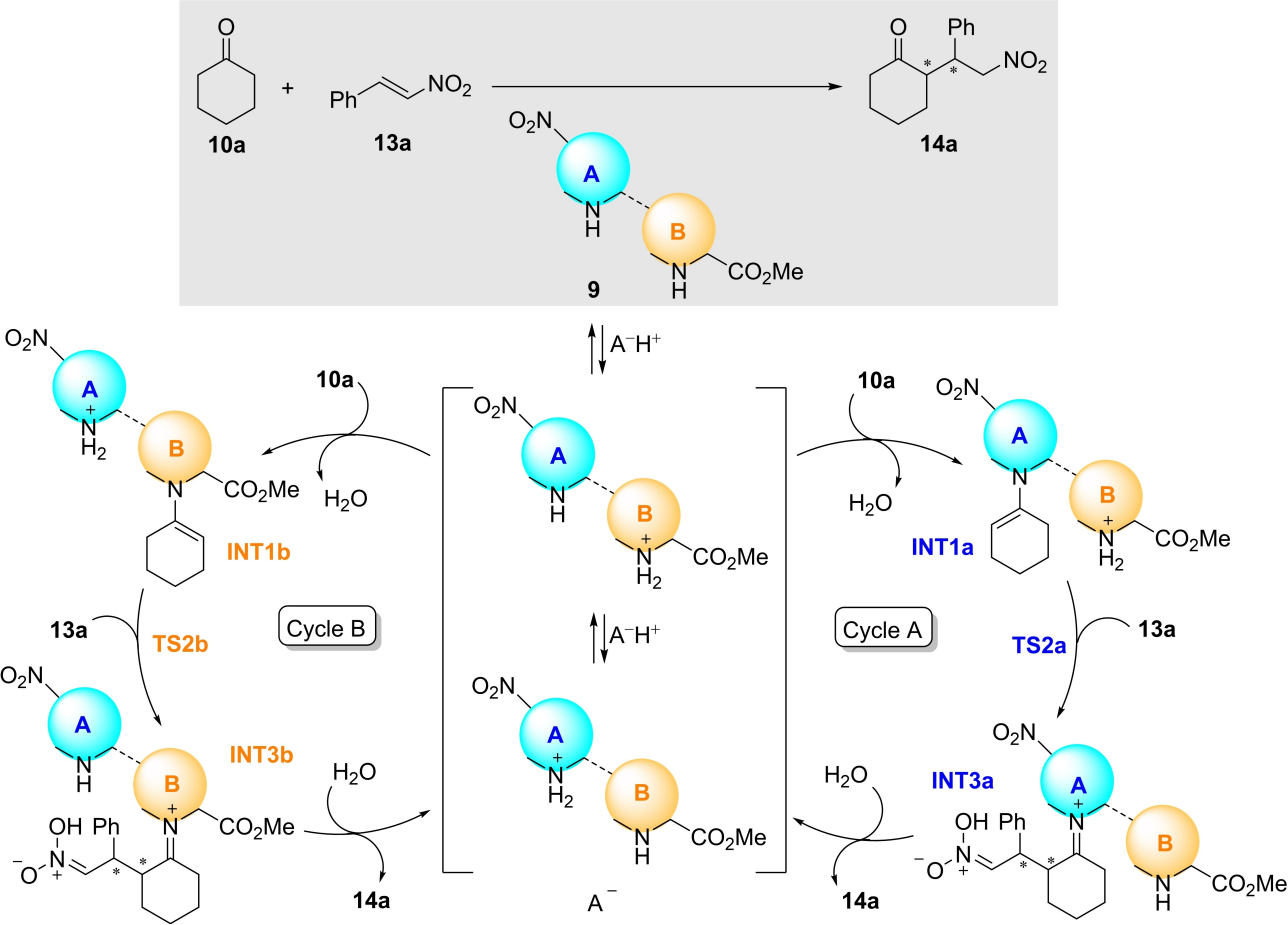
Catalytic cycles associated with dimeric organocatalysts 9 with two active sites in conjugate reactions.

[a] Reaction conditions are indicated in Scheme 8. Conversions were measured by ^1^H NMR of crude reaction mixtures. Conversions to γ‐lactam **15** product were ca. 20 % (see Scheme 9). [b] X stands for the catalysts and acid catalytic load (see Scheme 8). [c] S*yn:anti* ratio was measured by ^1^H NMR of crude reaction mixtures. [d] Yields refer to isolated pure Michael adducts. [e] Enantiomeric excesses measured by HPLC are referred to *syn*‐diastereomer (2*R*,1’*S*)‐**14 a** (see Scheme 8). [f] TFA (40 % mol) was used instead of benzoic acid [g] The **14 a**:**15 a** ratio of the crude reaction mixture was 69 : 31. [h] The **14 a**:**15 a** ratio of the crude reaction mixture was 78 : 22. [i] Salicylic acid (40 % mol) was used instead of benzoic acid.

The analysis of the enantioselectivity of organocatalysts **9** in terms of the contribution of their first and second pyrrolidine units is quite complex. Among the different catalysts and reaction conditions gathered in Table 5, the best ee's were 96 % (with X_L_X_L_^Me^‐**9 k** entry 17) and −88 % (with N_L_X_D_ **‐9 i**, entry 14). Organocatalyst X_L_X_L_ **‐9 b** showed a remarkable ee of 93 %. This latter dimer was chosen for a computational study involving the C−C bond‐forming step associated with this conjugate addition.

**Figure 8:**
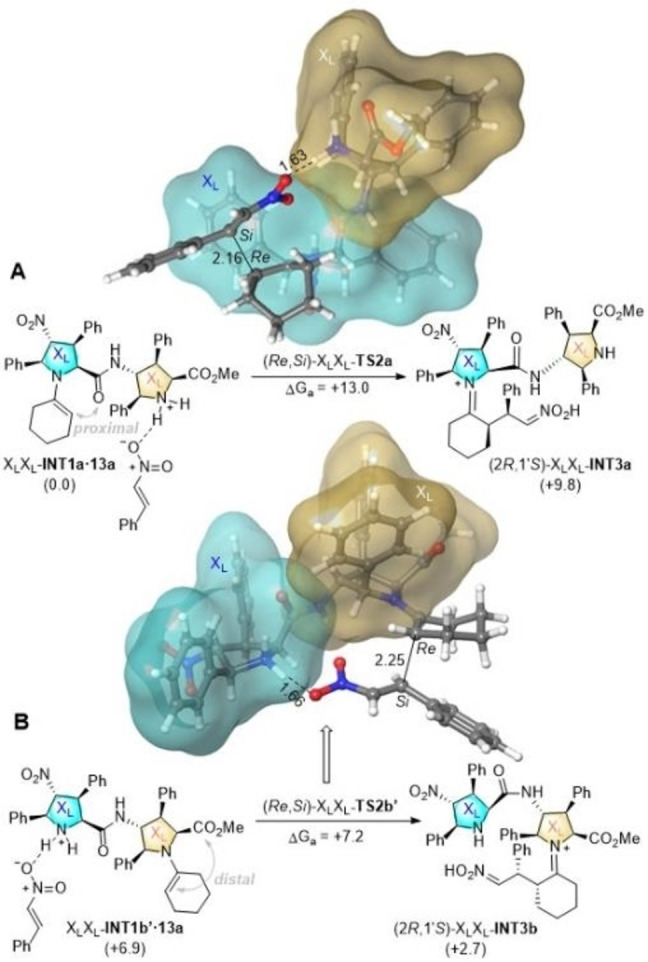
DFT free energy profiles (B3LYP‐D3/6‐31G* level of theory, at 298 K) for the C−C bond formation step associated with the **10 a**+**13 a→14** M reaction catalysed by **X_L_X_L_‐9 b** γ‐dipeptide. Numbers in parentheses are relative Gibbs energies, in kcal/mol. Bond distances are given in Å.

Figure 7 includes two representative examples of the elementary steps that determine the stereochemistry of the **10 a**+**13 a→14 a** reaction catalysed by X_L_X_L_‐**9 b**. In Figure 8A, the less energetic transformation associated with cycle A is shown. This elementary step starts from the reactive enamine complex X_L_X_L_‐**INT1a** ⋅ **13 a** and leads to the stereochemistry‐determinant imino intermediate (2*R*,1’*S*)‐X_L_X_L_ **‐INT3a** via saddle point (*Re*,*Si*)‐X_L_X_L_ **‐TS2a**. In the starting intermediate, the enamine moiety is in a proximal disposition with respect to the second X_L_ unit. Also in this case, reactive complex **X_L_X_L_**‐**INT1a** ⋅ **13 a** is less energetic and more reactive that its congener **X_L_X_L_**‐**INT1b’** ⋅ **13 a**, thus confirming that in this latter reactive complex the interaction between the enamine **X_L_X_L_**‐**INT1b’** and electrophile **13 a** is less efficient than in the case of reactive complex **X_L_X_L_**‐**INT1a** ⋅ **13 a** (see above). The corresponding saddle point exhibits an extended conformation, in which the α‐carbon atom of the enamine interacts with the β‐carbon of Michael electrophile **13 a**, whose nitro group is close to the protonated amine of the second X_L_ unit, thus introducing a LUMO lowering that facilitates the conjugate reaction.

**Figure 9:**
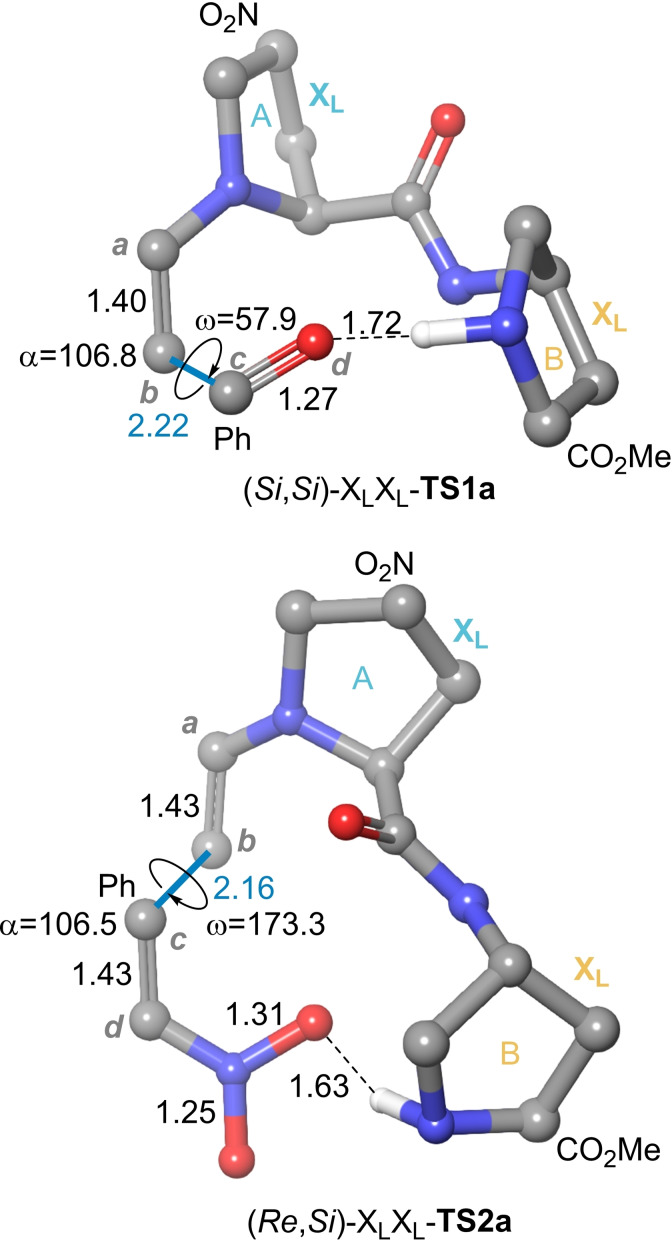
Compared basic geometries of transition structures (*Si*,*Si*)‐X_L_X_L_ **‐TS1a** and (*Re*,*Si*)‐X_L_X_L_ **‐TS2a**, associated with the formation of major isomers of aldol and conjugated reactions catalysed by γ‐dipeptide X_L_X_L_ **‐9 b**. Angles a and w (in absolute value) are defined as a=*a‐b‐c* and w=*a‐b‐c‐d* and are given in deg. Bond distances are given in Å.

Comparison of the geometry of (*Re*,*Si*)‐X_L_X_L_‐**TS2a** saddle point with that optimized for the minimum energy aldol transition structure (*Si*,*Si*)‐X_L_X_L_‐**TS1a** (Figure 9) shows that in the Michael transition structure there is an antiperiplanar disposition of the enamine and nitroalkene C=C units, the associated dihedral angle being of ca. 180 deg. In contrast, in the case of the aldol congener, the enamine C=C and aldehyde C=O units give rise to a *gauche* conformation, the corresponding dihedral angle being of ca. 60 deg. It is remarkable that the catalytic action of X_L_X_L_‐**3 b** relies on the HOMO uprising effect of the A unit of the catalyst on the nucleophilic enamine and the LUMO lowering effect of the protonated B unit. Aside this LUMO lowering associated with a two‐electron interaction, there is a lowering of the Pauli repulsion^[38]^ between the electrophile and the catalyst, promoted by the sterically less demanding geometry associated with the =O⋅⋅⋅HN(H,Me)(+) interaction. This flexibility permits to expand the organocatalytic activity of these dimers with respect to the corresponding monomers, thus giving rise to emergent catalytic properties since the dimeric catalysts accept substrates that cannot interact efficiently with the monomers. However, this occurs at the cost of a relatively lower preorganization, which introduces an entropic penalty that, in turn, generates a higher activation free energy. This results in feasible – but relatively slow – reactions.

The less energetic elementary step via the B unit of X_L_X_L_ **‐9 b** corresponds to the interaction between the distal conformation of enamine X_L_X_L_ **‐INT1b’** and nitroalkene **13 a** to form the corresponding reactive complex, which lies ca. 7 kcal/mol above its A‐analogue (Figure 8B). The saddle point (*Re*,*Si*)‐X_L_X_L_‐**TS2b’** that connects these local minima is quite similar to that shown in Figure 8A and yields intermediate (2*R*,1’*S*)‐X_L_X_L_‐**INT3b**. Both reaction paths gathered in Figure 8 lead to the same Michael cycloadducts. It is also remarkable that these elementary steps are endergonic, which facilitates the progression of these iminium intermediates along the catalytic cycle once the stereochemistry‐determining step has been accomplished.

**Figure 10:**
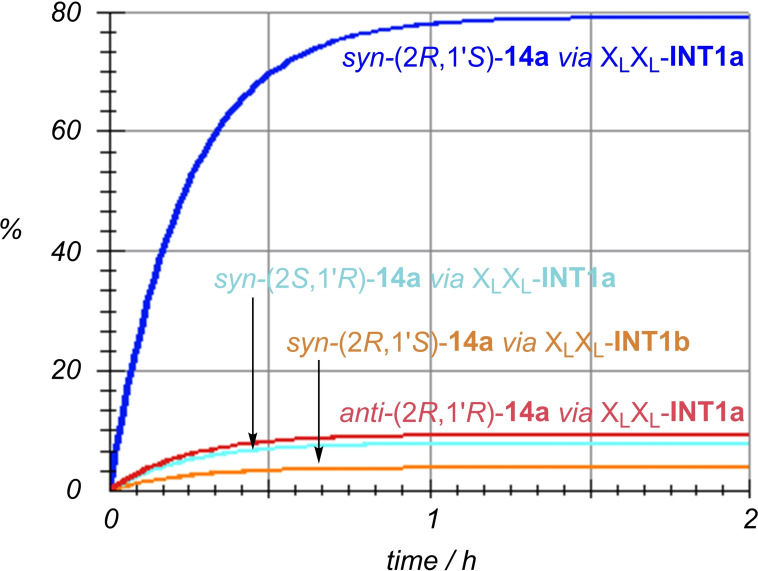
Numerical simulation of the four most relevant kinetic pathways involving *syn‐* and *anti‐*intermediates and catalytic cycles A and B gathered in Scheme 9.

We computed the possible stereochemical pathways associated with the A and B cycles shown in Scheme 9 (see the Supporting Information). After numerical integration of the corresponding kinetic equations, we obtained only four relevant reaction paths, whose kinetic profiles are collected in Figure 10. Inspection of this Figure reveals that, in effect, the cycle A is much more efficient than B, in contrast with the results computed for the aldol reaction (Figure 7). These results are in line with our experimental observations for X_L_X_L_‐**3 b** and *N‐*methylated catalytic dimers (see above). In addition, the results gathered in Figure 9 lead to a computed *syn*:anti ratio of 90 : 10, in excellent agreement with the 85 : 15—91 : 09 ratios observed experimentally (Table 5, entries 1–4). As far as the enantiomeric control is concerned, the computed *ee* value is 87 %, which is also within the experimental range of 65–93 % observed for X_L_X_L_‐**3 b** under different conditions (Table 5, entries 1–4).

**Scheme 10:**
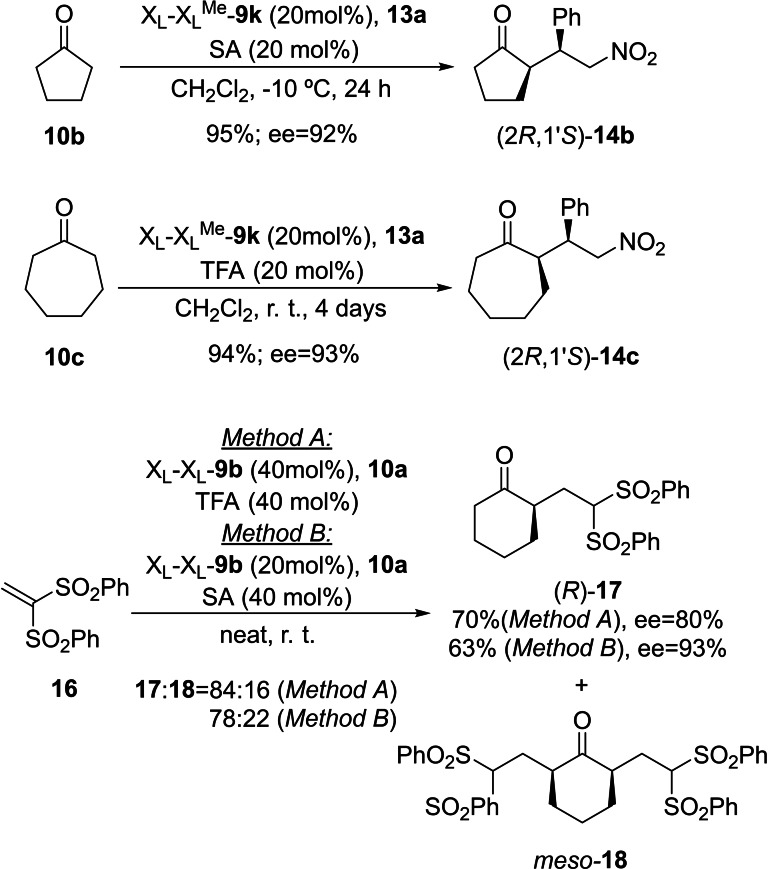
Scope of the reaction between ketones **10 a**–**c** and Michael acceptors **13 a** and **16** catalysed by γ‐dipeptides X_L_X_L_‐**9 b** and X_L_X_L_^Me^‐**9 m**. SA: salicylic acid.

We explored the scope of these conjugate reactions catalysed by γ‐dipeptides **9** by testing ketones **10 b**,**c** as nucleophiles and disulfone **16** as Michael acceptor (Scheme 10). After experiments including different organocatalysts, acid additives and reaction conditions, we observed that X_L_X_L_^Me^‐**9 m** was the most efficient catalyst in the conjugate addition of cyclopentanone **10 b** and cycloheptanone **10 c** with (*E*)‐β‐nitrostyrene **13 a**. In particular, both the chemical yields and ee's were excellent, in contrast with the low selectivity shown by monomeric diamino organocatalysts **5**.^[26]^ In the case of disulfonyl electrophile **16**, dipeptide X_L_X_L_‐**9 b**, in the presence of either TFA and salicylic acid, resulted to be the most efficient one in terms of chemical yield and enantiocontrol. However, formation of *meso*‐diadduct **18**, whose structure was confirmed by X‐ray diffraction analysis (see Supporting Information), could not be avoided. These results indicate that the scope of this reaction in the presence of γ‐dipeptides **9** can be expanded by checking different catalysts, whose behaviour can be optimized depending on the nucleophile‐electrophile combination.

### Formation of γ‐lactams 15

**Scheme 11:**
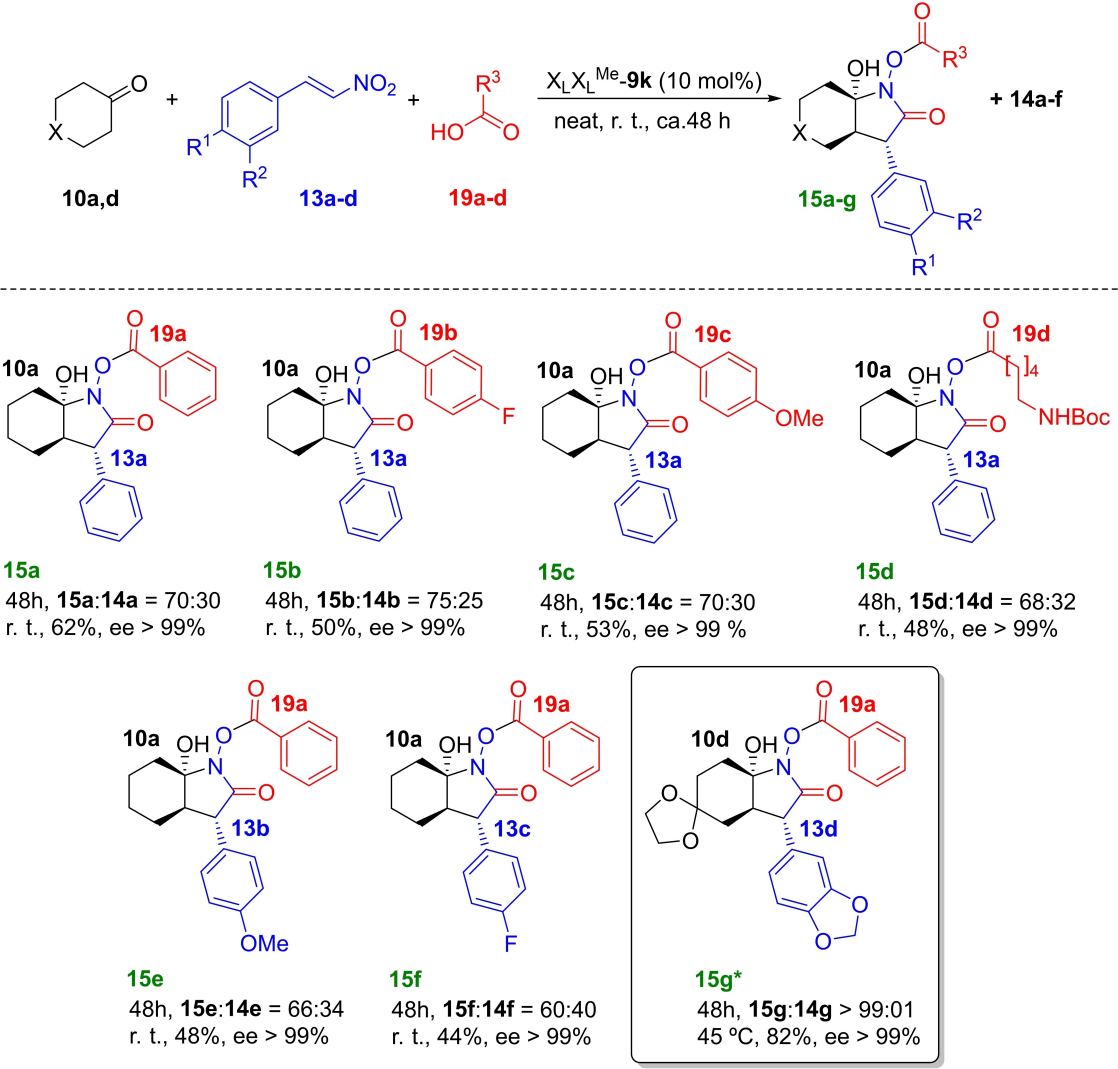
Three‐component synthesis of γ‐lactams 15 catalysed by *N‐*methylated γ‐dipeptide X_L_X_L_^Me^‐9 m. (*) Adduct **15 g**, a key intermediate in the synthesis of (+)‐pancracine, is highlighted. The reaction was performed at 45 °C with 20 mol% of catalytic load.

In the conjugate additions discussed in the previous section, the presence of another compound was detected in the crude reaction mixtures. This compound was identified as the bicyclic γ‐lactam **15 a**, which was previously observed in the three‐component reaction between **10 a**, **13 a** and an equimolar amount of benzoic acid **16 a** (Scheme 11) in the presence of monomeric nitroproline *exo‐*L‐**3 a**.^[27]^ Mechanistic studies showed that in this reaction the two carboxy and carboxamide C=O groups in adducts **15** stem from the carboxylic acid **19** and that the stereocontrol in the three‐component reaction was complete. Within this context, we considered it interesting to explore the competition between the Michael addition and the three‐component cyclisation catalysed by γ‐dipeptides **9**, since these latter compounds catalyse conjugate additions and *exo‐*L‐**3 a** does not.

Initial experiments with X_L_X_L_‐**9 b** catalyst showed a poor conversion to γ‐lactams **15** despite using equimolar amounts of acids **19**. Since our previous experimental and computational studies suggested that the A‐unit of the organocatalysts **9** is the most active one, we tested the behaviour of dimer X_L_X_L_^Me^‐**9 m**, in which the *N*‐methylated B‐unit cannot act as the HOMO rising enamine unit. The results of these experiments are collected in Scheme 11. According to these results, γ‐dipeptide X_L_X_L_^Me^‐**9 m** produces adducts **15 a**–**f** in **14 : 15** ratios of ca. 70 : 30 with catalytic loadings of only 10 mol% and with moderate yields. These results include cyclohexanone **10 a**, aryl and alkyl carboxylic acids **19 a**–**d** and nitrostyrenes **13 a**–**c**. The exception was bulkier cyclohexanone **1 b**, which only produced the three‐component adduct **15 g**, although both the reaction temperature and the catalytic loading were slightly higher. However, also in this case the diastereo‐ and enantiocontrol were essentially complete. It is noteworthy that γ‐lactam **15 g** is a key intermediate in the synthesis of (+)‐pancracine.

## Conclusions

Our experimental and computational studies on the catalytic activity of γ‐dipeptides in aldol and conjugate reactions permit to conclude that:

- These dimeric organocatalysts exhibit an additive behavior in aldol reactions, with key transition structures showing gauche electrophile‐nucleophile moieties. Both densely substituted proline units participate in the catalytic cycle in comparable extents. When two *N*‐unsubstituted units are present in the dimeric catalysts, both proline rings can act as either enamine or ammonium active sites.
- In the case of conjugate additions, dimers **9** show catalytic activity. Since the corresponding 4‐nitroproline esters do not promote this reaction, the organocatalytic behavior of the dimers can be considered as an emergent property with respect to the monomeric units. In addition, the unnatural proline closer to the nitro group has higher activity, the other pyrrolidine unit being a LUMO‐activating group. The key TS in this reaction requires an antiperiplanar disposition between the C=C groups of the enamine unit and the Michael acceptor.
- These observations are confirmed by blocking one unnatural proline as enamine catalytic unit by *N*‐methylation: the *N*‐methylated proline ring acts as a LUMO‐lowering unit via interaction between the NHMe(+) protonated unit and the heteroatom of the electrophile.
- Formation of three‐component adducts is observed in the reaction between cyclohexanones, nitrostyrenes and carboxylic acids. This reaction, also observed in the monomeric state, is favored by *N*‐methylation of the second densely substituted proline unit.

## Computational Methods

All the reported DFT calculations were carried out using the B3LYP hybrid gradient‐corrected functional^[39]^ with the D3 correction^[40]^ for dispersion forces. The polarized split‐valence 6–31G(d) basis set^[41]^ was used within the Gaussian suite of programs.^[42]^ Harmonic analyses were performed on the fully optimized structures by means of the rigid rotor approximation at 298.14 K. Local minima showed positive definite Hessian matrices (NMAG=0), whereas transition structures showed one and only one imaginary frequency (NIMAG=1) associated with nuclear motion along the reaction coordinate under study.^[43]^ Numerical kinetic simulations were performed by means of the FACSIMILE program.^[44]^ Molecular Mechanics and Monte Carlo (MM/MC) simulations were carried out with the OPLS_2004 force field^[45]^ as implemented in the MacroModel program.^[46]^ All the molecular structures were drawn by using the Maestro interface.^[47]^

## Experimental Section

### General procedure for the synthesis of monomers

#### General procedure for the synthesis of exo‐cycloadducts 3^[22]^

A solution of **NH−D−EhuPhos** or **NH−L−EhuPhos** (0.015 mmol, 9.27 mg) and Cu(CH_3_CN)_4_PF_6_ (0.014 mmol, 5.2 mg) in 1.0 mL of dry THF was stirred at −20 °C for 15 min. Then, a solution of the corresponding imine **2 a** or **2 b** (0.45 mmol) in 1.0 mL of solvent, triethylamine (0.023 mmol, 3.2 μl) and the nitroalkene **1** (0.50 mmol) in 1.0 mL of solvent were successively added. The course of the reaction was monitored by TLC and, once the starting material was consumed, the mixture was filtered through a celite pad and the filtrate was concentrated under reduced pressure. The residue was purified by flash chromatography on silica gel (EtOAc/hexane) to yield the corresponding *exo‐*cycloadducts **3 a**–**b**.

#### General procedure for the synthesis of endo‐cycloadducts 3^[22]^

A solution of **NMe−L−EhuPhos** or **NMe−D−EhuPhos** (9.48 mg, 0.015 mmol) and Cu(CH_3_CN)_4_PF_6_ (5.2 mg, 0.014 mmol) in 1.0 mL of dry THF was stirred at −60 °C for 15 min. Then, a solution of the corresponding imine **2 a** or **2 b** (0.45 mmol) in 1.0 mL of solvent, triethylamine (3.2 μl, 0.023 mmol) and the nitroalkenes **1** (0.50 mmol) in 1.0 mL of solvent were successively added. The reaction was monitored by TLC and, once the starting material was consumed, the mixture was filtered through a celite pad and the filtrate was concentrated under reduced pressure. The residue was purified by flash chromatography on silica gel (EtOAc/hexane, 1/2) to yield the corresponding *endo‐*cycloadducts **3**.

#### General procedure for the methylation of exo‐3 a^[26]^

Pyrrolidine *exo*‐L/D‐ **3 a** (500 mg, 1.53 mmol) was dissolved in 10 mL of 88 % aqueous formic acid. 10 mL of 35 % aqueous formaldehyde were added and the reaction mixture was heated at 100 °C for two hours. After cooling to room temperature, the acidic solution was basified with saturated K_2_CO_3_ solution from which a precipitated appeared. Then, this solution was diluted with H_2_O and extracted with CH_2_Cl_2._ The combined organic layers were dried over Na_2_SO_4_, filtered and concentrated under reduced pressure. The crude mixture was filtered through a plug of silica eluting with ethyl acetate affording the pure product.

### General procedure for the synthesis of γ‐amino esters 5 and 7^[26]^

A solution of the corresponding 4‐nitro cycloadducts **3 a**–**b** or **6 a** (1 mmol) in 100 mL of methanol was pumped at 1 mL/min through the H‐Cube® Hydrogenation Reactor using a Raney/Nickel CatCart® as catalyst. The pressure of the system was set to 20 bars and the temperature to 65 °C. After all the reaction mixture had passed through the reactor, the solvent was reduced to dryness. The crude mixture was filtered through a plug of silica eluting with ethyl acetate affording the pure products **5 a**–**b** or **7 a** respectively.

### General procedure for the synthesis of γ‐nitro amino acids 4 and 8

#### General procedure for hydrolysis in basic conditions

To a solution of *exo*‐**3 a** or *exo*‐**6 a** (1.0 mmol) in acetone (3 mL) stirred at room temperature, a solution of sodium hydroxide (88 mg, 2.2 mmol) in water (3 mL) was added. The reaction mixture was stirred for 16 h. Then, the solution was cooled to 0 °C and acidified with 2 N HCl to pH ≅ 2. A solid precipitated from the solution. This solid was filtered, washed with water and dried under vacuum to afford the desired products *exo*‐**4 a** or *exo*‐**8 a** respectively.

#### Simultaneous methylation‐hydrolysis of exo‐D/L‐3 b

See general procedure for the methylation of *exo*‐**3 a**.

#### General procedure for hydrolysis in acidic conditions

To a solution of *endo*‐L/D‐**3 b** (326 mg, 1.0 mmol) in dichloromethane (15 mL) stirred at room temperature, trifluoroacetic acid (8 mL) was added. The reaction mixture was stirred for 16 h. Then, the solvent was evaporated *in vacuo*. The obtained crude product was purified by precipitation to afford the desired compound *endo*‐L/D‐**4 a**.

### General procedure for the synthesis of γ‐dipeptides

To a stirred solution of the corresponding amine (0.8 mmol) in CH_2_Cl_2_ (10 mL) was added acid (1.0 mmol), PyBOP (1.0 mmol) and by diisopropyl ethyl amine (1.4 mmol). The resulting mixture was then stirred until completion of the reaction. Then, the reaction mixture was diluted with CH_2_Cl_2_, washed with a 1 M HCl solution, saturated aqueous NaHCO_3_, brine and then dried over Na_2_SO_4_. Evaporation of the solvent followed by column chromatography eluting with EtOAc/hexane provided the corresponding dipeptides **9 a**–**k**.

### Additional procedures for the synthesis of 9 l–m

To a stirred solution of the corresponding amine *exo*‐L/D‐**5 a** (80 mg, 0.27 mmol) in 8 mL of dichloromethane, *exo*‐L‐**7 a** (104 mg, 0.31 mmol), HATU (104 mg, 0.31 mmol) and diisopropyl ethyl amine (48 μL, 0.31 mmol) were added. The resulting mixture was then stirred until completion of the reaction. Then, the reaction mixture was diluted with CH_2_Cl_2_, washed with a 1 M HCl solution, saturated aqueous NaHCO_3_, brine and then dried over Na_2_SO_4_. Filtration and evaporation of the solvent followed by column chromatography eluting with EtOAc/hexane provided the corresponding dipeptides **9 l**–**m**.

### General procedure for the synthesis of β‐hydroxyketones 12

The corresponding aldehyde **11** (0.25 mmol) was dissolved in neat ketone **10** or DCM (1.5 mL, 15.3 mmol, 61.2 equiv.). The organocatalyst (0.0125–0.075 mmol, 0.05–0.3 equiv.) was added, followed by trifluoroacetic acid (75.0 μmol, 0.3 equiv.). The resulting mixture was stirred at room temperature, diluted with ethyl acetate, washed with 0.1 M (pH 7) phosphate buffer solution, dried onto sodium sulfate, filtered and concentrated under reduced pressure. The afforded crude product was purified by flash chromatography over silica gel using ethyl acetate:hexane system as eluent to provide the corresponding β‐hydroxyketone *anti*‐**12**.

### General procedure for the Michael reaction

#### Synthesis of γ‐nitroketone 14 a

A reaction mixture of catalyst **9** (0.005–0.03 mmol), acid (0.02–0.04 mmol), ketone **10 a**–**c** (0.8 mmol) and nitroalkene **13 a** (0.1 mmol) was stirred at room temperature. The progress of the reaction was monitored by TLC (EtOAc/hexane, 1/3). After consumption of the nitroalkene, unreacted ketone was evaporated under reduced pressure. The afforded crude product was purified by flash chromatography over silica gel using ethyl acetate:hexane system as eluent to provide the corresponding γ‐nitrketone *syn*‐**14 a**.

#### Synthesis of γ‐nitroketones 14 b–c

A reaction mixture of dimeric catalyst (0.02 mmol), salicylic acid or TFA (0.02 mmol), ketone **11 b**,**c** (0.10 mmol) and nitroalkene **13 a** (0.11 mmol) in DCM was stirred at −10 °C or room temperature until total consumption of the nitroalkene **13 a**. Afterwards, the crude mixture was evaporated under reduced pressure and purified by flash column chromatography on silica gel (1 : 2 EtOAc:hexane).

#### Synthesis of (R)‐2‐(2,2‐bis(Phenylsulfonyl)ethyl)cyclohexan‐1‐one 17

A reaction mixture of dimeric catalyst (0.02–004 mmol), salicylic acid or TFA (0.04 mmol), ketone **10 a** (0.8 mmol) and (ethene‐1,1‐diyldisulfonyl)dibenzene **16** (0.1 mmol) was stirred at room temperature. The progress of the reaction was monitored by TLC (EtOAc/hexane, 1/3). After consumption of **16**, unreacted ketone was evaporated under reduced pressure. The afforded crude product was purified by flash chromatography over silica gel using ethyl acetate:hexane system as eluent to provide the corresponding adduct **17**.

### General procedure for the synthesis of lactams

A reaction mixture of nitroalkene **13** (0.1 mmol), ketone **10** (0.8 mmol), the corresponding carboxylic acid **19** (0.11 mmol) and catalyst **9 j**–**k** (0.01 mmol) was stirred at room temperature. The progress of the reactions was monitored by TLC with EtOAc/hexane elution mixtures. After consumption of the nitroalkene, the crude product was purified by column chromatography over silica gel using EtOAc/hexane system as eluent to provide the Michael addition product first followed by the lactam product (elution order). TLC plates were stained with vanillin. Michael products showed blue colour and lactam products produced pink spots.

### Crystallographic data

Deposition Numbers 2090672 (for X_L_X_L_‐**9 a**), 1451833 (for X_L_X_L_^Me^‐**9 k**) and 2090670 (for *meso‐* **18**) contain the supplementary crystallographic data for this paper. These data are provided free of charge by the joint Cambridge Crystallographic Data Centre and Fachinformationszentrum Karlsruhe Access Structures service.

## Conflict of interest

The authors declare no conflict of interest.

## Supporting information

As a service to our authors and readers, this journal provides supporting information supplied by the authors. Such materials are peer reviewed and may be re‐organized for online delivery, but are not copy‐edited or typeset. Technical support issues arising from supporting information (other than missing files) should be addressed to the authors.

Supporting Information
