# Supplementary material for: Additive and Emergent Catalytic Properties of Dimeric Unnatural Amino Acid Derivatives: Aldol and Conjugate Additions
Source: Chemistry. 2021 Oct 13;27(63):15671–87. doi: 10.1002/chem.202102394 (PMC9293019; doi:10.1002/chem.202102394)
Supplement: Supplementary file 1 — Supporting Information [file CHEM-27-15671-s001.pdf]

Chemistry–A European Journal

Supporting Information

Additive and Emergent Catalytic Properties of Dimeric Unnatural Amino Acid Derivatives: Aldol and Conjugate Additions

María de Gracia Retamosa, Andrea Ruiz-Olalla, Maddalen Agirre, Abel de Cózar, Tamara Bello, and Fernando P. Cossío*

TABLE OF CONTENTS

1 General Remarks S2
2 Synthesis of ligands NH-D/L-EhuPhos and NMe-D/L-EhuPhos S3
2.1 General procedure for the synthesis of NH-D/L-EhuPhos S3
2.2 General procedure for the synthesis of NMe-L/D-EhuPhos S4
3 Synthesis of densely substituted unnatural L-Proline derived catalysts S4
3.1 General procedure for the (3+2) cycloaddition reactions leading to pyrrolidines **3a** .. S5
3.1.1 *Exo* cycloadducts S5
3.1.2 *Endo* cycloadducts S7
3.2 General procedure for the *N*-methylation of cycloadducts **3** S8
3.3 General procedure for the hydrogenation of the nitro groups of cycloadducts **3** S9
3.4 General procedure for the hydrolysis of the ester groups of cycloadducts **3** S11
3.4.1 Basic conditions S11
3.4.2 Simultaneous methylation-hydrolysis of cycloadduct *exo*-L-**3b** S12
3.4.3 Acidic conditions S12
3.5 General procedure for the coupling reaction between 4-amino-pyrrolidines and 2-carboxy-pyrrolidines S12
4 Procedures for asymmetric C-C bond forming transformations S19
4.1 Organocatalytic asymmetric aldol reactions S19
4.2 Organocatalytic asymmetric Michael reactions S20
4.3 Organocatalytic enantioselective lactamization reaction S23
5 Pseudo-first order linear plots of organocatalyzed reactions S30
6 Standard deviations and error calculations S32
7 NMR spectra S33
8 X-Ray diffraction structures S65
8.1 Dimer X_LX_L-**9a** S65
8.2 Dimer X_LX_L-**9k** S65
8.3 2,6-bis(2,2-bis(Phenylsulfonyl)ethyl)cyclohexan-1-one (meso-**18**) S66
9 DFT calculations S67
9.1 Computational studies on Aldol reactions S67
9.2 Computational studies on Michael reactions S71
9.3 Computational data S74
10 References S139

1 General Remarks

Unless otherwise stated, reagents and substrates were purchased from commercial suppliers and were used without further purification. Cyclohexanone **10** was freshly distilled on thermally activated 4 Å molecular sieves before use. Catalysts **NMe-L/D-EhuPhos** and **NH-L/D-EhuPhos** were prepared following our previously described procedure.[1] Imines **2a,b** and nitroalkene **13d** are known compounds and were synthesized following reported procedures.[2] Compounds **3a-b**, **4a**, **5a**, **6a** and **7a** have been described in previous works from our group.[1,3] Adducts **12**, **14**, **15** and **17** are known compounds (See the Supporting Information for additional details). TLC was performed on silica gel 60 F254, using aluminum plates and visualized with UV lamps, potassium permanganate or vanillin stain. Flash chromatography was carried out on columns of silica gel 60 (230−400 mesh). Hydrogenation reactions were performed with a flow reactor equipped with a Raney-Nickel or Palladium on carbon cartridge. Hydrogen gas was generated electrochemically. Optical rotations were measured using a polarimeter with a thermally jacketed 5 cm cell at approximately 20 °C, and concentrations (c) are given in g/100 mL. FT-IR spectra were recorded with a spectrophotometer equipped with a single-reflection ATR module; wavenumbers are given in cm^{-1}. HRMS analyses were carried out using the electron impact (EI) mode at 70 eV or by Q-TOF using electrospray ionization (ESI) mode. ^{1}H-NMR spectra were recorded at 400 or 500 MHz for ^{1}H-NMR and 75 or 100 MHz for ^{13}C-NMR with ^{1}H- decoupling, using $CDCl_3$ as the solvent and referenced at 7.26 ppm and 77.16 ppm, respectively. The data are reported as s = singlet, d = doublet, t = triplet, m = multiplet or unresolved, br s = broad signal, coupling constant(s) in Hz, integration. All melting points are uncorrected. The absolute configurations of the known products were determined by comparing optical rotation values with the literature data.The HPLC chromatograms of the racemic and enantiomerically enriched products were performed using Daicel Chiralpak IA, IB, IC, AD-H, AS-H and OD-H columns.

2 Synthesis of ligands NH-D/L-EhuPhos and NMe-D/L-EhuPhos

Catalysts **NMe-L/D-EhuPhos** and **NH-L/D-EhuPhos** were prepared following our previously described procedure.[1]

2.1 General procedure for the synthesis of NH-D/L-EhuPhos

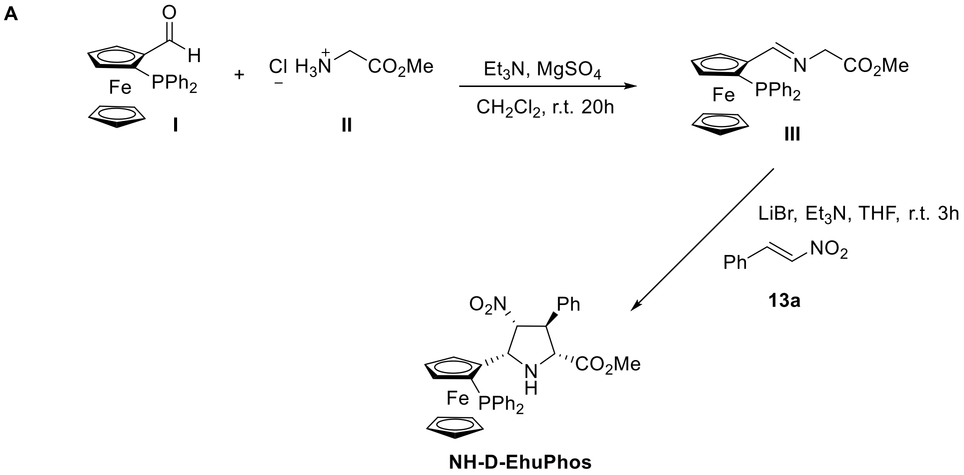

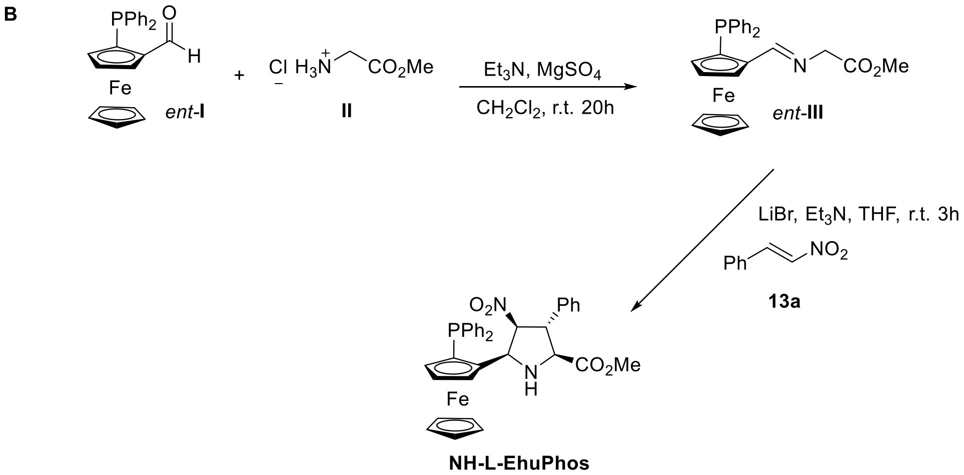

Synthesis of (S_P) and (R_P)-2-(Diphenylphosphino)-[(2-methoxy-2oxoethyl)-iminomethyl]ferrocene, III and ent-III

To a solution of glycine methyl ester hydrochloride **II** (0.38 g, 3.0 mmol) in dry CH_2Cl_2 (10 ml), $MgSO_4$ and Et_3N (0.4 ml, 3.0 mmol) were added at room temperature. The resulting mixture was stirred at the same temperature for 1h. Then the aldehyde **I** or *ent*-**I** (1.00 g, 2.5 mmol) was added, and the resulting mixture was stirred for 20 h. Then the $MgSO_4$ was filtered off and the organic layer washed three times with water, dried over anhydrous Na_2SO_4, and evaporated under reduced pressure to afford the pure product **III**

and *ent*-**III** as orange oils (1.08 g, 92% and 1.12 g, 96% respectively) which were used in the next step without further purification.

Synthesis of NH-D-EhuPhos and NH-L-EhuPhos

A mixture of imine **III** or *ent*-**III** (0.49 g, 1.0 mmol), nitroalkene **13a** (0.18 g, 1.2 mmol), triethylamine (0.14 ml, 1.0 mmol) and LiBr (0.14 g, 1.6 mmol) in dry THF (10 ml) was stirred at room temperature for 2 hours and 45 minutes. The resulting reaction mixture was treated with aqueous saturated solution of NH_4Cl and ethyl acetate. After usual work-up the crude product was purified by flash chromatography on silica gel (Ethyl acetate:hexanes 1:4). The compound **NH-D-EhuPhos** and **NH-L-EhuPhos** were obtained as yellow solids (0.42 g, 66 % and 0.39 g, 62%). Analytical and spectroscopic data were coincident with the previously reported material.[1]

2.2 General procedure for the synthesis of NMe-L/D-EhuPhos

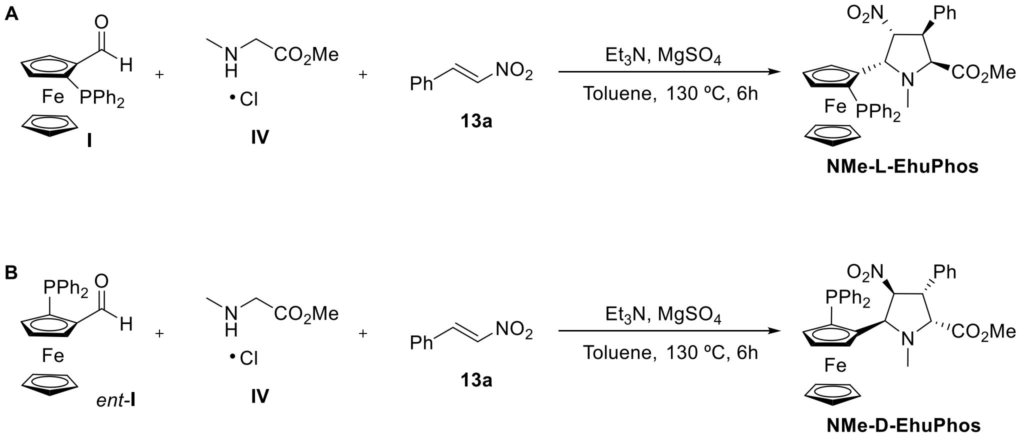

A mixture of aldehyde **I** or *ent*-**I** (0.52 g, 1.3 mmol), sarcosine methyl ester hydrochloride **IV** (0.24 g, 1.7 mmol), nitroalkene **13a** (0.25 g, 1.7 mmol), triethylamine (0.24 ml, 1.7 mmol) and MgSO4 in toluene (20 ml) was refluxed for 6 hours. After usual work-up, the resulting oily residue was purified by flash chromatography on silica gel (Ethyl acetate:hexanes 1:4) to yield **NMe-L-EhuPhos** and **NMe-D-EhuPhos** ligands as orange solids (0.46 g, 56 % and 0.44 g, 54% respectively). Analytical and spectroscopic data were coincident with the previously reported material.[1]

3 Synthesis of densely substituted unnatural L-Proline derived catalysts

Catalysts **NMe-L/D-EhuPhos** and **NH-L/D-EhuPhos** were prepared following our previously described procedure.[1]

3.1 General procedure for the (3+2) cycloaddition reactions leading to pyrrolidines 3a

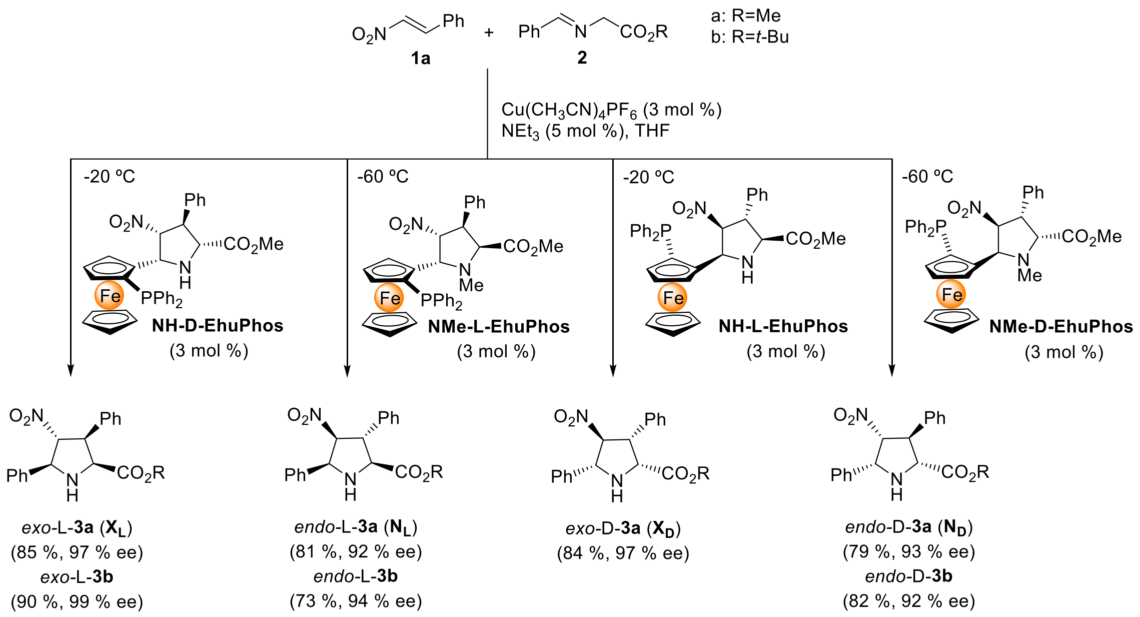

The different γ-nitroesters were prepared following our previously described procedure.[1]

3.1.1 *Exo* cycloadducts

A solution of chiral **NH-D-EhuPhos** or **NH-L-EhuPhos** (0.015 mmol) and $Cu(CH_3CN)_4PF_6$ (5.2 mg, 0.014 mmol) in 1.0 mL of dry THF was stirred at -20 ºC for 15 minutes. Then, a solution of imine (0.45 mmol) in 1.0 mL of solvent, triethylamine (3.2 ml, 0.023 mmol) and the corresponding nitroalkene (0.50 mmol) in 1.0 mL of solvent were successively added. The reaction was monitored by TLC and once the starting material was consumed the mixture was filtered through a celite pad and the filtrate was concentrated under reduced pressure. The residue was purified by flash chromatography on silica gel (ethyl acetate:hexanes 1:2) to yield the corresponding exo-cycloadduct. The enantiomeric excess was determined by comparison of the HPLC chromatogram recorded for the racemic mixture with the corresponding one of the enantiomerically enriched cycloadduct.

Nomenclature: For a better assignment of protons on ^{1}H NMR spectra, the IUPAC carbon order in pyrrolidines-type rings has been used. The following monomeric structures follow the same assignment pattern.

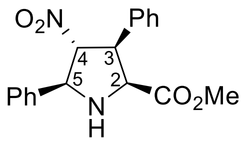

Methyl (2S,3S,4R,5S)-4-nitro-3,5-diphenylpyrrolidine-2-carboxylate (*exo*-L-**3a**). The expected product was obtained out of 10 parallel reactions from imine **2a** and nitroalkene **1a** using **NH-D-EhuPhos**. Yield: 125 mg, 85%, white solid. 97% ee after column chromatography and >99% ee after recrystallization in EtOAc/hexane mixture. Analytical and spectroscopic data were in good agreement with those reported in the literature.[1] ^{1}H NMR (500 MHz, $CDCl_3$) δ 7.58 – 7.55 (m, 2H, ArH), 7.47 – 7.18 (m, 8H, ArH), 5.22 (t, *J* = 8.1,1H, C^4H), 4.77 (d, *J* = 8.2, 1H, C^5H), 4.51 (d, *J* = 9.1, 1H, C^2H), 4.39 (t, *J* =8.5, 1H, C^3H), 3.29 (s, 3H, OMe), 2.75 (sa, 1H, NH). HPLC (Chiralcel IB, hexane/^{i}PrOH = 80/20, flow rate 1.0 mL/min, λ = 254 nm), t_R (major) = 6.92 min, t_R (minor) = 12.49 min; ee = 97%.

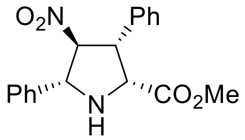

Methyl (2R,3R,4S,5R)-4-nitro-3,5-diphenylpyrrolidine-2-carboxylate (*exo*-D-**3a**).The title product was obtained out of 10 parallel reactions from imine **2a** and nitroalkene **1a** using **NH-L-EhuPhos**. Yield: 1.23 g, 84%, white solid. 94% ee after column chromatography and >99% ee after recrystallization in EtOAc/hexane crystallization mixture. Analytical and spectroscopic data were coincident with the previously reported material.[1] ^{1}H NMR (500 MHz, $CDCl_3$) δ 7.57 (d, *J* = 7.0 Hz, ArH), 7.45 – 7.36 (m, 3H, ArH), 7.34 – 7.27 (m, 3H, ArH), 7.26 – 7.22 (m, 2H, ArH), 5.22 (t, *J* = 8.1 Hz, C^4H), 4.77 (d, *J* = 7.9 Hz, C^5H), 4.51 (d, *J* = 8.9 Hz, C^2H), 4.39 (t, *J* = 8.5 Hz, C^3H), 3.30 (s, 3H, CO_2Me), 2.75 (bs, 1H, NH). HPLC (Chiralcel IB, hexane/^{i}PrOH = 80/20, flow rate 1.0 mL/min, λ = 254 nm), t_R (minor) = 9.6 min, t_R (major) = 18.2 min; ee = 94%.

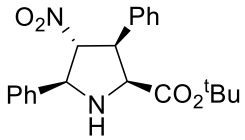

tert-Butyl (2S,3S,4R,5S)-4-nitro-3,5-diphenylpyrrolidine-2-carboxylate (*exo*-L-**3b**).The title product was obtained from imine **2b** and nitroalkene **1a** using **NH-D-EhuPhos**. Yield: 149 mg, 90%, white solid. >99% ee after column chromatography. Analytical and spectroscopic data were in good agreement with those reported in the literature.[1] ^{1}H NMR (500 MHz, $CDCl_3$) δ 7.55 (d, *J* = 7.2 Hz, 2H, ArH), 7.46 – 7.33 (m, 3H, ArH), 7.33 – 7.23 (m, 5H, ArH), 5.15 (t, *J* = 7.7 Hz, 1H, C^4H), 4.72 (d, *J* = 7.5 Hz, 1H, C^5H), 4.41 (d, *J* = 9.0 Hz, 1H, C^2H), 4.31 (dd, *J* =8.9, 7.4 Hz, 1H, C^3H), 2.72 (sa, 1H, NH), 1.05 (s, 3H, $(CH_3)_3$). HPLC (Chiralcel IB, hexane/^{i}PrOH = 90/10, flow rate 1.0 mL/min, λ = 254 nm) t_R (major) = 9.87 min; ee = >99%.

3.1.2 *Endo* cycloadducts

A solution of chiral ligand **NMe-L-EhuPhos** or **NMe-D-EhuPhos** (0.015 mmol) and $Cu(CH_3CN)_4PF_6$ (5.2 mg, 0.014 mmol) in 1.0 mL of dry THF was stirred at -60 ºC for 15 minutes. Then, a solution of imine (0.45 mmol) in 1.0 mL of solvent, triethylamine (3.2 μl, 0.023 mmol) and the corresponding nitroalkene (0.50 mmol) in 1.0 mL of solvent were successively added. The reaction was monitored by TLC and once the starting material was consumed the mixture was filtered through a celite pad and the filtrate was concentrated under reduced pressure. The residue was purified by flash chromatography on silica gel (ethyl acetate:hexanes 1:2) to yield the corresponding *endo*-cycloadduct. The enantiomeric excess was determined by comparison of the HPLC chromatogram recorded for the racemic mixture with the corresponding one of the enantiomerically enriched cycloadduct.

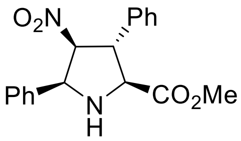

Methyl (2S,3R,4S,5S)-4-nitro-3,5-diphenylpyrrolidine-2-carboxylate (*endo*-L-**3a**).The expected product was obtained from imine **2a** and nitroalkene **1a** using **NMe-L-EhuPhos**. Yield: 119 mg, 81%, white solid. 92% ee after column chromatography and >99% ee after recrystallization in EtOAc/hexane recrystallization mixture. Analytical and spectroscopic data were in good agreement with those reported in the literature.[1] ^{1}H NMR (500 MHz, $CDCl_3$) δ 7.46 – 7.27 (m, 10H, ArH), 5.28 (dd, J = 6.4, 3.5, 1H, C^4H), 4.91 (d, J = 6.4, 1H, C^5H), 4.22 (dd, J = 7.2, 3.4, 1H, C^3H), 4.15 (d, J = 7.3, 1H, C^2H), 3.81 (s, 3H, CO_2Me), 2.90 (sa, 1H, NH). HPLC (Chiralcel IB, hexane/^{i}PrOH = 85/15, flow rate 1.0 mL/min, λ = 254 nm), t_R (major) = 14.84 min, t_R (minor) = 21.02 min; ee = 92%.

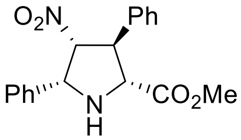

Methyl (2R,3S,4R,5R)-4-nitro-3,5-diphenylpyrrolidine-2-carboxylate (*endo*-D-**3a**). The expected product was obtained from imine **2a** and nitroalkene **1a** using **NMe-D-EhuPhos**. Yield: 243.8 mg, 83%, yellow oil. 94% ee after column chromatography. Analytical and spectroscopic data were coincident with the previously reported material.[1] ^{1}H NMR (500 MHz, $CDCl_3$) δ 7.41 – 7.28 (m, 10H, ArH), 5.25 (dd, J = 6.5, 3.5 Hz, 1H, C^4H), 4.89 (d, J = 6.0 Hz, 1H, C^5H), 4.24 (dd, J = 7.5, 3.5 Hz, 1H, C^3H), 4.14 (d, J = 7.5 Hz, 1H, C^2H), 3.79 (s, 3H, CO_2Me). HPLC (Chiralcel IB, hexane/^{i}PrOH = 85/15, flow rate 1.0 mL/min, λ = 254 nm), t_R (minor) = 15.05 min, t_R (major) = 21.56 min; ee = 94%.

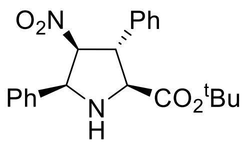

tert-Butyl (2S,3R,4S,5S)-4-nitro-3,5-diphenylpyrrolidine-2-carboxylate (*endo*-L-**3b**).[3] The title product was obtained from imine **2b** and nitroalkene **1a** using **NMe-L-EhuPhos**. Yield: 242.0 mg, 73%, white solid. 94% ee after column chromatography and >99% ee after recrystallization in EtOAc/hexane crystallization mixture. Analytical and spectroscopic data were in good agreement with those reported in the literature. ^{1}H NMR (500 MHz, $CDCl_3$) δ 7.42 – 7.23 (m, 10H, ArH), 5.30 (dd, J = 6.5, 4.0 Hz, 1H, C^4H), 4.92 (dd, J = 10.8, 6.7 Hz, 1H, C^3H), 4.10 (dd, J = 7.3, 3.9 Hz, 1H, C^5H), 4.01 (t, J = 8.2 Hz, 1H, NH), 3.34 (t, J = 9.6 Hz, 1H, C^2H), 1.45 (s, 9H, $(CH_3)_3$). HPLC (Chiralcel IB, hexane/^{i}PrOH = 80/20, flow rate 1.0 mL/min, λ = 254 nm), t_R (minor) = 7.29 min, t_R (major) = 9.22 min; ee = 94%.

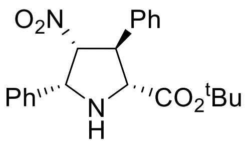

tert-Butyl (2R,3S,4R,5R)-4-nitro-3,5-diphenylpyrrolidine-2-carboxylate (*endo*-D-**3b**).[3] The title product was obtained from imine **2b** and nitroalkene **1a** using **NMe-D-EhuPhos**. Yield: 272.0 mg, 82%, pale yellow solid. 92% ee after column chromatography and >99% ee after recrystallization in EtOAc/hexane crystallization mixture. Analytical and spectroscopic data were coincident with the previously reported material. ^{1}H NMR (500 MHz, $CDCl_3$) δ 7.42 – 7.23 (m, 10H, ArH), 5.30 (dd, J = 6.5, 4.0 Hz, 1H, C^4H), 4.92 (dd, J = 10.8, 6.7 Hz, 1H, C^3H), 4.10 (dd, J = 7.3, 3.9 Hz, 1H, C^5H), 4.01 (t, J = 8.2 Hz, 1H, NH), 3.34 (t, J = 9.6 Hz, 1H, C^2H), 1.45 (s, 9H, $(CH_3)_3$). HPLC (Chiralcel IB, hexane/^{i}PrOH = 80/20, flow rate 1.0 mL/min, λ = 254 nm), t_R (major) = 7.10 min, t_R (minor) = 9.02 min; ee = 92%.

3.2 General procedure for the *N*-methylation of cycloadducts 3[4]

Pyrrolidine *exo*-L-**3a** or *exo*-D-**3a** (500 mg, 1.53 mmol) was dissolved in 10 mL of 88% aqueous formic acid. 10 mL of 35% aqueous formaldehyde were added and the reaction mixture was heated at 100ºC for two hours. After cooling to room temperature, the acidic solution was basified with saturated K_2CO_3 solution from which a precipitated appeared. Then, this solution was diluted with H_2O and extracted with CH_2Cl_2. The combined organic layers were dried over Na_2SO_4, filtered and concentrated under reduced pressure. The crude mixture was filtered through a plug of silica eluting with ethyl acetate affording the pure product.

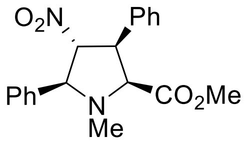

Methyl (2S,3S,4R,5S)-1-methyl-4-nitro-3,5-diphenylpyrrolidine-2-carboxylate (*exo*-L-**6a**). Yield: 406 mg, 78 %, dark yellow solid. Analytical and spectroscopic data were in good agreement with those reported in the literature.[3b] ^{1}H NMR (400 MHz, $CDCl_3$) δ 7.51 (d, *J* = 7.3 Hz, 2H, ArH), 7.45 – 7.21 (m, 8H, ArH), 5.00 (m, 1H, C^4H), 4.24 (dd, *J* = 9.3, 5.9 Hz, 1H, C^3H), 3.97 (d, *J* = 8.0 Hz, 1H, C^5H), 3.92 (d, *J* = 9.3 Hz, 1H, C^2H), 3.27 (s, 3H, CO_2Me), 2.33 (s, 3H, NMe).

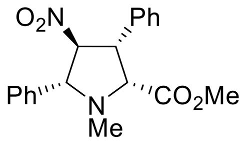

Methyl (2R,3R,4S,5R)-1-methyl-4-nitro-3,5-diphenylpyrrolidine-2-carboxylate (*exo*-D-**6a**). The expected product was obtained from *exo*-D-**3a** (100 mg, 0.31 mmol). Yield: 83 mg, 79%, white solid. Analytical and spectroscopic data were coincident with the previously reported material.[3b] ^{1}H NMR (400 MHz, $CDCl_3$) δ 7.50 (d, *J* = 7.3 Hz, 2H, ArH), 7.43 – 7.19 (m, 8H, ArH), 5.03 (m, 1H, C^4H), 4.27 (dd, *J* = 9.3, 5.9 Hz, 1H, C^3H), 3.96 (d, *J* = 8.0 Hz, 1H, C^5H), 3.90 (d, *J* = 9.3 Hz, 1H, C^2H), 3.25 (s, 3H, CO_2Me), 2.31 (s, 3H, NMe).

3.3 General procedure for the hydrogenation of the nitro groups of cycloadducts 3

A solution of the corresponding 4-nitro cycloadducts **3** (1 mmol) in 100 mL of methanol was pumped at 1 mL/min through the H-Cube® Hydrogenation Reactor using a Raney/Nickel CatCart® as catalyst. The pressure of the system was set to 20 bars and the temperature to 65ºC. After all the reaction mixture had passed through the reactor, the solvent was reduced to dryness. The crude mixture was filtered through a plug of silica eluting with ethyl acetate affording the pure product.

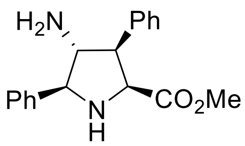

Methyl (2S,3R,4R,5S)-4-amino-3,5-diphenylpyrrolidine-2-carboxylate (*exo*-L-**5a**). The expected product was obtained from *exo*-L-**3a**. Yield: 266 mg, 90%, white solid. Analytical and spectroscopic data were in good agreement with those reported in the literature.[3b] ^{1}H NMR (500 MHz, $CDCl_3$) δ 7.70 - 7.64 (m, 2H, ArH), 7.42 (dd, *J* = 10.3, 4.7 Hz, 2H, ArH), 7.38 – 7.30 (m, 3H, ArH), 7.28 – 7.24 (m, 3H, ArH), 4.29 (d, *J* = 9.8 Hz, 1H, C^5H), 3.93 (d, *J* = 8.9 Hz, 1H, C^2H), 3.66 (dd, *J* = 10.3, 9.0 Hz, 1H, C^3H), 3.49 (t, *J* = 10.1 Hz, 1H, C^4H), 3.24 (s, 3H, CO_2Me), 1.65 (bs, 2H, NH_2).

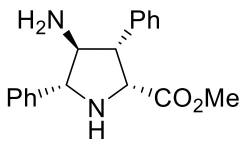

Methyl (2R,3S,4S,5R)-4-amino-3,5-diphenylpyrrolidine-2-carboxylate (*exo*-D-**5a**). The title product was obtained from *exo*-D-**3a**. (1.72 g, 5.29 mmol). Yield: 1.14 g, 73%, white solid. Analytical and spectroscopic data were coincident with the previously reported material.[3b] ^{1}H NMR (400 MHz, $CDCl_3$) δ 7.66 (d, *J* = 7.5 Hz, 2H, ArH), 7.42 (t, *J* = 7.5 Hz, 2H, ArH), 7.33 (dd, *J* = 11.8, 6.9 Hz, 3H, ArH), 7.25 (m, 3H, ArH), 4.27 (d, *J* = 9.7 Hz, 1H, C^5H), 3.91 (d, *J* = 8.8 Hz, 1H, C^2H), 3.64 (t, *J* = 9.7 Hz, 1H, C^3H), 3.48 (t, *J* = 10.1 Hz, 1H, C^4H), 3.23 (s, 3H, CO_2Me), 1.76 (s, 2H, NH_2).

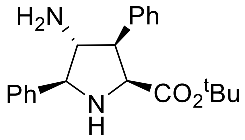

tert-Butyl (2S,3R,4R,5S)-4-amino-3,5-diphenylpyrrolidine-2-carboxylate (*exo*-L-**5b**). The title product was obtained from *exo*-L-**3b**. (368 mg, 1.0 mmol). Yield: 277 mg, 82%, white solid. m_p = 163 - 164 ºC. $[\alpha]_D^{25}$ = +67.3 (*c* 1.15, acetone). FTIR (neat, cm^{-1}) 1706, 1550, 1157, 697. ^{1}H NMR (500 MHz, $CDCl_3$) δ 7.63 (d, *J* = 7.3 Hz, 2H, ArH), 7.41 (t, *J* = 7.5 Hz, 2H, ArH), 7.36 – 7.29 (m, 6H, ArH), 4.18 (d, *J* = 9.9 Hz, 1H, C^5H), 3.87 (d, *J* = 8.8 Hz, 1H, C^2H), 3.62 (t, *J* = 9.4 Hz, 1H, C^3H), 3.45 (t, *J* = 9.9 Hz, 1H, C^4H), 1.04 (s, 9H, $C(CH_3)_3$). ^{13}C NMR (126 MHz, $CDCl_3$) δ 172.6, 141.0, 138.5, 129.0, 128.9, 128.6, 128.0, 127.5, 127.2, 81.1, 70.5, 64.1, 63.7, 56.9, 27.6. HRMS (ESI) for $C_{21}H_{27}N_2O_2$: calculated $[M+H]^+$: 339.2073. Found: 339.2074.

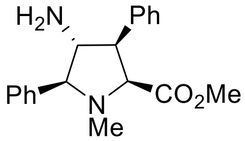

Methyl (2S,3R,4R,5S)-4-amino-1-methyl-3,5-diphenylpyrrolidine-2-carboxylate (*exo*-L-**7a**). The expected product was obtained from *exo*-L-**6a**. Yield: 230 mg, 74 %, bright yellow solid. Analytical and spectroscopic data were in good agreement with those reported in the literature.[3b] ^{1}H NMR (400 MHz, $CDCl_3$) δ 7.57 (d, *J* = 7.5 Hz, 2H, ArH), 7.40 (t, *J* = 7.5 Hz, 2H, ArH), 7.37 – 7.19 (m, 6H, ArH), 3.72 (d, *J* = 10.4 Hz, 1H, C^5H), 3.52 (t, *J* = 8.4 Hz, 1H, C^3H), 3.40 – 3.32 (m, 1H, C^4H), 3.21 (s, 3H, CO_2Me), 3.18 (d, *J* = 8.3 Hz, 1H, C^2H), 2.25 (s, 3H, NMe), 1.35 (bs, 2H, NH_2).

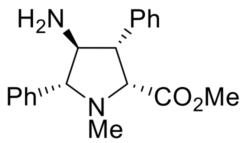

Methyl (2R,3S,4S,5R)-1-methyl-4-amino-3,5-diphenylpyrrolidine-2-carboxylate (*exo*-D-**7a**). The expected product was obtained from *exo*-D-**6a** (143 mg, 0.42 mmol). Yield: 98 mg, 75%, bright yellow solid. Analytical and spectroscopic data were coincident with the previously reported material.[3b] ^{1}H NMR (400 MHz, $CDCl_3$) δ 7.57 (d, *J* = 7.5 Hz, 2H, ArH), 7.40 (t, *J* = 7.4 Hz, 2H, ArH), 7.36 – 7.24 (m, 6H, ArH), 3.72 (d, *J* = 10.5 Hz, 1H, C^5H), 3.52 (t, *J* = 8.4

Hz, 1H, C^3H), 3.35 (t, J = 9.4 Hz, 1H, C^4H), 3.21 (s, 3H, CO_2Me), 3.18 (d, J = 8.5 Hz, 1H, C^2H), 2.25 (s, 3H, NMe).

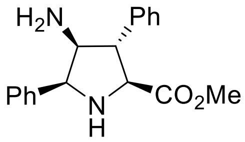

Methyl (2S,3S,4S,5S)-4-amino-3,5-diphenylpyrrolidine-2-carboxylate (*endo*-L-**5a**). The expected product was obtained from *endo*-L-**3a**. Yield: 207 mg, 70%, yellow syrup. Analytical and spectroscopic data were in good agreement with those reported in the literature.[3b] ^1H NMR (500 MHz, $CDCl_3$) δ 7.51 (d, J = 7.3 Hz, 2H, ArH), 7.44 – 7.31 (m, 8H, ArH), 4.63 (d, J = 6.1 Hz, 1H, C^5H), 4.12 (d, J = 7.8 Hz, 1H, C^2H), 3.74 (s, 3H, CO_2Me), 3.64 (t, J = 6.2 Hz, 1H, C^3H), 3.24 (t, J = 6.2 Hz, 1H, C^4H), 1.70 (bs, 2H, NH_2).

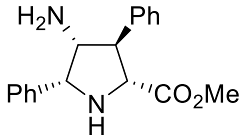

Methyl (2R,3R,4R,5R)-4-amino-3,5-diphenylpyrrolidine-2-carboxylate (*endo*-D-**5a**). The expected product was obtained from *endo*-D-**3a**. (720 mg, 2.20 mmol). Yield: 560 mg, 86%, yellow syrup. Analytical and spectroscopic data were coincident with the previously reported material.[3b] ^1H NMR (500 MHz, $CDCl_3$) δ 7.46 (d, J = 7.3 Hz, 2H, ArH), 7.42 – 7.24 (m, 8H, ArH), 4.57 (d, J = 6.1 Hz, 1H, C^5H), 4.07 (d, J = 7.8 Hz, 1H, C^2H), 3.69 (s, 3H, CO_2Me), 3.59 (t, J = 6.2 Hz, 1H, C^3H), 3.19 (t, J = 6.9 Hz, 1H, C^4H).

3.4 General procedure for the hydrolysis of the ester groups of cycloadducts 3

3.4.1 Basic conditions

To a solution of *exo*-L-**3a** or *exo*-L-**6a** (1.0 mmol) in acetone (3 mL) stirred at room temperature, a solution of sodium hydroxide (88 mg, 2.2 mmol) in water (3 mL) was added. The reaction mixture was stirred for 16 hours. Then, the solution was cooled to 0 ºC and acidified with 2N HCl to pH ≅ 2. A solid precipitated from the solution. This solid was filtered, washed with water and dried under vacuum to afford the desired product.

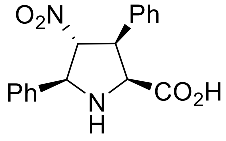

tert-Butyl (2S,3S,4R,5S)-4-nitro-3,5-diphenylpyrrolidine-2-carboxylic acid (*exo*-L-**4a**).[3a] The expected product was obtained from *exo*-L-**3a** (326 mg, 1.0 mmol). Yield: 259 mg, 83%, white solid. Analytical and spectroscopic data were in good agreement with those reported in the literature. ^1H NMR (500 MHz, acetone-d_6) δ 7.75 (d, J = 7.3 Hz, 2H, ArH), 7.53 (d, J = 7.6 Hz, 2H, ArH), 7.50 – 7.42 (m, 3H, ArH), 7.37 (t, J = 7.5 Hz, 2H, ArH), 7.35 – 7.28 (m, 1H, ArH), 5.72 (t, J = 9.2 Hz, 1H, C^4H), 5.13 (d, J = 9.2 Hz, 1H, C^5H), 4.80 (d, J = 9.4 Hz, 1H, C^2H), 4.66 (t, J = 9.4 Hz, 1H, C^3H).

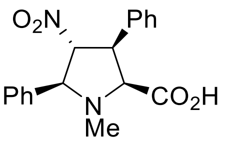

(2S,3S,4R,5S)-1-Methyl-4-nitro-3,5-diphenylpyrrolidine-2-carboxylic acid (*exo*-L-**8a**). The title product was obtained from *exo*-L-**6a** (100 mg, 0.29 mmol). Yield: 60 mg, 63%, orange syrup. $[\alpha]_D^{25}$ = +48.6 (*c* 0.33, $CHCl_3$). FTIR (neat, cm^{-1}) 1550, 700. ^{1}H NMR (400 MHz, $CDCl_3$) δ 7.59 – 7.37 (m, 5H, ArH), 7.32 (d, *J* = 4.2 Hz, 5H, ArH), 5.05 (t, *J* = 7.5 Hz, 1H, C^4H), 4.32 (dd, *J* = 9.8, 6.6 Hz, 1H, C^3H), 4.09 (d, *J* = 8.2 Hz, 1H, C^5H), 3.96 (d, *J* = 9.8 Hz, 1H, C^2H), 2.38 (s, 3H, NMe). ^{13}C NMR (101 MHz, $CDCl_3$) δ 172.1, 137.2, 136.8, 129.3, 129.1, 128.9, 128.6, 128.4, 127.7, 96.9, 75.0, 71.8, 51.1, 39.6. HRMS (ESI) for $C_{18}H_{19}N_2O_4$: calculated $[M + H]^+$: 327.1343. Found: 327.1338.

3.4.2 Simultaneous methylation-hydrolysis of cycloadduct *exo*-L-3b

See section 3.2. (General procedure for the methylation reaction).

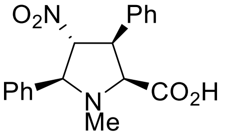

(2S,3S,4R,5S)-1-Methyl-4-nitro-3,5-diphenylpyrrolidine-2-carboxylic acid (*exo*-L-**8a**). The expected product was obtained from *exo*-L-**3b** (409 mg, 1.11 mmol). Yield: 327 mg, 90%, orange syrup. Analytical and spectroscopic data were in good agreement with the previously reported material.

3.4.3 Acidic conditions

To a solution of *endo*-L-**3b** (326 mg, 1.0 mmol) in dichloromethane (15 mL) stirred at room temperature, trifluoroacetic acid (8 mL) was added. The reaction mixture was stirred for 16 hours. Then, the solvent was evaporated *in vacuo*. The crude obtained was purified by precipitation to afford the desired product.

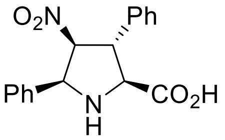

(2S,3R,4S,5S)-4-Nitro-3,5-diphenylpyrrolidine-2-carboxylic acid (*endo*-L-**4a**). The title product was obtained from *endo*-L-**3b** (368 mg, 1.0 mmol). Yield: 240 mg, 77%, white solid. Analytical and spectroscopic properties were coincident with the previously reported material.[3a] ^{1}H NMR (500 MHz, acetone-d_6) δ 7.51 (d, *J* = 7.7 Hz, 4H, ArH), 7.41 (t, *J* = 7.6 Hz, 2H, ArH), 7.38 – 7.29 (m, 4H, ArH), 5.57 (dd, *J* = 6.9, 4.4 Hz, 1H, C^4H), 5.21 (d, *J* = 6.9 Hz, 1H, C^5H), 4.28 (dd, *J* = 7.7, 4.3 Hz, 1H, C^3H), 4.20 (d, *J* = 7.8 Hz, 1H, C^2H).

3.5 General procedure for the coupling reaction between 4-amino-pyrrolidines and 2-carboxy-pyrrolidines

To a stirred solution of the corresponding amine *endo/exo*-L/D-**5a-b,7a** (0.8 mmol) in CH_2Cl_2 (10 mL) was added acid *endo/exo*-L/D-**4a** (1.0 mmol), PyBOP (1.0 mmol)

followed by diisopropyl ethyl amine (1.44 mmol). The reaction was then stirred until completion of the reaction. Then, the reaction mixture was diluted with CH_2Cl_2, washed with a 1M HCl solution, saturated aqueous $NaHCO_3$, brine and then dried over Na_2SO_4. Evaporation of the solvent followed by column chromatography eluting with ethyl acetate/hexane provided the products as described below.

Nomenclature: For a better assignment of protons on NMR spectra, the IUPAC carbon order in pyrrolidine-type rings has been used, being the cyclic system bearing the ester group the prioritary one. The following dimeric structures follow the same pattern.

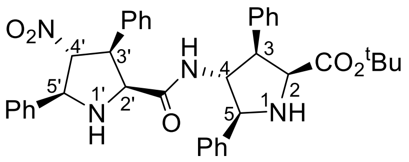

tert-Butyl (2S,3R,4R,5S)-3,5-dimethyl-4-((2'S,3'S,4'R,5'S)-4-nitro-3,5-diphenylpyrrolidine-2-carboxamido)pyrrolidine-2-carboxylate (X_LX_L-**9a**).

Yield: 374 mg, 74%, yellow solid. m_p = 135-137 ºC. $[\alpha]_D^{25}$ = +106.4 (*c* 0.65, acetone). FTIR (neat, cm^{-1}) 1721, 1675, 1553, 1367, 1153, 744, 697. ^1H NMR (500 MHz, $CDCl_3$) δ 7.51 – 7.33 (m, 12H, ArH), 7.28 – 7.18 (m, 3H, ArH), 7.12 (t, *J* = 7.4 Hz, 1H, ArH), 7.01 (m, 2H, ArH and amide NH), 6.80 (d, *J* = 7.5 Hz, 2H, ArH), 4.95 (t, *J* = 8.6 Hz, 1H, $C^{4'}H$), 4.77 (d, *J* = 8.4 Hz, 1H, $C^{5'}H$), 4.50 (q, *J* = 8.7 Hz, 1H, C^4H), 4.26 (t, *J* = 9.2 Hz, 1H, $C^{3'}H$), 4.21 (d, *J* = 9.3 Hz, 1H, $C^{2'}H$), 4.15 – 4.11 (m, 1H, C^2H), 4.11 – 4.03 (m, 1H, C^5H), 3.43 (t, *J* = 8.8 Hz, 1H, C^3H), 2.47 (bs, 2H, NH), 1.00 (s, 9H, $(CH_3)_3$). ^{13}C NMR (126 MHz, $CDCl_3$) δ 171.7, 169.5, 139.9, 138.2, 134.9, 129.6, 129.5, 129.4, 129.3, 129.0, 128.9, 128.8, 128.7, 128.5, 128.4, 127.9, 127.5, 127.0, 94.7, 81.8, 67.2, 66.4, 64.5, 64.0, 60.9, 54.2, 53.7, 27.8. HRMS (ESI) for $C_{38}H_{41}N_4O_5$: calculated $[M + H]^+$: 633.3077. Found: 633.3082.

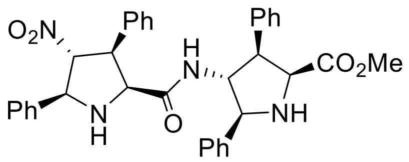

Methyl (2S,3R,4R,5S)-4-((2'S,3'S,4'R,5'S)-4-nitro-3,5-diphenylpyrrolidine-2-carboxamido)-3,5-diphenylpyrrolidine-2-carboxylate (X_LX_L-**9b**).

Yield: 307 mg, 65%, white solid. m_p = 136-137 ºC. $[\alpha]_D^{25}$ = +129.9 (*c* 0.43, acetone). FTIR (neat, cm^{-1}) 1732, 1671, 1546, 790, 696. ^1H NMR (500 MHz, $CDCl_3$) δ 7.56 – 7.33 (m, 10H, ArH), 7.30 – 7.17 (m, 5H, ArH), 7.11 (t, *J* = 7.4 Hz, 1H, ArH), 7.00 (t, *J* = 7.7 Hz, 2H, ArH), 6.93 (d, *J* = 8.8 Hz, 1H, CONH), 6.77 (d, *J* = 7.3 Hz, 2H, ArH), 4.93 (t, *J* = 8.6 Hz, 1H, $C^{4'}H$), 4.79 (d, *J* = 8.4 Hz, 1H, $C^{5'}H$), 4.59 (q, *J* = 9.0 Hz, 1H, C^4H), 4.33 – 4.17 (m, 3H, $C^{2'}H$, $C^{3'}H$, C^2H), 4.15 – 4.07 (m, 1H, C^5H), 3.52 (t, *J* = 9.1 Hz, 1H, C^3H), 3.20 (s, 3H, CO_2Me). ^{13}C NMR (101 MHz, $CDCl_3$) δ 173.1, 169.3, 137.2, 134.5, 129.4,

129.3, 129.2, 128.7, 128.6, 128.5, 128.4, 128.2 (2 signals), 127.7, 127.5, 126.7, 94.4, 67.08, 66.1, 64.2, 63.7, 60.2, 54.3, 53.5, 51.7. HRMS (ESI) for $C_{35}H_{35}N_4O_5$: calculated $[M + H]^+$: 591.2607. Found: 591.2617.

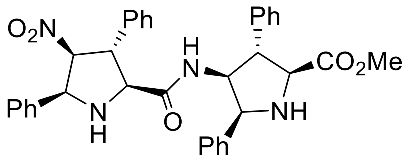

Methyl (2S,3S,4S,5S)-4-((2'S,3'R,4'S,5'S)-4-nitro-3,5-diphenylpyrrolidine-2-carboxamido)-3,5-diphenylpyrrolidine-2-carboxylate (N_LN_L-**9c**).

Yield: 354 mg, 75%, light brown solid. m_p = 103-104 ºC. $[\alpha]_D^{25}$ = -9.4 (*c* 0.90, acetone). FTIR (neat, cm^{-1}) 3358, 1733, 1661, 1551, 739, 698. ^1H NMR (500 MHz, $CDCl_3$) δ 7.54 (s, 2H, ArH), 7.46 – 7.26 (m, 14H, ArH), 7.19 – 7.11 (m, 2H, ArH), 6.98 (d, *J* = 9.7 Hz, 1H, ArH), 6.89 (d, *J* = 6.4 Hz, 2H, ArH and CONH), 5.03 (dt, *J* = 9.8, 5.0 Hz, 2H, $C^{4'}H$ and C^4H), 4.86 (d, *J* = 7.4 Hz, 1H, C^5H or $C^{5'}H$), 4.79 (d, *J* = 6.7 Hz, 1H, C^5H or $C^{5'}H$), 4.28 (d, *J* = 8.2 Hz, 1H, C^2H), 3.75 (s, 3H, CO_2Me), 3.64 (t, *J* = 8.2 Hz, 1H, $C^{3'}H$), 3.62 – 3.58 (m, 2H, C^3H and $C^{2'}H$). ^{13}C NMR (126 MHz, $CDCl_3$) δ 174.2, 170.9, 140.5, 138.7, 138.4, 135.0, 129.0, 128.8, 128.7, 128.4, 128.4, 128.1, 127.7, 127.6, 127.5, 127.4, 127.3, 126.8, 95.8, 67.0, 66.0, 64.2, 62.6, 58.4, 54.0, 53.9, 52.3. HRMS (ESI) for $C_{35}H_{35}N_4O_5$: calculated $[M + H]^+$: 591.2607. Found: 591.2618.

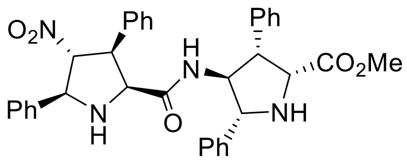

Methyl (2R,3S,4S,5R)-4-((2'S,3'S,4'R,5'S)-4-nitro-3,5-diphenylpyrrolidine-2-carboxamido)-3,5-diphenylpyrrolidine-2-carboxylate (X_LX_D-**9d**).

Yield: 217 mg, 46%, white solid. m_p = 114-115 ºC. $[\alpha]_D^{25}$ = +17.1 (*c* 0.43, acetone). FTIR (neat, cm^{-1}) 1733, 1667, 1603, 697. ^1H NMR (500 MHz, $CDCl_3$) δ 7.47 (m, 6H, ArH), 7.38 – 7.21 (m, 8H, ArH), 7.09 (dt, *J* = 14.9, 7.2 Hz, 4H, ArH), 6.97 (d, *J* = 7.3 Hz, 2H, ArH), 6.73 (d, *J* = 8.4 Hz, 1H, CONH), 5.02 – 4.94 (m, 1H, $C^{4'}H$), 4.78 (d, *J* = 8.0 Hz, 1H, $C^{5'}H$), 4.51 (dd, *J* = 16.7, 8.1 Hz, 1H, C^4H), 4.23 (dd, *J* = 13.9, 7.2 Hz, 3H, C^3H, $C^{2'}H$ and $C^{3'}H$), 4.01 (d, *J* = 8.6 Hz, 1H, C^5H), 3.31 – 3.18 (m, 4H, C^2H and CO_2Me), 2.56 (bs, 2H, NH). ^{13}C NMR (126 MHz, $CDCl_3$) δ 172.5, 169.2, 139.4, 138.4, 138.1, 135.5, 129.2, 129.0, 128.8, 128.6, 128.3, 128.3, 128.2, 128.2, 128.1, 127.3, 127.3, 126.7, 95.2, 67.3, 66.2, 64.4, 64.2, 61.2, 54.1, 53.0, 51.6. HRMS (ESI) for $C_{35}H_{35}N_4O_5$: calculated $[M + H]^+$: 591.2607. Found: 591.2609.

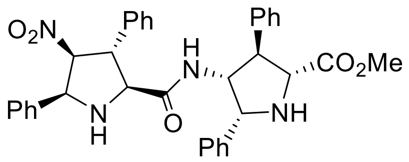

Methyl (2R,3R,4R,5R)-4-((2'S,3'R,4'S,5'S)-4-nitro-3,5-diphenylpyrrolidine-2-carboxamido)-3,5-diphenylpyrrolidine-2-carboxylate (N_LN_D-**9e**).

Yield: 255 mg, 54%, light orange solid. m_p = 118-119 ºC. $[\alpha]_D^{25}$ = +45.2 (*c* 0.50, acetone). FTIR (neat, cm^{-1}) 1735, 1673, 1550, 1496, 697. ^1H NMR (500 MHz, $CDCl_3$) δ 7.55 (d, *J* = 7.3 Hz, 2H, ArH), 7.42 (t, *J* = 7.5 Hz, 2H, ArH), 7.38 – 7.23 (m, 12H, ArH), 7.23 – 7.20 (m, 2H, ArH), 7.08 (d, *J* = 7.2 Hz, 2H, ArH), 6.89 (d, *J* = 7.8 Hz, 1H, CONH), 5.12 (dd, *J* = 7.3, 4.6 Hz, 1H, $C^{4'}H$), 4.88 (d, *J* = 6.1 Hz, 1H, C^5H), 4.86 – 4.80 (m, 1H, C^4H), 4.78 (d, *J* = 6.8 Hz, 1H, $C^{5'}H$), 4.20 (d, *J* = 6.7 Hz, 1H, C^2H), 3.78 – 3.74 (m, 1H, $C^{2'}H$), 3.73 (s, 3H, CO_2Me), 3.65 (dd, *J* = 7.9, 4.5 Hz, 1H, $C^{3'}H$), 3.57 (t, *J* = 6.6 Hz, 1H, C^3H). ^{13}C NMR (101 MHz, $CDCl_3$) δ 174.5, 170.4, 139.3, 139.1, 138.5, 135.2, 129.2, 128.9, 128.8, 128.6, 127.9, 127.8, 127.7, 127.5, 127.1, 96.4, 67.1, 66.1, 64.4, 63.2, 59.0, 54.3, 53.6, 52.4. HRMS (ESI) for $C_{35}H_{35}N_4O_5$: calculated $[M + H]^+$: 591.2607. Found: 591.2622.

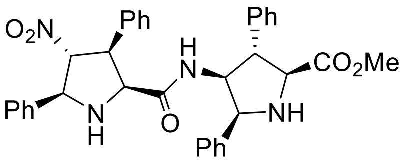

Methyl (2S,3S,4S,5S)-4-((2'S,3'S,4'R,5'S)-4-nitro-3,5-diphenylpyrrolidine-2-carboxamido)-3,5-diphenylpyrrolidine-2-carboxylate (X_LN_L-**9f**).

Yield: 335 mg, 71%, white solid. m_p = 93-95 ºC. $[\alpha]_D^{25}$ = +74.2 (*c* 0.50, acetone). FTIR (neat, cm^{-1})1733, 1670, 1549, 1494, 697. ^1H NMR (500 MHz, $CDCl_3$) δ 7.47 (d, *J* = 4.3 Hz, 3H, ArH), 7.41 (dd, *J* = 13.4, 5.9 Hz, 3H, ArH), 7.35 (t, *J* = 7.3 Hz, 2H, ArH), 7.31 – 7.23 (m, 5H, ArH), 7.18 (dt, *J* = 16.2, 8.1 Hz, 3H, ArH and CONH), 7.12 (dd, *J* = 7.3, 5.6 Hz, 3H, ArH), 6.95 (d, *J* = 7.2 Hz, 2H, ArH), 4.87 (t, *J* = 7.6 Hz, 1H, $C^{4'}H$), 4.73 (t, *J* = 6.7 Hz, 1H, $C^{5'}H$), 4.71 (t, *J* = 8.4 Hz, 1H, C^5H), 4.45 – 4.37 (m, 1H, C^4H), 4.21 – 4.13 (m, 2H, C^2H and $C^{3'}H$), 4.07 (d, *J* = 9.7 Hz, 1H, $C^{2'}H$), 3.84 (s, 3H, CO_2Me), 3.04 (t, *J* = 5.7 Hz, 1H, C^3H).^{13}C NMR (101 MHz, $CDCl_3$) δ 168.7, 139.7, 139.7, 138.1, 135.8, 129.2, 129.1, 129.0, 128.6, 128.5, 128.4, 128.1, 127.7, 127.6, 127.5 (2 signals), 126.7, 95.4, 65.8, 64.2, 64.1, 63.5, 59.2, 54.4, 52.7, 52.5. HRMS (ESI) for $C_{35}H_{35}N_4O_5$: calculated $[M + H]^+$: 591.2607. Found: 591.2615.

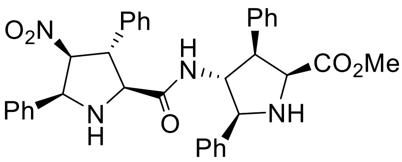

Methyl (2S,3R,4R,5S)-4-((2'S,3'R,4'S,5'S)-4-nitro-3,5-diphenylpyrrolidine-2-carboxamido)-3,5-diphenylpyrrolidine-2-carboxylate (N_LX_L-**9g**).

Yield: 359 mg, 76%, yellow solid. m_p = 180-181 ºC. $[\alpha]_D^{25}$ = +29.6 (*c* 0.50, acetone). FTIR (neat, cm^{-1}) 1734, 1655, 1554, 1528, 837, 697. ^1H NMR (500 MHz, $CDCl_3$) δ 7.74 (d, *J* = 7.2 Hz, 2H, ArH), 7.42 (t, *J* = 7.3 Hz, 2H, ArH), 7.33 (m, 10H, ArH), 7.25 – 7.20 (m, 4H, ArH), 6.89 (d, *J* = 9.8 Hz, 1H, CONH), 6.78 (dd, *J* = 6.4, 2.7 Hz, 2H, ArH), 5.11 (m, 2H, $C^{4'}H$ and C^4H), 4.80 (d, *J* = 6.2 Hz, 1H, $C^{5'}H$), 4.41 (d, *J* = 9.5 Hz, 1H, C^5H),

4.33 (d, J = 9.4 Hz, 1H, C^2H), 3.94 (t, J = 10.1 Hz, 1H, C^3H), 3.66 (d, J = 7.5 Hz, 1H, $C^{2'}H$), 3.59 (m, 1H, $C^{3'}H$), 3.20 (s, 3H, CO_2Me). ^{13}C NMR (126 MHz, $CDCl_3$) δ 173.6, 171.0, 139.7, 138.3, 136.6, 135.2, 129.2, 129.2, 129.0, 128.8, 128.6, 128.6, 128.5, 128.1, 128.0, 127.7, 127.6, 127.0, 96.2, 67.8, 67.3, 66.7, 63.9, 58.6, 54.8, 53.8, 51.8. HRMS (ESI) for $C_{35}H_{35}N_4O_5$: calculated $[M+H]^+$: 591.2607. Found: 591.2614.

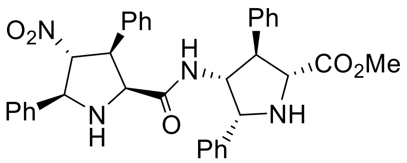

Methyl (2R,3R,4R,5R)-4-((2'S,3'S,4'R,5'S)-4-nitro-3,5-diphenylpyrrolidine-2-carboxamido)-3,5-diphenylpyrrolidine-2-carboxylate (X_LN_D-**9h**).

Yield: 312 mg, 66%, white solid. m_p = 101-102 ºC. $[\alpha]_D^{25}$ = +74.3 (*c* 0.44, acetone). FTIR (neat, cm^{-1}) 1734, 1667, 1548, 1494, 697. ^1H NMR (500 MHz, $CDCl_3$) δ 7.53 – 7.22 (m, 16H, ArH and CONH), 7.19 – 7.11 (m, 1H, ArH), 7.07 (t, J = 7.0 Hz, 2H, ArH), 6.67 (d, J = 7.2 Hz, 2H, ArH), 4.75 – 4.71 (m, 2H, $C^{4'}H$ and $C^{2'}$ or $C^{3'}$ or $C^{5'}H$), 4.65 (t, J = 7.7 Hz, 1H, C^5H), 4.22 (m, 3H, C^2H and $C^{2'}$ or $C^{3'}$ or $C^{5'}H$), 4.08 (m, 1H, C^4H), 3.71 (s, 3H CO_2Me), 3.41 (m, 1H, C^3H) ($C^{2'}$, $C^{3'}$ and $C^{5'}H$ cannot be assigned due to signal overlap in ^1H NMR and COSY experiments). ^{13}C NMR (126 MHz, $CDCl_3$) δ 175.3, 169.4, 140.1, 138.6, 137.7, 135.0, 129.0, 129.0, 128.8, 128.7, 128.5, 128.2, 128.1, 127.8, 127.5, 127.4, 127.2, 126.7, 94.6, 66.0, 63.7, 63.0, 62.8, 59.7, 55.7, 52.9, 52.3. HRMS (ESI) for $C_{35}H_{35}N_4O_5$: calculated $[M + H]^+$: 591.2607. Found: 591.2601.

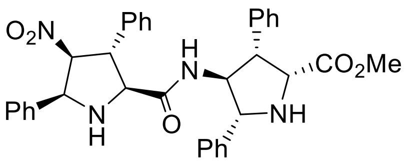

Methyl (2R,3S,4S,5R)-4-((2'S,3'R,4'S,5'S)-4-nitro-3,5-diphenylpyrrolidine-2-carboxamido)-3,5-diphenylpyrrolidine-2-carboxylate (N_LX_D-**9i**).

Yield: 340 mg, 72%, white solid. m_p = 201-202 ºC. $[\alpha]_D^{25}$ = -16.1 (*c* 0.24, acetone). FTIR (neat, cm^{-1}) 1737, 1654, 1551, 1525, 698. ^1H NMR (500 MHz, $CDCl_3$) δ 7.66 (d, J = 7.4 Hz, 2H, ArH), 7.46 – 7.27 (m, 16H, ArH), 6.98 – 6.84 (m, 3H, ArH and CONH), 5.11 (m, 1H, $C^{4'}H$), 4.94 (dd, J = 19.5, 9.7 Hz, 1H, C^4H), 4.87 (d, J = 6.9 Hz, 1H, $C^{5'}H$), 4.39 (d, J = 9.8 Hz, 1H, C^2H), 4.33 (d, J = 9.8 Hz, 1H, C^5H), 4.00 (t, J = 10.0 Hz, 1H, C^3H), 3.76 (d, J = 6.7 Hz, 1H, $C^{2'}H$), 3.56 (m, 1H, $C^{3'}H$), 3.27 (s, 3H, CO_2Me), 2.66 (broad s, 2H, NH). ^{13}C NMR (126 MHz, $CDCl_3$) δ 173.7, 171.2, 139.6, 138.5, 137.1, 135.1, 129.3, 129.21, 129.0, 128.9, 128.7, 128.6, 128.4, 128.1, 127.8, 127.7, 126.9, 96.2, 67.7, 66.7, 66.4, 63.8, 59.5, 54.7, 54.5, 51.8. HRMS (ESI) for $C_{35}H_{35}N_4O_5$: calculated $[M + H]^+$: 591.2607. Found: 591.2620.

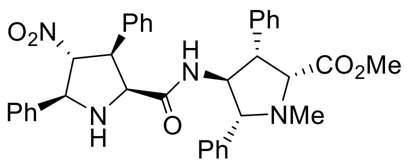

Methyl (2R,3S,4S,5R)-1-methyl-4-((2'S,3'S,4'R,5'S)-4-nitro-3,5-diphenylpyrrolidine-2-carboxamido)-3,5-diphenylpyrrolidine-2-carboxylate ($X_LX_D^{Me}$-**9j**).

Yield: 353 mg, 73%, white solid. m_p = 99-100 ºC. $[\alpha]_D^{25}$ = +19.0 (*c* 0.51, acetone). FTIR (neat, cm^{-1}) 1748, 1669, 1547, 698. ^1H NMR (400 MHz, $CDCl_3$) δ 7.59 – 7.11 (m, 20H, ArH), 6.70 (d, *J* = 7.2 Hz, 1H, CONH), 5.17 – 4.99 (m, 1H, $C^{4'}H$), 4.82 (d, *J* = 7.4 Hz, 1H, $C^{5'}H$), 4.28 (m, 2H, $C^{2'}H$ and $C^{3'}H$), 4.04 (dd, *J* = 13.7, 7.2 Hz, 1H, C^4H), 3.54 (d, *J* = 9.2 Hz, 1H, C^2H), 3.23 (s, 3H, CO_2Me), 3.16 (d, *J* = 8.1 Hz, 1H, C^5H), 2.90 (dd, *J* = 8.7, 6.1 Hz, 1H, C^3H), 2.62 (bs, 1H, NH), 2.23 (s, 3H, NMe). ^{13}C NMR (101 MHz, $CDCl_3$) δ 170.8, 169.0, 140.3, 139.2, 138.0, 136.2, 129.4, 129.2, 128.9, 128.9, 128.7, 128.7, 128.4, 128.4, 128.1, 127.9, 127.1, 126.8, 95.7, 76.4, 72.3, 66.5, 64.9, 64.4, 53.0, 52.2, 51.3, 39.8. HRMS (ESI) for $C_{36}H_{37}N_4O_5$: calculated $[M + H]^+$: 605.2764. Found: 605.2773.

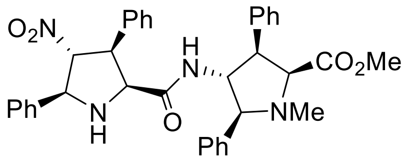

Methyl (2S,3R,4R,5S)-1-methyl-4-((2'S,3'S,4'R,5'S)-4-nitro-3,5-diphenylpyrrolidine-2-carboxamido)-3,5-diphenylpyrrolidine-2-carboxylate ($X_LX_L^{Me}$-**9k**).

Yield: 358 mg, 74%, white solid. m_p = 201-204 ºC. $[\alpha]_D^{25}$ = +125.3 (*c* 0.50, acetone). FTIR (neat, cm^{-1}) 3311, 1745, 1672, 1551, 1194, 1171, 698. ^1H NMR (400 MHz, $CDCl_3$) δ 7.42 (m, 10H, ArH), 7.33 – 7.16 (m, 6H, ArH), 7.10 (t, *J* = 7.4 Hz, 2H, ArH), 6.90 (d, *J* = 7.4 Hz, 2H, ArH), 6.83 (d, *J* = 7.8 Hz, 1H, CONH), 4.98 (d, *J* = 4.9 Hz, 1H, $C^{4'}H$), 4.81 (d, *J* = 7.8 Hz, 1H, $C^{5'}H$), 4.29 (s, 2H, $C^{2'}H$ and $C^{3'}H$), 4.11 (q, *J* = 7.8 Hz, 1H, C^4H), 3.68 (d, *J* = 9.5 Hz, 1H, C^2H), 3.43 (d, *J* = 8.6 Hz, 1H, C^5H), 3.31 (t, *J* = 8.2 Hz, 1H, C^3H), 3.19 (s, 3H, CO_2Me), 2.58 (m, 1H, NH), 2.22 (s, 3H, NMe). ^{13}C NMR (101 MHz, $CDCl_3$) δ 171.25, 169.5, 139.7, 139.6, 137.9, 135.0, 129.3, 129.3, 129.0, 128.9, 128.9, 128.5, 128.3 (x2), 128.2, 128.0, 127.2, 126.8, 94.8, 74.8, 72.1, 66.3, 64.3, 64.2, 53.4, 51.8, 51.3, 39.9. HRMS (ESI) for $C_{36}H_{37}N_4O_5$: calculated $[M + H]^+$: 605.2764. Found: 605.2778.

Additional procedure for the synthesis of catalysts $X_L^{Me}X_{D/L}$-9l-m

To a stirred solution of the corresponding amine *exo*-D/L-**5a** (80 mg, 0.27 mmol) in 8 mL of dichloromethane, *exo*-L-**8a** (104 mg, 0.31 mmol), HATU (104 mg, 0.31 mmol) and diisopropyl ethyl amine (48 μL, 0.31 mmol) were added. The resulting mixture was then stirred until completion of the reaction. Then, the reaction mixture was diluted with

CH_2Cl_2, washed with a 1M HCl solution, saturated aqueous $NaHCO_3$, brine and then dried over Na_2SO_4. Filtration and evaporation of the solvent followed by column chromatography eluting with EtOAc/hexane provided the products described below.

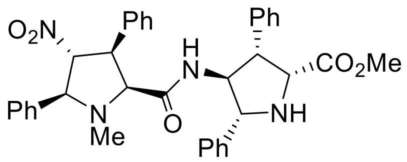

Methyl (2R,3S,4S,5R)-4-((2'S,3'S,4'R,5'S)-1-methyl-4-nitro-3,5-diphenylpyrrolidine-2-carboxamido)-3,5-diphenylpyrrolidine-2-carboxylate ($X_L^{Me}X_D$-**9l**).

Yield: 49 mg, 30%, light green solid. m_p = 96-100 ºC. $[\alpha]_D^{25}$ = +91.1 (*c* 0.65, acetone). FTIR (neat, cm^{-1}) 1735, 1677, 1548, 1495, 1369, 1207, 697. ^{1}H NMR (400 MHz, $CDCl_3$) δ 7.45 (dt, *J* = 23.1, 7.6 Hz, 7H, ArH), 7.37 – 7.20 (m, 6H, ArH), 7.15 (m, 3H, ArH), 7.11 – 6.97 (m, 6H, ArH), 6.86 (d, *J* = 8.5 Hz, 1H, CONH), 4.99 (t, *J* = 7.9 Hz, 1H, $C^{4'}H$), 4.53 (q, *J* = 8.5 Hz, 1H, C^4H), 4.28 – 4.14 (m, 2H, $C^{3'}H$ and C^2H), 4.03 (dd, *J* = 8.5, 3.4 Hz, 2H, $C^{5'}H$ and C^5H), 3.63 (d, *J* = 10.2 Hz, 1H, $C^{2'}H$), 3.22 (m, 4H, CO_2Me and C^3H), 2.49 (bs, 1H, NH), 2.08 (s, 3H, NMe). ^{13}C NMR (101 MHz, $CDCl_3$) δ 172.7, 168.1, 139.2, 138.1, 136.5, 135.6, 129.4, 129.4, 128.9, 128.6, 128.4, 128.3, 127.5, 127.2, 95.9, 74.1, 72.9, 68.0, 64.5, 60.8, 54.0, 51.7, 51.1, 39.9. HRMS (ESI) for $C_{36}H_{37}N_4O_5$: calculated $[M + H]^+$: 605.2764. Found: 605.2763.

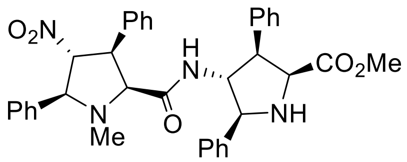

Methyl (2S,3R,4R,5S)-4-((2'S,3'S,4'R,5'S)-1-methyl-4-nitro-3,5-diphenylpyrrolidine-2-carboxamido)-3,5-diphenylpyrrolidine-2-carboxylate ($X_L^{Me}X_L$-**9m**).

Yield: 52 mg, 32%, light yellow solid. m_p = 126-130 ºC. $[\alpha]_D^{25}$ = +13.9 (*c* 1.00, acetone). FTIR (neat, cm^{-1}) 1733, 1676, 1549, 1496, 1454, 1210, 727, 696. ^{1}H NMR (400 MHz, $CDCl_3$) δ 7.53 (d, *J* = 7.0 Hz, 2H, ArH), 7.48 – 7.31 (m, 9H, ArH), 7.22 (t, *J* = 7.8 Hz, 4H, ArH), 7.06 (d, *J* = 7.6 Hz, 1H, ArH), 7.01 (d, *J* = 8.7 Hz, 1H, CONH), 6.93 (t, *J* = 7.6 Hz, 2H, ArH), 6.84 (d, *J* = 7.7 Hz, 2H, ArH), 4.89 (t, *J* = 8.6 Hz, 1H, $C^{4'}H$), 4.58 (q, *J* = 9.0 Hz, 1H, C^4H), 4.30 (d, *J* = 9.2 Hz, 1H, C^2H), 4.27 – 4.15 (m, 2H, C^5H and $C^{3'}H$), 4.03 (d, *J* = 8.4 Hz, 1H, $C^{5'}H$), 3.63 (dd, *J* = 12.4, 9.9 Hz, 2H, C^3H and $C^{2'}H$), 3.22 (s, 3H, CO_2Me), 1.99 (s, 3H, NMe). ^{13}C NMR (101 MHz, $CDCl_3$) δ 173.3, 168.4, 139.8, 137.2, 136.4, 135.0, 129.5 (2 signals), 129.1, 128.7, 128.5, 128.5, 128.4, 128.3, 128.2, 127.9, 127.6, 127.3, 95.7, 73.9, 72.5, 66.3, 64.1, 60.3, 54.5, 51.7, 51.3, 39.8. HRMS (ESI) for $C_{36}H_{37}N_4O_5$: calculated $[M + H]^+$: 605.2764. Found: 605.2764.

4 Procedures for asymmetric C-C bond forming transformations

4.1 Organocatalytic asymmetric aldol reactions

Aldehyde **11** (49 mg, 0.25 mmol) was dissolved in cyclohexanone **10a** (15 mmol) and the corresponding dimeric catalyst **9** (0.03 mmol) was added followed by TFA (2.87 µL, 0.0375 mmol).The resulting mixture was stirred at room temperature until completion of the reaction monitorized by TLC. Then, the reaction mixture was diluted with ethyl acetate, washed with 0.1 M of phosphate buffer solution (pH 7), dried onto sodium sulfate, filtered and concentrated under reduced pressure. The waxy solid was purified by flash chromatography eluting with ethyl acetate/hexane giving the corresponding aldol product.

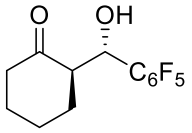

(R)-2-((S)-Hydroxy(perfluorophenyl)methyl)cyclohexan-1-one (**12**).

The reaction was carried out under the conditions described in Table 3, entry 11. Yield: 68.4 mg, 93%, white solid. Analytical and spectroscopic data were in good agreement with those reported in the literature.[5] ^{1}H NMR (500 MHz, $CDCl_3$) δ 5.39 – 5.30 (m, 1H, CHOH), 3.92 (d, *J* = 3.3 Hz, 1H, CH), 3.09 – 2.99 (m, 1H, CH_2), 2.60 – 2.48 (m, 1H, CH_2), 2.43 (td, *J* = 12.9, 6.3 Hz, 1H, CH_2), 2.22 – 2.11 (m, 1H, CH_2), 1.95 – 1.85 (m, 1H, CH_2), 1.76 – 1.61 (m, 3H, CH_2), 1.41 – 1.26 (m, 1H, CH_2). ^{19}F NMR (376 MHz, cyclohexanone) δ -143.00 (dd, *J* = 22.3, 7.1 Hz, $2F_o$), -157.75 (t, *J* = 21.0 Hz, $1F_p$), -164.31 (td, *J* = 22.1, 7.4 Hz, $2F_m$). HPLC (Daicel Chiralpak IA, hexane/^{i}PrOH = 90/10, flow rate 1.0 mL/min, λ = 254 nm), t_R (minor) = 8.25 min, t_R (major) = 10.05 min; ee = 90%.

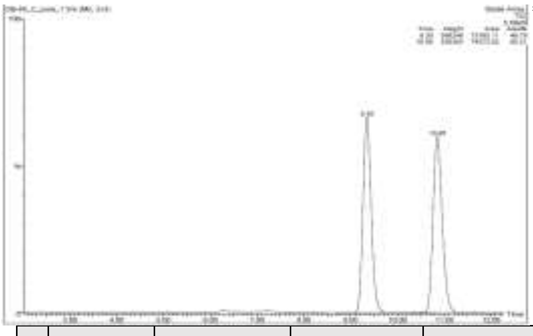

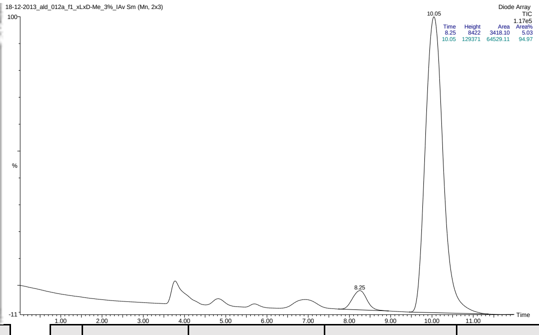

	Time	Height	Area	% Area
**1**	9.35	366246	73760.11	49.79
**2**	10.85	330393	74372.02	50.21

	Time	Height	Area	% Area
**1**	8.25	8422	3418.10	5.03
**2**	10.05	129371	64529.11	94.97

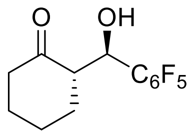

(S)-2-((R)-Hydroxy(perfluorophenyl)methyl)cyclohexan-1-one (**12**). The reaction was carried out under the conditions described in Table 3, entry 14. Yield: 62.0 mg, 84%, white solid. Analytical and spectroscopic data were in good agreement with those reported in the literature.[5] ^{1}H NMR (500 MHz, $CDCl_3$) δ 5.39 – 5.30 (m, 1H, CHOH), 3.92 (d, J = 3.3 Hz, 1H, CH), 3.09 – 2.99 (m, 1H, CH_2), 2.60 – 2.48 (m, 1H, CH_2), 2.43 (td, J = 12.9, 6.3 Hz, 1H, CH_2), 2.22 – 2.11 (m, 1H, CH_2), 1.95 – 1.85 (m, 1H, CH_2), 1.76 – 1.61 (m, 3H, CH_2), 1.41 – 1.26 (m, 1H, CH_2). ^{19}F NMR (376 MHz, cyclohexanone) δ -143.00 (dd, J = 22.3, 7.1 Hz, $2F_o$), -157.75 (t, J = 21.0 Hz, $1F_p$), -164.31 (td, J = 22.1, 7.4 Hz, $2F_m$). HPLC (Daicel Chiralpak IA, hexane/^{i}PrOH = 90/10, flow rate 1.0 mL/min, λ = 254 nm), t_R (minor) = 8.77 min, t_R (major) = 10.84 min; .ee = -88%.

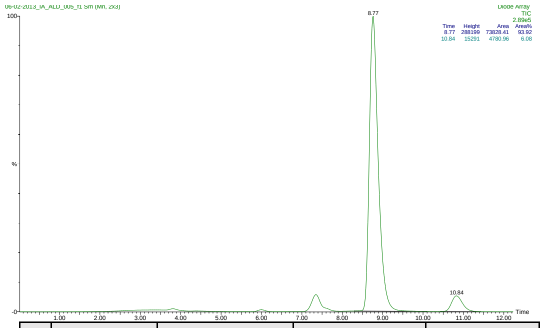

	Time	Height	Area	% Area
1	8.77	288199	73828.41	93.92
2	10.84	15291	4780.96	6.08

4.2 Organocatalytic asymmetric Michael reactions

A reaction mixture of dimeric catalyst **9** (0.02-0.04 mmol), the corresponding acid (0.02-0.04 mmol), cyclohexanone **10a** (0.8 mmol) and nitrostyrene **13a** or 1,1-bis(phenylsulphonyl)ethylene **16** (0.1 mmol) was allowed to stir at room temperature for 16 hours. The progress of the reaction was monitored by TLC (1:3 of EtOAc:Hex). After consumption of the nitrostyrene, cyclohexanone was evaporated under reduced pressure. The afforded crude product was purified by column chromatography over silica gel using EtOAc:Hex system as eluent.

For the racemic compounds, the reactions were carried out using pyrrolidine (8 μL, 0.10 mmol).

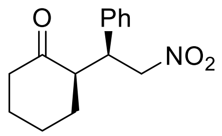

(R)-2-((S)-2-Nitro-1-phenylethyl)cyclohexanone (**14**).[6] The reaction was carried out under the conditions described in Table 5, entry 16. Yield: 22.5 mg, 91%, white solid. Analytical and spectroscopic data

were in good agreement with those reported in the literature. ^{1}H NMR (500 MHz, $CDCl_3$) δ 7.37 – 7.30 (m, 3H, ArH), 7.17 (d, *J* = 7.1 Hz, 2H, ArH), 4.94 (dd, *J* = 12.4, 4.4 Hz, 1H, $CHNO_2$), 4.69 – 4.57 (m, 1H, $CHNO_2$), 3.81 – 3.68 (m, 1H, CHAr), 2.76 – 2.62 (m, 1H, CH), 2.48 (d, *J* = 13.3 Hz, 1H, CH_2), 2.40 (dd, *J* = 12.4, 6.2 Hz, 1H, CH_2), 2.07 (m, 1H, CH_2), 1.85 – 1.58 (m, 4H, CH_2), 1.24 (m, 1H, CH_2). HPLC (Daicel Chiralpak AS-H, hexane/^{i}PrOH = 90/10, flow rate 1.0 mL/min, λ = 210 nm), t_R (major) = 22.21 min, t_R (minor) = 34.54 min; ee = 96%.

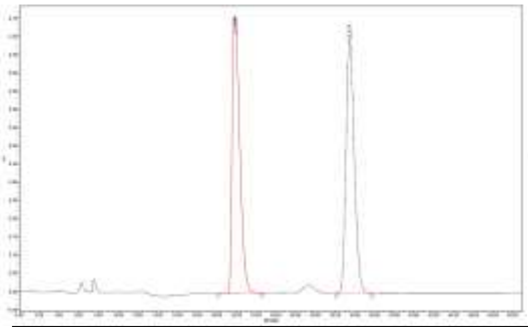

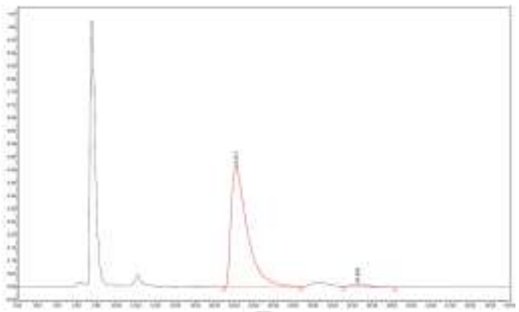

	Time	Area	% Height	% Area
1	21.839	40597597	52.39	50.03
2	33.471	40550593	47.61	49.97

	Time	Area	% Height	% Area
1	22.211	52028937	98.33	98.25
2	34.540	928288	1.67	1.75

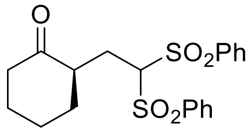

(R)-2-(2,2-bis(Phenylsulfonyl)ethyl)cyclohexan-1-one (**17**).[7]

Yield: 25.6 mg, 63%, white solid. Analytical and spectroscopic data were in good agreement with those reported in the literature. ^{1}H NMR (500 MHz, $CDCl_3$) δ 7.92 (dd, *J* = 24.1, 7.6 Hz, 4H, ArH), 7.68 (q, *J* = 8.2 Hz, 2H, ArH), 7.56 (q, *J* = 7.1 Hz, 4H, ArH), 4.98 (dd, *J* = 9.2, 3.8 Hz, 1H, $CH(SO_2Ph)_2$), 3.07 (dq, *J* = 13.6, 5.2 Hz, 1H, CH_2), 2.52 (m, 2H, CH_2), 2.08 (m, 2H, CH_2), 1.95 (m, 2H, CH_2), 1.63 – 1.49 (m, 2H, CH_2), 1.29 (m, 2H, CH_2). HPLC (Daicel Chiralpak AS-H, hexane/^{i}PrOH = 70/30, flow rate 1.0 mL/min, λ = 210 nm), t_R (major) = 32.62 min, t_R (minor) = 43.31 min; ee = 95%.

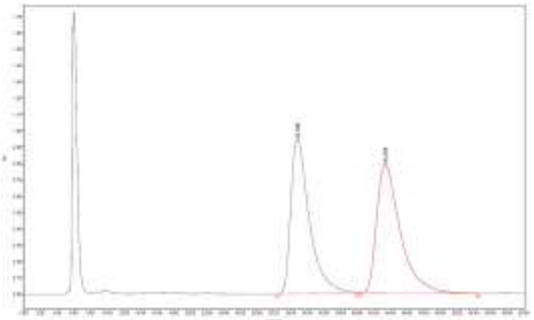

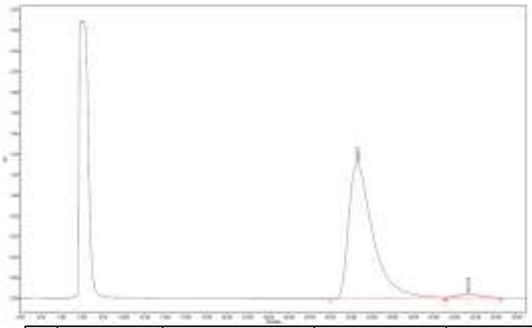

	Time	Area	% Height	% Area

	Time	Area	% Height	% Area

**1**	32.852	146278858	54.47	48.58
**2**	43.384	154835276	45.53	51.42

**1**	32.617	210804474	97.19	97.48
**2**	43.311	5451911	2.81	2.52

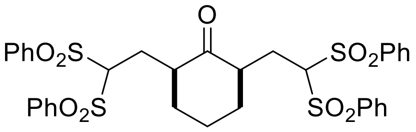

2,6-bis(2,2-bis(Phenylsulfonyl)ethyl)cyclohexan-1-one (meso-**18**).

The reaction was performed with NO_2-$X_LX_L^{Me}$-**9k** to obtain **17**:meso-**18** in 32:68 ratio. Yield: 11.0 mg, 62%, white solid. m_p = 134-135 ºC. FTIR (neat, cm^{-1}) 1704, 1327, 1311, 1147, 730, 686. ^1H NMR (400 MHz, $CDCl_3$) δ 7.90 (dd, *J* = 24.4, 7.9 Hz, 8H, ArH), 7.68 (q, *J* = 7.8 Hz, 4H, ArH), 7.53 (dt, *J* = 15.8, 7.7 Hz, 8H, ArH), 4.87 (dd, *J* = 9.4, 4.0 Hz, 2H, $CH(SO_2Ph)_2$), 3.36 – 3.00 (m, 2H, $CH_2CH(SO_2Ph)_2$), 2.48 (ddd, *J* = 14.4, 9.6, 4.2 Hz, 2H, $CH_2CH(SO_2Ph)_2$), 2.12 (d, *J* = 9.7 Hz, 2H, COCH), 1.99 (ddd, *J* = 14.5, 9.5, 4.6 Hz, 2H, CH_2), 1.83 (s, 2H, CH_2), 1.40 – 1.14 (m, 2H, CH_2). ^{13}C NMR (101 MHz, $CDCl_3$) δ 212.5, 138.4, 138.0, 134.7, 134.6, 129.8, 129.6, 129.3, 129.2, 80.7, 48.2, 36.1, 26.1, 25.1. HRMS (ESI) for $C_{34}H_{35}O_9S_4$: calculated $[M + H]^+$: 715.1164. Found: 715.1180.

Additional procedure for the synthesis of 14b-c

A reaction mixture of dimeric catalyst NO_2-$X_LX_L^{Me}$-**9k** (12.10 mg, 0.02 mmol), salicylic acid (2.76 mg, 0.02 mmol), ketone **10b-c** (0.10 mmol) and *trans*-β-nitrostyrene **13a** (14.90 mg, 0.11 mmol) was stirred at room temperature or-10 °C until total consumption of the nitroalkene. Afterwards, the crude mixture was evaporated under reduced pressure and purified by flash column chromatography on silica gel (1:2 EtOAc:Hexane).

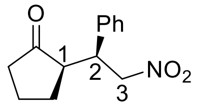

(R)-2-((S)-2-nitro-1-phenylethyl)cyclopentan-1-one (**14b**).

The title product was obtained from cyclopentanone **10b**. Yield: 95% (22 mg, 0.095 mmol), white solid. Analytical and spectroscopic data were in good agreement with those reported in the literature.[8] ^1H NMR (400 MHz, $CDCl_3$) δ 7.35 – 7.24 (m, 3H, ArH), 7.20 – 7.15 (m, 2H, ArH), 5.35 (d, *J* = 5.6 Hz, 1H, C^3H), 4.71 (dd, *J* = 12.8, 10.0 Hz, 1H, C^3H), 3.69 (td, *J* = 9.5, 5.5 Hz, 1H, C^2H), 2.45 – 2.27 (m, 2H, CH_2), 2.12 (ddd, *J* = 19.0, 10.6, 8.7 Hz, 1H, C^1H), 1.97 – 1.78 (m, 2H, CH_2), 1.74 – 1.66 (m, 1H, CH_2), 1.54 – 1.39 (m, 1H, CH_2). **HPLC** (Daicel Chiralcel OD-H, Hexane:^{i}PrOH = 90:10, flow rate 1 mL/min, λ = 210 nm), t_R (minor) = 17.98 min, t_R (major) = 22.12 min; *ee* = 90%.

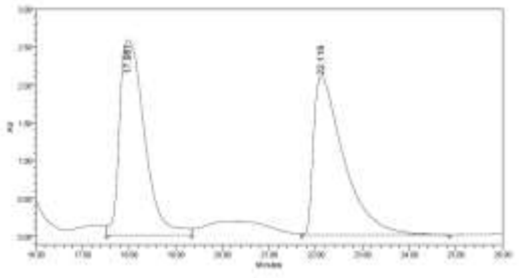

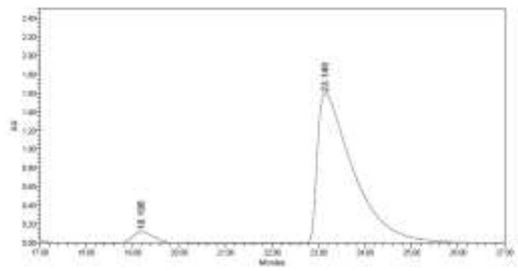

	RT	Height	Area	% Area
1	17.981	2585598	96504734	50.58
2	22.119	2092659	94307764	49.42

	RT	Height	Area	% Area
1	19.196	121177	3717563	4.13
2	23.149	1600476	86208468	95.87

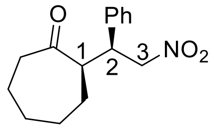

(R)-2-((S)-2-nitro-1-phenylethyl)cycloheptan-1-one (**14c**).

The title product was obtained from cycloheptanone **10c**. Yield 94% (25 mg, 0.094 mmol), white solid. Analytical and spectroscopic data were in good agreement with those reported in the literature.[9] ^{1}H NMR (400 MHz, $CDCl_3$) δ 7.32 (ddd, J = 14.8, 7.8, 6.2 Hz, 3H, ArH), 7.20 – 7.16 (m, 2H, ArH), 4.67 – 4.63 (m, 2H, C^3H), 3.68 (ddd, J = 10.2, 8.3, 5.1 Hz, 1H, C^2H), 3.00 (td, J = 10.3, 3.4 Hz, 1H, C^1H), 2.57 – 2.47 (m, 2H, CH_2), 1.99 – 1.82 (m, 2H, CH_2), 1.80 – 1.52 (m, 3H, CH_2), 1.29 – 1.12 (m, 3H, CH_2). **HPLC** (Daicel Chiralpak AD-H, Hexane:^{i}PrOH = 99:1, flow rate 1 mL/min, λ = 210 nm), t_R (major) = 74.25 min, t_R (minor) = 105.20 min; *ee* = 93%.

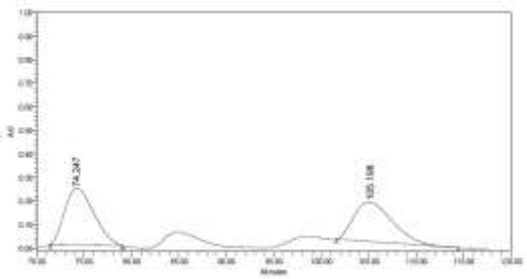

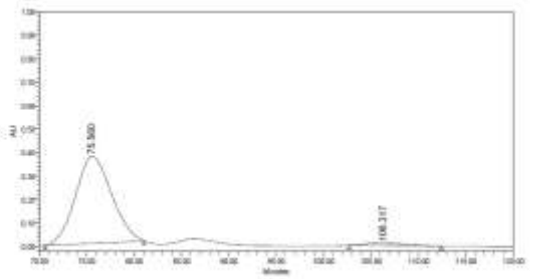

	RT	Height	Area	% Area
1	74.247	240065	51426808	52.07
2	105.198	166817	47346104	47.93

	RT	Height	Area	% Area
1	75.560	372306	98576963	96.58
2	106.317	12229	3491361	3.42

4.3 Organocatalytic enantioselective lactamization reaction

A reaction mixture of nitroalkene **13** (0.1 mmol), ketone **10** (0.8 mmol), the corresponding carboxylic acid **19** (0.11 mmol) and catalyst $X_LX_L^{Me}$-**9k** (6.05 mg, 0.01 mmol) was stirred

at room temperature. The progress of the reactions were monitored by TLC with EtOAc/hexane elution mixtures. After consumption of the nitroalkene, the crude product was purified by column chromatography over silica gel using EtOAc/hexane system as eluent to provide the Michael addition product first followed by the lactam product (elution order). TLC plates were stained with vanillin. Michael products showed blue colour and lactam products produced pink spots.

Nomenclature: For a better assignment of protons on NMR spectra, the IUPAC carbon order in octahydroindole-type rings has been used. The following lactam structures follow the same pattern.

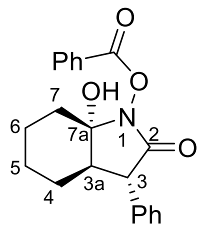

(3S,3aR,7aS)-7a-Hydroxy-2-oxo-3-phenyloctahydro-1H-indol-1-yl benzoate (**15a**).

Yield: 21.77 mg, 62%, white solid. Analytical and spectroscopic data were in good agreement with those reported in the literature.[10] ^{1}H NMR (400 MHz, $CDCl_3$) δ 8.11 (d, J = 7.8 Hz, 2H, ArH), 7.64 (t, J = 7.4 Hz, 1H, ArH), 7.48 (t, J = 7.7 Hz, 2H, ArH), 7.34 (dq, J = 15.2, 7.7 Hz, 5H, ArH), 3.53 (d, J = 9.7 Hz, 1H, C^{3}H), 3.17 (s, 1H, OH), 2.45 (t, J = 7.3 Hz, 1H, C^{3a}H), 2.22 (q, J = 5.3 Hz, 1H, C^{7}H), 1.88 – 1.78 (m, 1H, C^{4}H), 1.67 (m, 4H, C^{4}H, C^{5}H, C^{6}H and C^{7}H), 1.51 (m, 2H, C^{5}H and C^{6}H). HPLC (Daicel Chiralpak IB, hexane/^{i}PrOH = 90/10, flow rate 1.0 mL/min, λ = 210 nm), t_R (minor) = 15.67 min, t_R (major) = 20.17 min; ee = >99%.

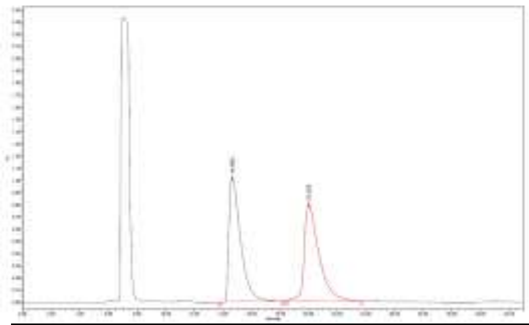

	Time	Area	% Height	% Area
**1**	14.668	57664653	56.30	50.74
**2**	20.003	55991546	43.70	49.26

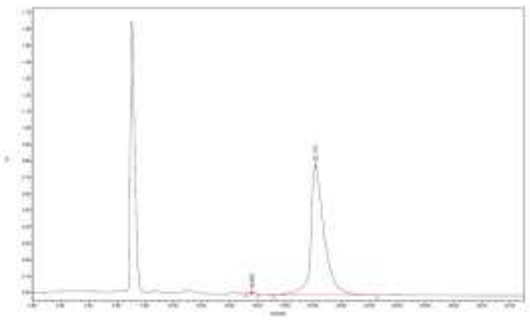

	Time	Area	% Height	% Area
**1**	15.667	114110	0.59	0.22
**2**	20.174	51671567	99.41	99.78

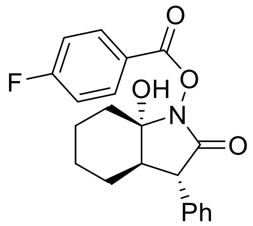

(3S,3aR,7aS)-7a-Hydroxy-2-oxo-3-phenyloctahydro-1H-indol-1-yl 4-fluorobenzoate (**15b**).

Yield: 18.46 mg, 50%, white solid. Analytical and spectroscopic data were in good agreement with those reported in the literature.[10] ^{1}H NMR (400 MHz, $CDCl_3$) δ 8.16 (dd, *J* = 8.4, 5.2 Hz, 2H, ArH), 7.46 – 7.26 (m, 5H, ArH), 7.18 (t, *J* = 8.5 Hz, 2H, ArH), 3.56 (d, *J* = 9.7 Hz, 1H, C^3H), 3.23 (s, 1H, OH), 2.47 (t, *J* = 7.0 Hz, 1H, C^{3a}H), 2.32 – 2.17 (m, 1H, C^7H), 1.95 – 1.80 (m, 1H, C^4H), 1.80 – 1.63 (m, 4H, C^4H, C^5H, C^6H and C^7H), 1.61 – 1.48 (m, 2H, C^5H and C^6H). HPLC (Daicel Chiralpak IB, hexane/^{i}PrOH = 90/10, flow rate 1.0 mL/min, λ = 210 nm), t_R (major) = 25.02 min; ee = >99%.

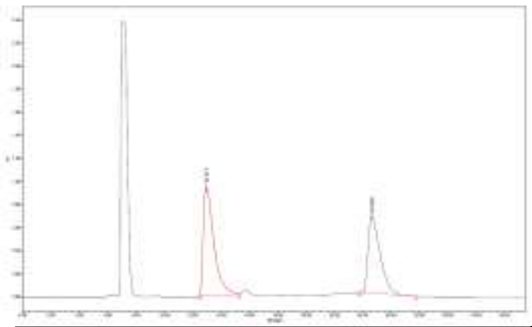

	Time	Area	% Height	% Area
1	12.971	49160238	58.59	55.60
2	24.643	39252427	41.41	44.40

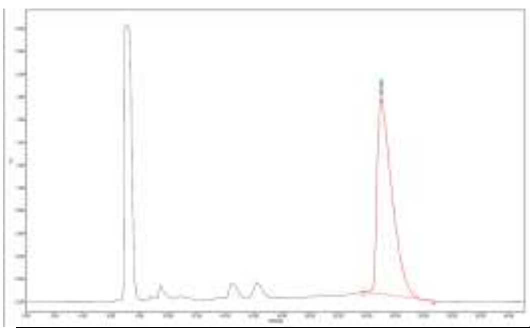

	Time	Area	% Height	% Area
1	25.023	125605278	100.00	100.00

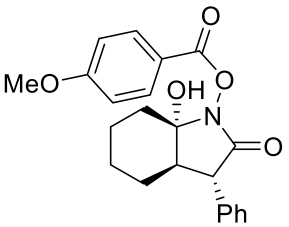

(3S,3aR,7aS)-7a-Hydroxy-2-oxo-3-phenyloctahydro-1H-indol-1-yl 4-methoxybenzoate (**15c**).

Yield: 20.20 mg, 53%, white solid Analytical and spectroscopic data were in good agreement with those reported in the literature.[10] ^{1}H NMR (400 MHz, $CDCl_3$) δ 7.98 (d, *J* = 8.5 Hz, 2H, ArH), 7.43 – 7.09 (m, 5H, ArH), 6.86 (d, *J* = 8.5 Hz, 2H, ArH), 3.80 (s, 3H, OMe), 3.45 (d, *J* = 9.6 Hz, 1H, C^3H), 3.25 (s, 1H, OH), 2.52 – 2.24 (m, 1H, C^{3a}H), 2.23 – 2.08 (m, 1H, C^7H), 1.82 – 1.70 (m, 1H, C^4H), 1.69 – 1.53 (m, 4H, C^4H, C^5H, C^6H and C^7H), 1.53 – 1.33 (m, 2H, C^5H and C^6H). HPLC (Daicel Chiralpak IB, hexane/^{i}PrOH = 80/20, flow rate 1.0 mL/min, λ = 210 nm), t_R (major) = 18.67 min; ee = >99%.

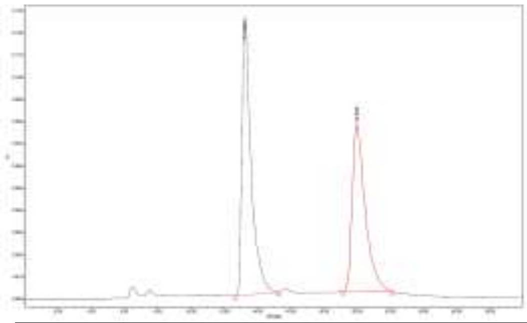

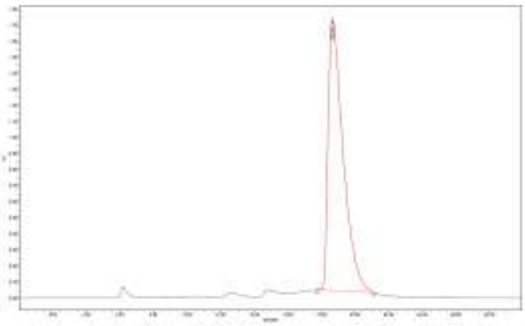

	Time	Area	% Height	% Area
1	13.249	4808086	62.56	54.03
2	19.996	4090627	37.44	45.97

	Time	Area	% Height	% Area
1	18.667	104735105	100.00	100.00

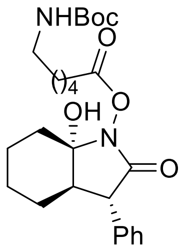

(3S,3aR,7aS)-7a-Hydroxy-2-oxo-3-phenyloctahydro-1H-indol-1-yl 3-((tert-butoxycarbonyl)amino)hexanoate (**15d**).

Yield: 22.09 mg, 48%, white solid. Analytical and spectroscopic data were in good agreement with those reported in the literature.[10] ^{1}H NMR (500 MHz, $CDCl_3$) δ 7.40 – 7.31 (m, 2H, ArH), 7.30 – 7.22 (m, 3H, ArH), 4.63 (s, 1H, NH), 3.45 (d, J = 9.7 Hz, 1H, C^3H), 3.18 (s, 1H, OH), 3.14 – 3.05 (m, 2H, $NHCH_2$), 2.55 (hept, J = 7.9, 7.3 Hz, 2H, $COCH_2$), 2.44 – 2.30 (m, 1H, $C^{3a}H$), 2.19 – 2.10 (m, 1H, C^7H), 1.91 – 1.71 (m, 3H, C^4H and CH_2), 1.68 – 1.55 (m, 5H, C^4H, C^5H, C^6H, C^7H and CH_2), 1.56 – 1.46 (m, 5H, C^5H, C^6H and CH_2), 1.43 (s, 9H, $(CH_3)_3$). 483.2473. HPLC (Daicel Chiralpak IB, hexane/^{i}PrOH = 90/10, flow rate 1.0 mL/min, λ = 210 nm), t_R (major) = 23.38 min; ee = >99%.

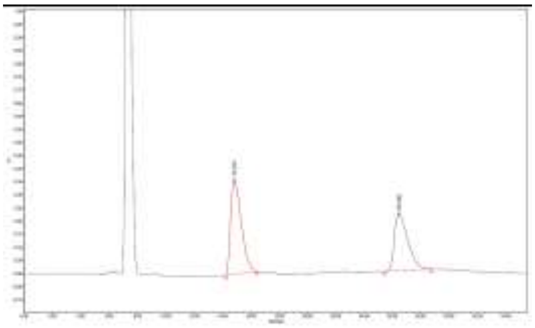

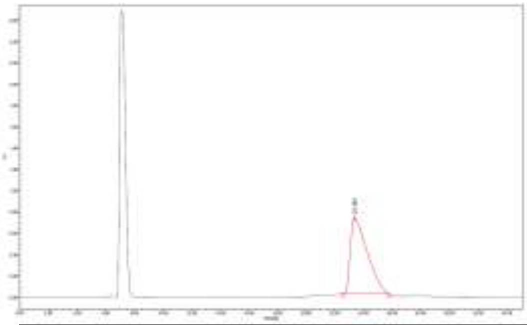

	Time	Area	% Height	% Area
1	14.827	18739372	62.53	56.69
2	26.463	14315018	37.47	43.31

	Time	Area	% Height	% Area
1	23.385	58249025	100.00	100.00

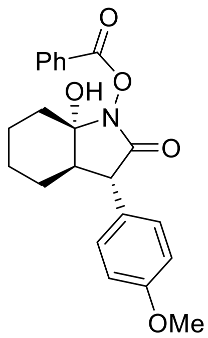

(3S,3aR,7aS)-7a-Hydroxy-3-(4-methoxyphenyl)-2-oxooctahydro-1H-indol-1-yl benzoate (**15e**).

Yield: 18.30 mg, 48%, white solid Analytical and spectroscopic data were in good agreement with those reported in the literature.[10] ^{1}H NMR (400 MHz, $CDCl_3$) δ 8.13 (d, *J* = 7.8 Hz, 2H, ArH), 7.64 (t, *J* = 7.4 Hz, 1H, ArH), 7.49 (t, *J* = 7.7 Hz, 2H, ArH), 7.25 (d, *J* = 8.9 Hz, 2H, ArH), 6.91 (d, *J* = 8.3 Hz, 2H, ArH), 3.80 (s, 3H, OMe), 3.49 (d, *J* = 9.5 Hz, 1H, C^{3}H), 2.88 (s, 1H, OH), 2.40 (t, *J* = 7.5 Hz, 1H, C^{3a}H), 2.20 (t, *J* = 9.0 Hz, 1H, C^{7}H), 1.85 (dd, *J* = 10.7, 5.9 Hz, 1H, C^{4}H), 1.67 (dd, *J* = 26.9, 15.2 Hz, 4H, C^{4}H, C^{5}H, C^{6}H and C^{7}H), 1.53 (d, *J* = 10.3 Hz, 2H, C^{5}H and C^{6}H). HPLC (Daicel Chiralpak IB, hexane/^{i}PrOH = 80/20, flow rate 1.0 mL/min, λ = 210 nm), t_R (major) = 17.41 min; ee = >99%.

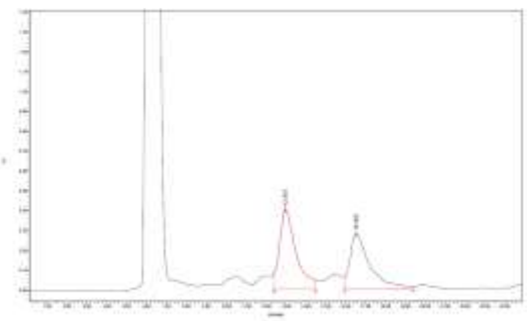

	Time	Area	% Height	% Area
1	12.975	23251604	59.28	54.93
2	16.525	19078763	40.72	45.07

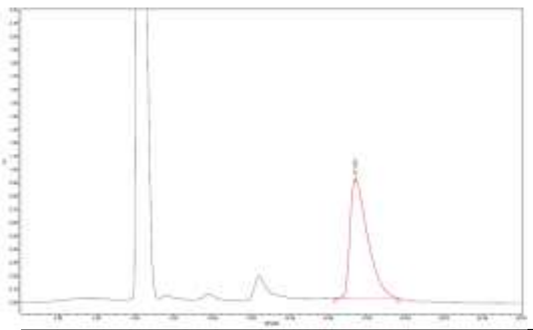

	Time	Area	% Height	% Area
1	17.413	56550758	100.00	100.00

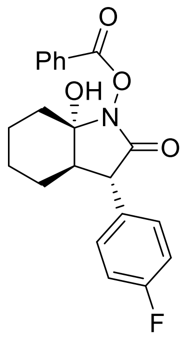

(3S,3aR,7aS)-3-(4-Fluorophenyl)-7a-hydroxy-2-oxooctahydro-1H-indol-1-yl benzoate (**15f**).

Yield: 16.24 mg, 44%, white solid. Analytical and spectroscopic data were in good agreement with those reported in the literature.[10] ^{1}H NMR (400 MHz, $CDCl_3$) δ 8.11 (d, *J* = 7.8 Hz, 2H, ArH), 7.65 (t, *J* = 7.6 Hz, 1H, ArH), 7.49 (t, *J* = 7.7 Hz, 2H, ArH), 7.29 (dd, *J* = 8.1, 5.1 Hz, 2H, ArH), 7.05 (t, *J* = 8.5 Hz, 2H, ArH), 3.51 (d, *J* = 9.3 Hz, 1H, C^{3}H), 3.06 (s, 1H, OH), 2.53 – 2.36 (m, 1H, C^{3a}H), 2.30 – 2.13 (m, 1H, C^{7}H), 1.96 – 1.81 (m, 1H, C^{4}H), 1.68 (q, *J* = 13.6 Hz, 4H, C^{4}H, C^{5}H, C^{6}H and C^{7}H), 1.51 (q, *J* = 9.9, 9.3 Hz, 2H, C^{5}H and C^{6}H). HPLC (Daicel Chiralpak IB, hexane/^{i}PrOH = 90/10, flow rate 1.0 mL/min, λ = 210 nm), t_R (major) = 18.39 min; ee = >99%.

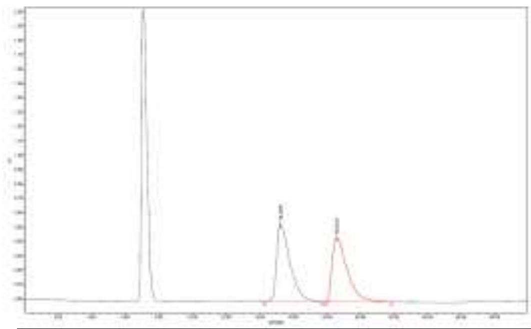

	Time	Area	% Height	% Area
**1**	15.265	29836339	54.94	52.24
**2**	18.610	27274302	45.06	47.76

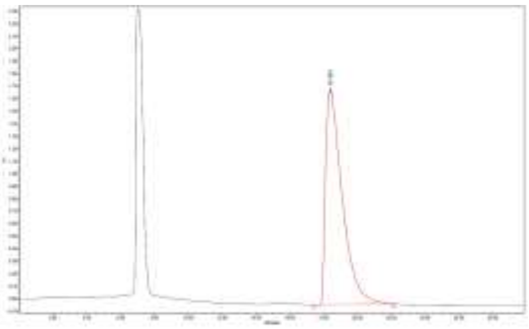

	Time	Area	% Height	% Area
**1**	18.394	117559812	100.00	100.00

Additional procedure for the synthesis of 15g

1,4-Cyclohexanedione monoethylene acetal **10d** (124.96 mg, 0.8 mmol), (*E*)-5-(2-nitrovinyl) benzo[*d*][1,3]dioxole **13d** (38.64 mg, 0.2 mmol), benzoic acid **16a** (36.64 mg, 0.3 mmol) and $X_LX_L^{Me}$-**9k** (24.20 mg, 0.04 mmol) were dissolved in 600 µL of toluene and stirred for 72 hours at 45 ºC. Then, the solvent was evaporated and the afforded crude mixture was purified by column chromatography over silica gel using EtOAc/hexane 1/3 as eluent to provide **15g** as a white solid (70.8 mg, 78%). Once the enantiomeric excess (>99% ee) was verified after column chromatography the other option to purify this adduct was tested: the solvent was evaporated and the product precipitated and filtered from a solution of ethyl acetate enhancing the yield of the process to 82%.

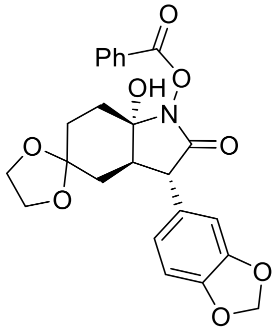

(3S,3aR,7aS)-3-(Benzo[d][1,3]dioxol-5-yl)-7a-hydroxy-2-oxohexahydrospiro[indole-5,2'-[1,3]dioxolan]-1(4H)-yl benzoate (**15g**). Yield: 74.0 mg, 82%, white solid. Analytical and spectroscopic data were in good agreement with those reported in the literature.[10] ^{1}H NMR (400 MHz, $CDCl_3$) δ 8.13 (d, J = 7.7 Hz, 2H, ArH), 7.65 (t, J = 7.6 Hz, 1H, ArH), 7.50 (t, J = 7.6 Hz, 2H, ArH), 6.78 (q, J = 8.1 Hz, 3H, ArH), 5.96 (s, 2H, OCH_2O), 4.15 – 3.90 (m, 5H, C^3H and $O(CH_2)_2O$), 2.81 (s, 1H, OH), 2.48 (t, J = 8.4 Hz, 1H, $C^{3a}H$), 2.28 (d, J = 13.8 Hz, 1H, C^7H), 2.03 (dd, J = 14.7, 6.7 Hz, 2H, C^4H and C^7H), 1.86 (td, J = 13.5, 4.0 Hz, 1H, C^6H), 1.77 (dd, J = 14.0, 8.1 Hz, 2H, C^4H and C^6H). HPLC (Daicel Chiralpak IC, hexane/^{i}PrOH = 60/40, flow rate 1.0 mL/min, λ = 210 nm), t_R (major) = 20.31 min, t_R (minor) = 29.25 min; ee = >99%.

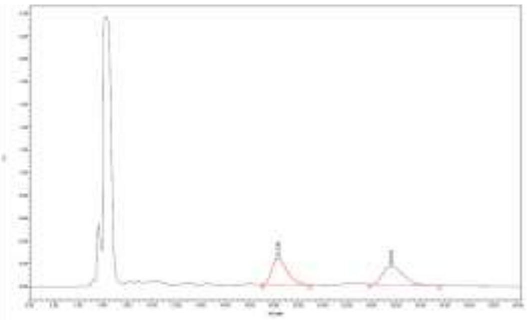

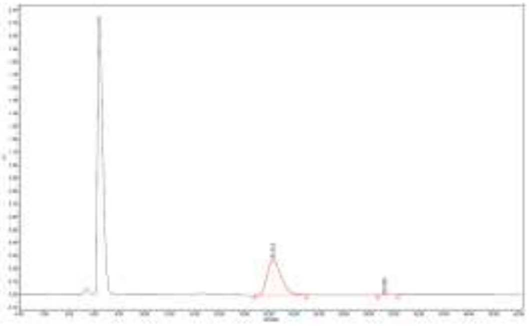

	Time	Area	% Height	% Area
**1**	20.339	19952866	57.98	50.89
**2**	29.611	19254902	42.02	49.11

	Time	Area	% Height	% Area
**1**	20.312	21166513	99.98	99.96
**2**	29.250	3671	0.02	0.04

5 Pseudo-first order linear plots of organocatalyzed reactions

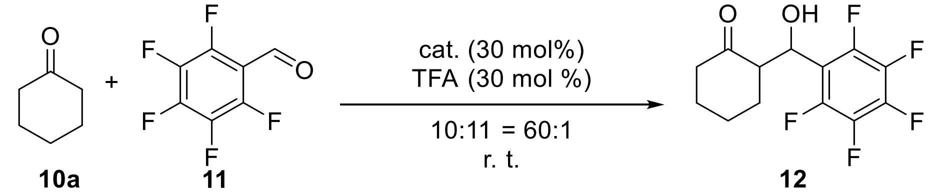

<u>X_LX_L-**9b**</u>

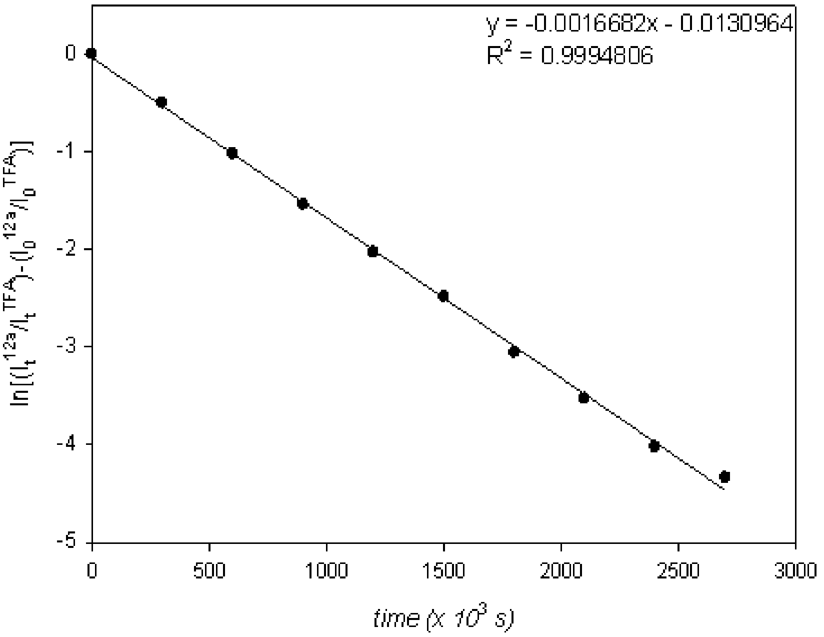

<u>N_LN_L-**9b**</u>

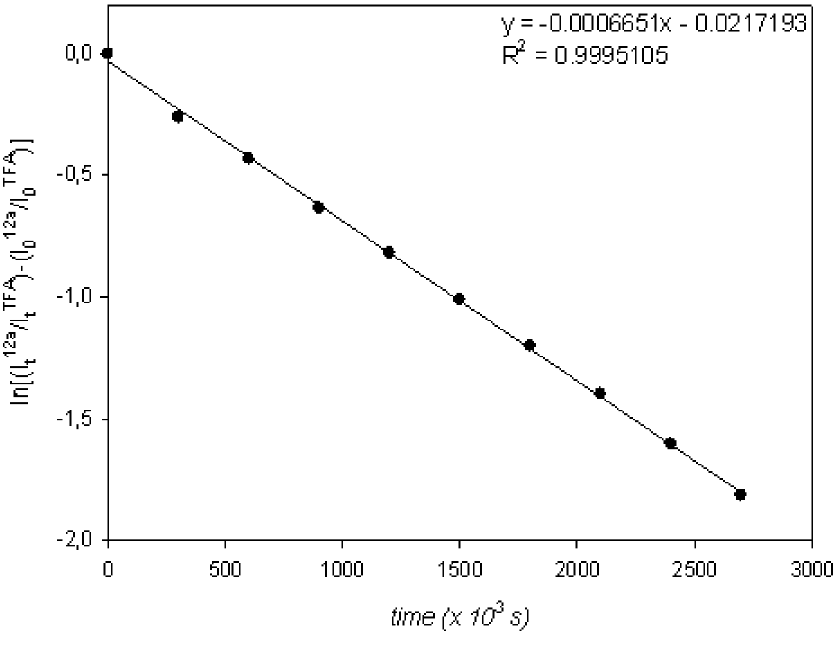

$X_L N_L$-**9b**

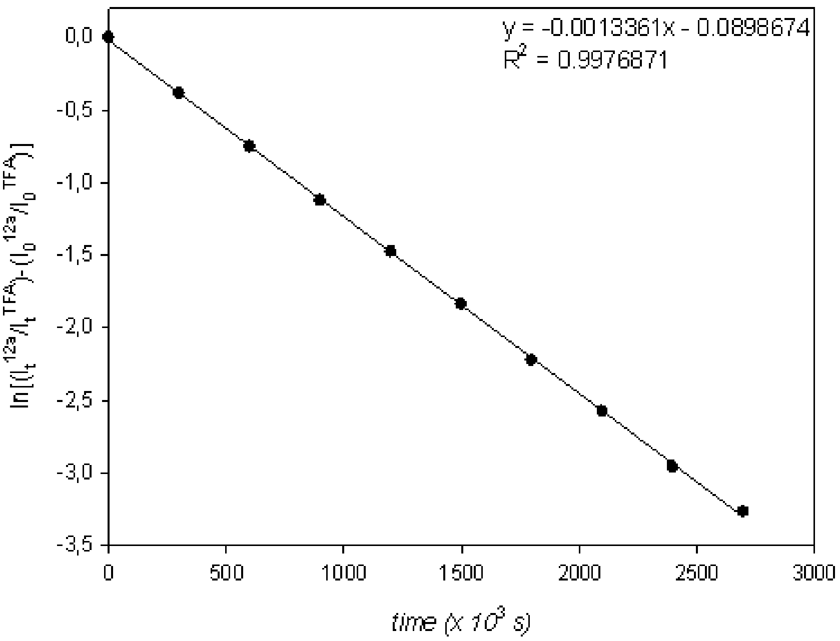

$N_L X_L$-**9b**

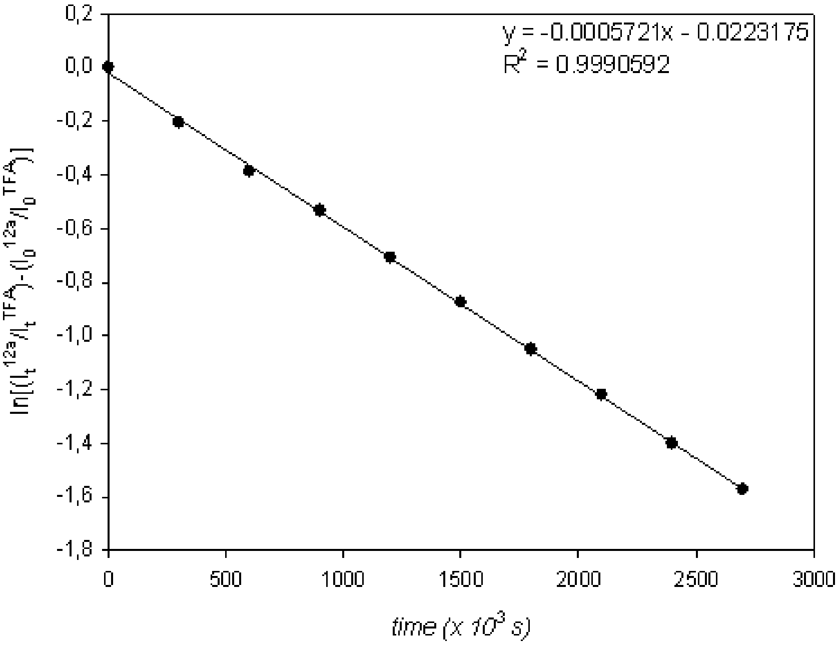

6 Standard deviations and error calculations[11]

Errors for k_{obs} were calculated employing the standard deviation (*s*), equation 1:

$$s = \sqrt{\frac{\sum_{i=1}^{n}(X_i - \bar{X})^2}{n-1}} \qquad (1)$$

where

X stands for the different k_{obs} values calculated by ^{19}F-NMR.

$\bar{X}$ is the average k_{obs}

n is the number of values (k_{obs}) in the final calculation

7 NMR spectra

exo-L-**5b**

^{1}**H NMR** (500 MHz, $CDCl_3$)

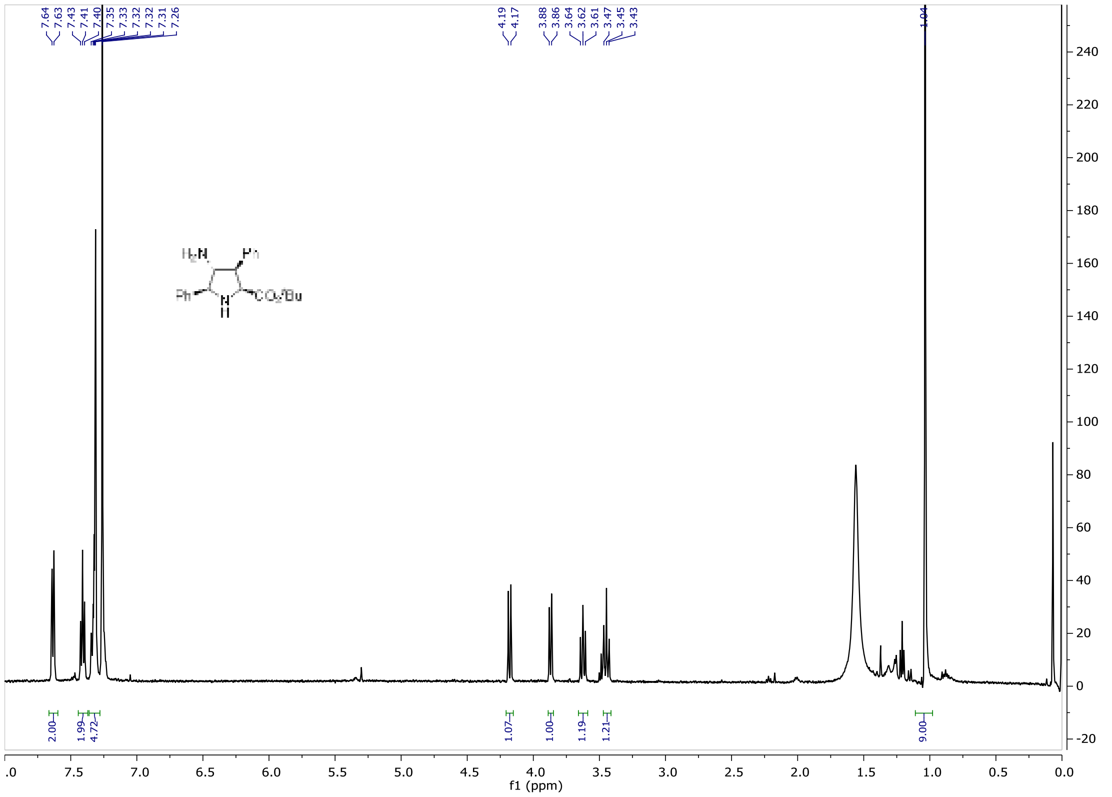

^{13}**C NMR** (126 MHz, $CDCl_3$)

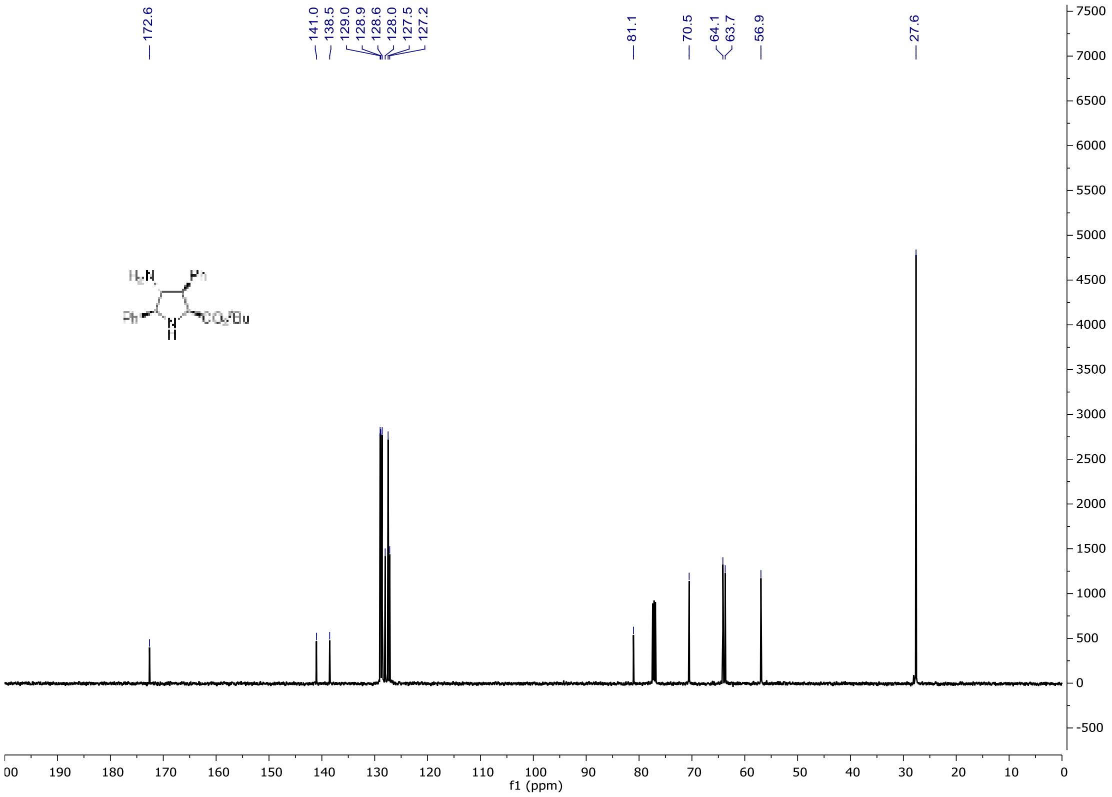

COSY ($CDCl_3$)

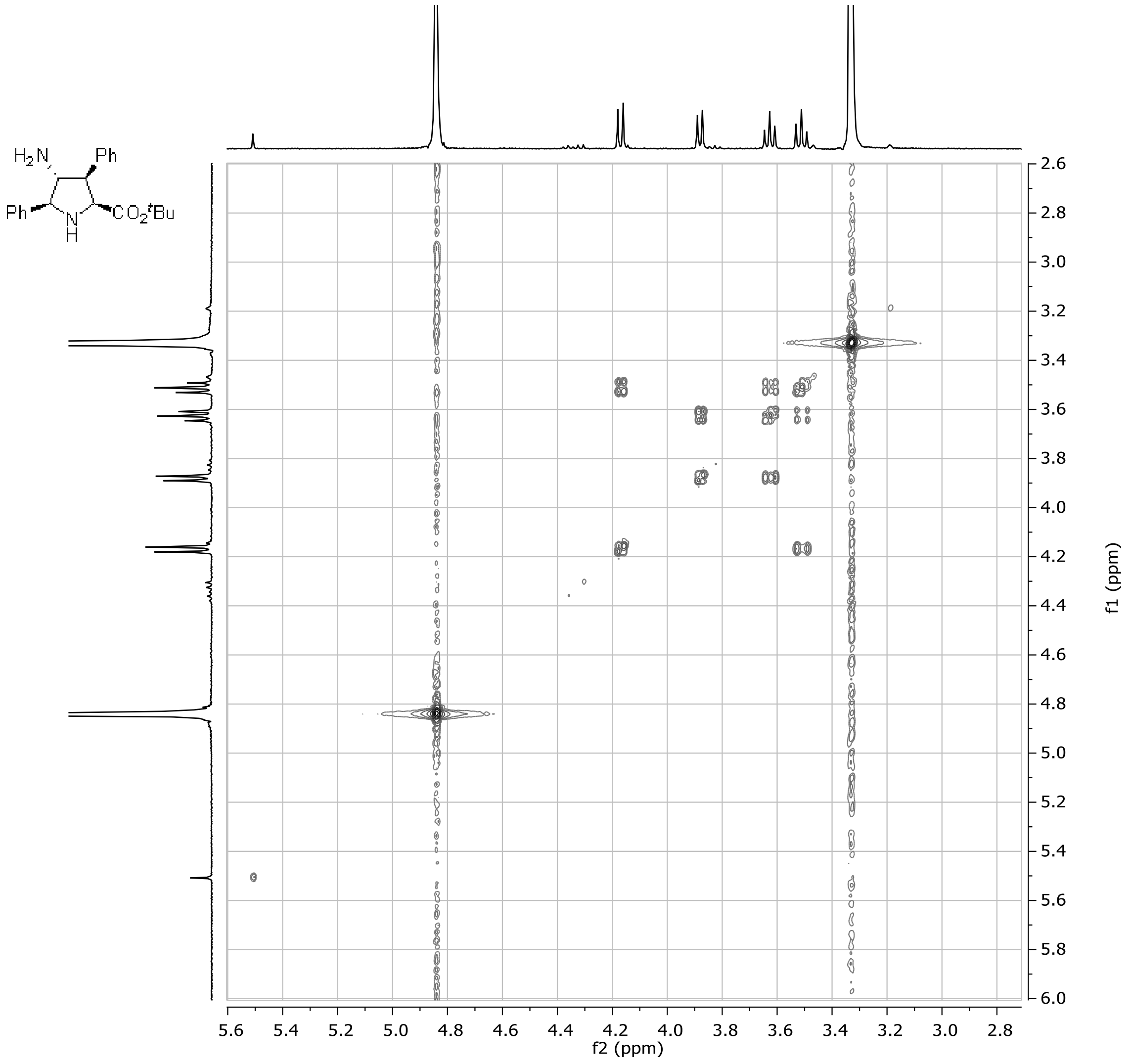

exo-L-**8a**

¹H NMR (400 MHz, $CDCl_3$)

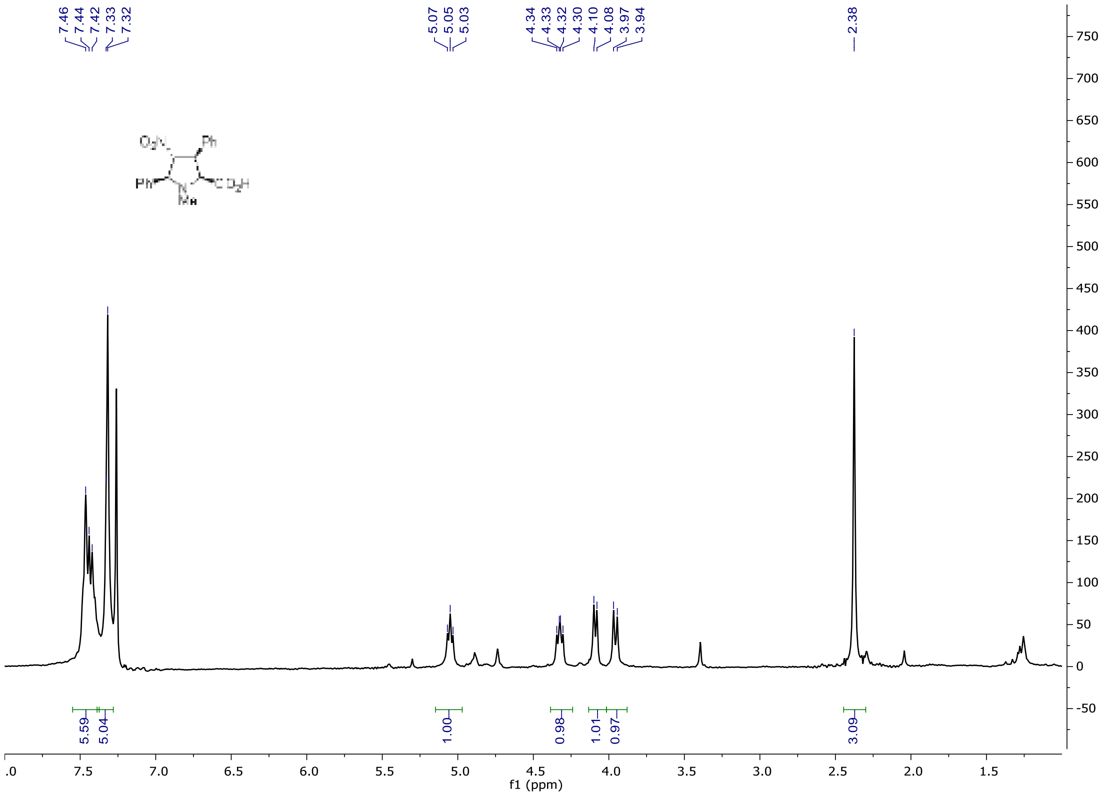

¹³C NMR (101 MHz, $CDCl_3$)

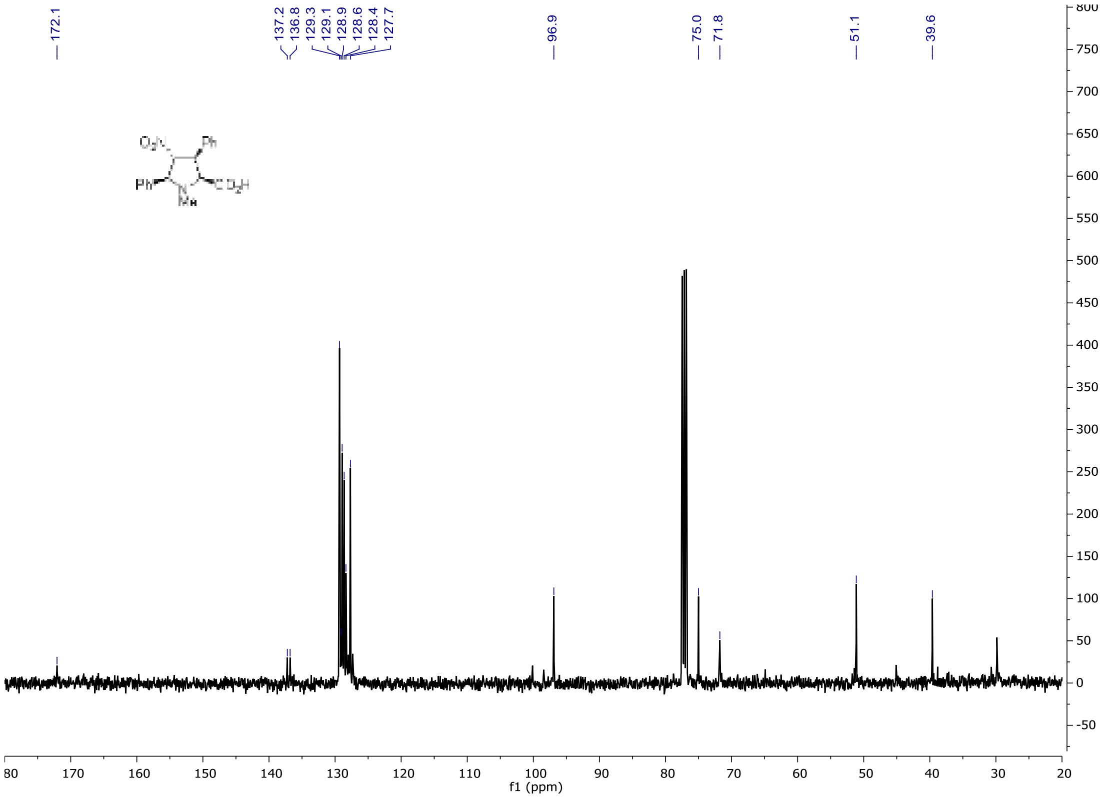

COSY ($CDCl_3$)

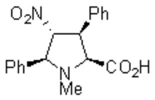

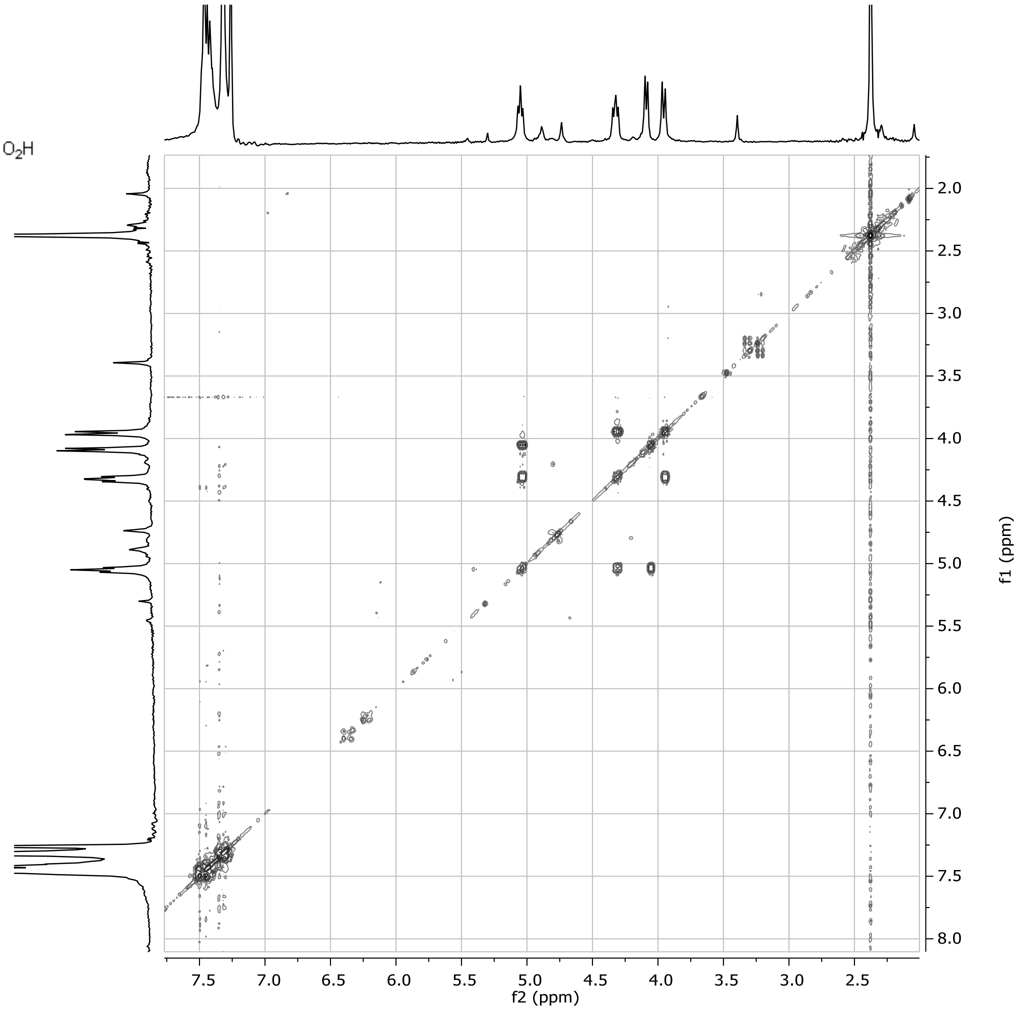

XLXL-9a

¹H NMR (500 MHz, $CDCl_3$)

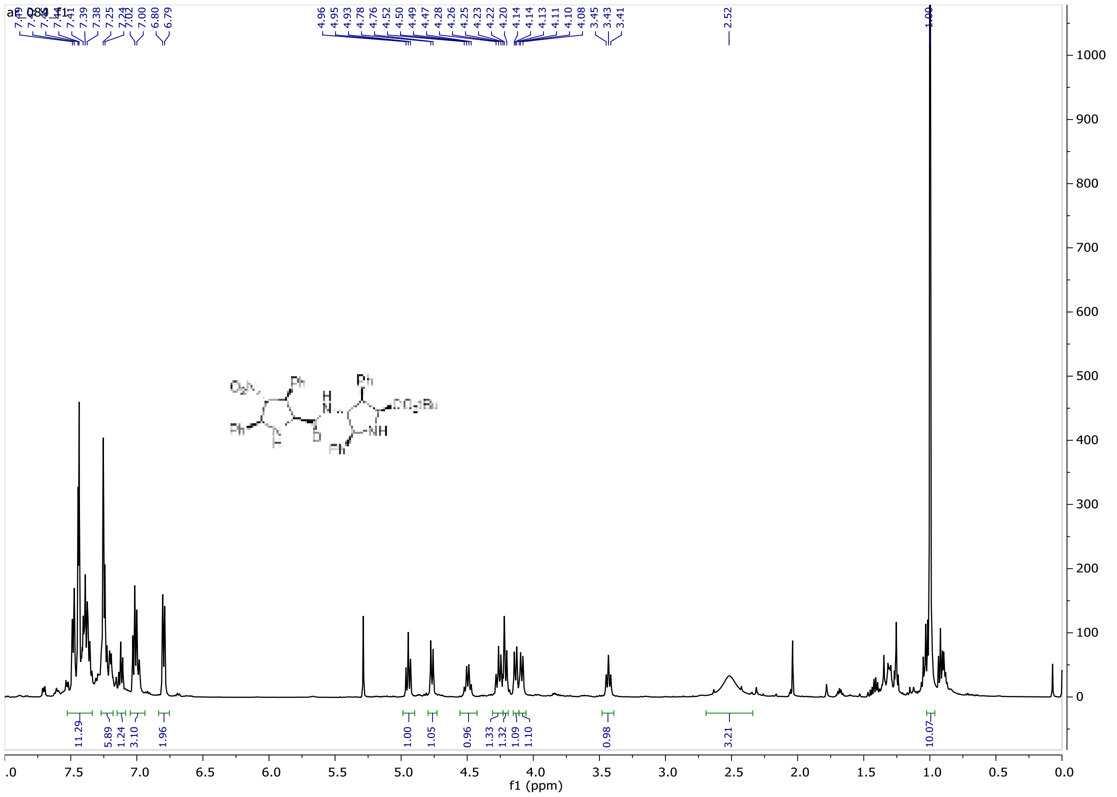

¹³C NMR (126 MHz, $CDCl_3$)

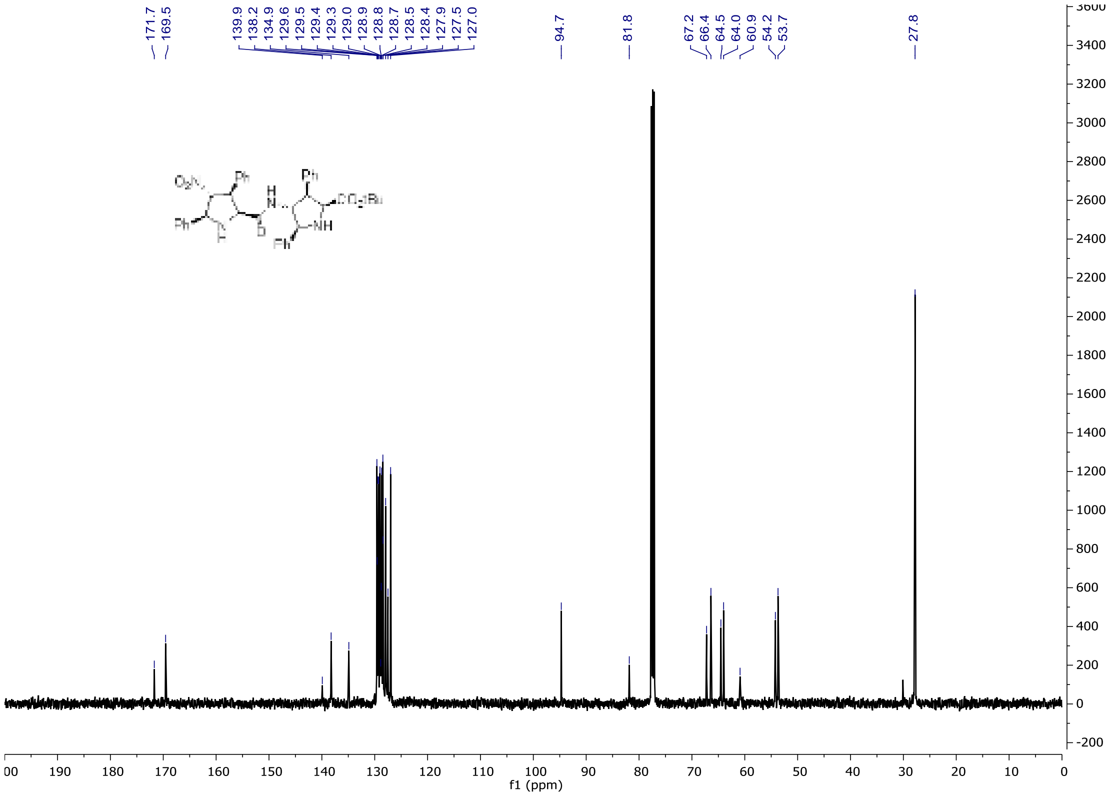

COSY ($CDCl_3$)

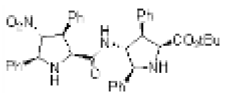

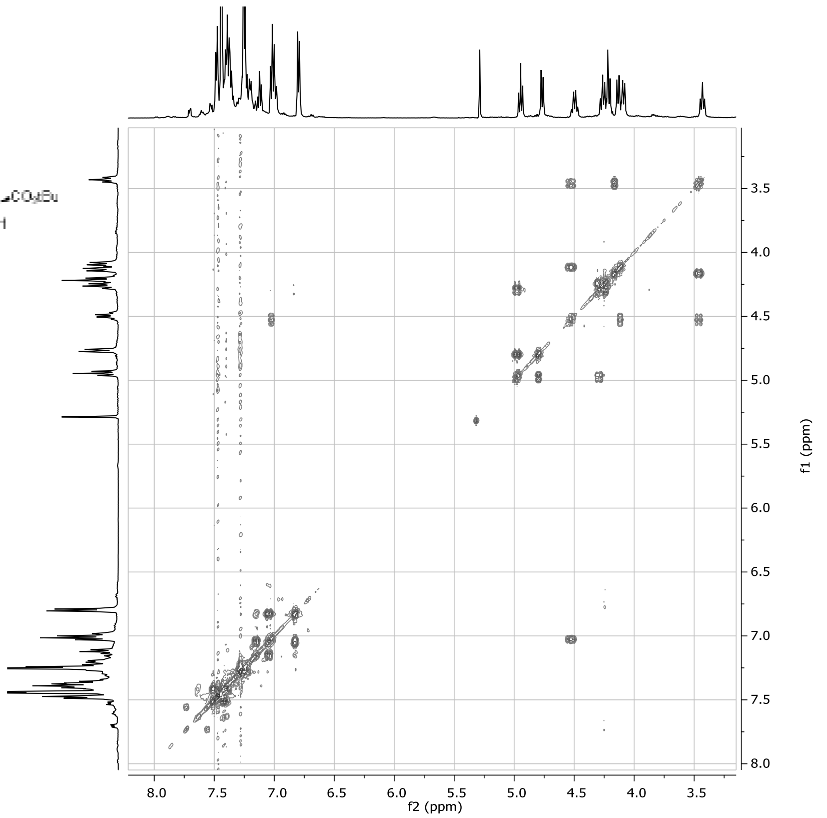

X_LX_L-**9b**

^{1}H NMR (500 MHz, $CDCl_3$)

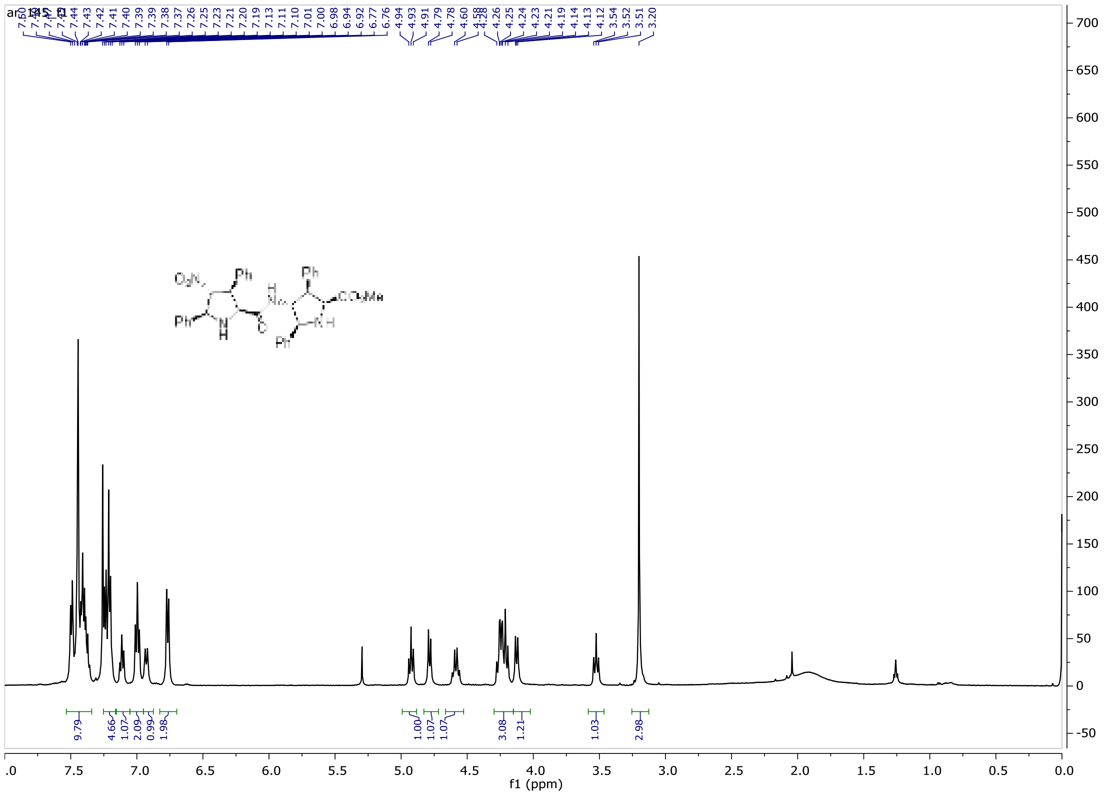

^{13}C NMR (126 MHz, $CDCl_3$)

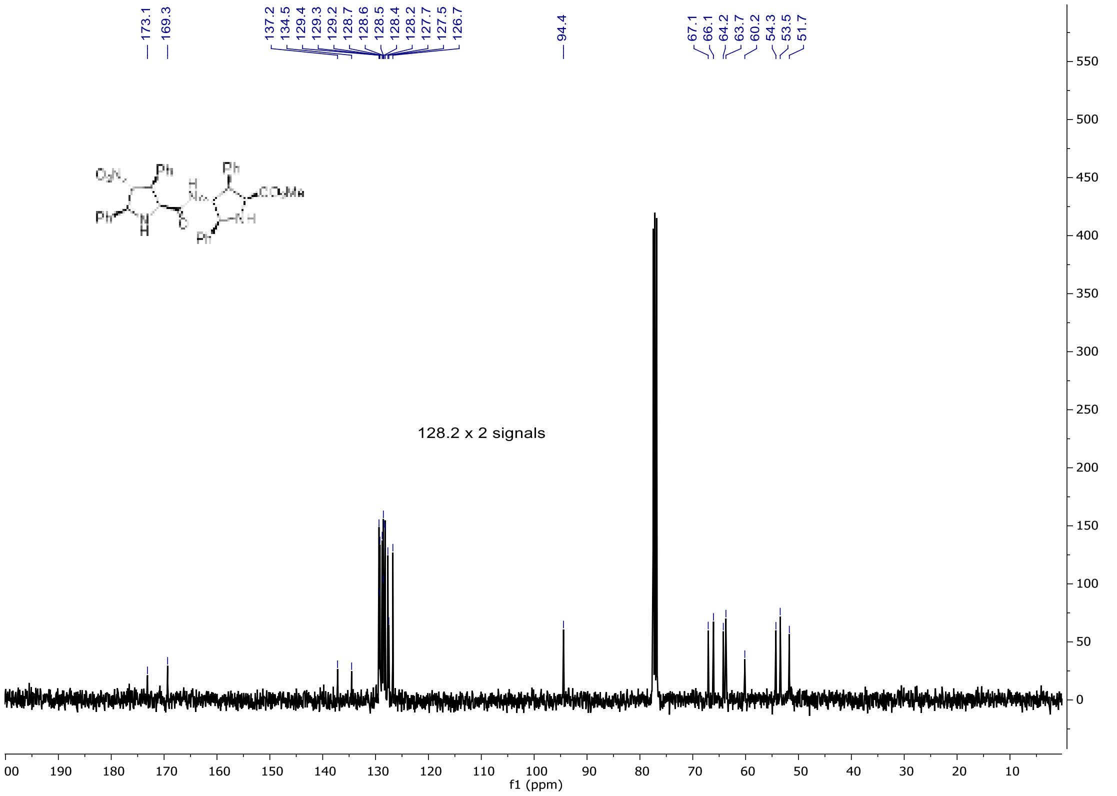

COSY ($CDCl_3$)

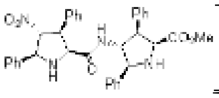

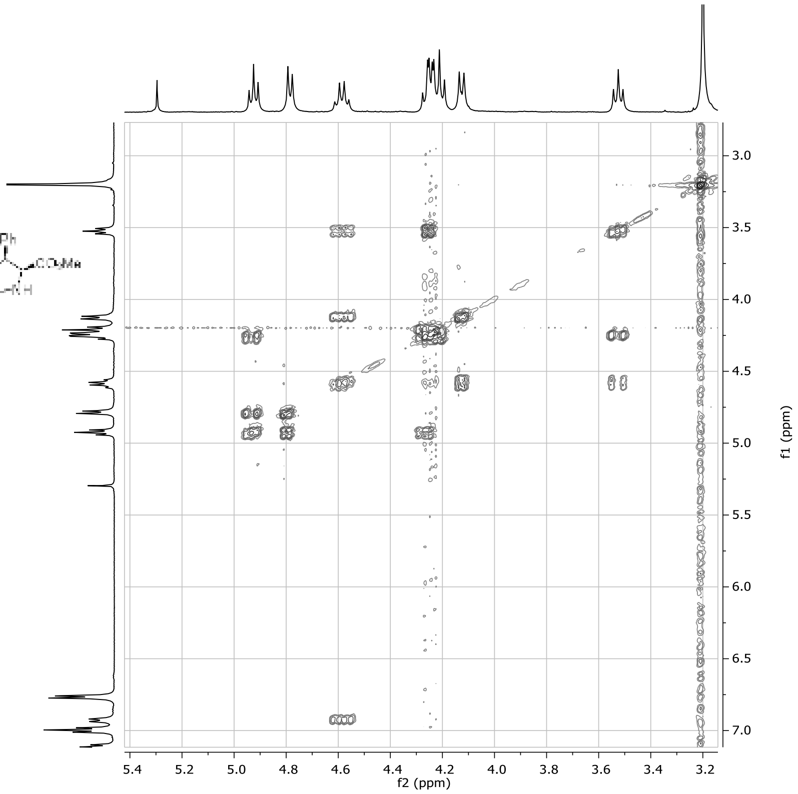

N$_L$N$_L$-**9c**

^{1}H NMR (500 MHz, $CDCl_3$)

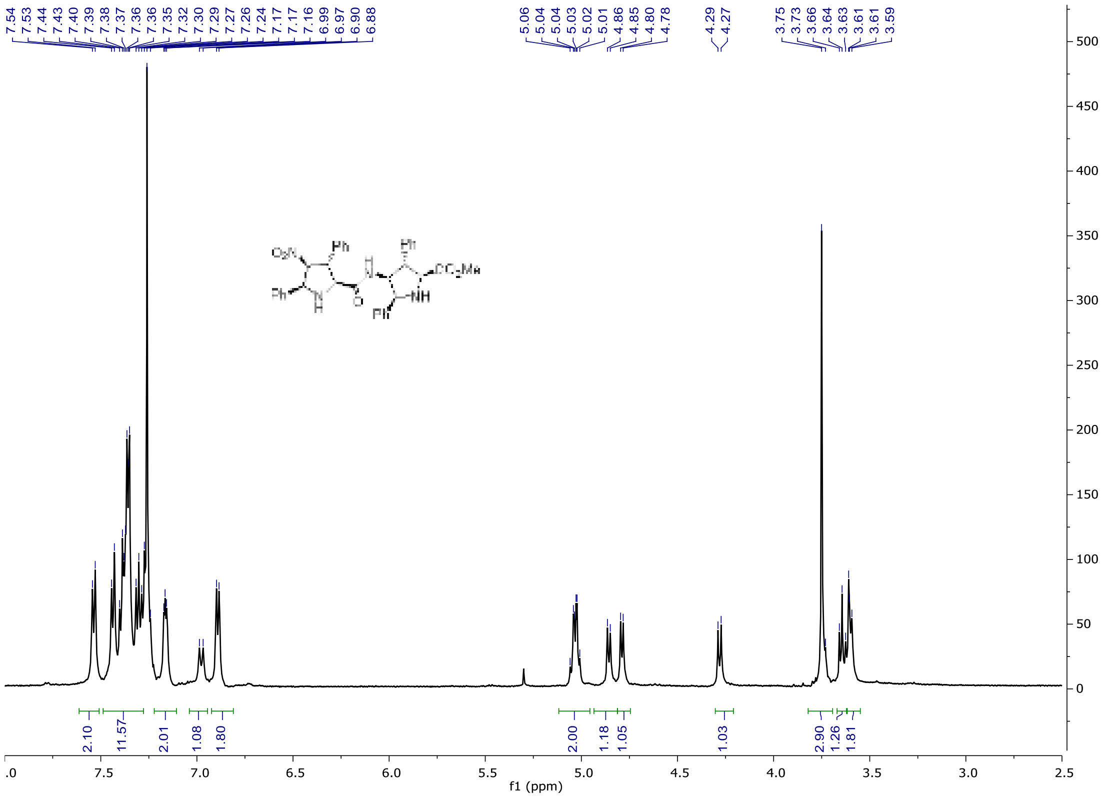

^{13}C NMR (126 MHz, $CDCl_3$)

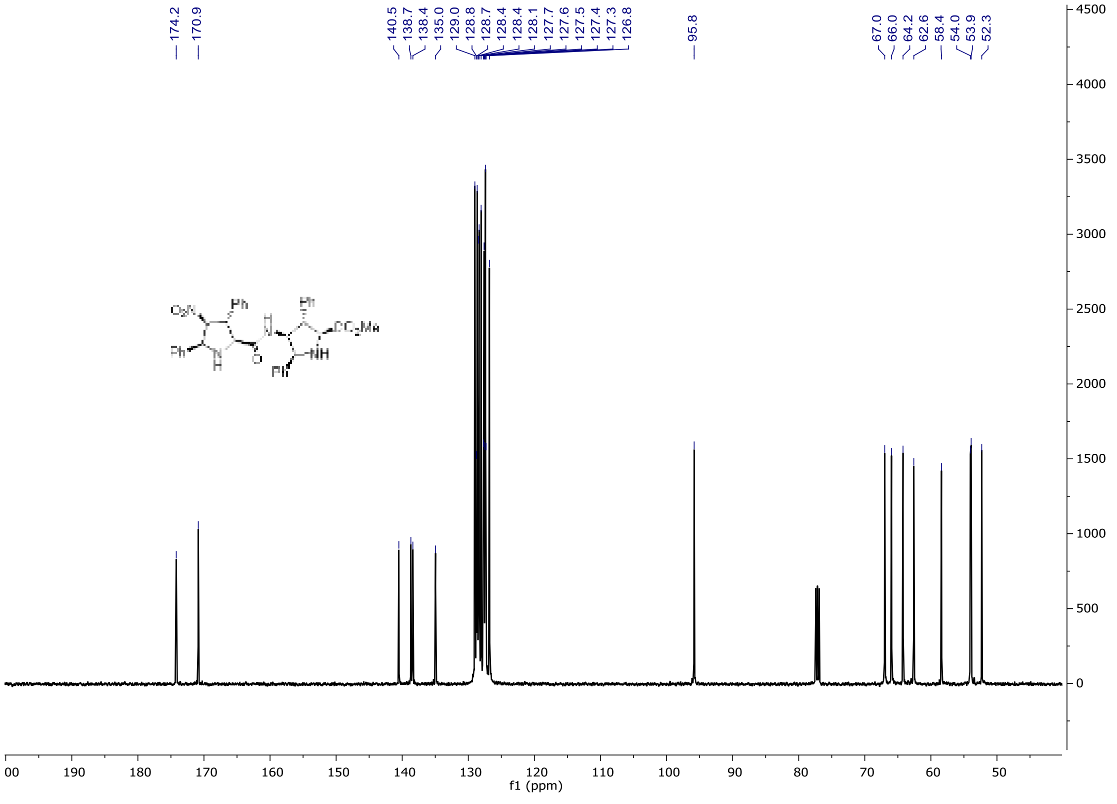

COSY ($CDCl_3$)

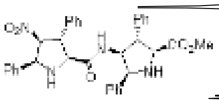

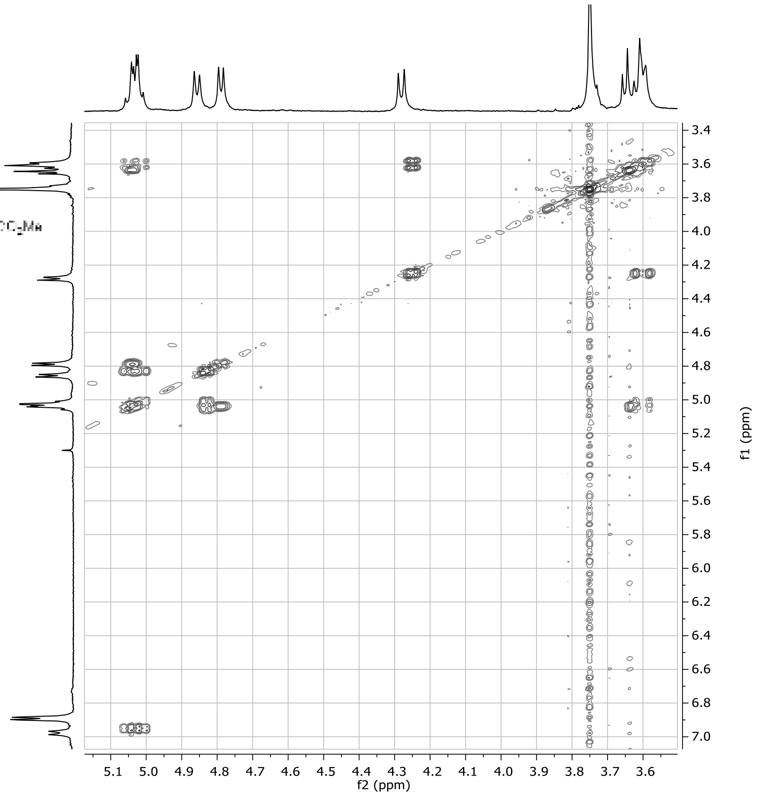

X_LX_D-**9d**

^{1}H NMR (500 MHz, $CDCl_3$)

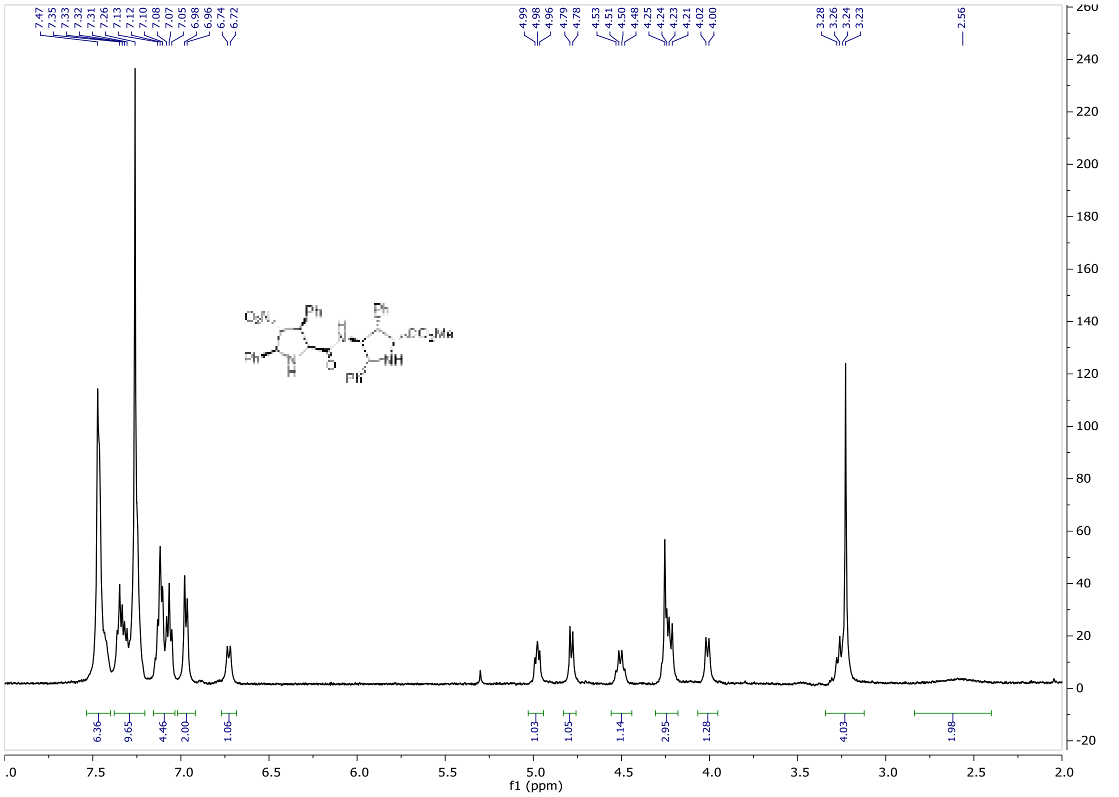

^{13}C NMR (126 MHz, $CDCl_3$)

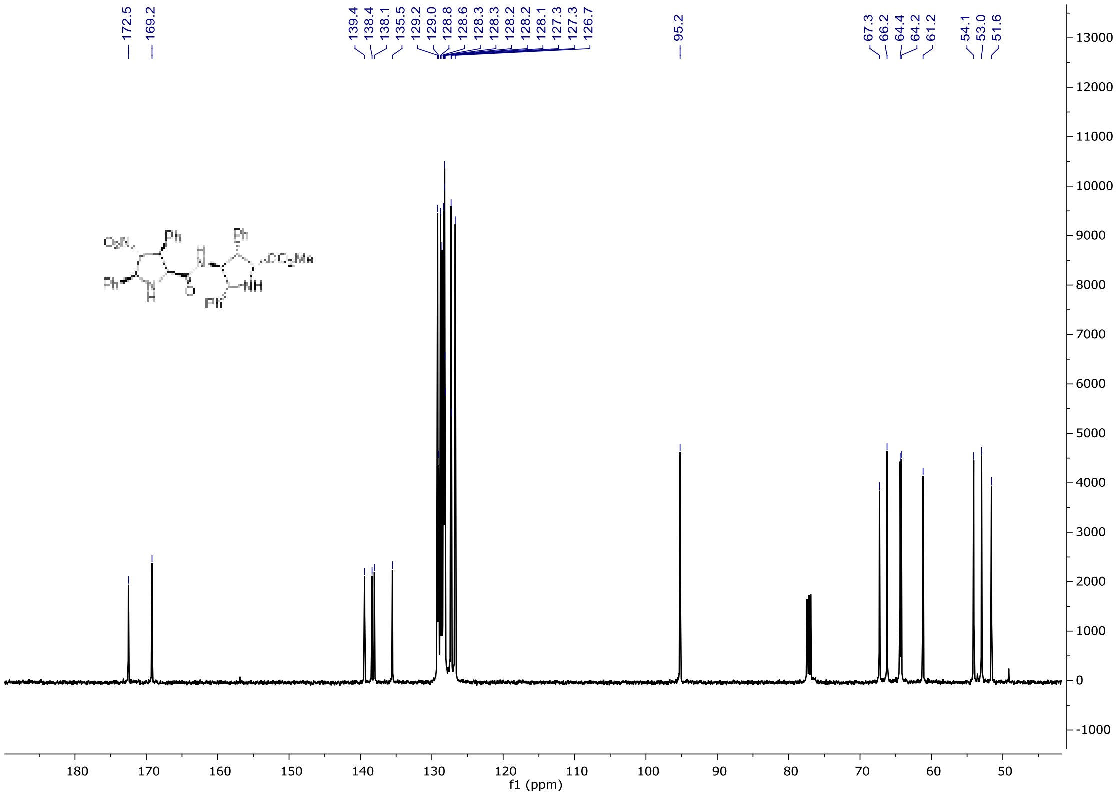

COSY ($CDCl_3$)

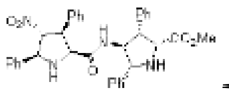

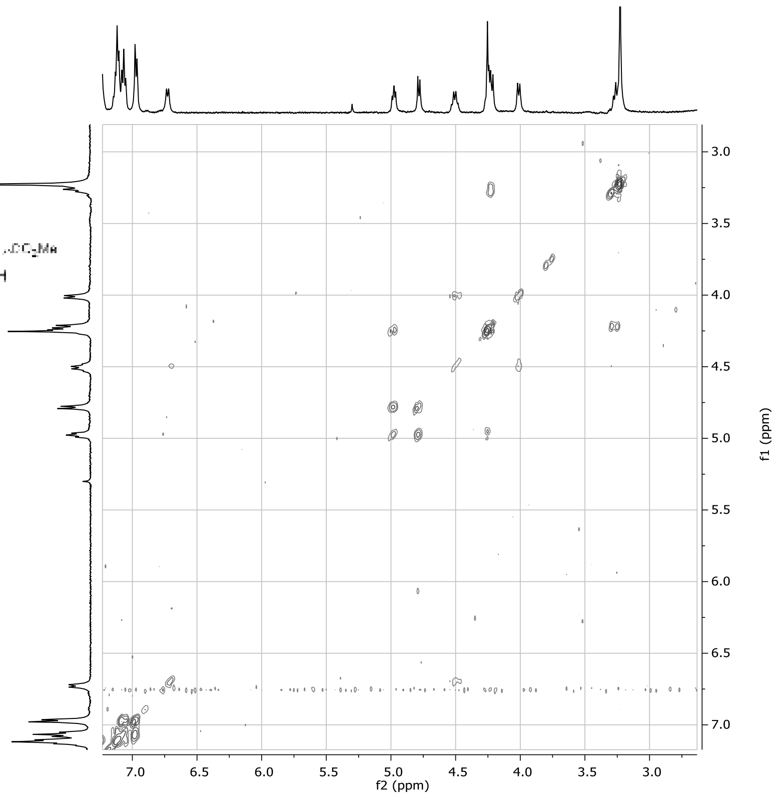

N$_L$N$_D$-**9e**

^{1}H NMR (500 MHz, $CDCl_3$)

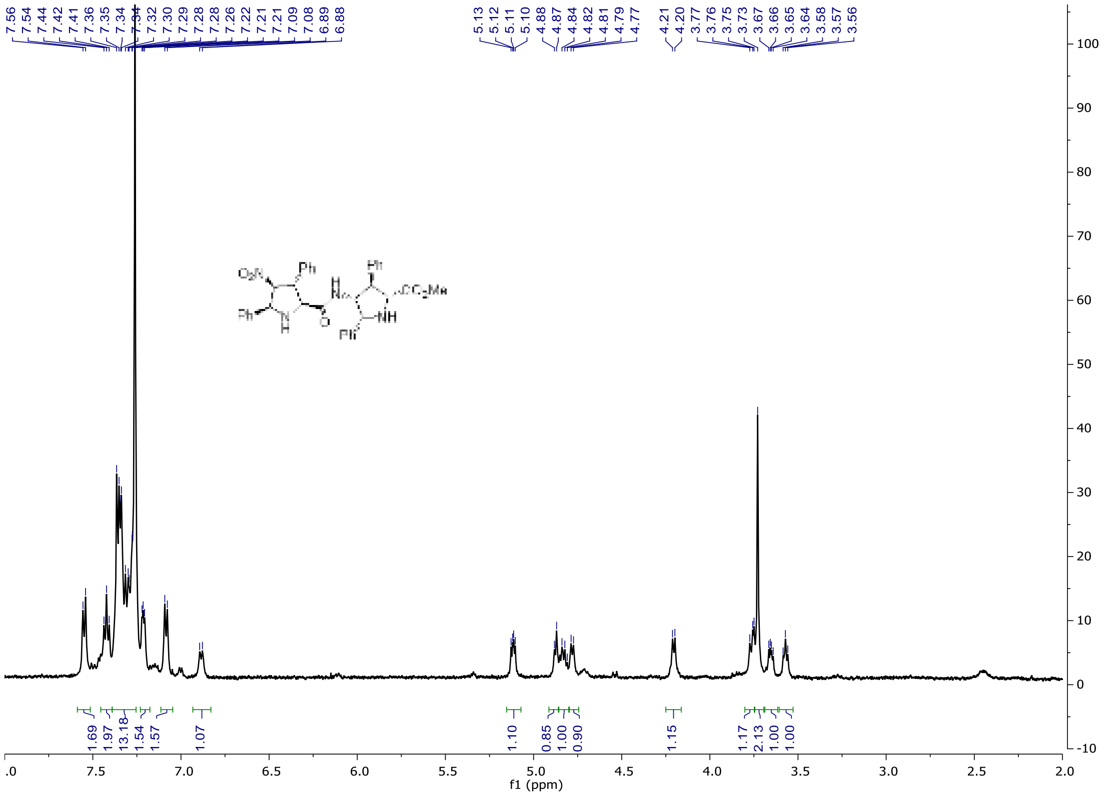

^{13}C NMR (126 MHz, $CDCl_3$)

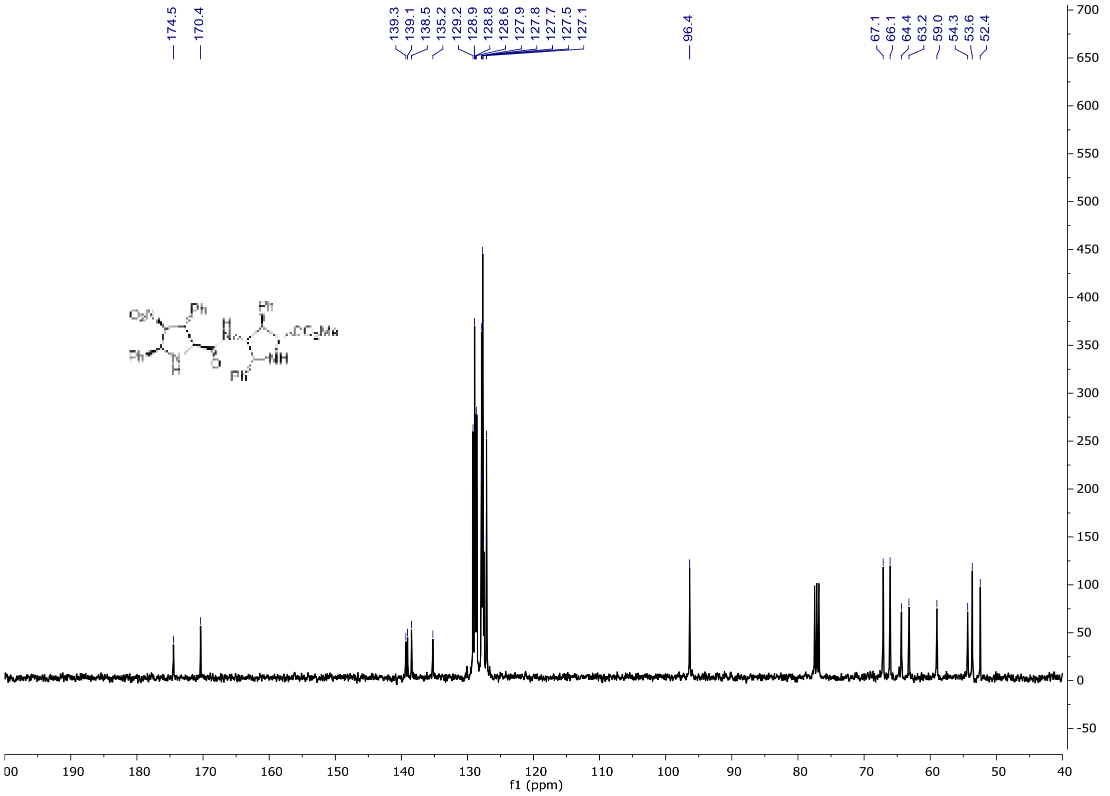

COSY ($CDCl_3$)

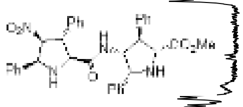

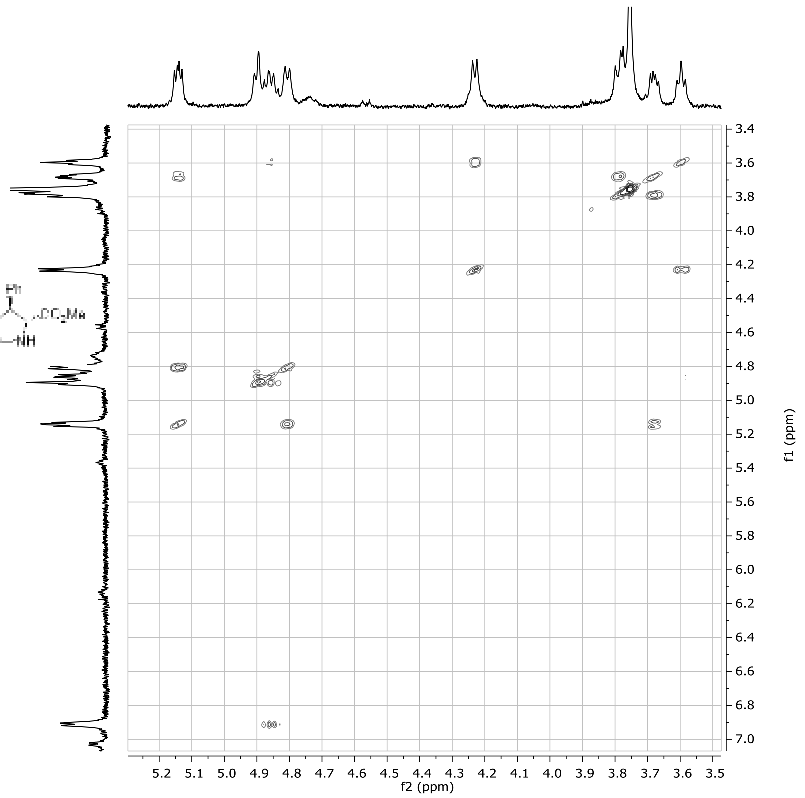

X<u>L</u>N<u>L</u>-**9f**

^{1}H NMR (500 MHz, $CDCl_3$)

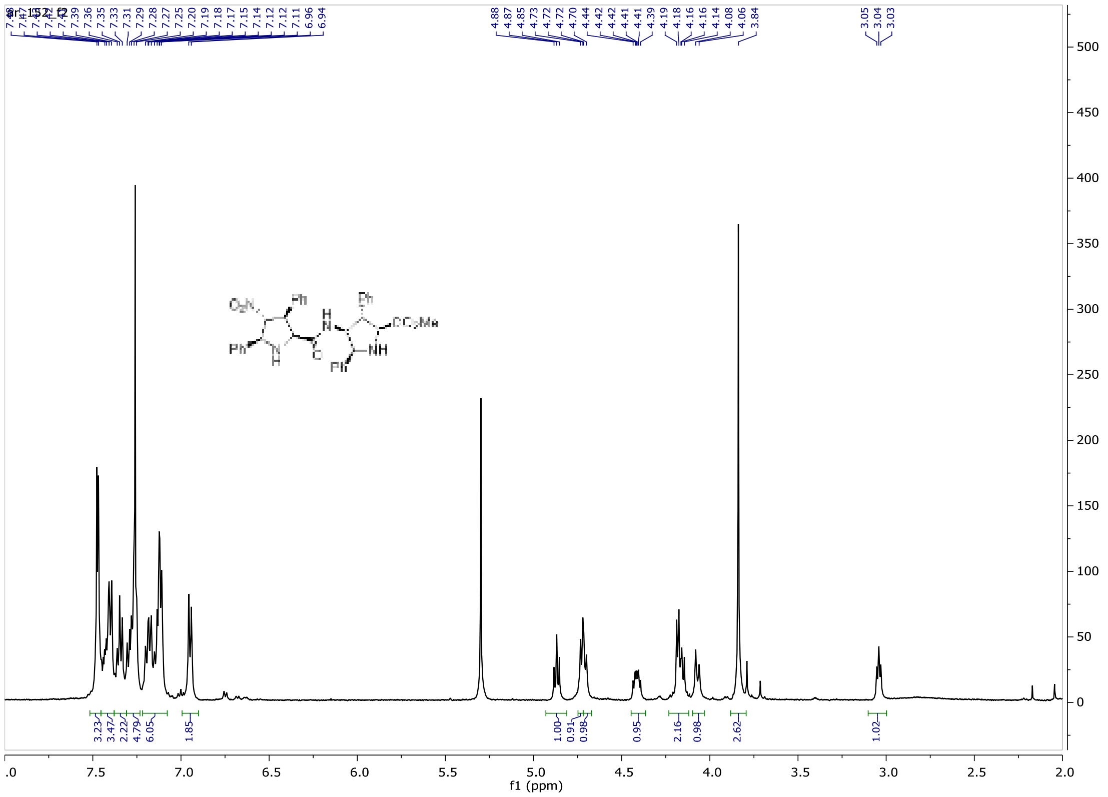

^{13}C NMR (126 MHz, $CDCl_3$)

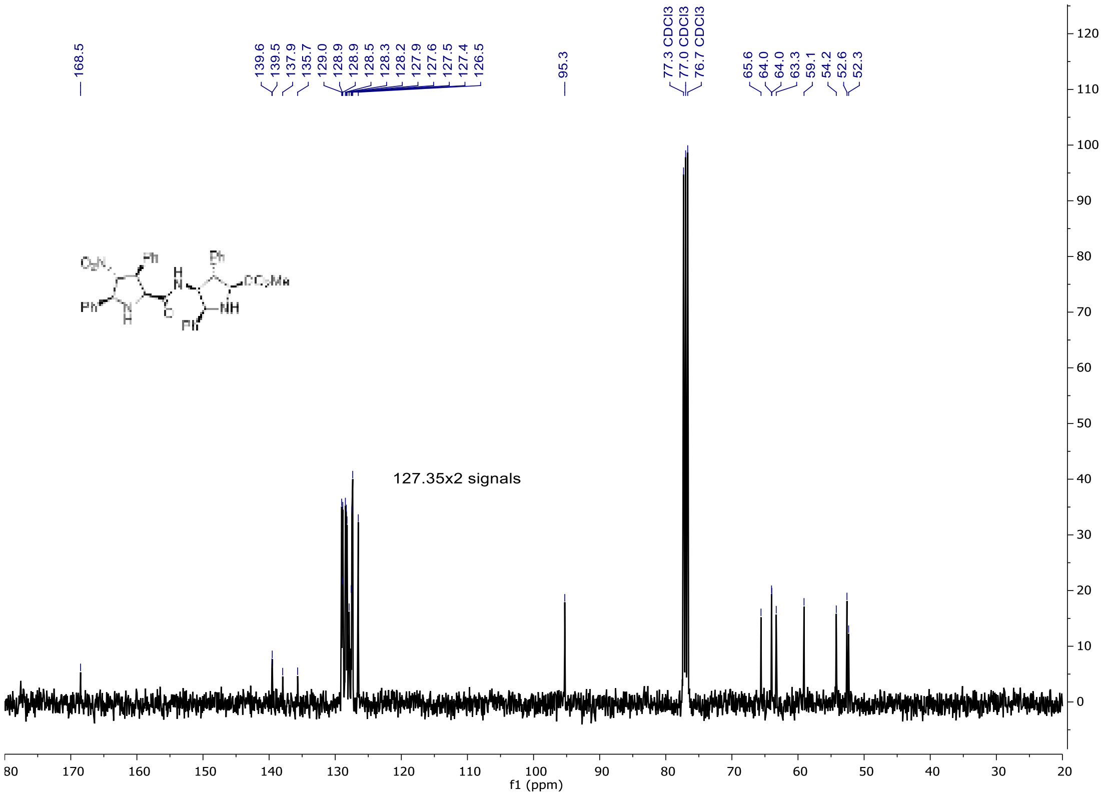

COSY ($CDCl_3$)

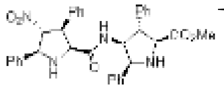

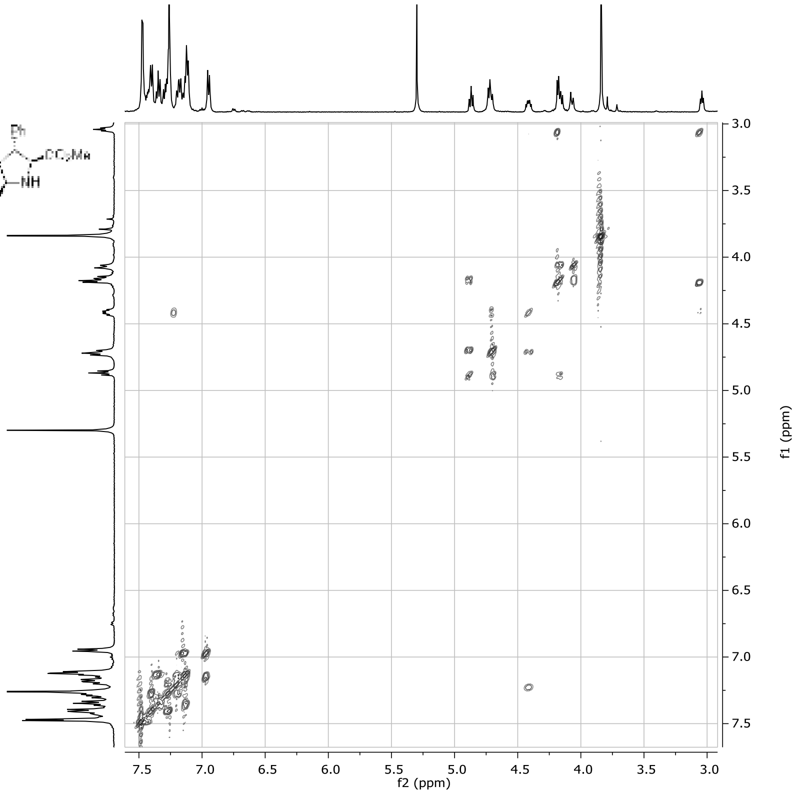

N_LX_L-**9g**

^{1}H NMR (500 MHz, $CDCl_3$)

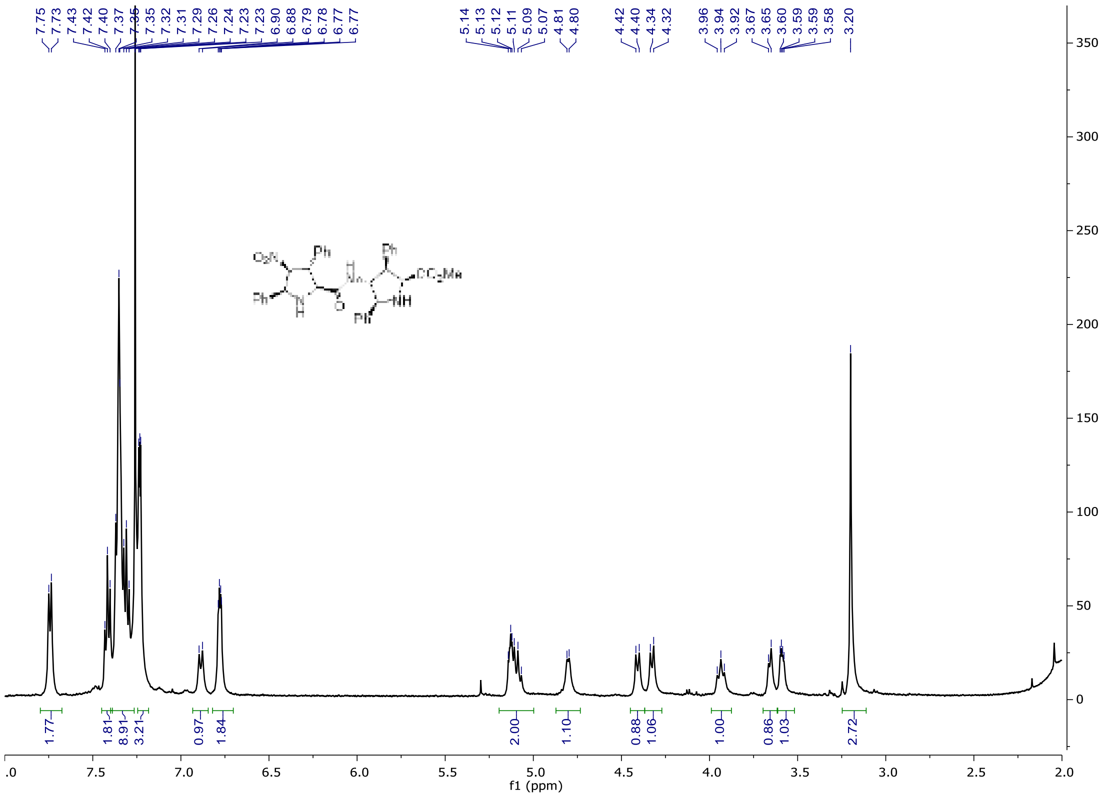

^{13}C NMR (126 MHz, $CDCl_3$)

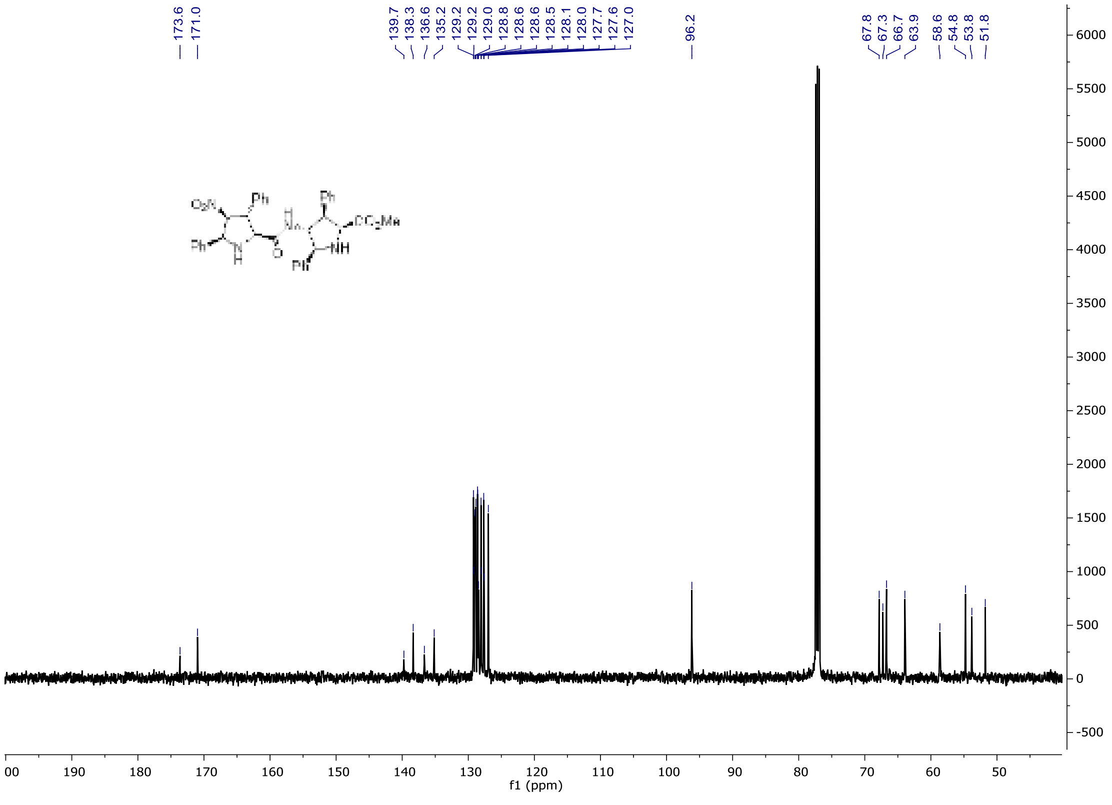

COSY ($CDCl_3$)

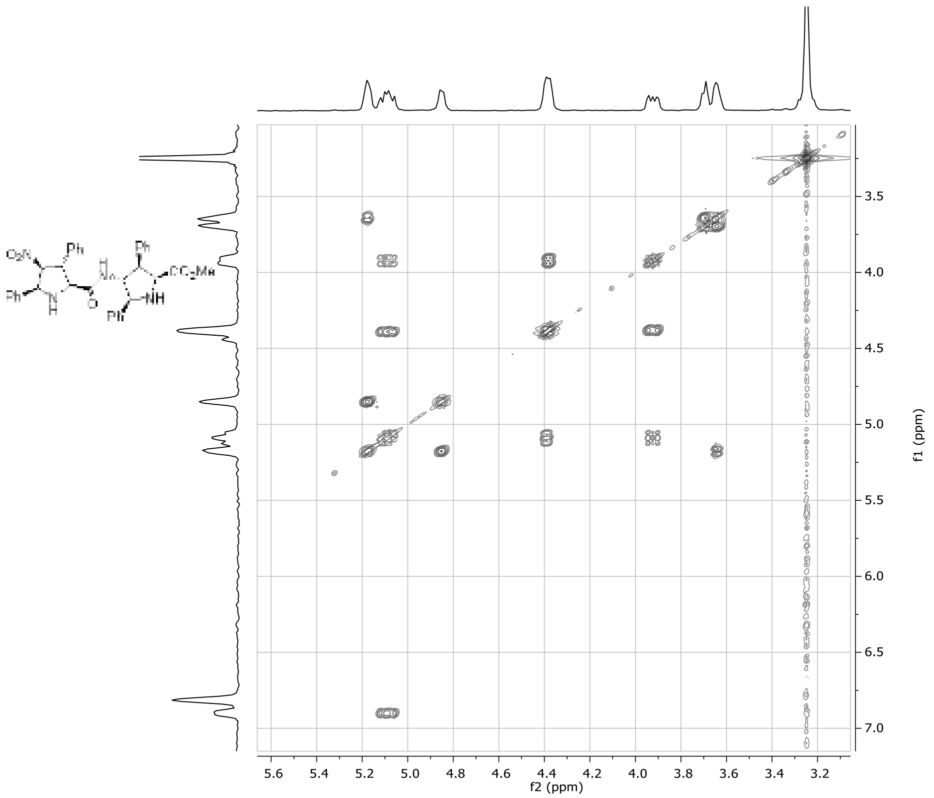

X_LN_D-**9h**

^{1}H NMR (500 MHz, $CDCl_3$)

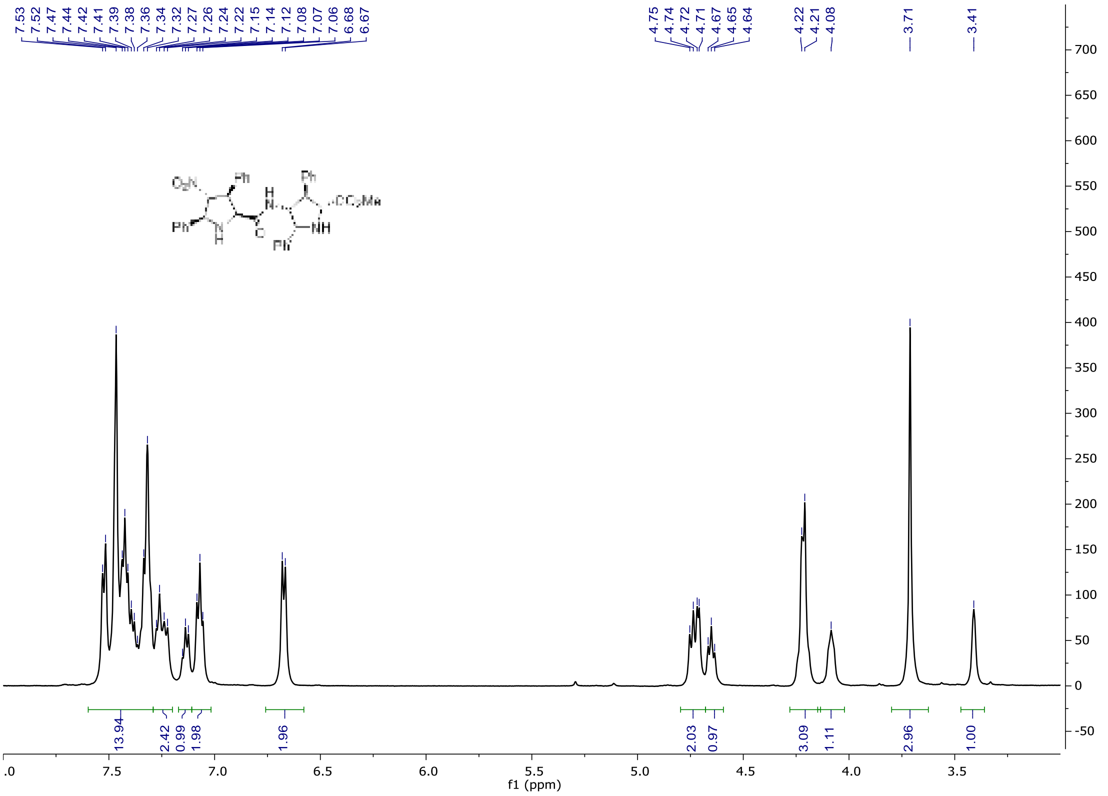

^{13}C NMR (126 MHz, $CDCl_3$)

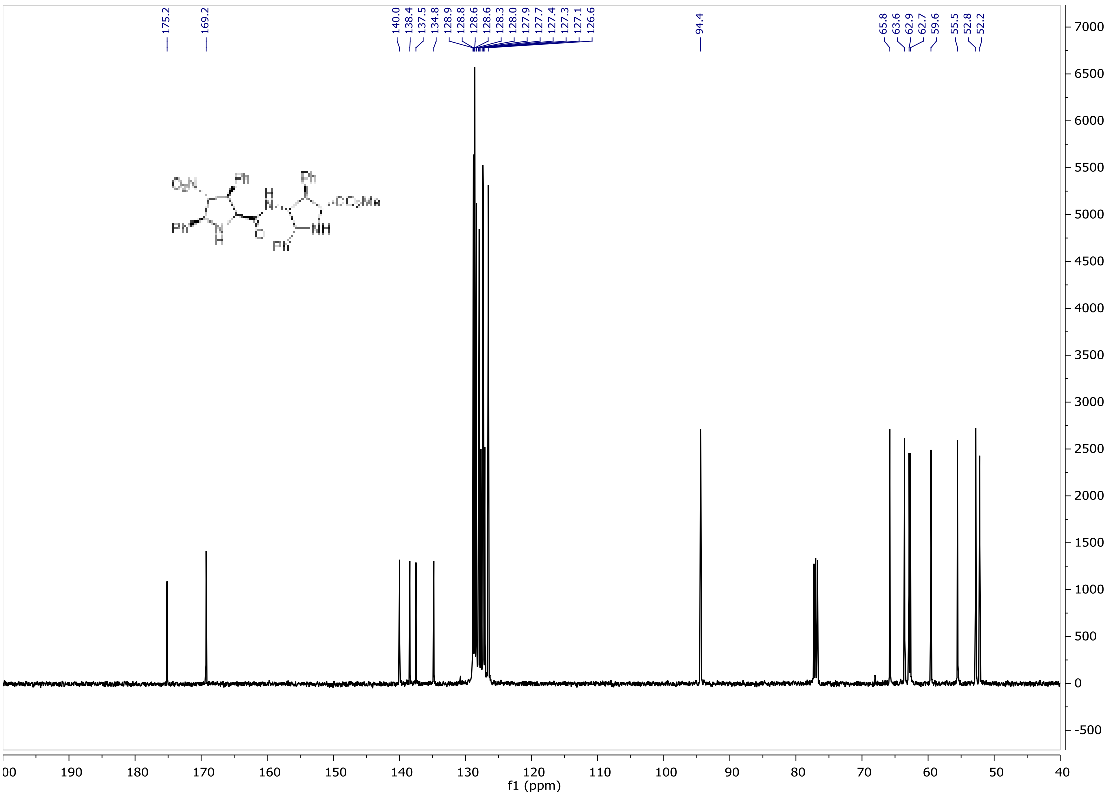

COSY ($CDCl_3$)

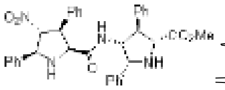

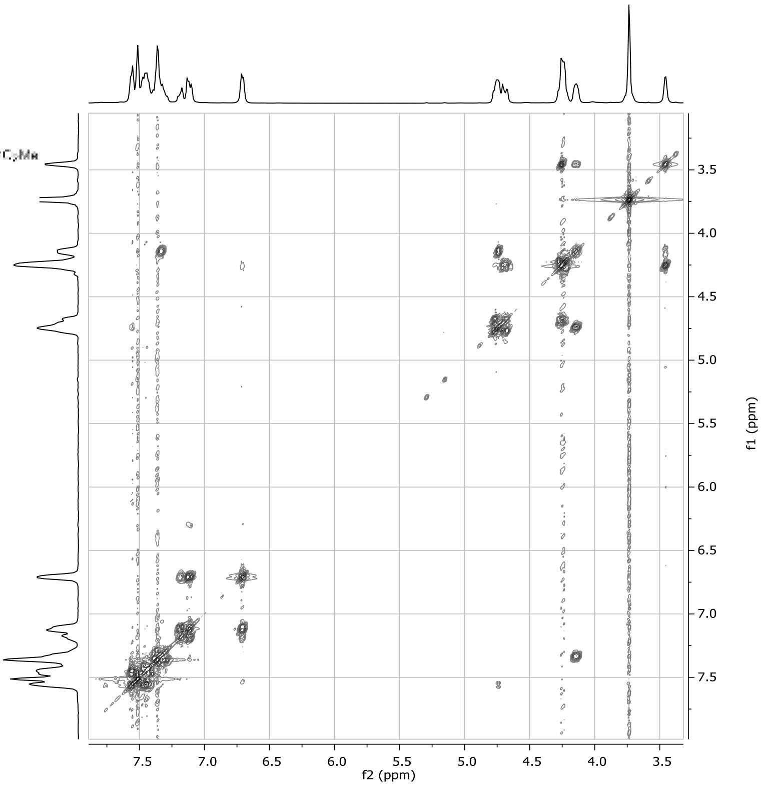

N_LX_D-**9i**

^{1}H NMR (500 MHz, $CDCl_3$)

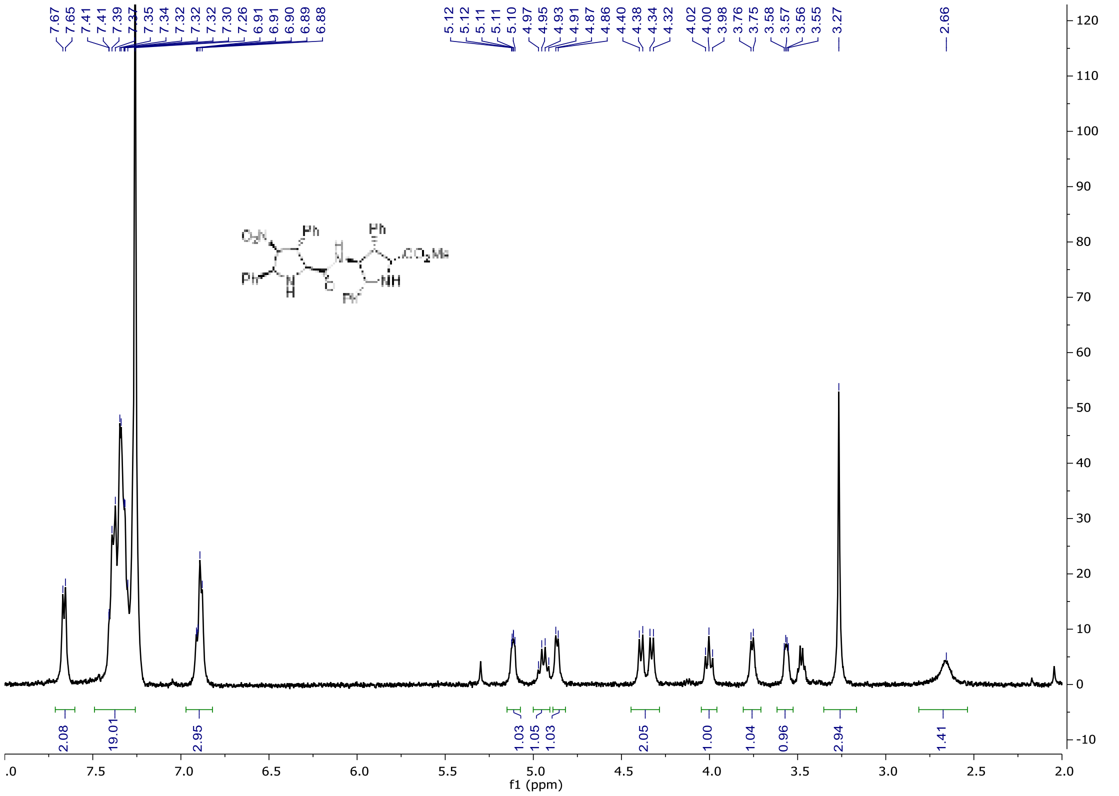

^{13}C NMR (126 MHz, $CDCl_3$)

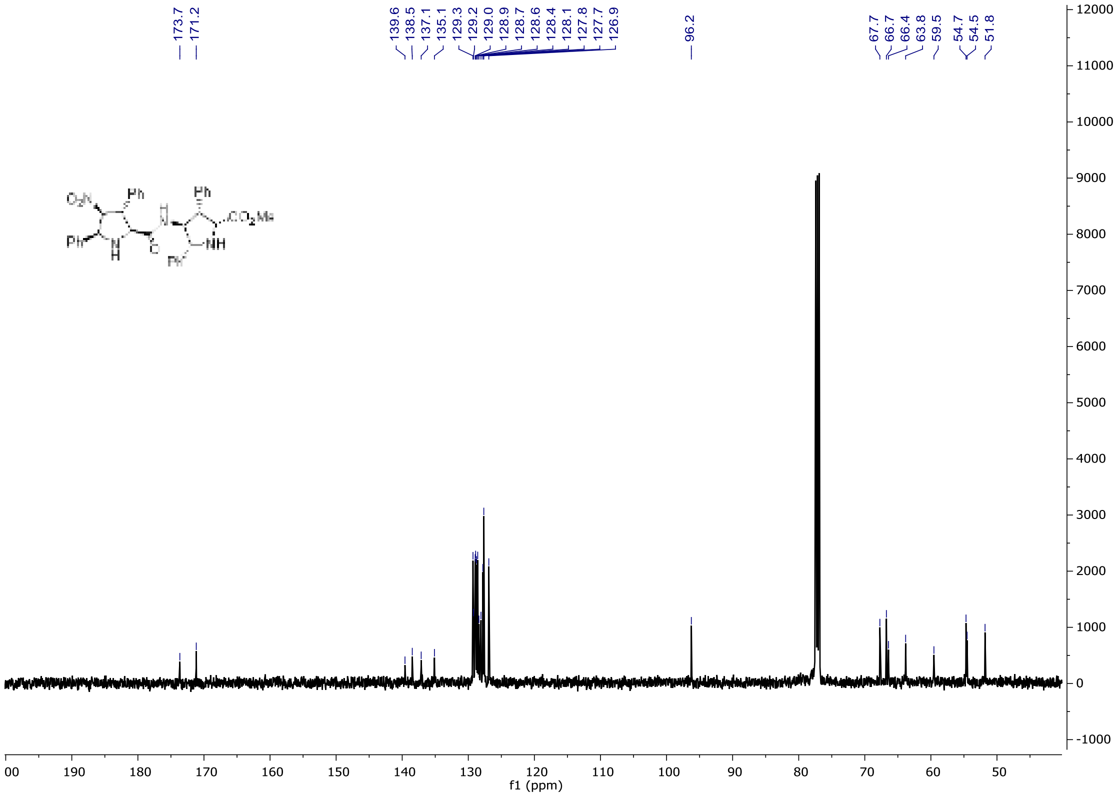

COSY ($CDCl_3$)

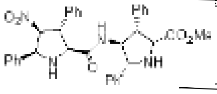

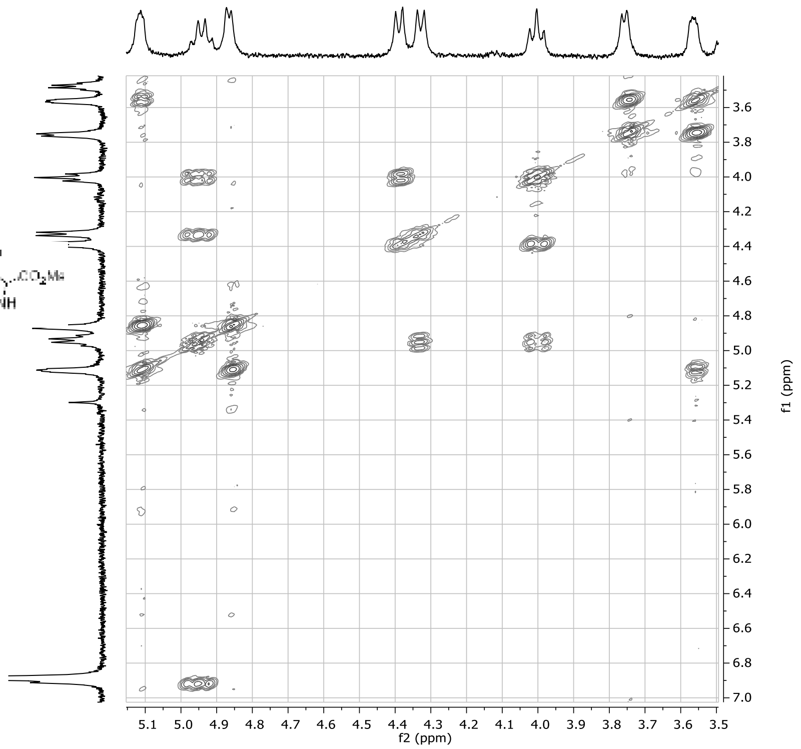

$X_LX_D^{Me}$-**9j**

^{1}H NMR (400 MHz, $CDCl_3$)

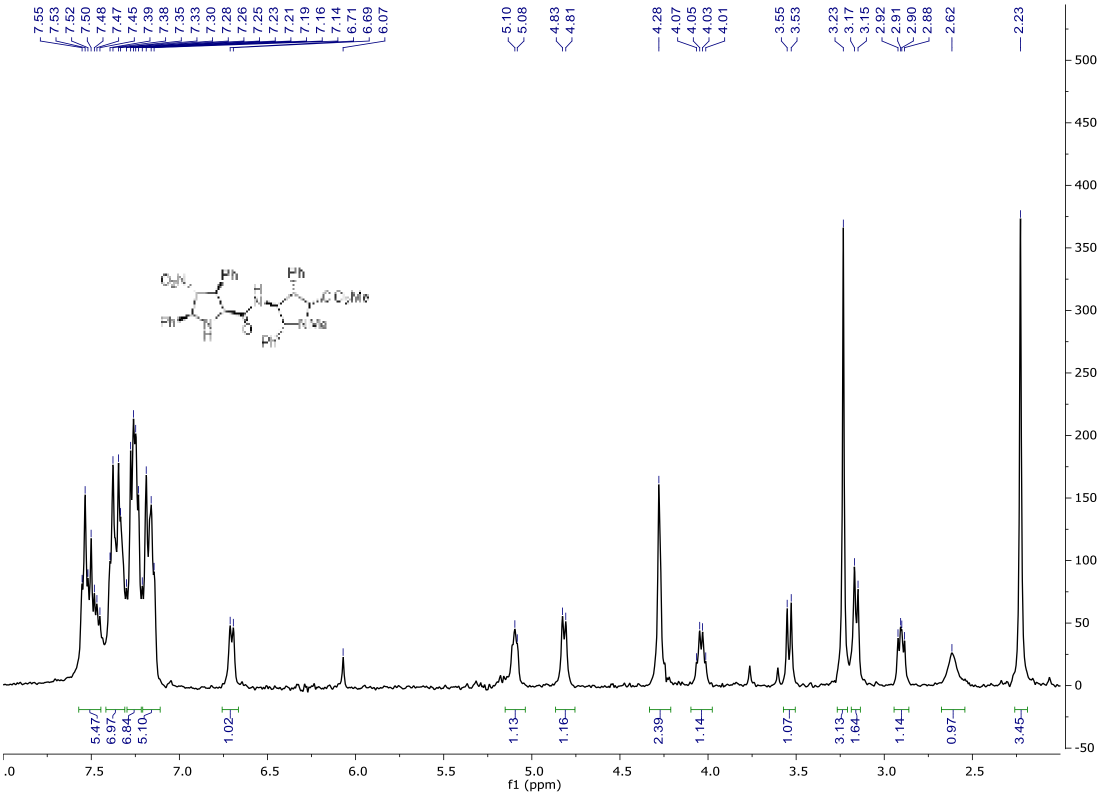

^{13}C NMR (101 MHz, $CDCl_3$)

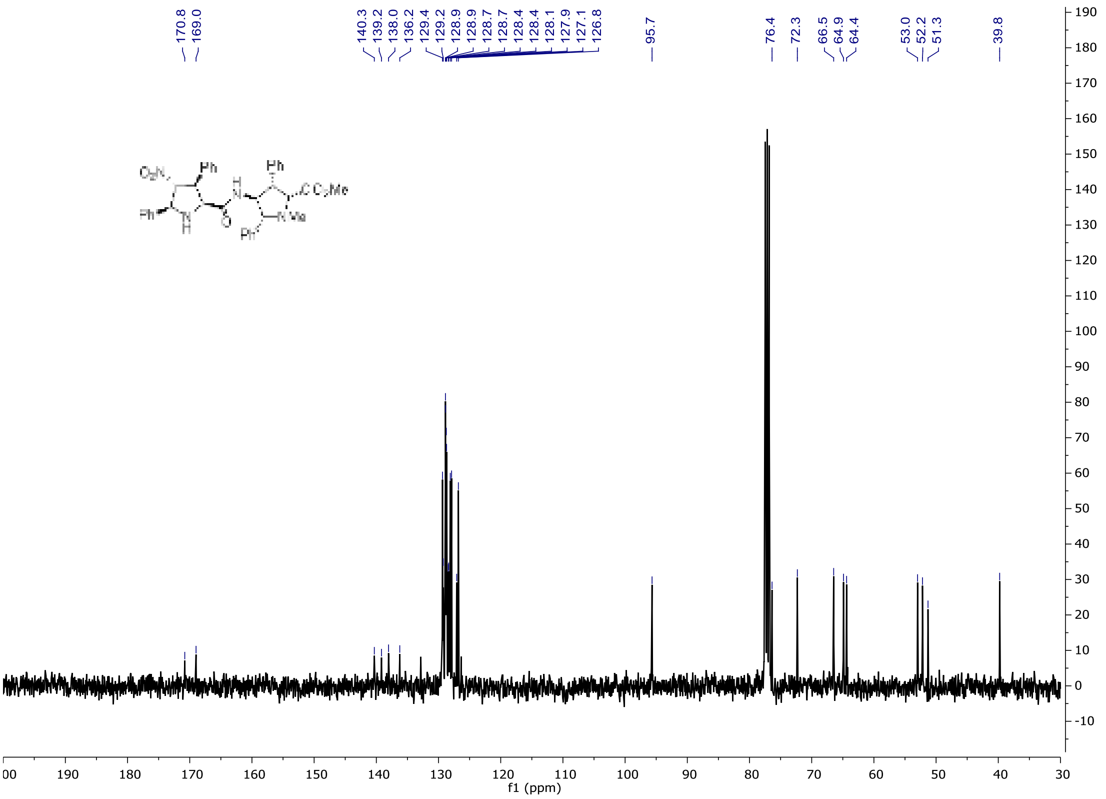

COSY ($CDCl_3$)

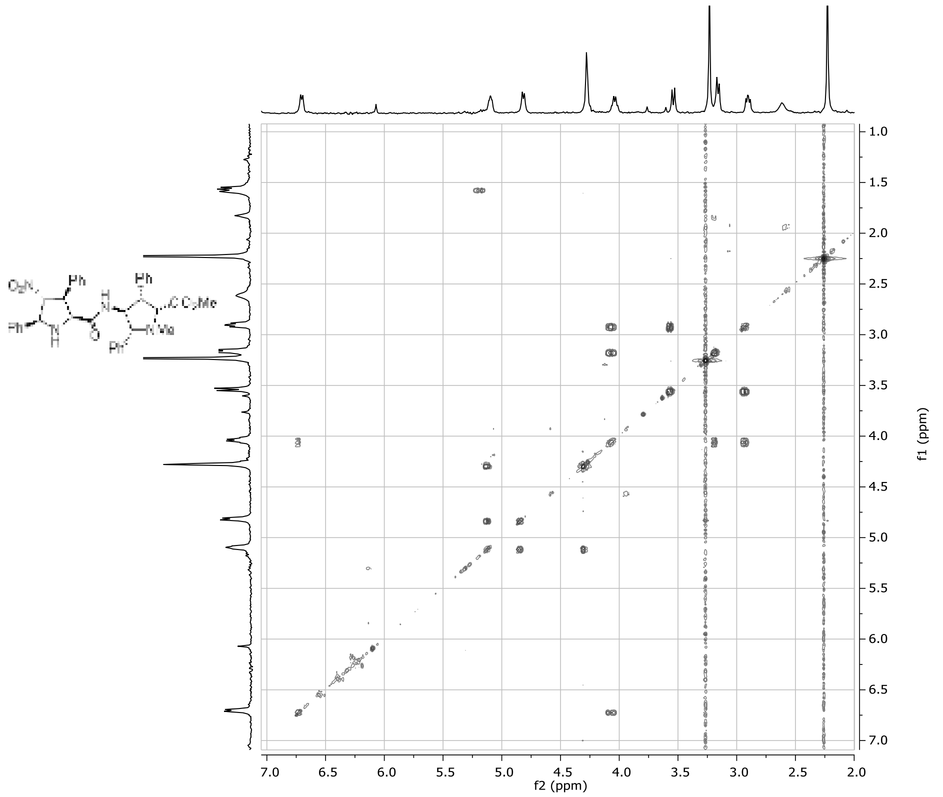

f2 (ppm)
f1 (ppm)
Ph
Ph
Ph
Ph
N
H
O
Me

$X_LX_L^{Me}$-**9k**

^{1}H NMR (400 MHz, $CDCl_3$)

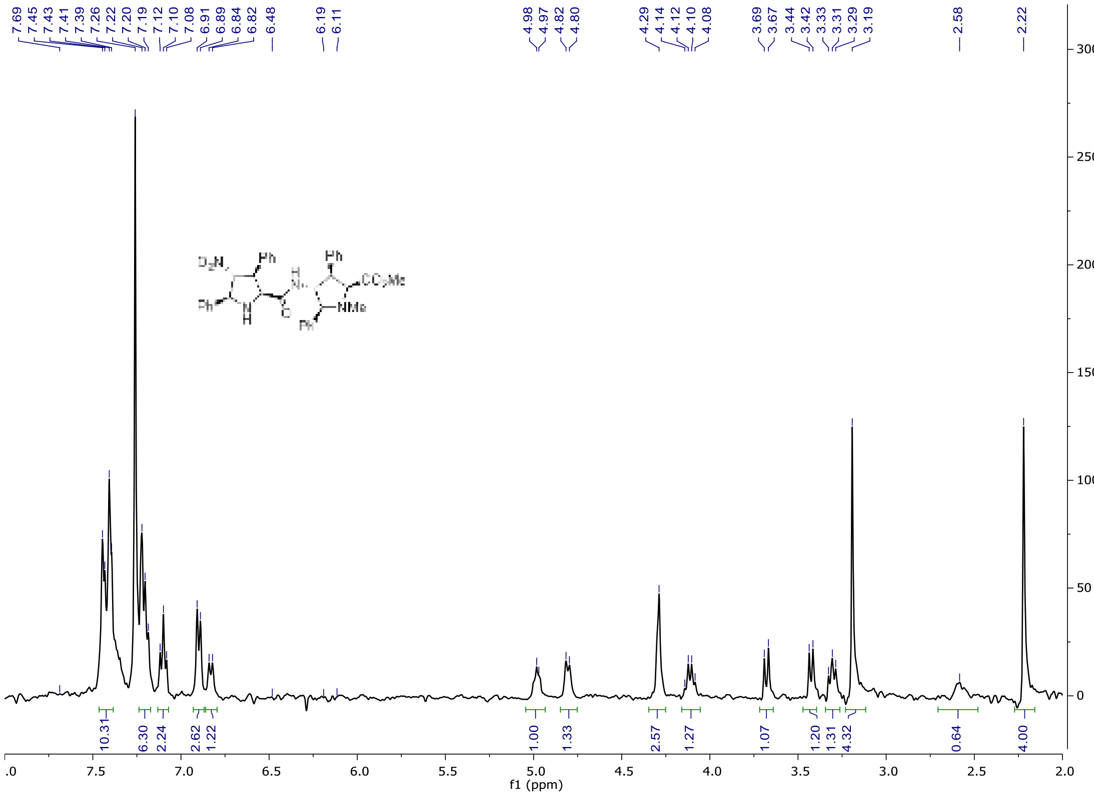

^{13}C NMR (101 MHz, $CDCl_3$)

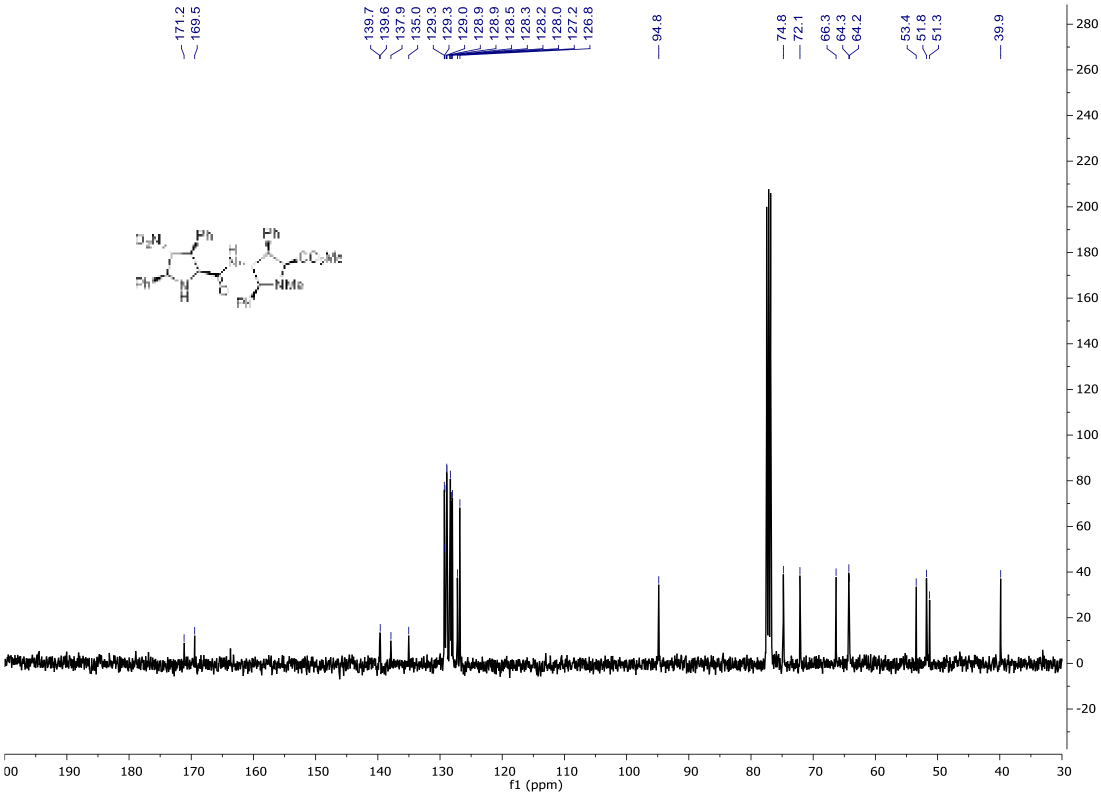

COSY ($CDCl_3$)

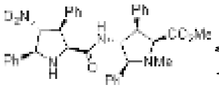

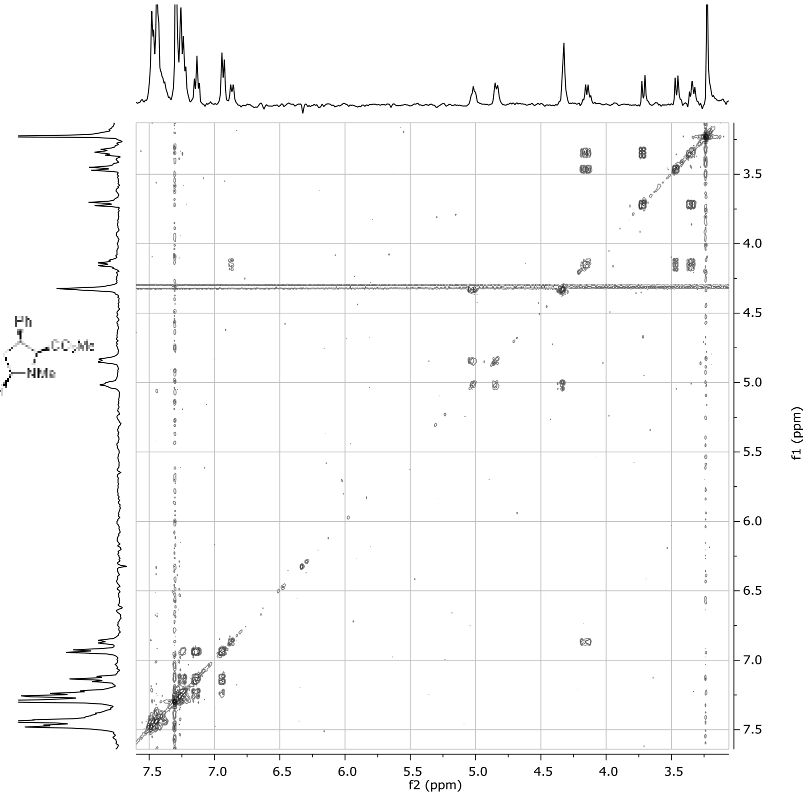

$X_L{}^{Me}X_D$-**9l**

^{1}H NMR (400 MHz, $CDCl_3$)

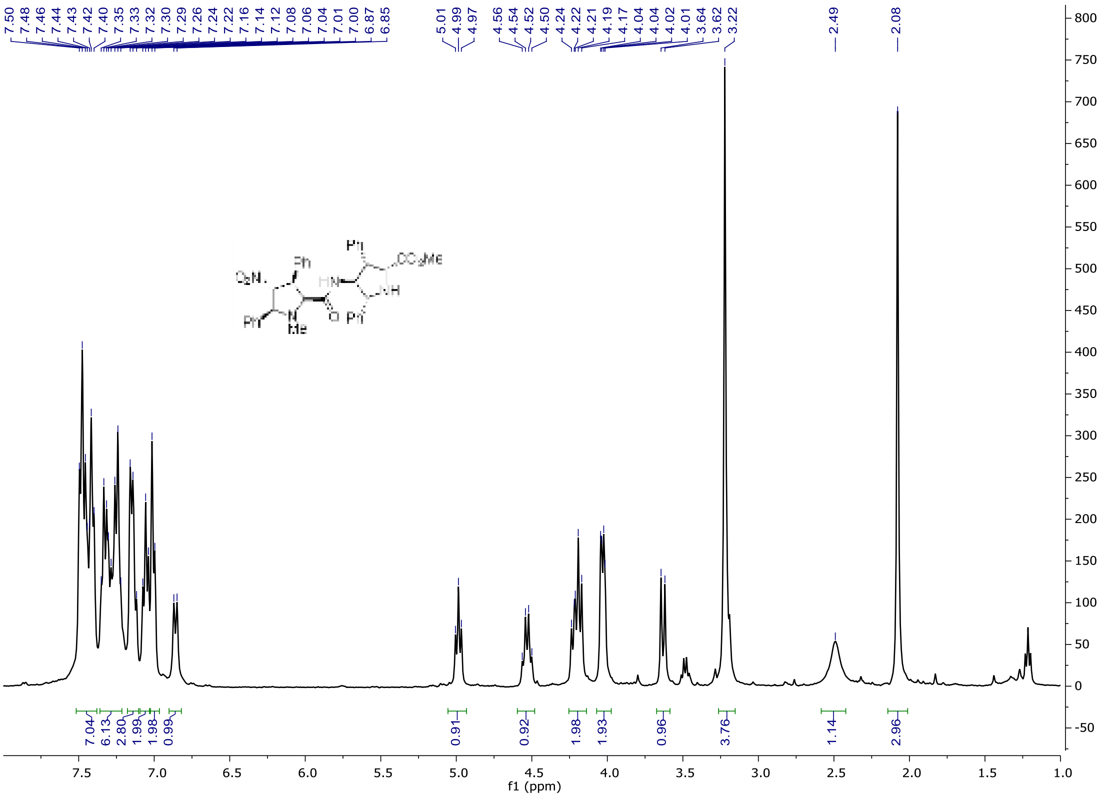

^{13}C NMR (101 MHz, $CDCl_3$)

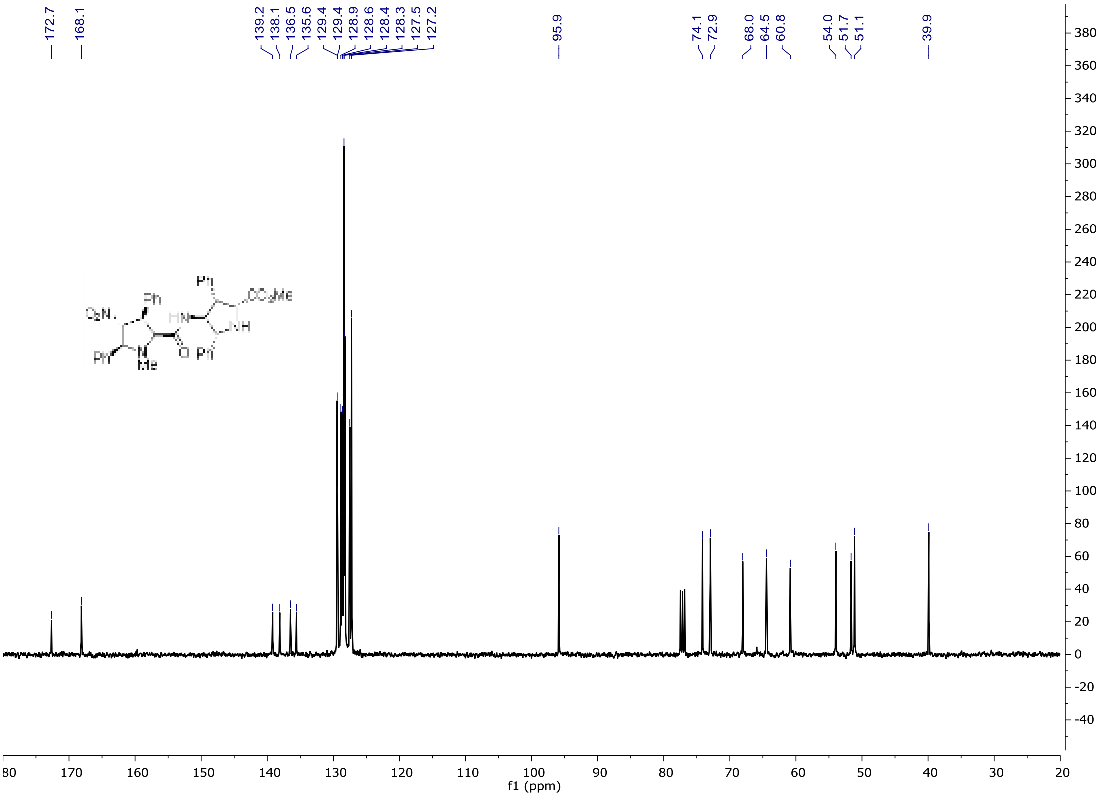

COSY ($CDCl_3$)

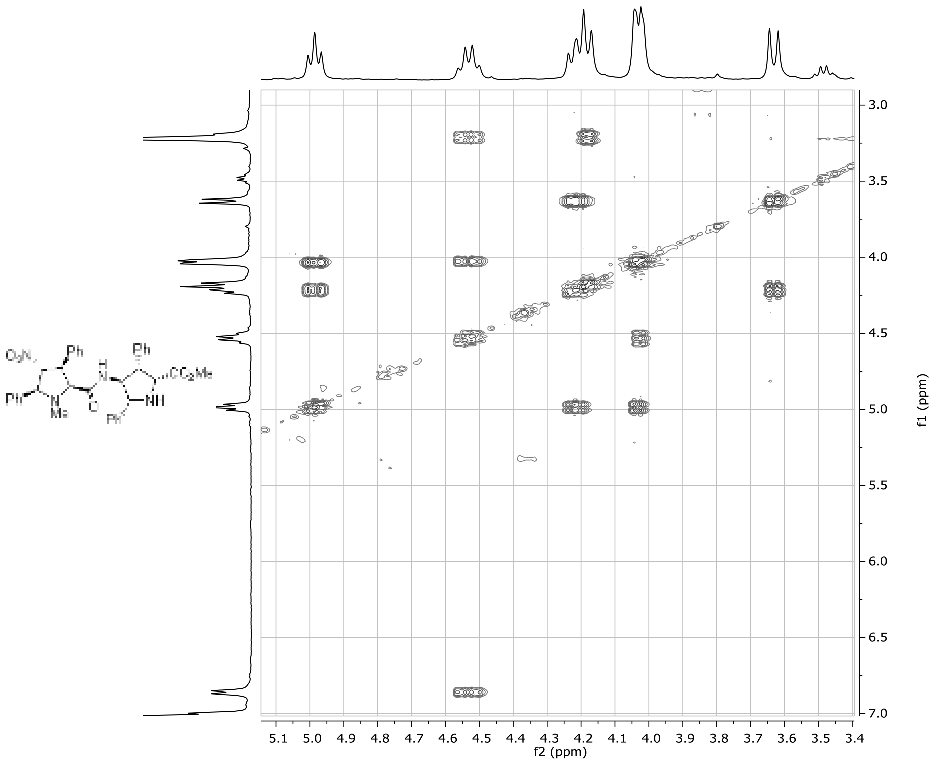

$X_L{}^{Me}X_L$-**9m**

^{1}H NMR (400 MHz, $CDCl_3$)

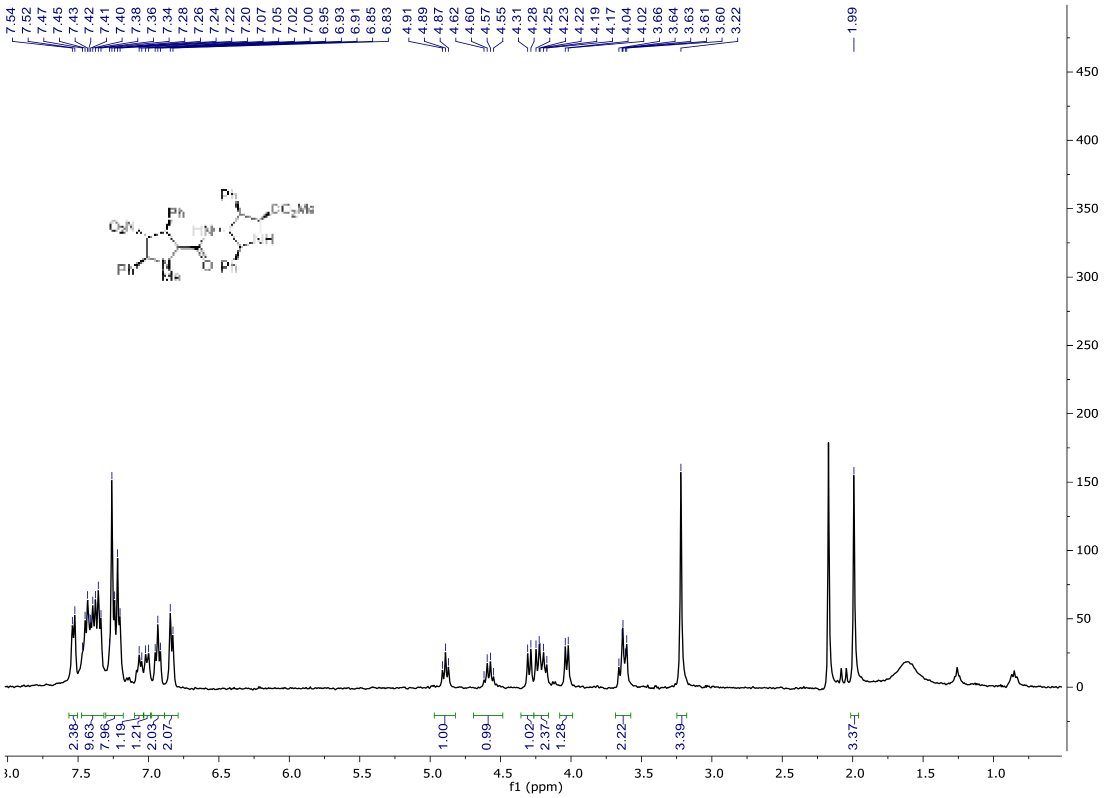

^{13}C NMR (101 MHz, $CDCl_3$)

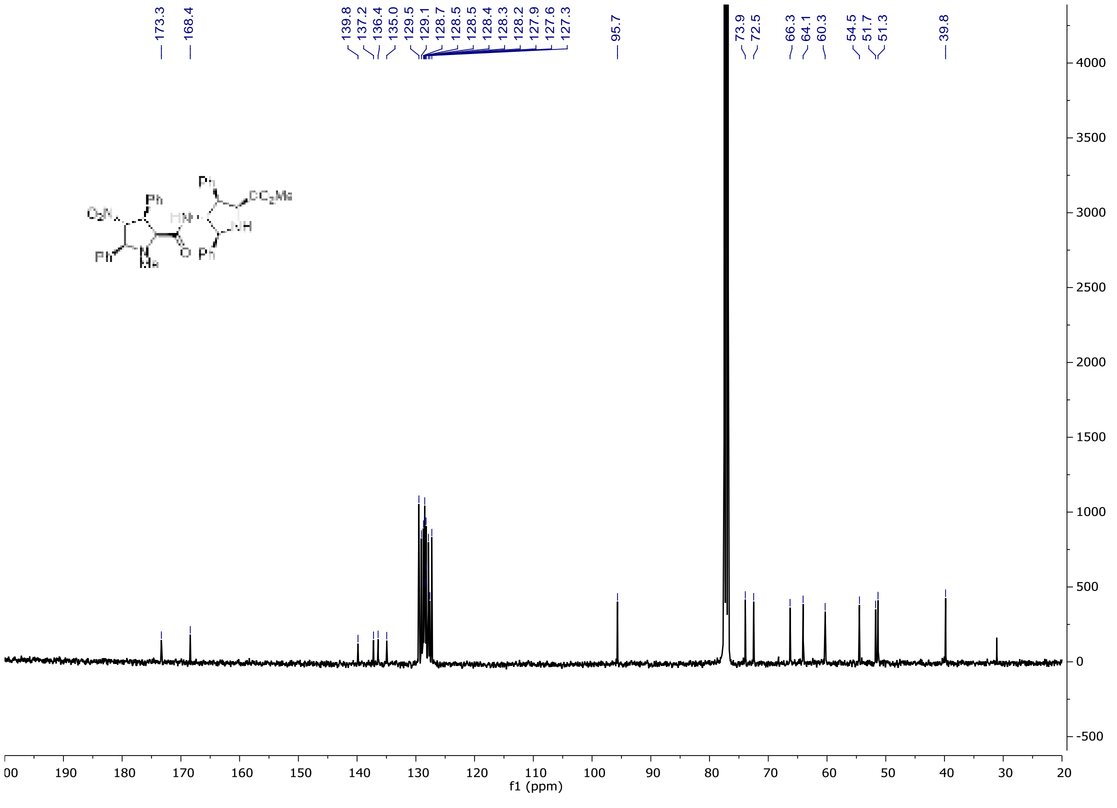

COSY ($CDCl_3$)

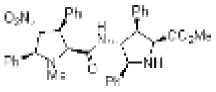

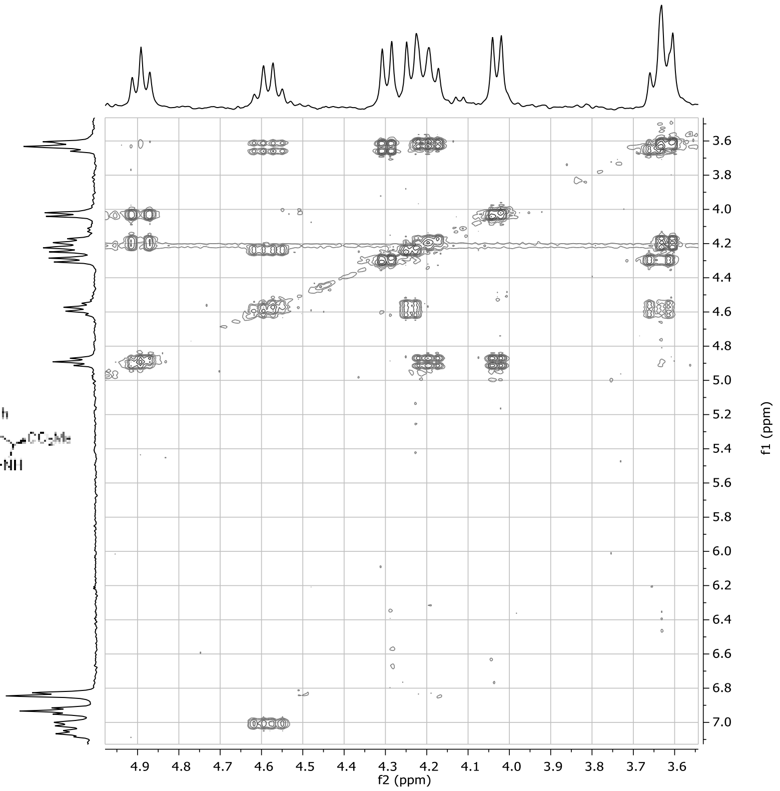

Meso-**18**

^{1}H NMR (400 MHz, $CDCl_3$)

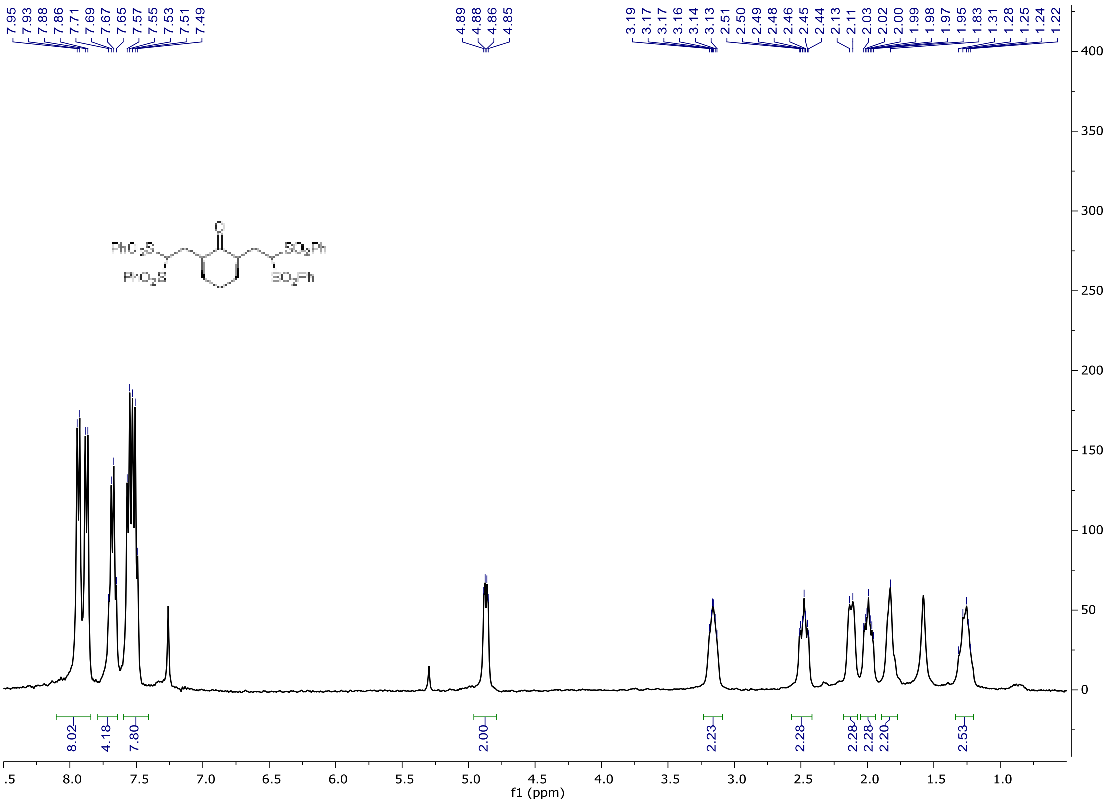

^{13}C NMR (101 MHz, $CDCl_3$)

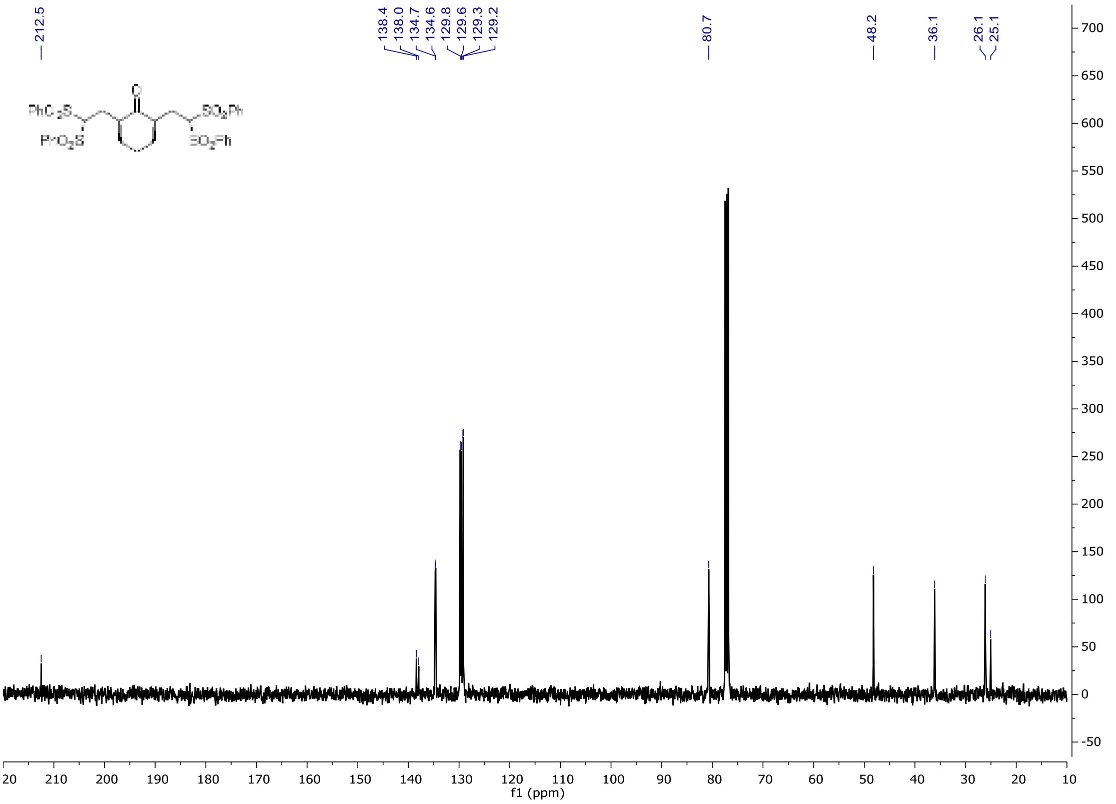

COSY ($CDCl_3$)

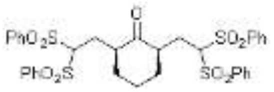

PhO2S
SO2Ph
PhO2S
SO2Ph

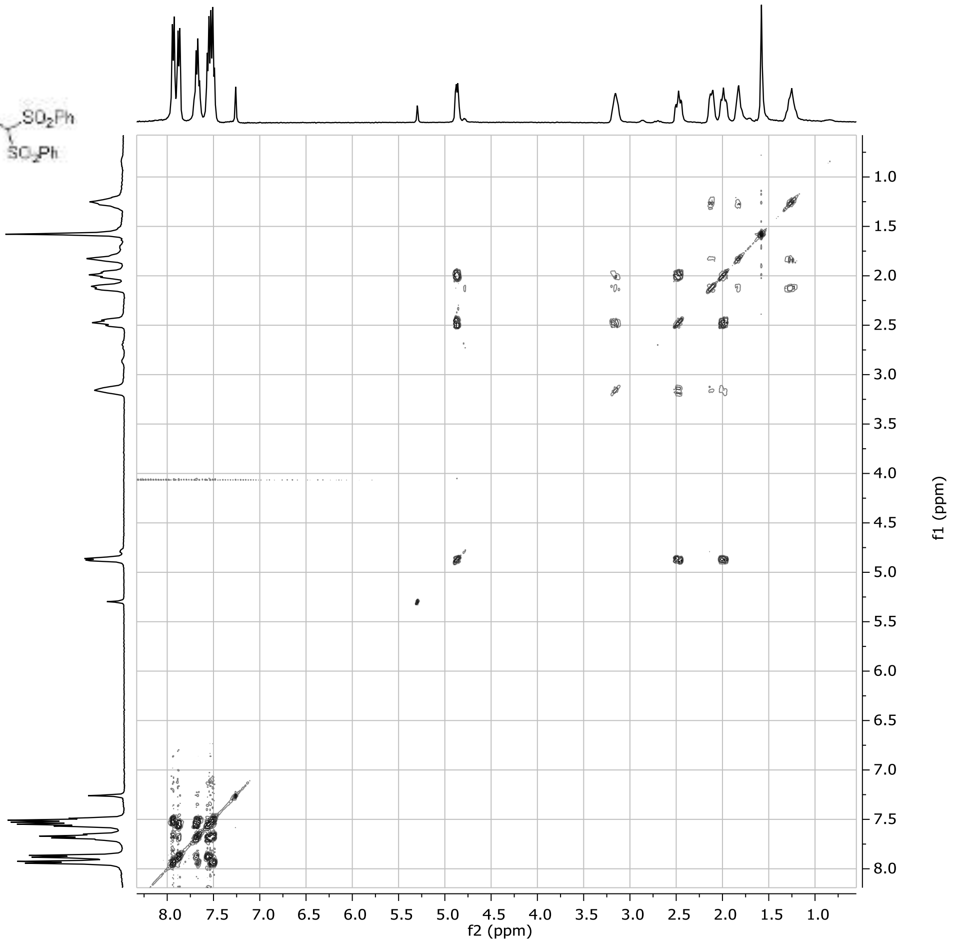

1.0
1.5
2.0
2.5
3.0
3.5
4.0
4.5
5.0
5.5
6.0
6.5
7.0
7.5
8.0
f1 (ppm)
8.0 7.5 7.0 6.5 6.0 5.5 5.0 4.5 4.0 3.5 3.0 2.5 2.0 1.5 1.0
f2 (ppm)

8 X-Ray diffraction structures

8.1 Dimer X_LX_L-9a (CCDC 2090672)

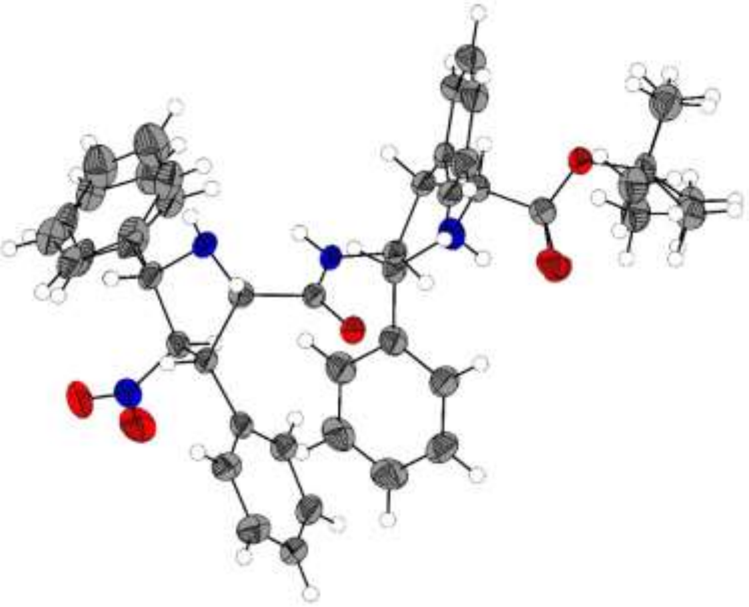

Figure S1. ORTEP drawing of X_LX_L-**9a**

8.2 Dimer $X_LX_L^{Me}$-9k (CCDC 1451833)

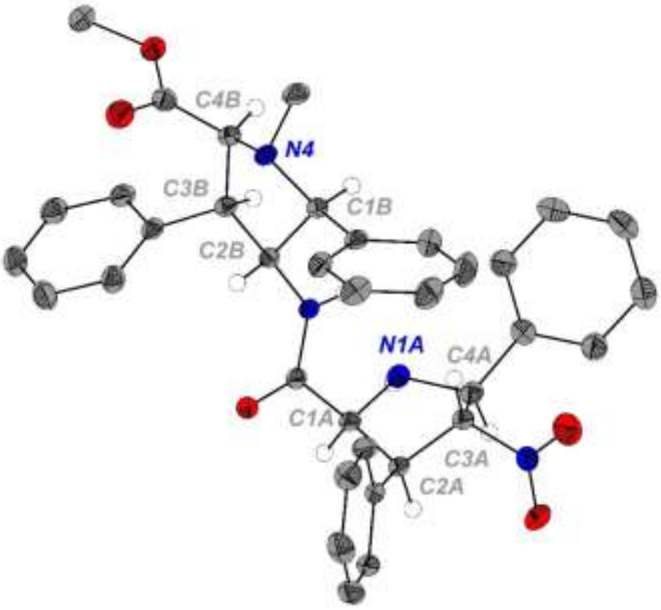

Figure S2. ORTEP drawing of $X_LX_L^{Me}$-**9k**

8.3 2,6-bis(2,2-bis(Phenylsulfonyl)ethyl)cyclohexan-1-one (meso-18) (CCDC 2090670)

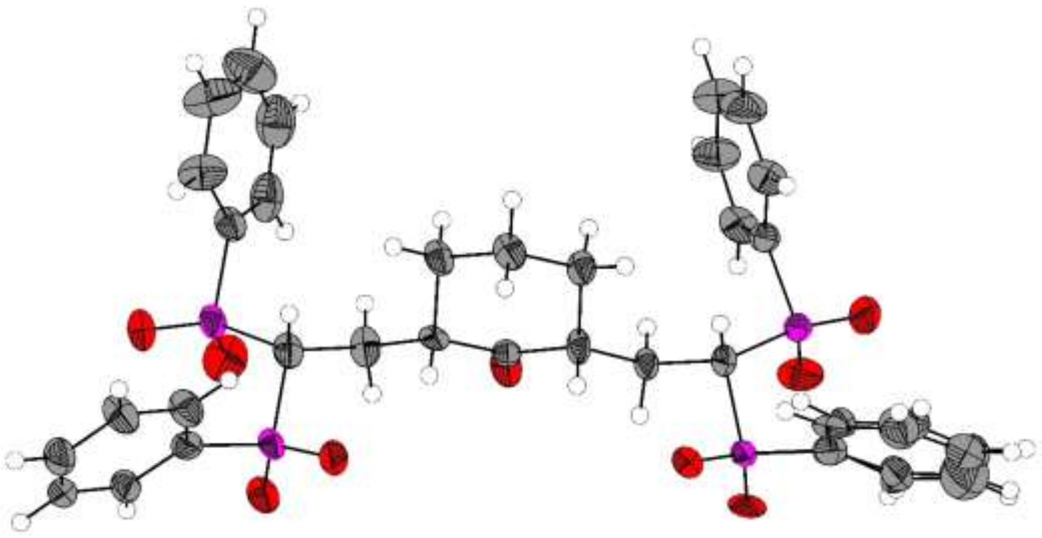

Figure S3. ORTEP drawing of meso-**18**

9 DFT calculations

9.1 Computational studies on Aldol reactions

In order to obtain a complete overview of the aldol reaction between cyclohexanone (**10**) and aldehyde **11** catalysed by **X_LX_L-9b** γ–dipeptide, we computed the energetic profile associated with the C–C bond formation step considering all the possible approaches of proximal enamine intermediates **INT1a,b** or distal enamine intermediates **INT1a',b'** to aldehyde **11** associated with catalytic cycles A and B. The results are collected in Figure S4.

The respective rate constants k_i collected in Table S1 used for the numerical simulation were estimated considering Curtin–Hammett kinetics scheme, that is it, fast interconversion between reactive intermediates and irreversible C–C bond step formation.

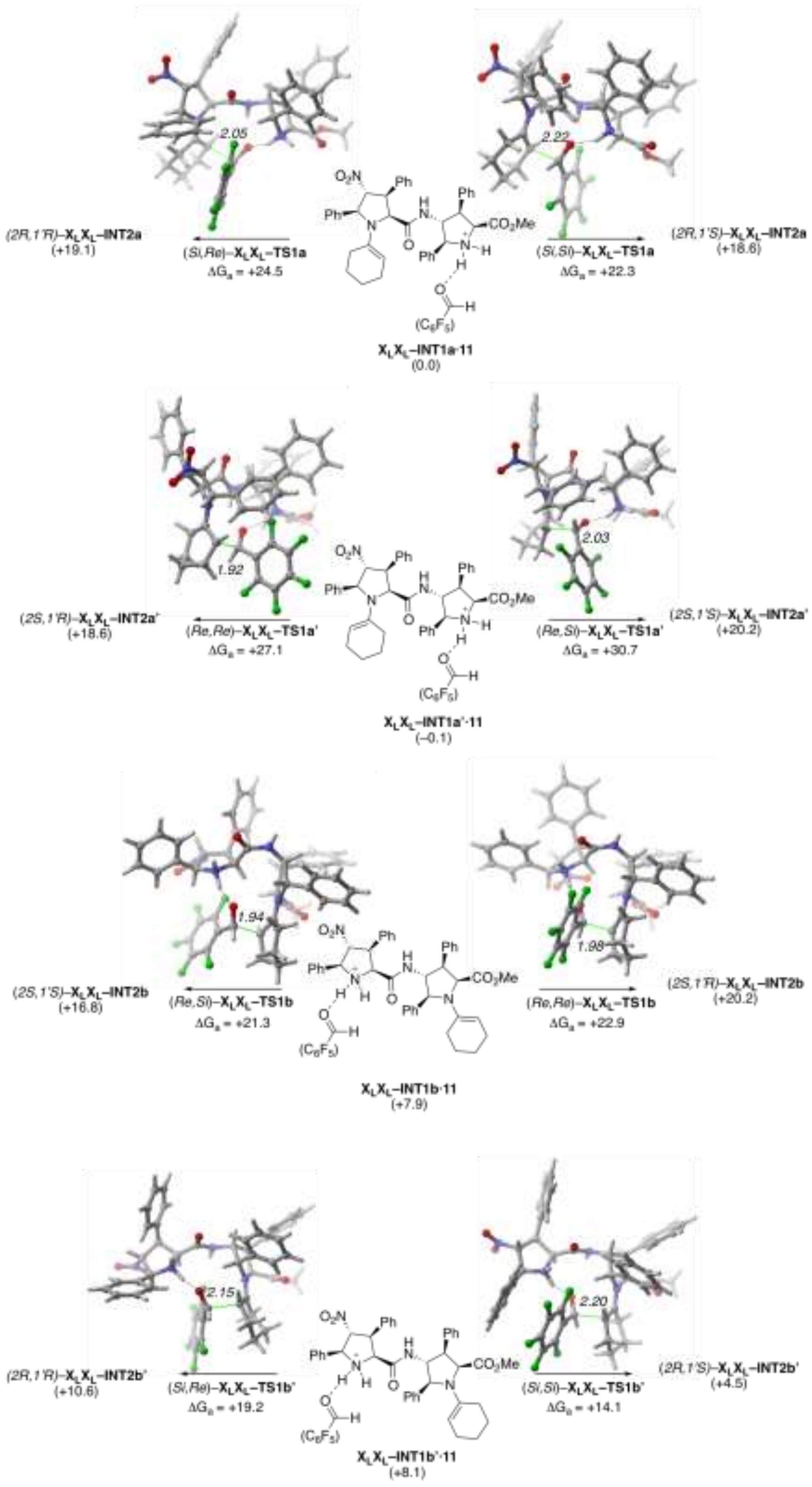

Figure S4. Main geometrical features, relative and activation Gibbs free energies (in kcal/mol) of all possible reactive complexes and transition structures associated with for the C-C bond formation step associated with the **10**+**11**→**12** Aldol reaction catalysed by **XLXL-9b** γ–dipeptide. computed at 298 K at B3LYP-GD3/6- 31G(d) level. Bond distances are given in Å.

Table S1. Calculated kinetic constants (k_i) associated with the C-C bond formation step of **10**+**11**→**12** aldol reaction catalysed by **X$_L$X$_L$-9b** γ–dipeptide.

		equilibrium between intermediates		*C–C step*
	Final product	k_i / s^{-1}	k_{-i} / s^{-1}	k_i / s^{-1}
cycle A	*(2R,1'S)*–**12**	–	–	$2.98\ 10^{-4}$
	(2R,1'R)–**12**	–	–	$7.28\ 10^{-6}$
	(2S,1'R)–**12**	$1.36\ 10^{12}$	$1.14\ 10^{12}$	$7.38\ 10^{-8}$
	(2S,1'S)–**12**	$1.36\ 10^{12}$	$1.14\ 10^{12}$	$1.94\ 10^{-10}$
cycle B	*(2R,1'S)*–**12**	$1.84\ 10^{5}$	$1.14\ 10^{12}$	$2.82\ 10^{2}$
	(2R,1'R)–**12**	$1.84\ 10^{5}$	$1.14\ 10^{12}$	$5.22\ 10^{-2}$
	(2S,1'R)–**12**	$1.35\ 10^{6}$	$1.14\ 10^{12}$	$1.01\ 10^{-4}$
	(2S,1'S)–**12**	$1.35\ 10^{6}$	$1.14\ 10^{12}$	$1.50\ 10^{-3}$

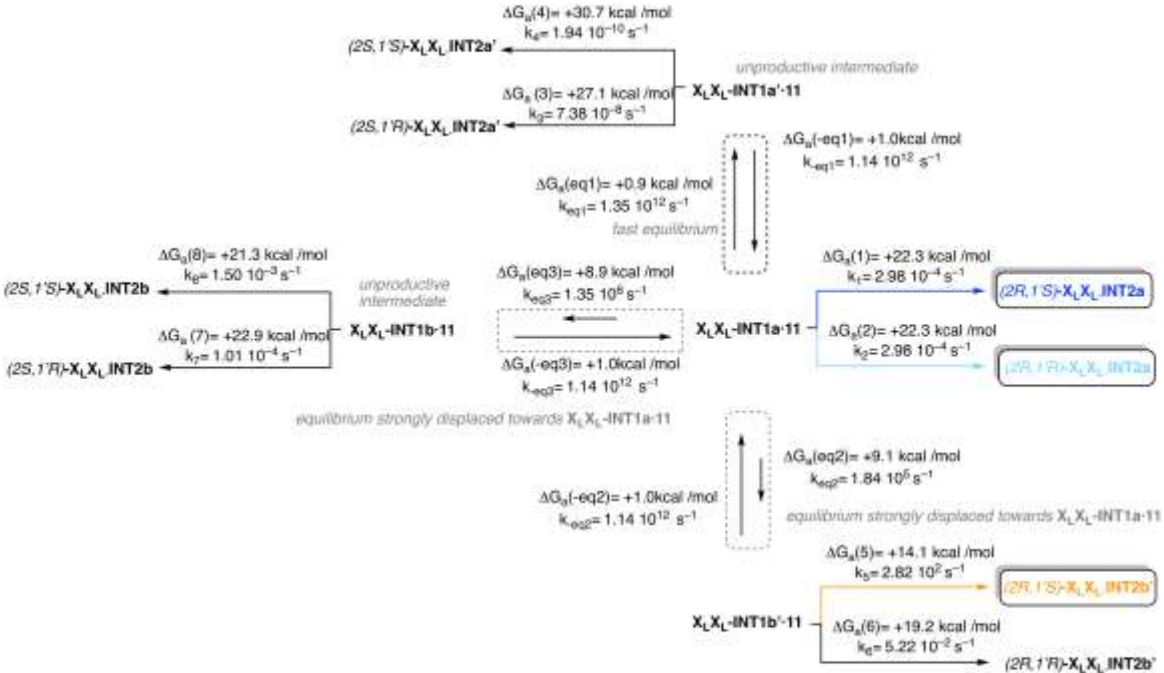

Scheme S1. Representation of the data used in the simulations for **10**+**11**→**12** aldol reaction catalysed by **X$_L$X$_L$-9b** γ–dipeptide.

Kinetic equations associated with **10**+**11**→**12** aldol reaction catalysed by **X$_L$X$_L$-9b** γ–dipeptide.

$$-\frac{\left[X_LX_L - INT1a{\cdot}11\right]}{dt} = +k_1\left[X_LX_L - INT1a{\cdot}11\right] + k_2\left[X_LX_L - INT1a{\cdot}11\right]$$
$$-k_{-eq1}\left[X_LX_L - INT1a'{\cdot}11\right] - k_{-eq2}\left[X_LX_L - INT1b{\cdot}11\right] - k_{-eq3}\left[X_LX_L - INT1b'{\cdot}11\right]$$
$$+k_{eq1}\left[X_LX_L - INT1a{\cdot}11\right] + k_{eq2}\left[X_LX_L - INT1a11\right] + k_{eq3}\left[X_LX_L - INT1a{\cdot}11\right] \quad \text{(eq S1)}$$

$$\frac{\left[(2R,1'S)-INT\,2a(anti\ -12\ fromINT\,1a)\right]}{dt}=+k_1\left[X_L X_L-INT\,1a\cdot 11\right] \qquad \text{(eq S2)}$$

$$\frac{\left[(2R,1'R)-INT\,2a(syn\ -12\ fromINT\,1a)\right]}{dt}=+k_2\left[X_L X_L-INT\,1a\cdot 11\right] \qquad \text{(eq S3)}$$

$$-\frac{\left[X_L X_L-INT\,1b'\cdot 11\right]}{dt}=+k_5\left[X_L X_L-INT\,1b'\cdot 11\right]+k_6\left[X_L X_L-INT\,1b'\cdot 11\right]$$
$$+k_{-eq2}\left[X_L X_L-INT\,1b'\cdot 11\right]-k_{eq2}\left[X_L X_L-INT\,1a\cdot 11\right] \qquad \text{(eq S4)}$$

$$\frac{\left[(2R,1'S)-INT\,2b'(anti\ -12\ fromINT\,1b')\right]}{dt}=+k_5\left[X_L X_L-INTb'\cdot 11\right] \qquad \text{(eq S5)}$$

$$\frac{\left[(2R,1'R)-INT\,2b'(syn\ -12\ fromINT\,1b')\right]}{dt}=+k_6\left[X_L X_L-INT\,1b'\cdot 11\right] \qquad \text{(eq S6)}$$

$$-\frac{\left[X_L X_L-INT\,1a'\cdot 11\right]}{dt}=+k_3\left[X_L X_L-INT\,1a'\cdot 11\right]+k_4\left[X_L X_L-INT\,1a'\cdot 11\right]$$
$$+k_{-eq1}\left[X_L X_L-INT\,1a'\cdot 11\right]-k_{eq1}\left[X_L X_L-INT\,1a\cdot 11\right] \qquad \text{(eq S7)}$$

$$\frac{\left[(2S,1'S)-INT\,2a(syn\ -12\ fromINT\,1a')\right]}{dt}=+k_4\left[X_L X_L-INTa'\cdot 11\right] \qquad \text{(eq S8)}$$

$$\frac{\left[(2S,1'R)-INT\,2a(anti\ -12\ fromINT\,1a')\right]}{dt}=+k_3\left[X_L X_L-INT\,1a'\cdot 11\right] \qquad \text{(eq S9)}$$

$$-\frac{\left[X_L X_L-INT\,1b\cdot 11\right]}{dt}=+k_7\left[X_L X_L-INT\,1b\cdot 11\right]+k_8\left[X_L X_L-INT\,1b\cdot 11\right]$$
$$+k_{-eq3}\left[X_L X_L-INT\,1b\cdot 11\right]-k_{eq3}\left[X_L X_L-INT\,1a\cdot 11\right] \qquad \text{(eq S10)}$$

$$\frac{\left[(2S,1'S)-INT\,2a(syn\ -12\ fromINTb)\right]}{dt}=+k_8\left[X_L X_L-INTb\cdot 11\right] \qquad \text{(eq S11)}$$

$$\frac{\left[(2S,1'R)-INT\,2a(anti\ -12\ fromINT\,1b)\right]}{dt}=+k_7\left[X_L X_L-INT\,1b\cdot 11\right] \qquad \text{(eq S12)}$$

9.2 Computational studies on Michael reactions

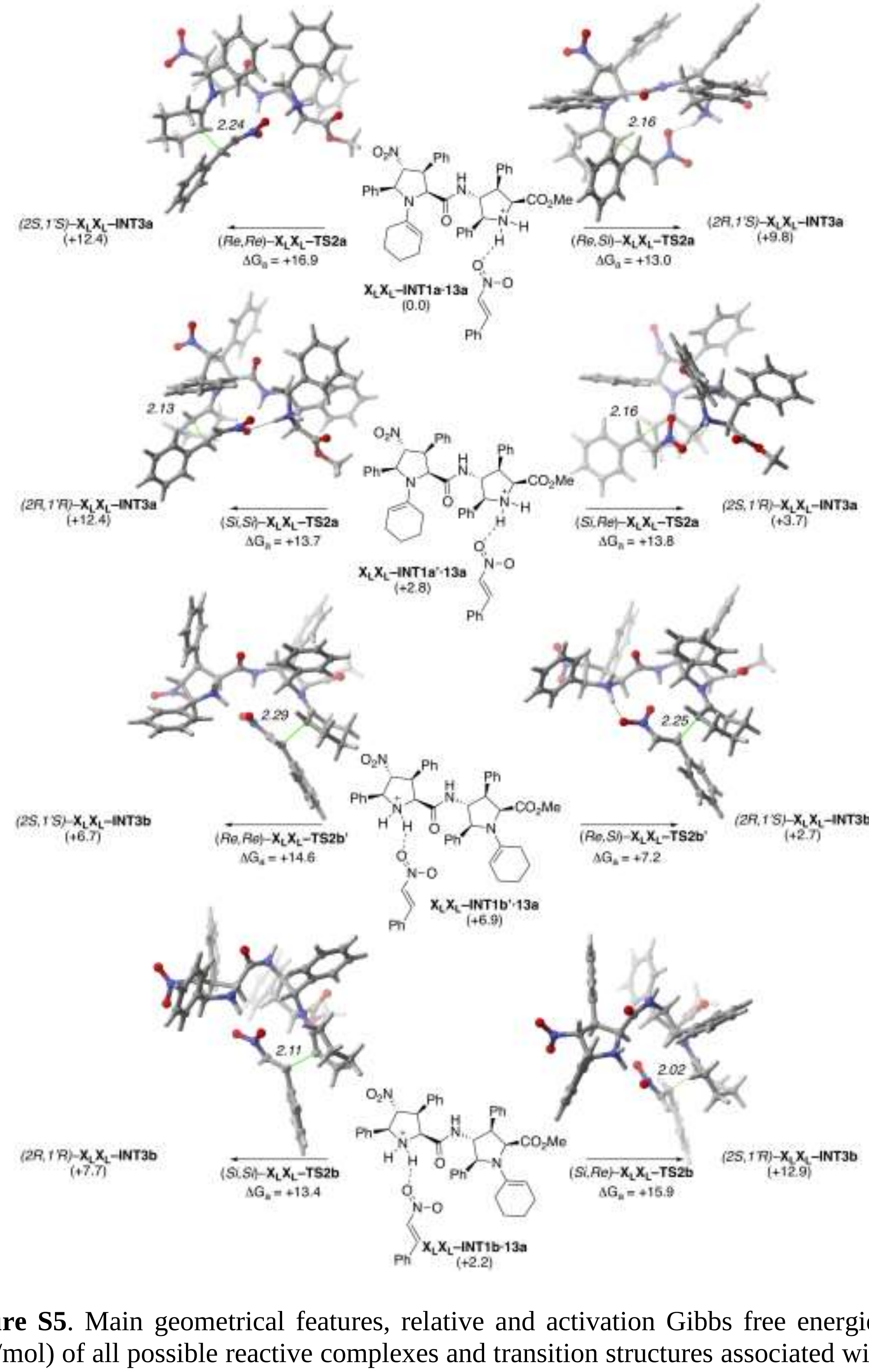

Figure S5. Main geometrical features, relative and activation Gibbs free energies (in kcal/mol) of all possible reactive complexes and transition structures associated with for the C-C bond formation step associated with the **10**+**13a**→**14** Michael reaction catalysed by **XLXL-9b** γ–dipeptide. computed at 298 K at B3LYP-GD3/6- 31G(d) level. Bond distances are given in Å.

Table S2. Calculated kinetic constants (k_i) associated with the C-C bond formation step of **10**+**13a**→**14** Michael reaction catalysed by **X$_L$X$_L$-9b** γ–dipeptide.

		equilibrium between intermediates		*C–C step*
	Final product	k_i / s^{-1}	k_{-i} / s^{-1}	k_i / s^{-1}
cycle A	*(2R,1'S)*–**X$_L$X$_L$–INT3a**	–	–	1.54 10^3
	(2S,1'S)–**X$_L$X$_L$–INT3a**	–	–	2.53 10^0
	(2S,1'R)–**X$_L$X$_L$–INT3a**	1.02 10^{10}	1.14 10^{12}	4.75 10^2
	(2R,1'R)–**X$_L$X$_L$–INT3a**	1.02 10^{10}	1.14 10^{12}	5.62 10^2
cycle B	*(2R,1'S)*–**X$_L$X$_L$–INT3b**	1.00 10^7	1.14 10^{12}	3.27 10^7
	(2S,1'S)–**X$_L$X$_L$–INT3b**	1.00 10^7	1.14 10^{12}	1.23 10^2
	(2S,1'R)–**X$_L$X$_L$–INT3b**	2.80 10^{10}	1.14 10^{12}	1.37 10^1
	(2R,1'R)–**X$_L$X$_L$–INT3b**	2.80 10^{10}	1.14 10^{12}	9.33 10^2

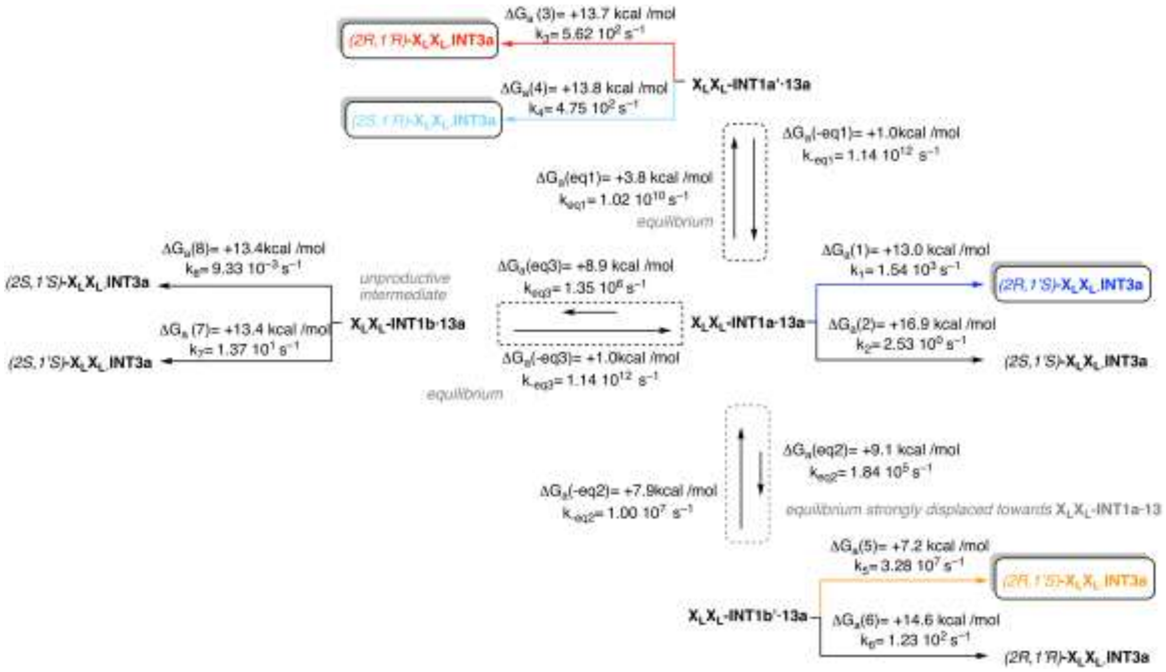

Scheme S2. Representation of the data used in the simulations for **10+13a→14** Michael reaction catalysed by **X$_L$X$_L$-9b** γ–dipeptide.

Kinetic equations associated with **10+13a→14** Michael reaction catalysed by **X$_L$X$_L$-9b** γ–dipeptide.

$$
\begin{aligned}
-\frac{\left[X_L X_L - INT1a{\cdot}13a\right]}{dt} &= +k_1\left[X_L X_L - INT1a{\cdot}13a\right] + k_2\left[X_L X_L - INT1a{\cdot}13a\right] \\
&-k_{-eq1}\left[X_L X_L - INT1a'{\cdot}13a\right] - k_{-eq2}\left[X_L X_L - INT1b{\cdot}13a\right] - k_{-eq3}\left[X_L X_L - INT1b'{\cdot}13a\right] \\
&+k_{eq1}\left[X_L X_L - INT1a{\cdot}13a\right] + k_{eq2}\left[X_L X_L - INT1a13a\right] + k_{eq3}\left[X_L X_L - INT1a{\cdot}13a\right]
\end{aligned}
$$

(eq S13)

$$\frac{\left[(2R,1'S)-INT\,3a(syn-(2R,1'S)-14\,fromINT\,1a)\right]}{dt}=+k_1\left[X_LX_L-INT\,1a\cdot 13a\right]$$

(eq S14)

$$\frac{\left[(2S,1'S)-INT\,3a(anti-(2S,1'S)-14\,fromINT\,1a)\right]}{dt}=+k_2\left[X_LX_L-INT\,1a\cdot 13a\right]$$

(eq S15)

$$-\frac{\left[X_LX_L-INT\,1b'\cdot 13a\right]}{dt}=+k_5\left[X_LX_L-INT\,1b'\cdot 13a\right]+k_6\left[X_LX_L-INT\,1b'\cdot 13a\right]$$
$$+k_{-eq2}\left[X_LX_L-INT\,1b'\cdot 13a\right]-k_{eq2}\left[X_LX_L-INT\,1a\cdot 13a\right]$$

(eq S16)

$$\frac{\left[(2R,1'S)-INT\,3b'(syn-(2R,1'S)-14\,fromINT\,1b')\right]}{dt}=+k_5\left[X_LX_L-INTb'\cdot 13a\right]$$

(eq S17)

$$\frac{\left[(2S,1'S)-INT\,3b'(syn-(2S,1'S)-14\,fromINT\,1b')\right]}{dt}=+k_6\left[X_LX_L-INT\,1b'\cdot 13b\right]$$

(eq S18)

$$-\frac{\left[X_LX_L-INT\,1a'\cdot 13a\right]}{dt}=+k_3\left[X_LX_L-INT\,1a'\cdot 13a\right]+k_4\left[X_LX_L-INT\,1a'\cdot 13a\right]$$
$$+k_{-eq1}\left[X_LX_L-INT\,1a'\cdot 13a\right]-k_{eq1}\left[X_LX_L-INT\,1a\cdot 13a\right]$$

(eq S19)

$$\frac{\left[(2R,1'R)-INT\,3a(anti-(2S,1'S)-14\,fromINT\,1a')\right]}{dt}=+k_4\left[X_LX_L-INTa'\cdot 13a\right]$$

(eq S20)

$$\frac{\left[(2S,1'R)-INT\,3a(syn-(2S,1'R)-14\,fromINT\,1a')\right]}{dt}=+k_3\left[X_LX_L-INT\,1a'\cdot 13a\right]$$

(eq S21)

$$-\frac{\left[X_LX_L-INT\,1b\cdot 13a\right]}{dt}=+k_7\left[X_LX_L-INT\,1b\cdot 13a\right]+k_8\left[X_LX_L-INT\,1b\cdot 13a\right]$$
$$+k_{-eq3}\left[X_LX_L-INT\,1b\cdot 13a\right]-k_{eq3}\left[X_LX_L-INT\,1a\cdot 13a\right]$$

(eq S22)

$$\frac{\left[(2R,1'R)-INT\,3a(anti-(2R,1'R)-14\,fromINTb)\right]}{dt}=+k_8\left[X_LX_L-INTb\cdot 13a\right]$$

(eq S23)

$$\frac{\left[(2S,1'R)-INT\,3a(syn-(2S,1'R)-14\,fromINT\,1b)\right]}{dt}=+k_7\left[X_LX_L-INT\,1b\cdot 13a\right]$$

(eq S24)

9.3 Computational data

Table S3. Total electronic energies (E, in a.u.),[a] zero point correction of the energy (ZPCE),[b] thermal corrections to Gibbs free energies (TCGFE, in a.u.),[b] and number of imaginary frequencies (NIMAG)[c] of all stationary points associated with the **10+11→12** Aldol reaction catalysed by **X_LX_L-9b** γ–dipeptide discussed in the main text and the Supporting Information.

Structure	E	ZPCE	TCGFE	NIMAG(ν)
**X_LX_L–INT1a·11**	-3024.988317	0.854386	0.761245	0
**X_LX_L–INT1a'·11**	-3024.989778	0.854690	0.762602	0
**X_LX_L–INT1b·11**	-3024.971100	0.853369	0.756606	0
**X_LX_L–INT1b'·11**	-3024.971208	0.853344	0.757037	0
**X_LX_L–INT1a**	-2183.204888	0.781897	0.699379	0
**X_LX_L–INT1b'**	-2183.207951	0.782678	0.702058	0
(*Si*,*Si*)–**X_LX_L–TS1a**	-3024.953627	0.854857	0.762043	1 (-123.6869)
(*2R*,*1'S*)–**X_LX_L–INT2a**	-3024.956559	0.854538	0.759151	0
(*Si*,*Si*)–**X_LX_L–TS1b'**	-3024.953858	0.854051	0.762172	1 (-97.5909)
(*2R*,*1'S*)–**X_LX_L–INT2b'**	-3024.984089	0.856604	0.764140	0
(*Si*,*Re*)–**X_LX_L–TS1a**	-3024.949082	0.854579	0.760988	1 (-243.0954)
(*2R*,*1'R*)–**X_LX_L–INT2a**	-3024.957764	0.854252	0.761069	0
(*Si*,*Re*)–**X_LX_L–TS1b'**	-3024.946788	0.854563	0.763238	1 (-126.0334)
(*2R*,*1'R*)–**X_LX_L–INT2b'**	-3024.974486	0.856830	0.764285	0
(*Re*,*Si*)–**X_LX_L–TS1a'**	-3024.939067	0.853976	0.760950	1(-262.1432)
(*2S*,*1'S*)–**X_LX_L–INT2a'**	-3024.957718	0.855787	0.762900	0
(*Re*,*Re*)–**X_LX_L–TS1b**	-3024.939067	0.853976	0.760950	1 (-262.1432)
(*2S*,*1'R*)–**X_LX_L–INT2b**	-3024.957718	0.855787	0.762900	0
(*Re*,*Re*)–**X_LX_L–TS1a'**	-3024.947535	0.854510	0.763686	1 (-290.7386)
(*2S*,*1'R*)–**X_LX_L–INT2a'**	-3024.957326	0.855502	0.764116	0
(*Re*,*Si*)–**X_LX_L–TS1b**	-3024.941439	0.853945	0.761284	1 (-287.1902)
(*2S*,*1'S*)–**X_LX_L–INT2b**	-3024.963777	0.856474	0.763431	0

[a] Computed at B3LYP-GD3/6-31G(d) level. [b] Computed at 298.15 K at B3LYP-GD3/6-31G(d) level [f] If NIMAG=1, the corresponding imaginary frequency ν (in parentheses) is given in cm^{-1}.

Table S4. Total electronic energies (E, in a.u.),[a] zero point correction of the energy (ZPCE),[b] thermal corrections to Gibbs free energies (TCGFE, in a.u.),[b] and number of imaginary frequencies (NIMAG)[c] of all stationary points associated with the **10+13a→14** Michael reaction catalysed by **X_LX_L-9b** γ–dipeptide discussed in the main text and the Supporting Information.

Structure	E	ZPCE	TCGFE	NIMAG(ν)
**X_LX_L–INT1a·13a**	-2697.432856	0.922349	0.828548	0
(*Re*,*Si*)–**X_LX_L–TS2a**	-2697.416090	0.922402	0.832607	1 (-377.5525)
(*2R*,*1'S*)–**X_LX_L–INT3a**	-2697.420653	0.922905	0.832099	0
(*Re*,*Re*)–**X_LX_L–TS2a**	-2697.409484	0.922861	0.832158	1 (-265.5073)
(*Re*,*Re*)–**X_LX_L–TS2a**	-2697.421326	0.92574	0.836794	0
**X_LX_L–INT1b'·13a**	-2697.418039	0.920754	0.824785	0
(*Re*,*Si*)–**X_LX_L–TS2b'**	-2697.411983	0.922210	0.830245	1 (-210.6123)

(2R,1'S)–**X_LX_L–INT3b**	-2697.430846	0.923545	0.830927	0
(*Re,Re*)–**X_LX_L–TS2b'**	-2697.412849	0.922711	0.831805	1 (-184.9989)
(2R,1'R)–**X_LX_L–INT3b**	-2697.428761	0.924995	0.835066	0
**X_LX_L–INT1a'·13a**	-2697.429958	0.92269	0.830169	0
(*Si,Re)*–**X_LX_L–TS2b**	-2697.414228	0.922163	0.831922	1 (-228.6582)
(2S,1'R)–**X_LX_L–INT3a**	-2697.432393	0.924276	0.834059	0
(*Si,Si*)–**X_LX_L–TS2a**	-2697.415286	0.922465	0.832900	1 (-256.5501)
(*Si,Si*)–**X_LX_L–TS2a**	-2697.4333	0.924177	0.835231	0
**X_LX_L–INT1b·13a**	-2697.429018	0.922065	0.828188	0
Si,Re)–**X_LX_L–TS2b**	-2697.409606	0.922336	0.830588	1 (-367.2144)
(2S,1'S)–**X_LX_L–INT3b**	-2697.417544	0.924503	0.833810	0
(*Si,Si*)–**X_LX_L–TS2b**	-2697.41333	0.922782	0.830386	1 (-243.8130)
(2S,1'S)–**X_LX_L–INT3a**	-2697.423132	0.924533	0.831132	0

Cartesian coordinates of all stationary points discussed in the main text

X_LX_L–INT1a·11

Center Number	Atomic Number	Atomic Type	Coordinates (Angstroms) X	Y	Z
1	7	0	-0.359655	2.422337	1.474951
2	6	0	-0.412104	1.098052	2.071030
3	6	0	-1.923865	0.831175	2.268083
4	6	0	-2.509371	1.518419	1.027972
5	6	0	-1.602692	2.756268	0.783831
6	6	0	0.195856	0.098608	1.071139
7	8	0	-0.345151	-0.105939	-0.019291
8	7	0	1.380671	-0.481364	1.412519
9	6	0	2.195302	-1.231755	0.471859
10	6	0	1.419840	-2.412755	-0.144958
11	7	0	2.099860	-2.594366	-1.521422
12	6	0	3.069972	-1.456828	-1.780736
13	6	0	2.649944	-0.382943	-0.740363
14	1	0	1.361230	-2.542990	-2.242239
15	1	0	2.584648	-3.497655	-1.553534
16	7	0	-3.935184	1.966994	1.240486
17	8	0	-4.755938	1.619896	0.390962
18	8	0	-4.177806	2.658036	2.222473
19	6	0	-2.304217	-0.620011	2.497399
20	6	0	-3.463782	-1.179769	1.945697
21	6	0	-3.806530	-2.509366	2.199499
22	6	0	-2.995621	-3.303520	3.010125
23	6	0	-1.840753	-2.756022	3.571294
24	6	0	-1.503009	-1.426589	3.319256
25	6	0	4.487243	-1.968180	-1.582902
26	8	0	4.754747	-2.930193	-0.886348
27	8	0	5.351304	-1.207141	-2.233590
28	6	0	6.757662	-1.489217	-2.013428
29	6	0	1.327341	-3.684790	0.647721
30	6	0	0.058668	-4.163563	0.998683
31	6	0	-0.063978	-5.319904	1.769324
32	6	0	1.075829	-6.010433	2.182979
33	6	0	2.343933	-5.540042	1.829110
34	6	0	2.473171	-4.378057	1.069958
35	6	0	3.661286	0.698251	-0.433192
36	6	0	4.765959	0.468720	0.401025
37	6	0	5.675041	1.491113	0.672081
38	6	0	5.494409	2.758014	0.111630
39	6	0	4.399127	2.996640	-0.719313
40	6	0	3.489609	1.972378	-0.986819
41	6	0	-1.445673	3.070158	-0.698117
42	6	0	-2.494851	3.690018	-1.387290
43	6	0	-2.371151	3.986210	-2.745611
44	6	0	-1.194559	3.670778	-3.428059
45	6	0	-0.145481	3.051011	-2.744052
46	6	0	-0.273348	2.750079	-1.387641
47	1	0	0.114953	1.068328	3.026671
48	1	0	-2.229795	1.433592	3.133185
49	1	0	-2.533724	0.861557	0.165331
50	1	0	-2.065283	3.619454	1.284237
51	1	0	1.798042	-0.238067	2.298958
52	1	0	3.058587	-1.611679	1.021819
53	1	0	0.429199	-2.049655	-0.385394
54	1	0	2.924662	-1.100752	-2.799492
55	1	0	1.749920	0.079349	-1.154207
56	1	0	-4.122815	-0.586016	1.322986
57	1	0	-4.708123	-2.918600	1.755128
58	1	0	-3.265825	-4.336421	3.210754
59	1	0	-1.200068	-3.362708	4.204441
60	1	0	-0.608049	-1.016829	3.779970
61	1	0	6.968355	-2.538409	-2.228579
62	1	0	7.289792	-0.830475	-2.697133
63	1	0	7.013402	-1.260411	-0.976345
64	1	0	-0.834984	-3.638833	0.676399
65	1	0	-1.052109	-5.677559	2.041936
66	1	0	0.979015	-6.913621	2.778438
67	1	0	3.232776	-6.076167	2.148458
68	1	0	3.461167	-4.018221	0.796953

69	1	0	4.932198	-0.513353	0.837826
70	1	0	6.522852	1.299612	1.323648
71	1	0	6.199957	3.554740	0.327536
72	1	0	4.245132	3.979796	-1.153978
73	1	0	2.638130	2.167749	-1.632551
74	1	0	-3.410391	3.951336	-0.861747
75	1	0	-3.192229	4.467938	-3.268348
76	1	0	-1.093895	3.913155	-4.482205
77	1	0	0.780738	2.818460	-3.265990
78	1	0	0.540756	2.291404	-0.840935
79	6	0	0.573610	3.422160	1.820738
80	6	0	0.435298	4.702631	1.424951
81	6	0	1.764436	2.964195	2.642474
82	6	0	1.389249	5.808199	1.805319
83	1	0	-0.393083	4.991198	0.786263
84	6	0	2.887831	4.009698	2.690742
85	1	0	1.440731	2.728034	3.667961
86	1	0	2.166876	2.035627	2.215799
87	6	0	2.321985	5.406450	2.953369
88	1	0	0.815822	6.703506	2.079593
89	1	0	1.988434	6.100346	0.927283
90	1	0	3.617456	3.723242	3.457203
91	1	0	3.416365	4.006606	1.729750
92	1	0	3.130721	6.137872	3.063862
93	1	0	1.761778	5.401687	3.898396
94	6	0	-0.532223	-0.492915	-3.326809
95	8	0	0.517209	-1.128296	-3.265192
96	1	0	-0.563439	0.463543	-3.868739
97	6	0	-1.795650	-0.885572	-2.696554
98	6	0	-2.902421	-0.020181	-2.764500
99	6	0	-1.952979	-2.067255	-1.956235
100	6	0	-4.089981	-0.283499	-2.087938
101	9	0	-2.830556	1.090781	-3.496406
102	6	0	-3.119789	-2.349643	-1.268986
103	9	0	-0.957307	-2.967934	-1.874285
104	6	0	-4.187009	-1.448299	-1.325837
105	9	0	-5.120606	0.545329	-2.180907
106	9	0	-3.221222	-3.468708	-0.548206
107	9	0	-5.296311	-1.704413	-0.644073

X_LX_L–INT1a’·11

Center Number	Atomic Number	Atomic Type	Coordinates (Angstroms) X	Y	Z
1	7	0	0.416394	2.455357	-1.651912
2	6	0	0.442328	1.092927	-2.135776
3	6	0	1.957910	0.811895	-2.289897
4	6	0	2.520620	1.550812	-1.066959
5	6	0	1.610430	2.799231	-0.879331
6	6	0	-0.186611	0.131994	-1.107573
7	8	0	0.318310	-0.002568	0.011238
8	7	0	-1.332050	-0.511942	-1.464352
9	6	0	-2.144287	-1.268575	-0.525263
10	6	0	-1.339931	-2.410760	0.129241
11	7	0	-2.023167	-2.579326	1.505374
12	6	0	-3.039654	-1.476078	1.726108
13	6	0	-2.659494	-0.412369	0.658969
14	1	0	-1.294258	-2.480040	2.231174
15	1	0	-2.471366	-3.500268	1.557968
16	7	0	3.950279	1.992480	-1.272493
17	8	0	4.756580	1.681945	-0.395398
18	8	0	4.208736	2.644343	-2.276929
19	6	0	2.349406	-0.644209	-2.455880
20	6	0	3.498299	-1.177935	-1.858438
21	6	0	3.862656	-2.509170	-2.071407
22	6	0	3.083279	-3.330465	-2.885618
23	6	0	1.937925	-2.809542	-3.490306
24	6	0	1.578751	-1.478853	-3.279012
25	6	0	-4.433756	-2.052141	1.540325
26	8	0	-4.660444	-3.033914	0.857049
27	8	0	-5.328623	-1.324211	2.187413
28	6	0	-6.721713	-1.667892	1.971815
29	6	0	-1.209101	-3.703196	-0.625511
30	6	0	0.073581	-4.180488	-0.922907

31	6	0	0.230312	-5.360353	-1.650533
32	6	0	-0.889718	-6.075748	-2.075282
33	6	0	-2.172136	-5.607454	-1.774256
34	6	0	-2.334983	-4.423335	-1.057132
35	6	0	-3.729986	0.603028	0.327751
36	6	0	-4.827664	0.289843	-0.487270
37	6	0	-5.811035	1.243551	-0.750378
38	6	0	-5.710341	2.524838	-0.202220
39	6	0	-4.616751	2.848997	0.601700
40	6	0	-3.633458	1.893766	0.860260
41	6	0	1.376746	3.127365	0.590709
42	6	0	2.397754	3.752564	1.317966
43	6	0	2.215092	4.076310	2.662471
44	6	0	1.005869	3.782392	3.296403
45	6	0	-0.016367	3.160632	2.575476
46	6	0	0.169200	2.831792	1.231420
47	1	0	-0.081511	1.038738	-3.096877
48	1	0	2.279351	1.377857	-3.173293
49	1	0	2.531433	0.927822	-0.179166
50	1	0	2.117303	3.652851	-1.345327
51	1	0	-1.718880	-0.335166	-2.380120
52	1	0	-2.980945	-1.687345	-1.087750
53	1	0	-0.360368	-2.015343	0.366387
54	1	0	-2.915119	-1.084684	2.734468
55	1	0	-1.791767	0.107778	1.071657
56	1	0	4.135288	-0.560724	-1.235614
57	1	0	4.757085	-2.897130	-1.594775
58	1	0	3.370710	-4.364135	-3.055931
59	1	0	1.321945	-3.438394	-4.126292
60	1	0	0.694369	-1.087690	-3.775023
61	1	0	-6.885766	-2.725168	2.187897
62	1	0	-7.280328	-1.032573	2.656590
63	1	0	-6.989475	-1.450272	0.935345
64	1	0	0.952549	-3.636259	-0.593153
65	1	0	1.229559	-5.716007	-1.882042
66	1	0	-0.766516	-6.996581	-2.637934
67	1	0	-3.046055	-6.163013	-2.101453
68	1	0	-3.334094	-4.068501	-0.821623
69	1	0	-4.932926	-0.706305	-0.910834
70	1	0	-6.654182	0.987211	-1.385534
71	1	0	-6.474732	3.267842	-0.409442
72	1	0	-4.522987	3.846210	1.021056
73	1	0	-2.783151	2.154254	1.484563
74	1	0	3.339531	3.994917	0.830926
75	1	0	3.015569	4.561771	3.212989
76	1	0	0.858793	4.045769	4.339989
77	1	0	-0.967257	2.944632	3.058287
78	1	0	-0.631438	2.373848	0.664584
79	6	0	-0.651976	3.339642	-1.882793
80	6	0	-1.879383	2.918067	-2.248741
81	6	0	-0.322861	4.812337	-1.717239
82	6	0	-3.028701	3.844888	-2.559319
83	1	0	-2.091445	1.856255	-2.315250
84	6	0	-1.401060	5.732636	-2.309325
85	1	0	-0.188340	5.030738	-0.649077
86	1	0	0.640503	5.029032	-2.195640
87	6	0	-2.807188	5.237533	-1.959995
88	1	0	-3.957134	3.408345	-2.173103
89	1	0	-3.166886	3.931994	-3.649202
90	1	0	-1.240124	6.755270	-1.950345
91	1	0	-1.294861	5.759106	-3.402066
92	1	0	-3.566242	5.938628	-2.325603
93	1	0	-2.915011	5.181701	-0.867269
94	6	0	0.511604	-0.357129	3.279463
95	8	0	-0.525897	-1.012094	3.232040
96	1	0	0.517742	0.627414	3.769536
97	6	0	1.793338	-0.764290	2.695721
98	6	0	2.891121	0.112683	2.754750
99	6	0	1.978515	-1.974124	2.009447
100	6	0	4.097507	-0.167849	2.119591
101	9	0	2.792083	1.251500	3.439451
102	6	0	3.165243	-2.275626	1.365659
103	9	0	0.992344	-2.886522	1.938503
104	6	0	4.222392	-1.361940	1.408685
105	9	0	5.118369	0.674122	2.201550
106	9	0	3.293864	-3.422989	0.695802
107	9	0	5.347962	-1.633840	0.760613

X_LX_L–INT1b·11

Center Number	Atomic Number	Atomic Type	Coordinates (Angstroms) X	Y	Z
1	7	0	-2.109126	-1.242706	0.802032
2	6	0	-0.757551	-1.901853	0.768477
3	6	0	-0.800693	-2.774236	-0.520543
4	6	0	-1.598056	-1.846561	-1.448838
5	6	0	-2.774530	-1.399726	-0.560608
6	6	0	0.236846	-0.696617	0.760121
7	8	0	-0.233960	0.435308	0.924346
8	7	0	1.529247	-0.972756	0.579727
9	6	0	2.538653	0.054449	0.354904
10	6	0	3.926407	-0.392727	0.862653
11	7	0	4.822248	0.402220	0.029001
12	6	0	4.153947	1.071919	-1.067863
13	6	0	2.778574	0.350984	-1.137750
14	1	0	-2.761099	-1.584484	1.536267
15	1	0	-1.888560	-0.240439	0.999818
16	6	0	3.976077	2.575019	-0.818861
17	8	0	3.639213	3.060502	0.242774
18	8	0	4.180583	3.277812	-1.940520
19	6	0	3.941172	4.697631	-1.843920
20	6	0	0.537916	-3.307463	-0.987405
21	6	0	1.243121	-4.170116	-0.130794
22	6	0	2.523694	-4.611928	-0.464796
23	6	0	3.105818	-4.216342	-1.672877
24	6	0	2.392353	-3.394775	-2.546082
25	6	0	1.115960	-2.938703	-2.205639
26	6	0	1.642026	1.024274	-1.878673
27	6	0	0.981147	2.158996	-1.381043
28	6	0	-0.067100	2.738492	-2.095568
29	6	0	-0.498498	2.182177	-3.302870
30	6	0	0.143511	1.049842	-3.804914
31	6	0	1.212951	0.489572	-3.101263
32	7	0	-2.134292	-2.570598	-2.661330
33	8	0	-1.751675	-2.182022	-3.756066
34	8	0	-2.915206	-3.494178	-2.450171
35	6	0	4.056610	-0.208778	2.363071
36	6	0	3.937324	-1.313960	3.210078
37	6	0	4.005332	-1.155888	4.595788
38	6	0	4.200416	0.113766	5.141193
39	6	0	4.321590	1.221378	4.296528
40	6	0	4.247473	1.065020	2.913167
41	6	0	-3.543255	-0.184237	-1.002886
42	6	0	-4.864808	-0.343541	-1.439557
43	6	0	-5.594408	0.759429	-1.884022
44	6	0	-5.009442	2.028246	-1.884864
45	6	0	-3.689776	2.187519	-1.454205
46	6	0	-2.952226	1.086370	-1.021700
47	1	0	-0.614450	-2.501953	1.669247
48	1	0	-1.455319	-3.628232	-0.300757
49	1	0	-1.015369	-1.004963	-1.829393
50	1	0	-3.445824	-2.255705	-0.464726
51	1	0	1.796409	-1.932191	0.393136
52	1	0	2.227788	0.954487	0.885045
53	1	0	4.038160	-1.471674	0.638708
54	1	0	4.707866	0.942423	-2.003909
55	1	0	2.974168	-0.612765	-1.625180
56	1	0	4.213475	5.105895	-2.816623
57	1	0	2.884976	4.881947	-1.629069
58	1	0	4.553992	5.132821	-1.051114
59	1	0	0.792981	-4.490122	0.806911
60	1	0	3.060599	-5.270361	0.211460
61	1	0	4.101248	-4.560361	-1.936232
62	1	0	2.827425	-3.103437	-3.497648
63	1	0	0.581595	-2.298868	-2.897071
64	1	0	1.279725	2.591253	-0.433290
65	1	0	-0.556085	3.624604	-1.698484
66	1	0	-1.327286	2.626431	-3.846350
67	1	0	-0.179159	0.605003	-4.741602
68	1	0	1.731911	-0.370879	-3.515169
69	1	0	3.808152	-2.307456	2.783358

70	1	0	3.920259	-2.022609	5.245766
71	1	0	4.264837	0.239799	6.218356
72	1	0	4.477267	2.210828	4.717619
73	1	0	4.337802	1.919984	2.250209
74	1	0	-5.319355	-1.331208	-1.441374
75	1	0	-6.616123	0.627904	-2.227695
76	1	0	-5.580189	2.889497	-2.219327
77	1	0	-3.229090	3.170190	-1.455524
78	1	0	-1.921504	1.225502	-0.708465
79	6	0	-5.382855	-1.839739	2.283453
80	8	0	-4.219663	-2.231768	2.306370
81	1	0	-6.195937	-2.548146	2.505169
82	6	0	-5.817624	-0.477068	1.946880
83	6	0	-4.950788	0.629283	1.899414
84	6	0	-7.162687	-0.255286	1.597671
85	6	0	-5.384523	1.882066	1.499255
86	9	0	-3.663669	0.501245	2.255279
87	6	0	-7.616517	0.989478	1.176923
88	9	0	-8.029939	-1.269517	1.626576
89	6	0	-6.718011	2.055499	1.118427
90	9	0	-4.539229	2.908139	1.441248
91	9	0	-8.882616	1.163990	0.810061
92	9	0	-7.124180	3.239261	0.686119
93	6	0	6.224199	0.429946	0.144035
94	6	0	6.977009	1.319221	-0.535589
95	6	0	6.847776	-0.617882	1.048378
96	6	0	8.484434	1.371111	-0.472298
97	1	0	6.507105	2.058162	-1.179570
98	6	0	8.344481	-0.807282	0.766810
99	1	0	6.702455	-0.330347	2.097201
100	1	0	6.325472	-1.575730	0.925876
101	6	0	9.051596	0.548544	0.691492
102	1	0	8.810317	2.416768	-0.387042
103	1	0	8.910178	1.007845	-1.421933
104	1	0	8.784573	-1.438858	1.547347
105	1	0	8.474431	-1.337295	-0.186945
106	1	0	10.133699	0.418625	0.574935
107	1	0	8.891953	1.088552	1.634884

X_LX_L–INT1b’·11

Center Number	Atomic Number	Atomic Type	Coordinates (Angstroms) X	Y	Z
1	7	0	-2.090596	1.323587	-0.638897
2	6	0	-0.731448	1.959263	-0.529123
3	6	0	-0.762138	2.667550	0.857343
4	6	0	-1.568386	1.641552	1.666339
5	6	0	-2.751916	1.323587	0.734995
6	6	0	0.250165	0.752998	-0.673362
7	8	0	-0.232905	-0.345471	-0.974132
8	7	0	1.545799	0.992758	-0.468651
9	6	0	2.550343	-0.059627	-0.401632
10	6	0	3.932970	0.441432	-0.856595
11	7	0	4.825782	-0.527387	-0.237654
12	6	0	4.182677	-1.281784	0.835050
13	6	0	2.821372	-0.549799	1.033102
14	1	0	-2.739925	1.758974	-1.324054
15	1	0	-1.882037	0.350335	-0.957246
16	6	0	3.956034	-2.743965	0.442934
17	8	0	3.640490	-3.118040	-0.668689
18	8	0	4.084198	-3.548027	1.506850
19	6	0	3.768936	-4.937961	1.284094
20	6	0	0.584099	3.123173	1.380208
21	6	0	1.294669	4.080596	0.636120
22	6	0	2.584463	4.457570	1.011360
23	6	0	3.171142	3.899144	2.151086
24	6	0	2.452530	2.981170	2.917482
25	6	0	1.166335	2.591080	2.534533
26	6	0	1.686555	-1.286186	1.712539
27	6	0	0.988237	-2.334570	1.091784
28	6	0	-0.067721	-2.967377	1.747150
29	6	0	-0.468960	-2.548666	3.018919
30	6	0	0.212088	-1.504163	3.644696
31	6	0	1.288401	-0.891993	2.996991

32	7	0	-2.090152	2.215020	2.962598
33	8	0	-1.701089	1.693380	3.998141
34	8	0	-2.865680	3.162597	2.873009
35	6	0	4.042862	0.535486	-2.365306
36	6	0	3.945329	1.776446	-2.999827
37	6	0	3.979427	1.863820	-4.393223
38	6	0	4.113821	0.704681	-5.158578
39	6	0	4.211938	-0.538574	-4.526085
40	6	0	4.175238	-0.625886	-3.135429
41	6	0	-3.536370	0.074137	1.030519
42	6	0	-4.856451	0.196871	1.483105
43	6	0	-5.599724	-0.942050	1.793929
44	6	0	-5.030256	-2.208852	1.643036
45	6	0	-3.712279	-2.332388	1.195541
46	6	0	-2.960446	-1.196725	0.898283
47	1	0	-0.583121	2.664424	-1.349406
48	1	0	-1.407255	3.549369	0.745982
49	1	0	-0.994829	0.751912	1.935952
50	1	0	-3.411547	2.193681	0.744971
51	1	0	1.827062	1.921009	-0.174983
52	1	0	2.224357	-0.879740	-1.041783
53	1	0	4.089316	1.452521	-0.426870
54	1	0	4.753485	-1.258277	1.767110
55	1	0	3.049486	0.341929	1.631173
56	1	0	3.991056	-5.441863	2.224200
57	1	0	2.710124	-5.041588	1.031216
58	1	0	4.377091	-5.340778	0.470782
59	1	0	0.841975	4.526166	-0.247541
60	1	0	3.125730	5.190724	0.420961
61	1	0	4.174548	4.190727	2.445505
62	1	0	2.891687	2.562578	3.818423
63	1	0	0.629210	1.873315	3.142308
64	1	0	1.262587	-2.656321	0.093649
65	1	0	-0.587890	-3.784591	1.253528
66	1	0	-1.304860	-3.031742	3.516513
67	1	0	-0.086172	-1.167973	4.633332
68	1	0	1.835875	-0.099893	3.500918
69	1	0	3.859079	2.682172	-2.400848
70	1	0	3.913176	2.834086	-4.878192
71	1	0	4.148876	0.769121	-6.242572
72	1	0	4.320868	-1.442321	-5.119417
73	1	0	4.249370	-1.585519	-2.631536
74	1	0	-5.298750	1.182988	1.601715
75	1	0	-6.620024	-0.840041	2.151567
76	1	0	-5.611951	-3.096706	1.872793
77	1	0	-3.264088	-3.314170	1.080227
78	1	0	-1.931135	-1.308459	0.569974
79	6	0	-5.368771	2.129003	-2.009618
80	8	0	-4.200189	2.505166	-1.994092
81	1	0	-6.172709	2.870353	-2.136771
82	6	0	-5.822632	0.741964	-1.839439
83	6	0	-4.973907	-0.375411	-1.932223
84	6	0	-7.171200	0.499682	-1.518170
85	6	0	-5.428218	-1.661579	-1.693017
86	9	0	-3.684478	-0.225049	-2.269727
87	6	0	-7.645530	-0.780714	-1.257465
88	9	0	-8.022047	1.523337	-1.418288
89	6	0	-6.764794	-1.860197	-1.336215
90	9	0	-4.599485	-2.700098	-1.766174
91	9	0	-8.914511	-0.979780	-0.913826
92	9	0	-7.191137	-3.082440	-1.057807
93	6	0	6.224297	-0.344887	-0.249330
94	6	0	6.814524	0.768663	-0.721314
95	6	0	7.025513	-1.538069	0.240669
96	6	0	8.309732	0.951912	-0.808311
97	1	0	6.211755	1.582075	-1.114004
98	6	0	8.489229	-1.483411	-0.219078
99	1	0	6.990054	-1.593680	1.339165
100	1	0	6.556631	-2.459282	-0.128523
101	6	0	9.078142	-0.089269	0.015849
102	1	0	8.574511	1.965360	-0.476468
103	1	0	8.625911	0.895897	-1.862412
104	1	0	9.069200	-2.251010	0.306067
105	1	0	8.538350	-1.720346	-1.290351
106	1	0	10.143036	-0.068850	-0.242897
107	1	0	9.002843	0.158958	1.083871

X_LX_L–INT1a

Center Number	Atomic Number	Atomic Type	Coordinates (Angstroms) X	Y	Z
1	7	0	3.177930	0.367335	-0.592307
2	6	0	1.923974	0.008732	-1.196305
3	6	0	1.932830	-1.552440	-1.366733
4	6	0	3.380467	-1.944306	-1.020207
5	6	0	3.998421	-0.774029	-0.210751
6	6	0	0.710904	0.509422	-0.393872
7	8	0	0.769575	0.961964	0.737825
8	7	0	-0.485260	0.428625	-1.081329
9	6	0	-1.737267	0.649446	-0.392450
10	6	0	-2.491210	1.888923	-0.987494
11	7	0	-3.864589	1.329011	-1.353521
12	6	0	-3.665181	-0.134592	-1.637042
13	6	0	-2.723753	-0.552263	-0.476352
14	6	0	3.662553	1.685279	-0.677934
15	6	0	5.083687	1.899044	-0.190876
16	6	0	5.407502	3.382065	0.031945
17	6	0	4.942797	4.222326	-1.159665
18	6	0	3.419592	4.119317	-1.307023
19	6	0	2.919613	2.702349	-1.161441
20	1	0	-4.312569	1.821721	-2.130909
21	1	0	-4.522143	1.399485	-0.560794
22	6	0	0.884319	-2.344627	-0.603764
23	6	0	0.761358	-2.288817	0.793058
24	6	0	-0.214554	-3.039711	1.447864
25	6	0	-1.075847	-3.867287	0.723300
26	6	0	-0.962748	-3.932603	-0.666840
27	6	0	0.009034	-3.171354	-1.322668
28	6	0	4.068161	-1.080064	1.285451
29	6	0	4.850222	-2.161456	1.714192
30	6	0	4.952385	-2.463275	3.070660
31	6	0	4.279242	-1.680861	4.014031
32	6	0	3.511913	-0.595804	3.590784
33	6	0	3.407424	-0.290458	2.230066
34	7	0	4.225399	-2.093296	-2.275470
35	8	0	5.224739	-2.794065	-2.164590
36	8	0	3.897371	-1.464751	-3.278987
37	6	0	-3.521448	-0.804714	0.796669
38	6	0	-4.012232	-2.097628	1.034293
39	6	0	-4.812370	-2.361884	2.144422
40	6	0	-5.137593	-1.336279	3.035826
41	6	0	-4.649610	-0.049443	2.813635
42	6	0	-3.845502	0.216482	1.702052
43	6	0	-5.035317	-0.797018	-1.668316
44	8	0	-6.046641	-0.191220	-1.384304
45	8	0	-4.933956	-2.070866	-2.015679
46	6	0	-6.168891	-2.839446	-1.967920
47	6	0	-2.593768	3.125980	-0.119298
48	6	0	-1.505116	3.481706	0.691436
49	6	0	-1.562678	4.642521	1.462780
50	6	0	-2.697470	5.455236	1.438446
51	6	0	-3.778610	5.109807	0.627311
52	6	0	-3.723865	3.955496	-0.154911
53	1	0	1.858370	0.467061	-2.196610
54	1	0	1.789949	-1.761004	-2.428354
55	1	0	3.467087	-2.901854	-0.512170
56	1	0	5.026905	-0.630507	-0.557706
57	1	0	-0.491931	-0.101577	-1.944059
58	1	0	-1.468786	0.831940	0.646993
59	1	0	-2.027677	2.131470	-1.949761
60	1	0	-3.167314	-0.223572	-2.607584
61	1	0	-2.185026	-1.462170	-0.752076
62	1	0	5.785934	1.477122	-0.925754
63	1	0	5.243850	1.346991	0.742660
64	1	0	4.899974	3.729221	0.942344
65	1	0	6.483803	3.497143	0.202090
66	1	0	5.426042	3.850343	-2.073483
67	1	0	5.243760	5.269514	-1.040925
68	1	0	2.933945	4.762628	-0.554441
69	1	0	3.108817	4.521277	-2.281554
70	1	0	1.886336	2.540072	-1.456666

71	1	0	1.426806	-1.658892	1.372299
72	1	0	-0.294112	-2.984443	2.529572
73	1	0	-1.822322	-4.462423	1.241112
74	1	0	-1.616271	-4.584077	-1.241095
75	1	0	0.102836	-3.239784	-2.404778
76	1	0	5.385463	-2.763572	0.981962
77	1	0	5.560575	-3.303922	3.392483
78	1	0	4.360408	-1.914297	5.071906
79	1	0	2.992369	0.021102	4.319113
80	1	0	2.799000	0.542469	1.895765
81	1	0	-3.754002	-2.897628	0.348254
82	1	0	-5.174434	-3.370986	2.319541
83	1	0	-5.759031	-1.542317	3.901967
84	1	0	-4.885156	0.751989	3.507445
85	1	0	-3.454827	1.223661	1.577186
86	1	0	-6.533506	-2.868290	-0.938758
87	1	0	-6.917825	-2.381954	-2.616863
88	1	0	-5.898553	-3.833621	-2.318949
89	1	0	-0.614347	2.861177	0.730168
90	1	0	-0.715157	4.906389	2.087959
91	1	0	-2.738341	6.354670	2.045182
92	1	0	-4.663786	5.737674	0.596068
93	1	0	-4.576372	3.728913	-0.792648

X_LX_L–INT1b’

Center Number	Atomic Number	Atomic Type	Coordinates (Angstroms) X	Y	Z
1	7	0	-2.978526	0.123422	-1.025004
2	6	0	-2.252283	1.438722	-1.232239
3	6	0	-2.729566	2.278741	-0.020337
4	6	0	-4.224037	1.831558	0.128667
5	6	0	-4.407287	0.482224	-0.609672
6	6	0	-0.813232	1.006490	-1.593830
7	8	0	-0.718313	0.415725	-2.664060
8	7	0	0.161884	1.135993	-0.673057
9	6	0	1.292010	0.220269	-0.655697
10	6	0	2.578496	0.807252	-0.055748
11	7	0	3.306058	-0.402829	0.317918
12	6	0	2.446794	-1.583661	0.390276
13	6	0	1.018237	-0.986197	0.266550
14	6	0	4.496904	-0.338606	1.072251
15	6	0	4.975341	0.807965	1.588937
16	6	0	6.285252	0.910406	2.330716
17	6	0	6.835249	-0.460558	2.745154
18	6	0	6.719158	-1.447150	1.579585
19	6	0	5.248750	-1.652930	1.188934
20	1	0	-2.513061	-0.448313	-0.298337
21	1	0	-2.928702	-0.451260	-1.875585
22	6	0	-1.929631	2.137666	1.262635
23	6	0	-1.060495	3.175870	1.633185
24	6	0	-0.224334	3.044108	2.744056
25	6	0	-0.255557	1.876763	3.509810
26	6	0	-1.136129	0.848793	3.165537
27	6	0	-1.965447	0.978090	2.050871
28	6	0	-5.075959	-0.675002	0.091128
29	6	0	-5.155887	-0.793890	1.484352
30	6	0	-5.718343	-1.934076	2.061098
31	6	0	-6.204993	-2.963455	1.253550
32	6	0	-6.143641	-2.844545	-0.137392
33	6	0	-5.585986	-1.705011	-0.713116
34	7	0	-5.098034	2.883892	-0.536491
35	8	0	-5.795490	3.564105	0.195881
36	8	0	-4.990307	2.994853	-1.758140
37	6	0	-0.145221	-1.863758	-0.147071
38	6	0	-1.143659	-2.169622	0.794260
39	6	0	-2.286799	-2.892389	0.434774
40	6	0	-2.445922	-3.327731	-0.883677
41	6	0	-1.453832	-3.043864	-1.826750
42	6	0	-0.316643	-2.317802	-1.464891
43	6	0	2.718366	-2.562511	-0.755819
44	8	0	2.952586	-2.233410	-1.900772
45	8	0	2.592518	-3.830845	-0.341198
46	6	0	2.739603	-4.840485	-1.362867

47	6	0	3.311035	1.711812	-1.025489
48	6	0	4.015851	1.166149	-2.104687
49	6	0	4.637810	2.006733	-3.026305
50	6	0	4.558932	3.395064	-2.880948
51	6	0	3.854081	3.941622	-1.807765
52	6	0	3.233405	3.099323	-0.883105
53	1	0	-2.689914	1.866991	-2.136321
54	1	0	-2.703311	3.328391	-0.323059
55	1	0	-4.546092	1.819278	1.165397
56	1	0	-4.909490	0.689735	-1.557232
57	1	0	-0.048449	1.617522	0.191648
58	1	0	1.480919	-0.100441	-1.679640
59	1	0	2.303820	1.397386	0.841934
60	1	0	2.549732	-2.121315	1.336523
61	1	0	0.784393	-0.575245	1.258126
62	1	0	4.440193	1.740283	1.436530
63	1	0	7.023765	1.429951	1.699357
64	1	0	6.156399	1.548450	3.215927
65	1	0	7.876673	-0.368965	3.074101
66	1	0	6.261412	-0.843953	3.600663
67	1	0	7.268306	-1.051408	0.714832
68	1	0	7.171780	-2.412776	1.832584
69	1	0	5.192602	-2.184273	0.229898
70	1	0	4.762577	-2.304906	1.930548
71	1	0	-1.024316	4.085686	1.039184
72	1	0	0.445699	3.855389	3.011405
73	1	0	0.393971	1.772921	4.373286
74	1	0	-1.178412	-0.055855	3.765250
75	1	0	-2.632442	0.158553	1.804809
76	1	0	-4.798436	-0.003594	2.135963
77	1	0	-5.784111	-2.010864	3.142048
78	1	0	-6.645695	-3.846984	1.705125
79	1	0	-6.540441	-3.631246	-0.771737
80	1	0	-5.551021	-1.614350	-1.796826
81	1	0	-1.017830	-1.841869	1.822786
82	1	0	-3.050349	-3.108919	1.176066
83	1	0	-3.332318	-3.886718	-1.166960
84	1	0	-1.563394	-3.383766	-2.852796
85	1	0	0.425821	-2.084833	-2.218945
86	1	0	3.713220	-4.747578	-1.849688
87	1	0	2.652672	-5.794727	-0.844409
88	1	0	1.950586	-4.732681	-2.112132
89	1	0	4.068828	0.086067	-2.208821
90	1	0	5.187475	1.578534	-3.859969
91	1	0	5.048493	4.046866	-3.599064
92	1	0	3.794791	5.019675	-1.685367
93	1	0	2.693699	3.524115	-0.037874

(*Si*,*Si*)–**X_LX_L–TS1a**

Center Number	Atomic Number	Atomic Type	Coordinates (Angstroms) X	Y	Z
1	6	0	3.971475	4.484522	0.799669
2	6	0	3.410206	3.355836	1.401975
3	6	0	2.347862	2.712152	0.781670
4	6	0	1.810729	3.142227	-0.435989
5	6	0	2.392367	4.281305	-1.005076
6	6	0	3.457658	4.953027	-0.408828
7	6	0	0.695034	2.435380	-1.135665
8	8	0	0.609402	1.172595	-1.094139
9	9	0	1.814043	1.644839	1.430107
10	9	0	1.924143	4.754595	-2.170811
11	9	0	3.888537	2.909283	2.564043
12	9	0	3.988730	6.027239	-0.987799
13	9	0	4.988723	5.109636	1.381603
14	6	0	-0.923514	3.231023	0.165592
15	6	0	-1.983991	2.615008	-0.510509
16	6	0	-2.548245	3.321564	-1.731083
17	6	0	-2.581892	4.851277	-1.577238
18	6	0	-1.226509	5.402223	-1.134549
19	6	0	-0.802978	4.747399	0.184149
20	7	0	-2.428579	1.365689	-0.218788
21	6	0	-2.161505	0.660013	1.053534
22	6	0	-3.593214	0.460945	1.605191

23	6	0	-4.347881	0.022845	0.341213
24	6	0	-3.631844	0.742144	-0.841214
25	6	0	-1.448678	-0.675239	0.771253
26	7	0	-0.108748	-0.706078	0.993564
27	6	0	0.638505	-1.867713	0.509536
28	6	0	0.612968	-1.921835	-1.049993
29	7	0	1.852935	-1.124170	-1.433404
30	6	0	2.777233	-0.970111	-0.254451
31	6	0	2.119254	-1.745726	0.944392
32	6	0	-3.737584	-0.375049	2.859337
33	6	0	-3.817503	-1.774419	2.850372
34	6	0	-3.932520	-2.481195	4.047813
35	6	0	-3.964195	-1.805027	5.268531
36	6	0	-3.885652	-0.411660	5.287799
37	6	0	-3.777688	0.294886	4.090623
38	7	0	-5.794628	0.470955	0.397471
39	8	0	-6.007378	1.664287	0.603253
40	6	0	-3.321511	-0.160087	-2.026662
41	6	0	-4.309232	-1.038242	-2.489735
42	6	0	-4.058453	-1.867353	-3.583373
43	6	0	-2.822155	-1.824094	-4.231384
44	6	0	-1.843801	-0.936299	-3.783476
45	6	0	-2.091787	-0.103200	-2.689674
46	6	0	0.523230	-3.274361	-1.721366
47	6	0	-0.763322	-3.822043	-1.839195
48	6	0	-0.942877	-5.061544	-2.449701
49	6	0	0.154445	-5.760999	-2.958981
50	6	0	1.434023	-5.217608	-2.846197
51	6	0	1.622478	-3.980222	-2.225971
52	6	0	4.168633	-1.425987	-0.664998
53	8	0	5.070139	-1.022068	0.217777
54	6	0	6.420672	-1.524918	0.033211
55	6	0	2.819017	-3.034634	1.359451
56	6	0	3.929012	-2.929479	2.214091
57	6	0	4.632554	-4.062210	2.618862
58	6	0	4.228832	-5.327710	2.185911
59	6	0	3.112538	-5.446145	1.359663
60	6	0	2.410376	-4.309457	0.951929
61	8	0	-2.062200	-1.654082	0.353466
62	8	0	-6.648541	-0.387478	0.213531
63	8	0	4.388296	-2.058175	-1.679238
64	1	0	1.513009	-0.167581	-1.673558
65	1	0	2.367504	-1.548801	-2.216084
66	1	0	-1.570003	1.279818	1.724681
67	1	0	-3.944359	1.475244	1.827092
68	1	0	-4.379676	-1.051275	0.209940
69	1	0	-4.297171	1.543017	-1.164008
70	1	0	0.375001	0.184677	0.986620
71	1	0	0.158453	-2.756508	0.916792
72	1	0	-0.230294	-1.329314	-1.398385
73	1	0	2.805881	0.091712	-0.017760
74	1	0	2.164896	-1.071430	1.803815
75	1	0	-3.778250	-2.317817	1.913881
76	1	0	-4.001204	-3.565083	4.024047
77	1	0	-4.057632	-2.359518	6.197842
78	1	0	-3.920644	0.125987	6.230953
79	1	0	-3.735539	1.382019	4.111670
80	1	0	6.764231	-1.315219	-0.981181
81	1	0	7.023717	-0.998357	0.770976
82	1	0	6.427796	-2.601114	0.220214
83	1	0	-1.614898	-3.271839	-1.450934
84	1	0	-1.943187	-5.475336	-2.537120
85	1	0	0.012028	-6.723588	-3.441345
86	1	0	2.293736	-5.756021	-3.233980
87	1	0	2.634751	-3.601759	-2.117700
88	1	0	4.244645	-1.950280	2.562999
89	1	0	5.484536	-3.957736	3.284673
90	1	0	4.771805	-6.213102	2.503215
91	1	0	2.777492	-6.424940	1.029218
92	1	0	1.542299	-4.437353	0.318267
93	1	0	-5.275551	-1.086097	-1.993316
94	1	0	-4.831596	-2.548386	-3.926940
95	1	0	-2.624737	-2.475096	-5.077739
96	1	0	-0.880380	-0.892468	-4.285124
97	1	0	-1.311556	0.561618	-2.333595
98	1	0	-0.535395	2.740865	1.053887
99	1	0	-1.911093	3.044990	-2.584692

100	1	0	-3.545828	2.966173	-1.987861
101	1	0	0.221774	5.032351	0.446789
102	1	0	-1.433196	5.134062	0.999083
103	1	0	-2.894149	5.292702	-2.529587
104	1	0	-3.348375	5.117558	-0.837783
105	1	0	-1.276594	6.489438	-1.011495
106	1	0	-0.473344	5.207868	-1.907188
107	1	0	0.325482	2.974906	-2.015416

(2R,1'S)–**X$_L$X$_L$–INT2a**

Center Number	Atomic Number	Atomic Type	Coordinates (Angstroms) X	Y	Z
1	6	0	-2.512550	-5.598538	0.797822
2	6	0	-2.472128	-4.296571	1.311133
3	6	0	-1.860385	-3.294401	0.574223
4	6	0	-1.258836	-3.545847	-0.668919
5	6	0	-1.309687	-4.863803	-1.148733
6	6	0	-1.925830	-5.887158	-0.435054
7	6	0	-0.578030	-2.509154	-1.456692
8	8	0	-0.770341	-1.300902	-1.306940
9	9	0	-1.811365	-2.059899	1.121057
10	9	0	-0.750348	-5.158482	-2.327549
11	9	0	-3.021252	-4.031432	2.494750
12	9	0	-1.966843	-7.123445	-0.920539
13	9	0	-3.106638	-6.557983	1.491329
14	6	0	1.615756	-2.971394	0.444354
15	6	0	2.479476	-2.228361	-0.299597
16	6	0	3.455276	-2.897873	-1.253928
17	6	0	3.593472	-4.417226	-1.046112
18	6	0	2.259968	-5.097974	-0.738196
19	6	0	1.661608	-4.477547	0.527455
20	7	0	2.486767	-0.823457	-0.248569
21	6	0	2.217951	-0.140040	1.029862
22	6	0	3.629161	0.340359	1.450598
23	6	0	4.177905	0.863070	0.117015
24	6	0	3.476225	0.000955	-0.980994
25	6	0	1.229480	1.007241	0.793947
26	7	0	-0.095701	0.713643	0.942971
27	6	0	-1.067450	1.728109	0.539421
28	6	0	-1.030017	1.941983	-1.006718
29	7	0	-2.157990	1.040169	-1.513074
30	6	0	-3.019845	0.560281	-0.372729
31	6	0	-2.498133	1.278068	0.923963
32	6	0	3.733735	1.217584	2.680073
33	6	0	3.609291	2.613118	2.652495
34	6	0	3.700117	3.353709	3.831409
35	6	0	3.909006	2.714719	5.054697
36	6	0	4.033661	1.325152	5.093704
37	6	0	3.951273	0.586771	3.913744
38	7	0	5.673774	0.663770	0.025062
39	8	0	6.105673	-0.472947	0.215362
40	6	0	2.878996	0.820236	-2.121727
41	6	0	3.563977	1.933514	-2.625804
42	6	0	3.047342	2.652189	-3.704943
43	6	0	1.847793	2.261735	-4.304337
44	6	0	1.177699	1.136385	-3.823547
45	6	0	1.692329	0.417889	-2.741006
46	6	0	-1.124299	3.349662	-1.553196
47	6	0	0.082975	4.045922	-1.711218
48	6	0	0.080420	5.344349	-2.216773
49	6	0	-1.121404	5.957820	-2.578684
50	6	0	-2.323892	5.267327	-2.427279
51	6	0	-2.329112	3.968691	-1.913622
52	6	0	-4.471824	0.833165	-0.735595
53	8	0	-5.288514	0.161875	0.060933
54	6	0	-6.703288	0.472680	-0.061131
55	6	0	-3.418776	2.352454	1.491565
56	6	0	-4.467903	1.941145	2.329874
57	6	0	-5.362557	2.866110	2.865139
58	6	0	-5.216008	4.226019	2.580367
59	6	0	-4.163532	4.647933	1.769444
60	6	0	-3.269520	3.719251	1.231578
61	8	0	1.594693	2.128563	0.449853

62	8	0	6.350910	1.645091	-0.258060
63	8	0	-4.796525	1.570416	-1.645948
64	1	0	-1.721994	0.218432	-1.950817
65	1	0	-2.758889	1.525411	-2.195142
66	1	0	1.818550	-0.838443	1.764212
67	1	0	4.171317	-0.591213	1.650841
68	1	0	4.016449	1.925158	-0.020952
69	1	0	4.229110	-0.650968	-1.419995
70	1	0	-0.381509	-0.258191	0.930679
71	1	0	-0.784800	2.655604	1.035930
72	1	0	-0.112149	1.500351	-1.388206
73	1	0	-2.856926	-0.511037	-0.279754
74	1	0	-2.417577	0.500213	1.687908
75	1	0	3.427550	3.125454	1.715619
76	1	0	3.609627	4.435625	3.791248
77	1	0	3.981960	3.295811	5.969583
78	1	0	4.207293	0.817017	6.038031
79	1	0	4.068035	-0.494660	3.948893
80	1	0	-7.021882	0.371436	-1.099917
81	1	0	-7.209172	-0.247767	0.579280
82	1	0	-6.872529	1.493077	0.289757
83	1	0	1.016895	3.566446	-1.437485
84	1	0	1.021512	5.872568	-2.338111
85	1	0	-1.120047	6.967489	-2.978778
86	1	0	-3.263699	5.735818	-2.704012
87	1	0	-3.284106	3.467747	-1.779560
88	1	0	-4.585214	0.886398	2.563037
89	1	0	-6.162703	2.526734	3.516782
90	1	0	-5.908003	4.950080	3.000169
91	1	0	-4.027910	5.703662	1.553829
92	1	0	-2.456558	4.079778	0.614264
93	1	0	4.507285	2.242186	-2.182227
94	1	0	3.586392	3.518002	-4.078599
95	1	0	1.445392	2.824904	-5.141021
96	1	0	0.253041	0.811938	-4.296330
97	1	0	1.159760	-0.442226	-2.353347
98	1	0	0.917197	-2.475842	1.113919
99	1	0	3.150106	-2.672178	-2.286801
100	1	0	4.450582	-2.459766	-1.125200
101	1	0	0.655829	-4.871545	0.718881
102	1	0	2.260277	-4.784066	1.399559
103	1	0	4.066382	-4.854698	-1.931768
104	1	0	4.277734	-4.594389	-0.206326
105	1	0	2.403517	-6.176098	-0.605317
106	1	0	1.567695	-4.970293	-1.580365
107	1	0	0.069754	-2.876680	-2.264224

(*Si*,*Si*)–$\mathbf{X_LX_L}$**–TS1b’**

Center Number	Atomic Number	Atomic Type	Coordinates (Angstroms) X	Y	Z
1	7	0	-2.112830	1.050181	-0.656927
2	6	0	-1.183564	2.145162	-1.088214
3	6	0	-2.064940	3.445287	-1.115524
4	6	0	-3.468850	2.946374	-0.680211
5	6	0	-3.509431	1.448769	-1.034978
6	6	0	-0.014154	2.017105	-0.104556
7	8	0	-0.249367	1.946783	1.101073
8	7	0	1.202320	1.838178	-0.655790
9	6	0	2.293977	1.239259	0.126744
10	6	0	1.927923	-0.224339	0.511661
11	7	0	2.454763	-1.003353	-0.627029
12	6	0	3.462091	-0.277167	-1.405292
13	6	0	3.551857	1.163458	-0.768396
14	1	0	-2.032417	0.969411	0.367262
15	6	0	4.832272	-0.963445	-1.405366
16	8	0	5.205852	-1.788450	-0.604250
17	8	0	5.555365	-0.496333	-2.430087
18	6	0	6.944670	-0.902557	-2.453140
19	6	0	-1.557003	4.660060	-0.367427
20	6	0	-1.142419	5.779859	-1.099120
21	6	0	-0.683490	6.925313	-0.447190
22	6	0	-0.638771	6.962916	0.946765
23	6	0	-1.052633	5.851245	1.684802

24	6	0	-1.509301	4.706924	1.033627
25	6	0	4.875613	1.504699	-0.096778
26	6	0	5.869206	2.117051	-0.875940
27	6	0	7.117025	2.423941	-0.335415
28	6	0	7.389930	2.128069	1.002143
29	6	0	6.404854	1.530973	1.788494
30	6	0	5.157020	1.221033	1.244717
31	7	0	-4.559704	3.678873	-1.427440
32	8	0	-5.361060	4.311985	-0.759054
33	8	0	-4.550952	3.571788	-2.652716
34	6	0	2.397080	-0.667358	1.890698
35	6	0	1.768974	-0.076400	2.997380
36	6	0	2.142870	-0.436016	4.291132
37	6	0	3.137539	-1.397417	4.491619
38	6	0	3.752773	-1.995195	3.391481
39	6	0	3.385398	-1.635198	2.092338
40	6	0	-4.529068	0.568502	-0.360719
41	6	0	-5.227103	-0.377373	-1.121308
42	6	0	-6.130805	-1.244942	-0.507233
43	6	0	-6.343352	-1.171994	0.871088
44	6	0	-5.649782	-0.229954	1.634947
45	6	0	-4.743081	0.634452	1.023453
46	1	0	-0.868477	1.892706	-2.102483
47	1	0	-2.159128	3.711160	-2.172032
48	1	0	-3.667433	3.130588	0.374168
49	1	0	-3.578593	1.361769	-2.122846
50	1	0	1.231127	1.693493	-1.657021
51	1	0	2.446962	1.853429	1.013248
52	1	0	0.844554	-0.312608	0.495814
53	1	0	3.137286	-0.194898	-2.447494
54	1	0	3.436360	1.882061	-1.584752
55	1	0	7.346007	-0.505827	-3.384818
56	1	0	7.460518	-0.469973	-1.592405
57	1	0	7.021158	-1.991388	-2.424590
58	1	0	-1.187617	5.761723	-2.185869
59	1	0	-0.370369	7.787631	-1.028451
60	1	0	-0.287288	7.854872	1.457180
61	1	0	-1.021008	5.874320	2.770210
62	1	0	-1.803088	3.842350	1.619572
63	1	0	5.666862	2.346514	-1.919071
64	1	0	7.870072	2.902398	-0.955268
65	1	0	8.359714	2.368422	1.428115
66	1	0	6.602474	1.300438	2.831282
67	1	0	4.413472	0.753053	1.876806
68	1	0	0.991082	0.665607	2.835492
69	1	0	1.655359	0.030622	5.142570
70	1	0	3.428380	-1.678503	5.499814
71	1	0	4.527628	-2.742208	3.539020
72	1	0	3.889828	-2.081131	1.243488
73	1	0	-5.067342	-0.432416	-2.195375
74	1	0	-6.670036	-1.973516	-1.105169
75	1	0	-7.042222	-1.850357	1.350253
76	1	0	-5.807171	-0.174299	2.707097
77	1	0	-4.207841	1.357324	1.635243
78	6	0	2.025307	-2.249108	-0.919774
79	6	0	1.023799	-2.878036	-0.171571
80	6	0	2.515379	-2.916478	-2.188150
81	6	0	0.860224	-4.388422	-0.217010
82	1	0	0.777585	-2.436070	0.787967
83	6	0	2.551403	-4.449101	-2.074312
84	1	0	1.824445	-2.615857	-2.992516
85	1	0	3.499802	-2.557407	-2.486953
86	6	0	1.216779	-5.005264	-1.574561
87	1	0	-0.162101	-4.659653	0.071644
88	1	0	1.508497	-4.819869	0.560107
89	1	0	2.812358	-4.872382	-3.050200
90	1	0	3.353542	-4.728589	-1.379079
91	1	0	1.267599	-6.095778	-1.484399
92	1	0	0.425288	-4.791429	-2.304451
93	6	0	-0.640652	-2.043710	-1.360698
94	1	0	-0.405641	-2.720504	-2.188018
95	8	0	-0.501427	-0.790315	-1.542822
96	6	0	-1.723603	-2.557535	-0.462935
97	6	0	-2.048836	-1.962544	0.760500
98	6	0	-2.505722	-3.649132	-0.854792
99	6	0	-3.096141	-2.409301	1.552834
100	9	0	-1.317389	-0.922167	1.227635

101	6	0	-3.579832	-4.105992	-0.093436
102	9	0	-2.235731	-4.278732	-2.009512
103	6	0	-3.880743	-3.475028	1.111739
104	9	0	-3.369712	-1.812425	2.715007
105	9	0	-4.326815	-5.124717	-0.517873
106	9	0	-4.919037	-3.878899	1.839659
107	1	0	-1.705303	0.137775	-1.048919

(2R,1'S)–**X_LX_L–INT2b'**

Center Number	Atomic Number	Atomic Type	Coordinates (Angstroms) X	Y	Z
1	7	0	-2.158340	0.944761	-0.767073
2	6	0	-1.198852	2.024505	-1.101524
3	6	0	-2.006561	3.382369	-1.118381
4	6	0	-3.397193	2.951355	-0.613643
5	6	0	-3.513579	1.469506	-1.041740
6	6	0	-0.055670	1.895334	-0.095100
7	8	0	-0.282713	1.723744	1.101030
8	7	0	1.206717	1.851799	-0.604407
9	6	0	2.282088	1.249903	0.191538
10	6	0	1.917667	-0.232557	0.483142
11	7	0	2.450108	-0.934448	-0.722679
12	6	0	3.490225	-0.136409	-1.416489
13	6	0	3.588407	1.228440	-0.634602
14	1	0	-2.078184	0.786801	0.240665
15	6	0	4.812059	-0.902135	-1.509598
16	8	0	5.081368	-1.900134	-0.880366
17	8	0	5.594355	-0.308299	-2.408177
18	6	0	6.955243	-0.805250	-2.495101
19	6	0	-1.387000	4.571731	-0.417216
20	6	0	-0.911892	5.643733	-1.183351
21	6	0	-0.333976	6.757911	-0.573219
22	6	0	-0.226621	6.814201	0.816608
23	6	0	-0.699690	5.751786	1.590388
24	6	0	-1.275781	4.639054	0.979834
25	6	0	4.886820	1.453956	0.131916
26	6	0	5.963609	2.027429	-0.563754
27	6	0	7.190774	2.239264	0.061874
28	6	0	7.359842	1.889650	1.403727
29	6	0	6.290947	1.339519	2.109670
30	6	0	5.062698	1.124319	1.480829
31	7	0	-4.493443	3.774932	-1.238202
32	8	0	-5.272438	4.336448	-0.480503
33	8	0	-4.537215	3.799645	-2.468125
34	6	0	2.371145	-0.816742	1.806978
35	6	0	1.732528	-0.323119	2.954703
36	6	0	2.083231	-0.813896	4.211052
37	6	0	3.059665	-1.807467	4.330592
38	6	0	3.682451	-2.307896	3.187242
39	6	0	3.341427	-1.816632	1.924239
40	6	0	-4.580600	0.643035	-0.364689
41	6	0	-5.427530	-0.163423	-1.133082
42	6	0	-6.395734	-0.961133	-0.521151
43	6	0	-6.527644	-0.957553	0.868588
44	6	0	-5.683828	-0.158697	1.643754
45	6	0	-4.713041	0.633004	1.030883
46	1	0	-0.815136	1.843393	-2.111576
47	1	0	-2.139996	3.639507	-2.173136
48	1	0	-3.516967	3.080856	0.460591
49	1	0	-3.670014	1.459849	-2.125638
50	1	0	1.298564	1.837798	-1.611579
51	1	0	2.383998	1.812051	1.119177
52	1	0	0.838231	-0.317122	0.433492
53	1	0	3.144475	0.057507	-2.434998
54	1	0	3.534226	2.023952	-1.383191
55	1	0	7.400640	-0.273541	-3.334354
56	1	0	7.479475	-0.574486	-1.564995
57	1	0	6.952069	-1.883059	-2.667629
58	1	0	-1.007321	5.612843	-2.266509
59	1	0	0.021546	7.583336	-1.183360
60	1	0	0.216155	7.682880	1.295367
61	1	0	-0.624439	5.790507	2.673394
62	1	0	-1.621132	3.813117	1.592924

63	1	0	5.840389	2.305305	-1.607002
64	1	0	8.007986	2.690262	-0.493773
65	1	0	8.313043	2.057566	1.896335
66	1	0	6.404662	1.075783	3.156998
67	1	0	4.251518	0.702842	2.059429
68	1	0	0.964979	0.439308	2.851108
69	1	0	1.588096	-0.426112	5.096533
70	1	0	3.329896	-2.189829	5.310542
71	1	0	4.443875	-3.077797	3.272623
72	1	0	3.856817	-2.189621	1.046436
73	1	0	-5.332800	-0.161796	-2.216119
74	1	0	-7.049783	-1.578775	-1.129787
75	1	0	-7.280164	-1.576824	1.346756
76	1	0	-5.774900	-0.158309	2.725301
77	1	0	-4.060099	1.243673	1.650527
78	6	0	1.937523	-2.032014	-1.194539
79	6	0	0.813358	-2.729175	-0.472462
80	6	0	2.366554	-2.634939	-2.497010
81	6	0	1.049346	-4.257397	-0.404139
82	1	0	0.747914	-2.358405	0.548079
83	6	0	2.666631	-4.144466	-2.334911
84	1	0	1.511254	-2.509247	-3.177823
85	1	0	3.212973	-2.121396	-2.950916
86	6	0	1.461890	-4.881348	-1.742158
87	1	0	0.135449	-4.722074	-0.017311
88	1	0	1.833044	-4.439652	0.341474
89	1	0	2.933521	-4.548844	-3.316244
90	1	0	3.544593	-4.256385	-1.686956
91	1	0	1.707440	-5.937762	-1.590255
92	1	0	0.624116	-4.856660	-2.451235
93	6	0	-0.515406	-2.331885	-1.212238
94	1	0	-0.586136	-2.925442	-2.129952
95	8	0	-0.412730	-0.959893	-1.550776
96	6	0	-1.729236	-2.658915	-0.368190
97	6	0	-1.989505	-2.028701	0.851622
98	6	0	-2.669557	-3.596610	-0.802195
99	6	0	-3.118734	-2.306822	1.609710
100	9	0	-1.132082	-1.099545	1.329743
101	6	0	-3.821821	-3.885385	-0.073269
102	9	0	-2.474067	-4.252694	-1.958297
103	6	0	-4.044183	-3.237779	1.139756
104	9	0	-3.325152	-1.680237	2.769159
105	9	0	-4.704145	-4.775086	-0.526738
106	9	0	-5.137522	-3.503205	1.848336
107	1	0	-1.262435	-0.460405	-1.380723

(*Si*,*Re*)–$\mathbf{X_LX_L}$**–TS1a**

Center Number	Atomic Number	Atomic Type	Coordinates (Angstroms) X	Y	Z
1	6	0	5.609902	-0.588642	0.275120
2	6	0	4.889084	0.043478	-0.745974
3	6	0	5.510101	1.073866	-1.464125
4	6	0	6.815684	1.467616	-1.169852
5	6	0	7.525947	0.828574	-0.152809
6	6	0	6.918398	-0.201908	0.566182
7	6	0	3.447809	-0.293772	-1.099684
8	6	0	2.396677	0.296938	-0.115326
9	6	0	1.991355	-0.856643	0.840957
10	7	0	2.048821	-2.050759	-0.089523
11	6	0	3.039312	-1.800190	-1.200647
12	7	0	1.249007	0.788048	-0.879426
13	6	0	0.228548	1.406594	-0.232001
14	8	0	0.243028	1.636155	0.975788
15	6	0	2.785280	-0.972040	2.127486
16	6	0	2.716171	0.133619	2.989886
17	6	0	3.377948	0.114186	4.215015
18	6	0	4.106719	-1.013188	4.603235
19	6	0	4.158730	-2.121957	3.760810
20	6	0	3.497601	-2.106245	2.528978
21	6	0	4.148511	-2.840868	-1.131071
22	8	0	4.186526	-3.716434	-0.287564
23	6	0	-0.904011	1.916689	-1.141451
24	7	0	-2.213847	1.306055	-0.832911

25	6	0	-3.001358	2.200738	0.095840
26	6	0	-2.045126	3.385928	0.339052
27	6	0	-1.151951	3.428638	-0.905065
28	6	0	-2.786444	0.357966	-1.599225
29	6	0	-2.116380	-0.314272	-2.661610
30	6	0	-2.957499	-0.725284	-3.877267
31	6	0	-4.367689	-1.215884	-3.539715
32	6	0	-5.048301	-0.243737	-2.578311
33	6	0	-4.211363	-0.056258	-1.299919
34	6	0	0.094110	4.288746	-0.885710
35	6	0	0.796803	4.593614	0.286859
36	6	0	1.966073	5.353211	0.228594
37	6	0	2.450574	5.811764	-0.997316
38	6	0	1.754178	5.515487	-2.170651
39	6	0	0.582472	4.762499	-2.111387
40	7	0	-2.818030	4.677839	0.474805
41	8	0	-2.518742	5.398864	1.414973
42	6	0	-3.524913	1.578926	1.371771
43	6	0	-4.859662	1.811260	1.728276
44	6	0	-5.370736	1.302534	2.922643
45	6	0	-4.550227	0.552896	3.766872
46	6	0	-3.216309	0.327798	3.419034
47	6	0	-2.700259	0.843508	2.230469
48	8	0	-3.664216	4.921754	-0.384412
49	8	0	5.006459	-2.666071	-2.121778
50	6	0	6.182564	-3.516067	-2.114274
51	6	0	-1.144481	-1.996877	-2.002622
52	8	0	0.060336	-1.648749	-1.719456
53	6	0	-1.997272	-2.626829	-0.936649
54	6	0	-2.902737	-3.647334	-1.245376
55	6	0	-3.693499	-4.265133	-0.278676
56	6	0	-3.567646	-3.882553	1.056142
57	6	0	-2.663485	-2.878002	1.404278
58	6	0	-1.903828	-2.270768	0.411557
59	9	0	-4.305033	-4.468971	1.994024
60	9	0	-4.549518	-5.227171	-0.619739
61	9	0	-3.046624	-4.050067	-2.519802
62	9	0	-1.071496	-1.276644	0.786206
63	9	0	-2.529602	-2.513712	2.681213
64	1	0	1.096992	-2.103146	-0.565176
65	1	0	2.279337	-2.934206	0.380581
66	1	0	-0.646048	1.745741	-2.185901
67	1	0	-1.793725	3.759700	-1.731447
68	1	0	-1.488378	3.270643	1.262534
69	1	0	-3.838709	2.593989	-0.487013
70	1	0	0.987442	0.249250	-1.702508
71	1	0	2.803446	1.135451	0.448350
72	1	0	0.945650	-0.741116	1.108890
73	1	0	2.489206	-1.965277	-2.129981
74	1	0	3.262443	0.153946	-2.079037
75	1	0	0.439590	4.243516	1.248104
76	1	0	2.494530	5.592024	1.147042
77	1	0	3.358204	6.407138	-1.037134
78	1	0	2.114249	5.880389	-3.128321
79	1	0	0.034210	4.548031	-3.026778
80	1	0	5.889017	-4.566297	-2.065582
81	1	0	6.701230	-3.292919	-3.044872
82	1	0	6.802053	-3.257205	-1.252411
83	1	0	2.124644	0.998917	2.701897
84	1	0	3.319084	0.978377	4.870189
85	1	0	4.622802	-1.027958	5.558549
86	1	0	4.715830	-3.006864	4.054207
87	1	0	3.589293	-2.978032	1.888889
88	1	0	4.968971	1.574678	-2.263848
89	1	0	7.277699	2.268186	-1.740233
90	1	0	8.543733	1.130051	0.076671
91	1	0	7.457633	-0.704903	1.363858
92	1	0	5.162352	-1.383035	0.858167
93	1	0	-5.500541	2.397862	1.073079
94	1	0	-6.406059	1.491522	3.190392
95	1	0	-4.946342	0.150887	4.694826
96	1	0	-2.573282	-0.250966	4.074268
97	1	0	-1.659849	0.690366	1.975647
98	1	0	-1.165166	0.111829	-2.965821
99	1	0	-4.156378	-1.011508	-0.758528
100	1	0	-4.708392	0.634746	-0.624343
101	1	0	-2.415757	-1.485799	-4.453007

102	1	0	-3.034969	0.149857	-4.538692
103	1	0	-6.044445	-0.599635	-2.294694
104	1	0	-5.185956	0.731092	-3.064919
105	1	0	-4.950830	-1.314820	-4.461829
106	1	0	-4.322650	-2.212417	-3.090874
107	1	0	-1.322772	-2.451176	-2.985410

(2R,1'R)–X_LX_L–**INT2a**

Center Number	Atomic Number	Atomic Type	Coordinates (Angstroms) X	Y	Z
1	6	0	5.439709	0.029977	0.490590
2	6	0	4.765154	0.610021	-0.592388
3	6	0	5.390352	1.651405	-1.289499
4	6	0	6.655251	2.107349	-0.916192
5	6	0	7.319554	1.520843	0.161508
6	6	0	6.707128	0.479656	0.861505
7	6	0	3.377588	0.177511	-1.039626
8	6	0	2.234927	0.544456	-0.045861
9	6	0	1.877573	-0.761851	0.716355
10	7	0	2.098590	-1.804712	-0.347521
11	6	0	3.149590	-1.348279	-1.317156
12	7	0	1.080590	1.031529	-0.809327
13	6	0	0.046095	1.600714	-0.152665
14	8	0	0.039861	1.865023	1.048856
15	6	0	2.573005	-0.969792	2.048365
16	6	0	2.331408	0.005548	3.029329
17	6	0	2.890378	-0.115855	4.298997
18	6	0	3.688418	-1.219349	4.613393
19	6	0	3.914612	-2.200090	3.649760
20	6	0	3.356798	-2.081057	2.372786
21	6	0	4.369463	-2.255720	-1.246807
22	8	0	4.461663	-3.203551	-0.491040
23	6	0	-1.141636	1.989295	-1.067144
24	7	0	-2.363691	1.185346	-0.769914
25	6	0	-3.235670	1.933432	0.237918
26	6	0	-2.473794	3.255029	0.451764
27	6	0	-1.627313	3.435574	-0.815051
28	6	0	-2.752334	0.118693	-1.427342
29	6	0	-1.969004	-0.474960	-2.563966
30	6	0	-2.911150	-0.749514	-3.769541
31	6	0	-4.158256	-1.557810	-3.417559
32	6	0	-4.940801	-0.825937	-2.328263
33	6	0	-4.060211	-0.548067	-1.088362
34	6	0	-0.550984	4.500425	-0.825078
35	6	0	0.191564	4.847824	0.311146
36	6	0	1.186019	5.822614	0.227862
37	6	0	1.454366	6.457127	-0.986147
38	6	0	0.717647	6.118012	-2.122251
39	6	0	-0.280761	5.148513	-2.038255
40	7	0	-3.444493	4.407809	0.590286
41	8	0	-3.220998	5.204035	1.487977
42	6	0	-3.590590	1.201582	1.513694
43	6	0	-4.936764	1.172041	1.904943
44	6	0	-5.314575	0.531294	3.084786
45	6	0	-4.350015	-0.095101	3.876108
46	6	0	-3.006540	-0.054678	3.496717
47	6	0	-2.622684	0.604707	2.329503
48	8	0	-4.358688	4.475809	-0.231233
49	8	0	5.270994	-1.886543	-2.146164
50	6	0	6.533719	-2.596096	-2.115226
51	6	0	-1.024960	-1.744819	-2.212216
52	8	0	0.218836	-1.292841	-1.877676
53	6	0	-1.595908	-2.716643	-1.174967
54	6	0	-2.072238	-3.980590	-1.525143
55	6	0	-2.506900	-4.907722	-0.576491
56	6	0	-2.458726	-4.581312	0.777996
57	6	0	-1.997586	-3.322570	1.167134
58	6	0	-1.573947	-2.431149	0.189930
59	9	0	-2.870157	-5.453938	1.693830
60	9	0	-2.960164	-6.101328	-0.957513
61	9	0	-2.162498	-4.326005	-2.821386
62	9	0	-1.192402	-1.197730	0.596874
63	9	0	-1.973412	-2.978574	2.457464

64	1	0	1.127877	-1.808892	-0.976421
65	1	0	2.342366	-2.726871	0.024674
66	1	0	-0.859456	1.865030	-2.109880
67	1	0	-2.336955	3.656735	-1.622920
68	1	0	-1.884710	3.243321	1.362192
69	1	0	-4.149399	2.182679	-0.307336
70	1	0	0.794686	0.403765	-1.577774
71	1	0	2.537766	1.330249	0.643734
72	1	0	0.808349	-0.762296	0.910753
73	1	0	2.713170	-1.469331	-2.312498
74	1	0	3.172738	0.708481	-1.971747
75	1	0	0.003947	4.359774	1.259881
76	1	0	1.748010	6.089618	1.118066
77	1	0	2.226486	7.218789	-1.044257
78	1	0	0.910177	6.614653	-3.068747
79	1	0	-0.864225	4.900718	-2.922830
80	1	0	6.366878	-3.670245	-2.218098
81	1	0	7.102952	-2.203317	-2.956049
82	1	0	7.041192	-2.385065	-1.170843
83	1	0	1.694408	0.854785	2.793269
84	1	0	2.699616	0.650115	5.045204
85	1	0	4.125183	-1.313935	5.603179
86	1	0	4.529874	-3.064366	3.882623
87	1	0	3.583190	-2.845780	1.637421
88	1	0	4.885763	2.109658	-2.137049
89	1	0	7.121387	2.916222	-1.471538
90	1	0	8.305387	1.871207	0.453121
91	1	0	7.211143	0.016547	1.705171
92	1	0	4.986629	-0.775681	1.054409
93	1	0	-5.691721	1.654744	1.287500
94	1	0	-6.359261	0.518694	3.380637
95	1	0	-4.643377	-0.606054	4.788398
96	1	0	-2.250933	-0.533172	4.112299
97	1	0	-1.577157	0.662348	2.059293
98	1	0	-1.216946	0.230154	-2.915940
99	1	0	-3.800221	-1.509200	-0.627602
100	1	0	-4.621503	-0.000115	-0.337675
101	1	0	-2.317524	-1.248629	-4.543029
102	1	0	-3.220817	0.216294	-4.191834
103	1	0	-5.811346	-1.402446	-1.999557
104	1	0	-5.320283	0.126544	-2.720823
105	1	0	-4.780997	-1.682688	-4.310020
106	1	0	-3.879902	-2.564529	-3.086496
107	1	0	-1.016906	-2.293708	-3.168056

(*Si*,*Re*)–$\mathbf{X_LX_L}$–**TS1b'**

Center Number	Atomic Number	Atomic Type	Coordinates (Angstroms) X	Y	Z
1	6	0	1.051063	4.636396	-1.685855
2	6	0	1.113054	5.116388	-0.377825
3	6	0	0.944255	4.234120	0.688843
4	6	0	0.700607	2.868856	0.503534
5	6	0	0.625861	2.436081	-0.823209
6	6	0	0.794229	3.282457	-1.910474
7	6	0	0.609827	1.933421	1.671974
8	8	0	1.102914	0.761035	1.583143
9	6	0	-1.477313	1.692580	2.148631
10	6	0	-2.157517	1.586780	0.920221
11	6	0	-2.666210	2.859287	0.273841
12	6	0	-3.288000	3.814709	1.306092
13	6	0	-2.291435	4.115692	2.427197
14	6	0	-1.842008	2.819407	3.109765
15	7	0	-2.308392	0.410168	0.278149
16	6	0	-1.754093	-0.865642	0.787905
17	6	0	-1.722194	-1.763236	-0.485251
18	6	0	-2.857973	-1.259743	-1.403894
19	6	0	-2.994200	0.261087	-1.008780
20	7	0	-0.438811	-1.609360	-1.185249
21	6	0	0.713249	-1.968328	-0.569868
22	8	0	0.768294	-2.669402	0.434723
23	6	0	-2.497779	-1.476419	1.967281
24	6	0	-1.907180	-2.591773	2.580504
25	6	0	-2.538100	-3.217064	3.654616

26	6	0	-3.756767	-2.727667	4.133435
27	6	0	-4.337557	-1.611195	3.531610
28	6	0	-3.712720	-0.983599	2.450772
29	6	0	-4.462500	0.695469	-0.943286
30	8	0	-4.887086	1.015088	-2.172466
31	6	0	-6.301529	1.303452	-2.287647
32	6	0	-4.156666	-2.059266	-1.378128
33	6	0	-4.981679	-2.000384	-2.513426
34	6	0	-6.203919	-2.669823	-2.548284
35	6	0	-6.617180	-3.425875	-1.448724
36	6	0	-5.794630	-3.510082	-0.325887
37	6	0	-4.574076	-2.833010	-0.289095
38	6	0	1.987462	-1.304687	-1.105514
39	7	0	2.621616	-0.676577	0.090811
40	6	0	3.968855	-0.187197	-0.351945
41	6	0	4.358635	-1.188987	-1.484183
42	6	0	3.154029	-2.157383	-1.670652
43	6	0	3.349451	-3.485665	-0.960112
44	6	0	2.702013	-4.617166	-1.471753
45	6	0	2.799904	-5.846194	-0.822802
46	6	0	3.550073	-5.961366	0.349483
47	6	0	4.203951	-4.842378	0.864066
48	6	0	4.105881	-3.611167	0.213763
49	7	0	4.606753	-0.400700	-2.751385
50	8	0	3.643701	0.205783	-3.225961
51	6	0	4.941715	-0.037230	0.798171
52	6	0	6.319971	-0.045927	0.544888
53	6	0	7.224353	0.138695	1.590203
54	6	0	6.761371	0.335291	2.892952
55	6	0	5.389312	0.362065	3.144716
56	6	0	4.477433	0.184508	2.102967
57	8	0	-5.132612	0.748534	0.061442
58	8	0	5.742043	-0.408089	-3.202209
59	1	0	1.989129	0.071687	0.554925
60	1	0	2.708026	-1.417637	0.802384
61	1	0	1.772533	-0.500468	-1.807797
62	1	0	2.979899	-2.334256	-2.735028
63	1	0	5.287187	-1.718334	-1.291687
64	1	0	3.771190	0.785999	-0.813712
65	1	0	-0.383848	-0.890284	-1.894331
66	1	0	-1.832477	-2.813942	-0.220412
67	1	0	-0.729248	-0.680319	1.103068
68	1	0	-2.496421	0.876560	-1.765415
69	1	0	-2.506536	-1.288966	-2.439703
70	1	0	-6.450597	1.608569	-3.322613
71	1	0	-6.875243	0.401847	-2.059568
72	1	0	-6.580287	2.103533	-1.598569
73	1	0	2.115356	-4.535750	-2.384163
74	1	0	2.295124	-6.714732	-1.235409
75	1	0	3.630016	-6.919591	0.853939
76	1	0	4.797046	-4.923667	1.770178
77	1	0	4.641976	-2.762187	0.632661
78	1	0	-4.667657	-1.415630	-3.374119
79	1	0	-6.825249	-2.611425	-3.437852
80	1	0	-7.567112	-3.951945	-1.472627
81	1	0	-6.098798	-4.102432	0.532349
82	1	0	-3.962933	-2.913965	0.600503
83	1	0	-0.957483	-2.966471	2.206230
84	1	0	-2.076361	-4.083211	4.120383
85	1	0	-4.248162	-3.214884	4.970803
86	1	0	-5.286556	-1.227494	3.895330
87	1	0	-4.190506	-0.141112	1.965035
88	1	0	6.692476	-0.179346	-0.467725
89	1	0	8.290344	0.128706	1.384465
90	1	0	7.467152	0.474929	3.706236
91	1	0	5.021732	0.526979	4.153191
92	1	0	3.413224	0.245880	2.317967
93	1	0	-1.258753	0.759792	2.658178
94	1	0	-1.803311	3.353412	-0.194769
95	1	0	-3.375531	2.670832	-0.529874
96	1	0	-1.001937	3.017740	3.787896
97	1	0	-2.656282	2.448411	3.749533
98	1	0	-3.597602	4.734678	0.798385
99	1	0	-4.194342	3.350220	1.714984
100	1	0	-2.740546	4.784975	3.169084
101	1	0	-1.422417	4.643243	2.017708
102	1	0	0.680461	2.449151	2.635487

103	9	0	1.003733	4.736534	1.931244
104	9	0	0.317154	1.141948	-1.093569
105	9	0	1.347741	6.407402	-0.154237
106	9	0	0.698781	2.807835	-3.154649
107	9	0	1.215607	5.463265	-2.712811

(2R,1'R)–**X_LX_L–INT2b'**

Center Number	Atomic Number	Atomic Type	Coordinates (Angstroms) X	Y	Z
1	6	0	4.513345	-0.208676	2.061732
2	6	0	4.836770	-0.479723	0.725449
3	6	0	6.185173	-0.593455	0.357832
4	6	0	7.190112	-0.461765	1.315096
5	6	0	6.861646	-0.210659	2.649318
6	6	0	5.522718	-0.080627	3.018224
7	6	0	3.758319	-0.587955	-0.333045
8	7	0	2.412234	-0.823747	0.216796
9	6	0	1.656256	-1.453869	-0.877273
10	6	0	2.641692	-2.505837	-1.479873
11	6	0	3.974909	-1.721060	-1.410901
12	6	0	0.328144	-1.932759	-0.293896
13	8	0	0.267012	-2.510941	0.783773
14	7	0	4.283141	-1.051520	-2.726387
15	8	0	5.435913	-1.110979	-3.134484
16	6	0	2.678203	-3.812667	-0.711458
17	6	0	1.875227	-4.873020	-1.151695
18	6	0	1.828568	-6.074286	-0.446808
19	6	0	2.588563	-6.234304	0.713648
20	6	0	3.395680	-5.187675	1.158908
21	6	0	3.441674	-3.985150	0.451814
22	7	0	-0.788694	-1.579643	-1.002105
23	6	0	-2.095521	-1.552054	-0.338764
24	6	0	-2.064812	-0.524645	0.828407
25	7	0	-2.377535	0.759625	0.121225
26	6	0	-3.065541	0.518411	-1.172976
27	6	0	-3.156461	-1.044541	-1.340978
28	6	0	-1.949070	1.930288	0.496634
29	6	0	-1.208973	2.108021	1.801341
30	6	0	-1.667134	3.400819	2.525298
31	6	0	-1.722080	4.641517	1.633679
32	6	0	-2.635147	4.372749	0.436323
33	6	0	-2.130866	3.151548	-0.362146
34	6	0	-2.950729	-0.819995	2.021917
35	6	0	-2.571796	-1.916624	2.811526
36	6	0	-3.332565	-2.267742	3.924971
37	6	0	-4.463664	-1.520079	4.267356
38	6	0	-4.827644	-0.419359	3.491972
39	6	0	-4.074800	-0.065392	2.369005
40	6	0	-4.556379	-1.641068	-1.239574
41	6	0	-5.350686	-1.629790	-2.398559
42	6	0	-6.649363	-2.135062	-2.384546
43	6	0	-7.174916	-2.676799	-1.209108
44	6	0	-6.386031	-2.717926	-0.060679
45	6	0	-5.086308	-2.206820	-0.074390
46	6	0	-4.417553	1.233099	-1.228302
47	8	0	-5.013581	1.666264	-0.268827
48	8	0	-4.810737	1.321199	-2.498346
49	6	0	-6.157323	1.817960	-2.713101
50	8	0	3.362261	-0.444097	-3.278344
51	6	0	0.361066	1.968075	1.674113
52	8	0	0.632981	0.585878	1.693174
53	6	0	1.082645	2.661009	0.526475
54	6	0	1.953063	3.726556	0.770391
55	6	0	2.780469	4.257580	-0.219912
56	6	0	2.759510	3.711264	-1.502875
57	6	0	1.888022	2.658360	-1.790625
58	6	0	1.071785	2.167927	-0.780431
59	9	0	3.544292	4.204311	-2.453266
60	9	0	3.594882	5.272440	0.057395
61	9	0	2.004801	4.275030	1.992663
62	9	0	0.202685	1.177613	-1.111364
63	9	0	1.831072	2.135362	-3.014936
64	1	0	1.403574	0.306925	1.121691

65	1	0	2.459567	-1.519090	0.968324
66	1	0	1.490254	-0.697172	-1.646023
67	1	0	2.375974	-2.703812	-2.521717
68	1	0	4.832515	-2.356318	-1.206237
69	1	0	3.701060	0.359931	-0.882904
70	1	0	-0.652078	-0.961687	-1.790675
71	1	0	-2.316329	-2.550098	0.038366
72	1	0	-1.041279	-0.442952	1.186595
73	1	0	-2.432873	0.924545	-1.966037
74	1	0	-2.805621	-1.268496	-2.352509
75	1	0	-6.258637	1.911817	-3.793124
76	1	0	-6.872124	1.093941	-2.315378
77	1	0	-6.285666	2.782854	-2.219143
78	1	0	1.281594	-4.756424	-2.055941
79	1	0	1.205129	-6.887403	-0.807626
80	1	0	2.557504	-7.171315	1.261912
81	1	0	3.996685	-5.304784	2.056080
82	1	0	4.090871	-3.192342	0.815830
83	1	0	-4.947781	-1.218024	-3.319795
84	1	0	-7.242658	-2.121093	-3.294445
85	1	0	-8.184780	-3.076084	-1.194768
86	1	0	-6.775834	-3.150701	0.855908
87	1	0	-4.499347	-2.262393	0.832291
88	1	0	-1.682953	-2.483586	2.544187
89	1	0	-3.037540	-3.119812	4.530401
90	1	0	-5.054271	-1.794117	5.136635
91	1	0	-5.706065	0.164582	3.751278
92	1	0	-4.389300	0.768976	1.752432
93	1	0	6.452697	-0.776927	-0.679933
94	1	0	8.230213	-0.556353	1.017190
95	1	0	7.645322	-0.112284	3.394719
96	1	0	5.258645	0.122298	4.052183
97	1	0	3.477027	-0.082563	2.362485
98	1	0	-1.460504	1.276296	2.462679
99	1	0	-1.130564	3.392720	-0.746371
100	1	0	-2.764897	2.973437	-1.228424
101	1	0	-1.006781	3.557380	3.385094
102	1	0	-2.668204	3.211856	2.933522
103	1	0	-2.671501	5.231377	-0.241523
104	1	0	-3.661313	4.178984	0.771772
105	1	0	-2.095606	5.493813	2.210980
106	1	0	-0.714552	4.910025	1.289582
107	1	0	0.736861	2.426872	2.596665

(*Re*,*Si*)–**X_LX_L–TS1a'**

Center Number	Atomic Number	Atomic Type	Coordinates (Angstroms) X	Y	Z
1	6	0	4.188084	1.421208	-0.806329
2	6	0	3.737129	0.279159	-0.128024
3	6	0	4.697642	-0.430100	0.605752
4	6	0	6.028457	-0.042279	0.683814
5	6	0	6.441428	1.105773	0.005861
6	6	0	5.518004	1.836605	-0.738498
7	6	0	2.310741	-0.181835	-0.145372
8	6	0	2.454213	-1.958535	-1.127048
9	6	0	1.113914	-2.001159	-1.595641
10	6	0	0.931598	-1.342664	-2.933845
11	6	0	1.773372	-2.062187	-4.029857
12	6	0	3.079143	-2.656854	-3.463037
13	6	0	3.521294	-1.870138	-2.219174
14	7	0	0.079013	-2.451189	-0.877969
15	6	0	-1.341089	-2.342527	-1.267744
16	6	0	-1.897057	-3.727757	-0.890725
17	6	0	-1.213724	-3.964435	0.458457
18	6	0	0.201019	-3.337550	0.334807
19	6	0	-1.984051	-1.135653	-0.532423
20	8	0	-2.907017	-1.265737	0.261578
21	6	0	0.657711	-2.662688	1.615237
22	6	0	1.873295	-3.034590	2.196653
23	6	0	2.320965	-2.418645	3.367621
24	6	0	1.549655	-1.428913	3.975908
25	6	0	0.314667	-1.075588	3.423353
26	6	0	-0.131299	-1.694955	2.256867

27	7	0	-1.098473	-5.443976	0.762216
28	8	0	-0.410996	-6.116404	-0.002371
29	6	0	-3.400843	-3.895531	-0.987998
30	6	0	-4.199002	-4.271600	0.096185
31	6	0	-5.577152	-4.424336	-0.064322
32	6	0	-6.173330	-4.201343	-1.305669
33	6	0	-5.380776	-3.837077	-2.396252
34	6	0	-4.003872	-3.692371	-2.237152
35	9	0	4.332833	-1.556654	1.247758
36	9	0	6.904905	-0.754352	1.393550
37	9	0	7.709971	1.498145	0.068936
38	9	0	5.908692	2.935573	-1.387163
39	9	0	3.360685	2.179640	-1.542208
40	7	0	-1.427385	0.067255	-0.869841
41	6	0	-1.852177	1.287623	-0.190571
42	6	0	-1.730160	2.512637	-1.150933
43	6	0	-0.357009	3.170265	-0.781607
44	7	0	0.121914	2.488216	0.469856
45	6	0	-0.948904	1.585344	1.042368
46	6	0	-2.924309	3.453012	-1.143977
47	6	0	-3.389451	4.069041	0.026303
48	6	0	-4.468988	4.952444	-0.019932
49	6	0	-5.101325	5.229958	-1.233751
50	6	0	-4.647903	4.616674	-2.402906
51	6	0	-3.565873	3.737174	-2.355723
52	6	0	-1.657866	2.084576	2.286421
53	6	0	-2.630078	1.225026	2.826176
54	6	0	-3.293332	1.561360	4.003871
55	6	0	-2.996318	2.758789	4.660918
56	6	0	-2.036262	3.616163	4.127300
57	6	0	-1.365061	3.282178	2.946640
58	6	0	-0.377634	4.675386	-0.541636
59	8	0	-0.695222	5.317962	-1.652339
60	6	0	-0.915355	6.746899	-1.527830
61	8	0	-0.114050	5.178837	0.533715
62	8	0	-1.706179	-5.859572	1.739142
63	8	0	1.424799	0.602190	-0.689635
64	1	0	0.895152	1.820731	0.149132
65	1	0	0.465822	3.193635	1.131551
66	1	0	-1.436813	-2.178281	-2.339011
67	1	0	-1.420711	-4.423767	-1.594974
68	1	0	-1.777126	-3.539704	1.284411
69	1	0	0.908108	-4.125662	0.063400
70	1	0	-0.441515	0.070201	-1.141872
71	1	0	-2.884959	1.141642	0.119725
72	1	0	-0.417814	0.669612	1.307506
73	1	0	0.387076	2.954693	-1.552695
74	1	0	-1.637131	2.114889	-2.163289
75	1	0	-3.758717	-4.465355	1.068592
76	1	0	-6.183653	-4.721498	0.786499
77	1	0	-7.246046	-4.320850	-1.426365
78	1	0	-5.831923	-3.674677	-3.371248
79	1	0	-3.392960	-3.428414	-3.099162
80	1	0	-0.031833	7.228907	-1.105001
81	1	0	-1.107917	7.094379	-2.541223
82	1	0	-1.782658	6.917635	-0.885767
83	1	0	-2.877809	0.298125	2.313369
84	1	0	-4.045015	0.888685	4.406159
85	1	0	-3.514343	3.021691	5.578326
86	1	0	-1.804633	4.554425	4.622650
87	1	0	-0.655776	3.995925	2.541039
88	1	0	-3.212394	3.272898	-3.273481
89	1	0	-5.134418	4.822157	-3.352286
90	1	0	-5.943327	5.915184	-1.267217
91	1	0	-4.817393	5.418393	0.897548
92	1	0	-2.916915	3.866453	0.980510
93	1	0	2.484698	-3.803421	1.731084
94	1	0	3.273540	-2.712576	3.797093
95	1	0	1.897493	-0.948559	4.885668
96	1	0	-0.311749	-0.328931	3.904042
97	1	0	-1.108413	-1.438179	1.862872
98	1	0	2.707902	-2.589123	-0.282812
99	1	0	4.469421	-2.249355	-1.826936
100	1	0	3.702095	-0.825118	-2.505782
101	1	0	1.168614	-2.846579	-4.496129
102	1	0	1.992508	-1.324975	-4.809188
103	1	0	3.863159	-2.648394	-4.227269

104	1	0	2.922242	-3.704955	-3.179261
105	1	0	2.030900	-0.692861	0.782000
106	1	0	-0.105472	-1.247619	-3.254004
107	1	0	1.307986	-0.318828	-2.791836

(2S,1'S)–**X$_L$X$_L$–INT2a'**

Center Number	Atomic Number	Atomic Type	Coordinates (Angstroms) X	Y	Z
1	6	0	-0.086866	-3.344489	2.785689
2	6	0	0.673544	-2.333630	2.189625
3	6	0	1.775763	-1.811655	2.888803
4	6	0	2.104593	-2.288557	4.156048
5	6	0	1.336586	-3.295671	4.747911
6	6	0	0.246877	-3.822631	4.057055
7	6	0	0.336956	-1.700768	0.844263
8	6	0	1.469532	-1.788267	-0.222742
9	6	0	1.070901	-2.933448	-1.197302
10	6	0	-0.483212	-3.086108	-0.995330
11	7	0	-0.854487	-2.194410	0.118634
12	7	0	1.596712	-0.520595	-0.958838
13	6	0	2.314572	0.513887	-0.450280
14	8	0	3.028542	0.439909	0.541502
15	6	0	1.881477	-4.208596	-1.031845
16	6	0	2.021317	-4.850291	0.206426
17	6	0	2.741780	-6.041181	0.309656
18	6	0	3.333973	-6.607630	-0.820533
19	6	0	3.202397	-5.973645	-2.057299
20	6	0	2.480319	-4.784581	-2.158857
21	6	0	-0.947772	-4.520583	-0.759972
22	8	0	-1.443303	-4.920488	0.273572
23	6	0	2.141018	1.859184	-1.222362
24	7	0	0.755851	2.332567	-0.956084
25	6	0	0.766229	3.199658	0.314015
26	6	0	2.278916	3.387774	0.585934
27	6	0	3.002619	3.024211	-0.714553
28	6	0	-0.259528	2.132490	-1.750801
29	6	0	-1.686295	2.366734	-1.313793
30	6	0	-2.590687	2.676733	-2.525871
31	6	0	-1.841569	3.569771	-3.521790
32	6	0	-0.610119	2.836112	-4.102577
33	6	0	-0.122095	1.680225	-3.173035
34	6	0	4.495492	2.759444	-0.676666
35	6	0	5.247769	2.780503	0.500816
36	6	0	6.621035	2.534963	0.460187
37	6	0	7.254747	2.261152	-0.752007
38	6	0	6.510613	2.247133	-1.933366
39	6	0	5.140975	2.501730	-1.893797
40	7	0	2.550037	4.831076	0.964615
41	8	0	3.204457	5.022543	1.977793
42	6	0	-0.013670	2.704350	1.510929
43	6	0	-1.146807	3.414541	1.924740
44	6	0	-1.901939	2.973546	3.012320
45	6	0	-1.522239	1.823063	3.704813
46	6	0	-0.368799	1.130816	3.324902
47	6	0	0.388100	1.576005	2.242034
48	8	0	2.106905	5.695194	0.210783
49	8	0	-0.753906	-5.254811	-1.855244
50	6	0	-0.998886	-6.673346	-1.716997
51	6	0	-2.075345	1.045610	-0.541779
52	8	0	-1.458442	-0.009753	-1.247081
53	6	0	-3.583437	0.863276	-0.368783
54	6	0	-4.264281	-0.326657	-0.656174
55	6	0	-5.634094	-0.469196	-0.423276
56	6	0	-6.367012	0.581810	0.119119
57	6	0	-5.718926	1.776897	0.427741
58	6	0	-4.354927	1.888665	0.188809
59	9	0	-3.638554	-1.410342	-1.146808
60	9	0	-3.770597	3.069068	0.496456
61	9	0	-6.399867	2.798613	0.948264
62	9	0	-7.670871	0.448791	0.341975
63	9	0	-6.238224	-1.622396	-0.711576

64	1	0	-1.412196	-0.843845	-0.666876
65	1	0	-1.535187	-2.647408	0.724557
66	1	0	2.297322	1.689868	-2.284458
67	1	0	2.810636	3.852345	-1.411378
68	1	0	2.618382	2.790166	1.425625
69	1	0	0.363212	4.158582	-0.018488
70	1	0	0.826828	-0.305758	-1.583175
71	1	0	2.435545	-1.977852	0.240969
72	1	0	0.164957	-0.639622	1.047033
73	1	0	-0.995822	-2.749614	-1.904917
74	1	0	1.242809	-2.581822	-2.218013
75	1	0	4.780923	3.002624	1.453838
76	1	0	7.194713	2.559640	1.381883
77	1	0	8.323286	2.069354	-0.778410
78	1	0	6.996112	2.047917	-2.884263
79	1	0	4.573243	2.511258	-2.823028
80	1	0	-2.027885	-6.852129	-1.397524
81	1	0	-0.816282	-7.094859	-2.704463
82	1	0	-0.304675	-7.091081	-0.983509
83	1	0	2.388498	-1.039890	2.428753
84	1	0	2.963564	-1.877656	4.679110
85	1	0	1.592294	-3.669516	5.735056
86	1	0	-0.349549	-4.614800	4.500613
87	1	0	-0.912810	-3.804105	2.254862
88	1	0	2.372029	-4.304479	-3.128697
89	1	0	3.660507	-6.403696	-2.943490
90	1	0	3.895558	-7.533681	-0.737873
91	1	0	2.839955	-6.523227	1.278204
92	1	0	1.565828	-4.430334	1.095525
93	1	0	-1.452309	4.312361	1.393860
94	1	0	-2.785006	3.529268	3.311451
95	1	0	-2.111984	1.475908	4.547977
96	1	0	-0.048420	0.246328	3.867418
97	1	0	1.294256	1.043780	1.978348
98	1	0	-1.735069	3.199529	-0.613667
99	1	0	-3.494413	3.171705	-2.161947
100	1	0	-2.911183	1.745353	-3.007068
101	1	0	0.211913	3.537943	-4.274297
102	1	0	-0.848789	2.380154	-5.068336
103	1	0	-2.505557	3.890280	-4.329583
104	1	0	-1.522346	4.483753	-3.002762
105	1	0	-1.643330	1.132902	0.462208
106	1	0	0.880612	1.363783	-3.456933
107	1	0	-0.785535	0.819622	-3.288108

(*Re*,*Re*)–**X_LX_L–TS1b**

Center Number	Atomic Number	Atomic Type	Coordinates (Angstroms) X	Y	Z
1	6	0	4.188084	1.421208	-0.806329
2	6	0	3.737129	0.279159	-0.128024
3	6	0	4.697642	-0.430100	0.605752
4	6	0	6.028457	-0.042279	0.683814
5	6	0	6.441428	1.105773	0.005861
6	6	0	5.518004	1.836605	-0.738498
7	6	0	2.310741	-0.181835	-0.145372
8	6	0	2.454213	-1.958535	-1.127048
9	6	0	1.113914	-2.001159	-1.595641
10	6	0	0.931598	-1.342664	-2.933845
11	6	0	1.773372	-2.062187	-4.029857
12	6	0	3.079143	-2.656854	-3.463037
13	6	0	3.521294	-1.870138	-2.219174
14	7	0	0.079013	-2.451189	-0.877969
15	6	0	-1.341089	-2.342527	-1.267744
16	6	0	-1.897057	-3.727757	-0.890725
17	6	0	-1.213724	-3.964435	0.458457
18	6	0	0.201019	-3.337550	0.334807
19	6	0	-1.984051	-1.135653	-0.532423
20	8	0	-2.907017	-1.265737	0.261578
21	6	0	0.657711	-2.662688	1.615237
22	6	0	1.873295	-3.034590	2.196653
23	6	0	2.320965	-2.418645	3.367621
24	6	0	1.549655	-1.428913	3.975908
25	6	0	0.314667	-1.075588	3.423353

26	6	0	-0.131299	-1.694955	2.256867
27	7	0	-1.098473	-5.443976	0.762216
28	8	0	-0.410996	-6.116404	-0.002371
29	6	0	-3.400843	-3.895531	-0.987998
30	6	0	-4.199002	-4.271600	0.096185
31	6	0	-5.577152	-4.424336	-0.064322
32	6	0	-6.173330	-4.201343	-1.305669
33	6	0	-5.380776	-3.837077	-2.396252
34	6	0	-4.003872	-3.692371	-2.237152
35	9	0	4.332833	-1.556654	1.247758
36	9	0	6.904905	-0.754352	1.393550
37	9	0	7.709971	1.498145	0.068936
38	9	0	5.908692	2.935573	-1.387163
39	9	0	3.360685	2.179640	-1.542208
40	7	0	-1.427385	0.067255	-0.869841
41	6	0	-1.852177	1.287623	-0.190571
42	6	0	-1.730160	2.512637	-1.150933
43	6	0	-0.357009	3.170265	-0.781607
44	7	0	0.121914	2.488216	0.469856
45	6	0	-0.948904	1.585344	1.042368
46	6	0	-2.924309	3.453012	-1.143977
47	6	0	-3.389451	4.069041	0.026303
48	6	0	-4.468988	4.952444	-0.019932
49	6	0	-5.101325	5.229958	-1.233751
50	6	0	-4.647903	4.616674	-2.402906
51	6	0	-3.565873	3.737174	-2.355723
52	6	0	-1.657866	2.084576	2.286421
53	6	0	-2.630078	1.225026	2.826176
54	6	0	-3.293332	1.561360	4.003871
55	6	0	-2.996318	2.758789	4.660918
56	6	0	-2.036262	3.616163	4.127300
57	6	0	-1.365061	3.282178	2.946640
58	6	0	-0.377634	4.675386	-0.541636
59	8	0	-0.695222	5.317962	-1.652339
60	6	0	-0.915355	6.746899	-1.527830
61	8	0	-0.114050	5.178837	0.533715
62	8	0	-1.706179	-5.859572	1.739142
63	8	0	1.424799	0.602190	-0.689635
64	1	0	0.895152	1.820731	0.149132
65	1	0	0.465822	3.193635	1.131551
66	1	0	-1.436813	-2.178281	-2.339011
67	1	0	-1.420711	-4.423767	-1.594974
68	1	0	-1.777126	-3.539704	1.284411
69	1	0	0.908108	-4.125662	0.063400
70	1	0	-0.441515	0.070201	-1.141872
71	1	0	-2.884959	1.141642	0.119725
72	1	0	-0.417814	0.669612	1.307506
73	1	0	0.387076	2.954693	-1.552695
74	1	0	-1.637131	2.114889	-2.163289
75	1	0	-3.758717	-4.465355	1.068592
76	1	0	-6.183653	-4.721498	0.786499
77	1	0	-7.246046	-4.320850	-1.426365
78	1	0	-5.831923	-3.674677	-3.371248
79	1	0	-3.392960	-3.428414	-3.099162
80	1	0	-0.031833	7.228907	-1.105001
81	1	0	-1.107917	7.094379	-2.541223
82	1	0	-1.782658	6.917635	-0.885767
83	1	0	-2.877809	0.298125	2.313369
84	1	0	-4.045015	0.888685	4.406159
85	1	0	-3.514343	3.021691	5.578326
86	1	0	-1.804633	4.554425	4.622650
87	1	0	-0.655776	3.995925	2.541039
88	1	0	-3.212394	3.272898	-3.273481
89	1	0	-5.134418	4.822157	-3.352286
90	1	0	-5.943327	5.915184	-1.267217
91	1	0	-4.817393	5.418393	0.897548
92	1	0	-2.916915	3.866453	0.980510
93	1	0	2.484698	-3.803421	1.731084
94	1	0	3.273540	-2.712576	3.797093
95	1	0	1.897493	-0.948559	4.885668
96	1	0	-0.311749	-0.328931	3.904042
97	1	0	-1.108413	-1.438179	1.862872
98	1	0	2.707902	-2.589123	-0.282812
99	1	0	4.469421	-2.249355	-1.826936
100	1	0	3.702095	-0.825118	-2.505782
101	1	0	1.168614	-2.846579	-4.496129
102	1	0	1.992508	-1.324975	-4.809188

103	1	0	3.863159	-2.648394	-4.227269
104	1	0	2.922242	-3.704955	-3.179261
105	1	0	2.030900	-0.692861	0.782000
106	1	0	-0.105472	-1.247619	-3.254004
107	1	0	1.307986	-0.318828	-2.791836

(*2S,1'R*)–X_LX_L–INT2b

Center Number	Atomic Number	Atomic Type	Coordinates (Angstroms) X	Y	Z
1	6	0	-0.086866	-3.344489	2.785689
2	6	0	0.673544	-2.333630	2.189625
3	6	0	1.775763	-1.811655	2.888803
4	6	0	2.104593	-2.288557	4.156048
5	6	0	1.336586	-3.295671	4.747911
6	6	0	0.246877	-3.822631	4.057055
7	6	0	0.336956	-1.700768	0.844263
8	6	0	1.469532	-1.788267	-0.222742
9	6	0	1.070901	-2.933448	-1.197302
10	6	0	-0.483212	-3.086108	-0.995330
11	7	0	-0.854487	-2.194410	0.118634
12	7	0	1.596712	-0.520595	-0.958838
13	6	0	2.314572	0.513887	-0.450280
14	8	0	3.028542	0.439909	0.541502
15	6	0	1.881477	-4.208596	-1.031845
16	6	0	2.021317	-4.850291	0.206426
17	6	0	2.741780	-6.041181	0.309656
18	6	0	3.333973	-6.607630	-0.820533
19	6	0	3.202397	-5.973645	-2.057299
20	6	0	2.480319	-4.784581	-2.158857
21	6	0	-0.947772	-4.520583	-0.759972
22	8	0	-1.443303	-4.920488	0.273572
23	6	0	2.141018	1.859184	-1.222362
24	7	0	0.755851	2.332567	-0.956084
25	6	0	0.766229	3.199658	0.314015
26	6	0	2.278916	3.387774	0.585934
27	6	0	3.002619	3.024211	-0.714553
28	6	0	-0.259528	2.132490	-1.750801
29	6	0	-1.686295	2.366734	-1.313793
30	6	0	-2.590687	2.676733	-2.525871
31	6	0	-1.841569	3.569771	-3.521790
32	6	0	-0.610119	2.836112	-4.102577
33	6	0	-0.122095	1.680225	-3.173035
34	6	0	4.495492	2.759444	-0.676666
35	6	0	5.247769	2.780503	0.500816
36	6	0	6.621035	2.534963	0.460187
37	6	0	7.254747	2.261152	-0.752007
38	6	0	6.510613	2.247133	-1.933366
39	6	0	5.140975	2.501730	-1.893797
40	7	0	2.550037	4.831076	0.964615
41	8	0	3.204457	5.022543	1.977793
42	6	0	-0.013670	2.704350	1.510929
43	6	0	-1.146807	3.414541	1.924740
44	6	0	-1.901939	2.973546	3.012320
45	6	0	-1.522239	1.823063	3.704813
46	6	0	-0.368799	1.130816	3.324902
47	6	0	0.388100	1.576005	2.242034
48	8	0	2.106905	5.695194	0.210783
49	8	0	-0.753906	-5.254811	-1.855244
50	6	0	-0.998886	-6.673346	-1.716997
51	6	0	-2.075345	1.045610	-0.541779
52	8	0	-1.458442	-0.009753	-1.247081
53	6	0	-3.583437	0.863276	-0.368783
54	6	0	-4.264281	-0.326657	-0.656174
55	6	0	-5.634094	-0.469196	-0.423276
56	6	0	-6.367012	0.581810	0.119119
57	6	0	-5.718926	1.776897	0.427741
58	6	0	-4.354927	1.888665	0.188809
59	9	0	-3.638554	-1.410342	-1.146808
60	9	0	-3.770597	3.069068	0.496456
61	9	0	-6.399867	2.798613	0.948264
62	9	0	-7.670871	0.448791	0.341975
63	9	0	-6.238224	-1.622396	-0.711576
64	1	0	-1.412196	-0.843845	-0.666876
65	1	0	-1.535187	-2.647408	0.724557

66	1	0	2.297322	1.689868	-2.284458
67	1	0	2.810636	3.852345	-1.411378
68	1	0	2.618382	2.790166	1.425625
69	1	0	0.363212	4.158582	-0.018488
70	1	0	0.826828	-0.305758	-1.583175
71	1	0	2.435545	-1.977852	0.240969
72	1	0	0.164957	-0.639622	1.047033
73	1	0	-0.995822	-2.749614	-1.904917
74	1	0	1.242809	-2.581822	-2.218013
75	1	0	4.780923	3.002624	1.453838
76	1	0	7.194713	2.559640	1.381883
77	1	0	8.323286	2.069354	-0.778410
78	1	0	6.996112	2.047917	-2.884263
79	1	0	4.573243	2.511258	-2.823028
80	1	0	-2.027885	-6.852129	-1.397524
81	1	0	-0.816282	-7.094859	-2.704463
82	1	0	-0.304675	-7.091081	-0.983509
83	1	0	2.388498	-1.039890	2.428753
84	1	0	2.963564	-1.877656	4.679110
85	1	0	1.592294	-3.669516	5.735056
86	1	0	-0.349549	-4.614800	4.500613
87	1	0	-0.912810	-3.804105	2.254862
88	1	0	2.372029	-4.304479	-3.128697
89	1	0	3.660507	-6.403696	-2.943490
90	1	0	3.895558	-7.533681	-0.737873
91	1	0	2.839955	-6.523227	1.278204
92	1	0	1.565828	-4.430334	1.095525
93	1	0	-1.452309	4.312361	1.393860
94	1	0	-2.785006	3.529268	3.311451
95	1	0	-2.111984	1.475908	4.547977
96	1	0	-0.048420	0.246328	3.867418
97	1	0	1.294256	1.043780	1.978348
98	1	0	-1.735069	3.199529	-0.613667
99	1	0	-3.494413	3.171705	-2.161947
100	1	0	-2.911183	1.745353	-3.007068
101	1	0	0.211913	3.537943	-4.274297
102	1	0	-0.848789	2.380154	-5.068336
103	1	0	-2.505557	3.890280	-4.329583
104	1	0	-1.522346	4.483753	-3.002762
105	1	0	-1.643330	1.132902	0.462208
106	1	0	0.880612	1.363783	-3.456933
107	1	0	-0.785535	0.819622	-3.288108

(*Re*,*Re*)–**X$_L$X$_L$–TS1a’**

Center Number	Atomic Number	Atomic Type	Coordinates (Angstroms) X	Y	Z
1	6	0	5.049713	-1.453234	-0.123617
2	6	0	4.467267	-0.912827	-1.279077
3	6	0	5.304664	-0.463203	-2.307076
4	6	0	6.692667	-0.547242	-2.187953
5	6	0	7.263541	-1.090216	-1.036197
6	6	0	6.437021	-1.543709	-0.005578
7	6	0	2.961916	-0.794431	-1.459249
8	6	0	2.272166	0.170462	-0.449243
9	6	0	1.590921	-0.719348	0.628100
10	7	0	1.228900	-1.954413	-0.160955
11	6	0	2.146654	-2.125844	-1.335938
12	7	0	1.270062	1.001604	-1.120382
13	6	0	0.847653	2.135804	-0.489438
14	8	0	1.399704	2.622240	0.490406
15	6	0	2.358868	-0.940485	1.916100
16	6	0	2.747671	0.212594	2.618170
17	6	0	3.382037	0.107356	3.854053
18	6	0	3.636932	-1.149441	4.409725
19	6	0	3.252602	-2.296550	3.717755
20	6	0	2.611922	-2.195419	2.479222
21	6	0	2.950397	-3.408661	-1.166091
22	8	0	2.857281	-4.128027	-0.190226
23	6	0	-0.427873	2.762164	-1.108968
24	7	0	-1.571126	1.877068	-0.810795
25	6	0	-2.146376	2.247289	0.550635
26	6	0	-1.469670	3.614929	0.843824
27	6	0	-0.863094	4.097050	-0.480824

28	6	0	-2.258254	1.208197	-1.734759
29	6	0	-3.163649	0.167532	-1.372919
30	6	0	-4.430374	-0.006794	-2.219926
31	6	0	-4.407802	0.854391	-3.492995
32	6	0	-3.020671	0.810198	-4.137740
33	6	0	-1.967581	1.439718	-3.201216
34	6	0	0.190916	5.186408	-0.446342
35	6	0	0.600184	5.816890	0.732289
36	6	0	1.564966	6.824982	0.692454
37	6	0	2.133055	7.212386	-0.521266
38	6	0	1.721617	6.594469	-1.704147
39	6	0	0.753297	5.592935	-1.664475
40	7	0	-2.508415	4.596727	1.349410
41	8	0	-2.270723	5.160919	2.407980
42	6	0	-2.027828	1.253094	1.692063
43	6	0	-3.184652	0.596408	2.134634
44	6	0	-3.135365	-0.298469	3.204054
45	6	0	-1.926425	-0.538416	3.857729
46	6	0	-0.775768	0.139545	3.451961
47	6	0	-0.827333	1.034207	2.382595
48	8	0	-3.505584	4.761657	0.650055
49	8	0	3.712428	-3.621283	-2.227278
50	6	0	4.640798	-4.731923	-2.139167
51	6	0	-1.977436	-1.314869	-1.700016
52	8	0	-0.714361	-1.026396	-1.517393
53	6	0	-2.590989	-2.367877	-0.802761
54	6	0	-3.538809	-3.276580	-1.286892
55	6	0	-4.140883	-4.237094	-0.477199
56	6	0	-3.786556	-4.318362	0.868921
57	6	0	-2.828174	-3.444784	1.381992
58	6	0	-2.241487	-2.502770	0.544947
59	9	0	-4.353892	-5.225548	1.658333
60	9	0	-5.042075	-5.078943	-0.981860
61	9	0	-3.905882	-3.239901	-2.581486
62	9	0	-1.304616	-1.703146	1.091542
63	9	0	-2.476549	-3.514458	2.669411
64	1	0	0.253449	-1.738232	-0.584624
65	1	0	1.212625	-2.809601	0.403132
66	1	0	-0.278624	2.851208	-2.182803
67	1	0	-1.707834	4.441224	-1.094103
68	1	0	-0.726147	3.543969	1.630522
69	1	0	-3.204579	2.431865	0.357435
70	1	0	0.550884	0.473686	-1.624058
71	1	0	2.999472	0.840869	0.002672
72	1	0	0.630769	-0.274957	0.890194
73	1	0	1.502762	-2.244243	-2.211132
74	1	0	2.788124	-0.394629	-2.460452
75	1	0	0.162867	5.544957	1.686874
76	1	0	1.869535	7.308632	1.616187
77	1	0	2.883761	7.996757	-0.547756
78	1	0	2.147579	6.896574	-2.656742
79	1	0	0.425895	5.132194	-2.595202
80	1	0	4.105439	-5.654913	-1.908012
81	1	0	5.115308	-4.785179	-3.117477
82	1	0	5.377471	-4.518851	-1.360836
83	1	0	2.547043	1.194213	2.196570
84	1	0	3.678774	1.008877	4.382042
85	1	0	4.133475	-1.231655	5.371936
86	1	0	3.450350	-3.279412	4.135232
87	1	0	2.361826	-3.113274	1.957823
88	1	0	4.869611	-0.047095	-3.212904
89	1	0	7.325282	-0.191226	-2.996348
90	1	0	8.343384	-1.158213	-0.941288
91	1	0	6.869493	-1.964157	0.898032
92	1	0	4.429485	-1.810612	0.689998
93	1	0	-4.138573	0.796111	1.651359
94	1	0	-4.042415	-0.797937	3.531454
95	1	0	-1.885163	-1.239076	4.685954
96	1	0	0.167915	-0.021752	3.964359
97	1	0	0.072885	1.569340	2.101567
98	1	0	-3.329343	0.073855	-0.306015
99	1	0	-5.300780	0.253662	-1.606979
100	1	0	-4.555481	-1.058370	-2.494063
101	1	0	-3.008464	1.353750	-5.087710
102	1	0	-2.753275	-0.226320	-4.371886
103	1	0	-5.171860	0.506537	-4.195119
104	1	0	-4.656015	1.895509	-3.243111

105	1	0	-2.286590	-1.472605	-2.740192
106	1	0	-1.935084	2.523450	-3.372720
107	1	0	-0.968397	1.050495	-3.435478

(2S,1'R)–**X$_L$X$_L$–INT2a'**

Center Number	Atomic Number	Atomic Type	Coordinates (Angstroms) X	Y	Z
1	6	0	-2.672807	2.082305	0.499115
2	6	0	-2.606902	2.453296	-0.845089
3	6	0	-3.628039	3.281188	-1.318157
4	6	0	-4.667329	3.720498	-0.500595
5	6	0	-4.698422	3.329134	0.838259
6	6	0	-3.692380	2.504826	1.343483
7	6	0	-1.458042	2.044367	-1.742422
8	8	0	-1.099650	0.679823	-1.686443
9	9	0	-3.608419	3.701736	-2.595827
10	9	0	-5.621477	4.512163	-0.986848
11	9	0	-5.679499	3.745315	1.632062
12	9	0	-3.702337	2.135713	2.626219
13	9	0	-1.715025	1.293323	1.014123
14	6	0	-0.187639	2.956775	-1.415795
15	6	0	0.983385	2.106345	-1.789470
16	6	0	1.155353	1.786602	-3.259050
17	6	0	0.519775	2.873902	-4.162642
18	6	0	0.605910	4.251404	-3.494698
19	6	0	-0.224167	4.300281	-2.196507
20	7	0	1.819050	1.659936	-0.894065
21	6	0	2.795577	0.573214	-1.139833
22	6	0	4.085162	1.129666	-0.520675
23	6	0	3.548720	1.751113	0.776545
24	6	0	2.144913	2.327991	0.458378
25	6	0	2.225483	-0.705288	-0.446302
26	8	0	2.740748	-1.153442	0.569580
27	6	0	1.158165	2.217341	1.601063
28	6	0	0.458614	3.369070	1.991789
29	6	0	-0.433301	3.330514	3.063334
30	6	0	-0.627198	2.140072	3.765881
31	6	0	0.093867	0.999034	3.409574
32	6	0	0.987625	1.038553	2.339634
33	7	0	4.456762	2.873555	1.243872
34	8	0	4.529601	3.865587	0.521085
35	6	0	5.242770	0.152996	-0.439020
36	6	0	5.862810	-0.199680	0.762653
37	6	0	6.931594	-1.097393	0.763968
38	6	0	7.388817	-1.654075	-0.430445
39	6	0	6.780245	-1.298579	-1.635782
40	6	0	5.718533	-0.395859	-1.637768
41	7	0	1.129629	-1.227611	-1.052999
42	6	0	0.316479	-2.243081	-0.363963
43	6	0	-0.563268	-3.017383	-1.385672
44	6	0	-1.970845	-2.320815	-1.280983
45	7	0	-1.877528	-1.341029	-0.182425
46	6	0	-0.662244	-1.562187	0.635559
47	6	0	-0.558074	-4.525144	-1.193165
48	6	0	-0.912500	-5.124348	0.023857
49	6	0	-0.925840	-6.513940	0.151899
50	6	0	-0.583339	-7.325695	-0.931248
51	6	0	-0.225969	-6.737631	-2.145596
52	6	0	-0.215897	-5.348328	-2.272530
53	6	0	-0.814471	-2.277039	1.973766
54	6	0	0.351381	-2.492666	2.729004
55	6	0	0.282765	-3.062884	3.998532
56	6	0	-0.954108	-3.427476	4.538513
57	6	0	-2.113690	-3.223033	3.792874
58	6	0	-2.047000	-2.649150	2.519242
59	6	0	-3.140386	-3.282760	-1.095617
60	8	0	-3.288456	-4.047350	-2.177793
61	6	0	-4.252460	-5.119155	-2.067597
62	8	0	-3.847815	-3.320562	-0.109407
63	8	0	5.059135	2.698551	2.291686
64	1	0	-1.566069	0.104894	-0.991476
65	1	0	-2.729047	-1.347855	0.374420
66	1	0	2.899376	0.379018	-2.205519

67	1	0	4.377833	1.971786	-1.163969
68	1	0	3.517548	1.034313	1.590060
69	1	0	2.269761	3.379129	0.193632
70	1	0	0.610501	-0.624667	-1.685322
71	1	0	1.010584	-2.909027	0.143936
72	1	0	-0.258731	-0.574407	0.868466
73	1	0	-2.164671	-1.782540	-2.216541
74	1	0	-0.166625	-2.824613	-2.385779
75	1	0	5.535101	0.228860	1.703243
76	1	0	7.406671	-1.359351	1.704686
77	1	0	8.219595	-2.353375	-0.424414
78	1	0	7.135795	-1.716610	-2.573051
79	1	0	5.265000	-0.109733	-2.585480
80	1	0	-5.239598	-4.720611	-1.823283
81	1	0	-4.256143	-5.605081	-3.042298
82	1	0	-3.930636	-5.815983	-1.289481
83	1	0	1.319121	-2.218736	2.315956
84	1	0	1.195775	-3.227064	4.564085
85	1	0	-1.009578	-3.873553	5.527236
86	1	0	-3.079894	-3.513734	4.195329
87	1	0	-2.962376	-2.544021	1.948349
88	1	0	0.052011	-4.899355	-3.226209
89	1	0	0.043652	-7.358624	-2.995221
90	1	0	-0.592771	-8.407037	-0.829096
91	1	0	-1.202506	-6.959501	1.103283
92	1	0	-1.187024	-4.513119	0.875557
93	1	0	0.621666	4.307780	1.465718
94	1	0	-0.965905	4.231103	3.354098
95	1	0	-1.328286	2.105548	4.593929
96	1	0	-0.037282	0.069874	3.955273
97	1	0	1.549788	0.146197	2.090757
98	1	0	-0.191794	3.130718	-0.344030
99	1	0	0.138058	5.106320	-1.550910
100	1	0	-1.269666	4.530420	-2.426678
101	1	0	1.052263	2.871181	-5.118482
102	1	0	-0.521573	2.626185	-4.390957
103	1	0	0.268097	5.032880	-4.181645
104	1	0	1.659625	4.469774	-3.269150
105	1	0	-1.758383	2.266037	-2.768800
106	1	0	2.228278	1.733377	-3.473648
107	1	0	0.729614	0.802738	-3.482291

(*Re*,*Si*)–**X$_L$X$_L$–TS1b**

Center Number	Atomic Number	Atomic Type	Coordinates (Angstroms) X	Y	Z
1	6	0	3.926066	3.081666	-2.122948
2	6	0	3.682303	4.097289	-1.198915
3	6	0	2.696200	3.925110	-0.228019
4	6	0	1.922519	2.763757	-0.140020
5	6	0	2.194018	1.780524	-1.092283
6	6	0	3.166800	1.911642	-2.074913
7	6	0	0.895099	2.582903	0.961328
8	8	0	0.821006	1.432271	1.560483
9	6	0	-0.733713	3.035419	0.010104
10	6	0	-1.609362	2.333819	0.891926
11	6	0	-1.917885	2.940892	2.235013
12	6	0	-1.621096	4.452762	2.319718
13	6	0	-1.844134	5.121303	0.961112
14	6	0	-0.854532	4.560895	-0.070038
15	7	0	-1.975865	1.073917	0.653072
16	6	0	-2.518330	0.159041	1.683661
17	6	0	-2.474110	-1.262313	1.024268
18	6	0	-2.261896	-1.059289	-0.504497
19	6	0	-1.821148	0.439602	-0.665895
20	7	0	-1.540130	-2.189238	1.684828
21	6	0	-0.190299	-2.155519	1.843271
22	8	0	0.373366	-2.745115	2.765429
23	6	0	-3.913928	0.495067	2.192783
24	6	0	-4.296883	-0.033022	3.432564
25	6	0	-5.583717	0.181491	3.925133
26	6	0	-6.497551	0.936022	3.185152
27	6	0	-6.115402	1.473841	1.955318
28	6	0	-4.828937	1.255547	1.455403

29	6	0	-2.623021	1.180528	-1.740573
30	8	0	-2.146638	0.841669	-2.941161
31	6	0	-2.924423	1.291426	-4.077332
32	6	0	-3.449494	-1.464542	-1.370427
33	6	0	-3.198890	-2.086845	-2.600741
34	6	0	-4.244970	-2.432026	-3.455452
35	6	0	-5.564737	-2.157164	-3.090894
36	6	0	-5.825829	-1.536691	-1.868506
37	6	0	-4.776622	-1.191356	-1.015333
38	6	0	0.655131	-1.399600	0.821655
39	7	0	1.854940	-0.874833	1.547554
40	6	0	3.129443	-1.146444	0.788554
41	6	0	2.720001	-2.189576	-0.271119
42	6	0	1.181214	-2.222832	-0.407483
43	6	0	0.615452	-3.613120	-0.627936
44	6	0	-0.034558	-3.889234	-1.836461
45	6	0	-0.565861	-5.153965	-2.090231
46	6	0	-0.438153	-6.165870	-1.138353
47	6	0	0.234677	-5.909117	0.058649
48	6	0	0.762525	-4.643418	0.312053
49	7	0	3.387869	-1.862318	-1.602321
50	8	0	2.812548	-2.212766	-2.622679
51	6	0	4.247004	-1.571426	1.711299
52	6	0	5.489879	-0.937144	1.635403
53	6	0	6.536122	-1.346193	2.463687
54	6	0	6.343490	-2.386969	3.373561
55	6	0	5.102714	-3.025016	3.451698
56	6	0	4.058179	-2.623962	2.620052
57	8	0	-3.542768	1.939049	-1.535708
58	8	0	4.479991	-1.307836	-1.545457
59	1	0	1.884167	-1.323984	2.471966
60	1	0	0.140028	-0.511704	0.479903
61	1	0	0.937392	-1.634467	-1.292104
62	1	0	3.118909	-3.175284	-0.021733
63	1	0	3.396884	-0.217930	0.286060
64	1	0	-1.969265	-2.782622	2.387448
65	1	0	-3.442701	-1.734332	1.176429
66	1	0	-1.821162	0.173907	2.526834
67	1	0	-0.776390	0.473256	-0.973897
68	1	0	-1.429649	-1.687989	-0.818920
69	1	0	-2.371404	0.957869	-4.954421
70	1	0	-3.914708	0.830374	-4.043276
71	1	0	-3.019476	2.379380	-4.062551
72	1	0	-0.091415	-3.116096	-2.597919
73	1	0	-1.068259	-5.348944	-3.033112
74	1	0	-0.847728	-7.152844	-1.332235
75	1	0	0.352640	-6.696675	0.797327
76	1	0	1.275193	-4.460184	1.250961
77	1	0	-2.175769	-2.295325	-2.895812
78	1	0	-4.028931	-2.919107	-4.402172
79	1	0	-6.382435	-2.427533	-3.752512
80	1	0	-6.847962	-1.316470	-1.574359
81	1	0	-5.006559	-0.698022	-0.077148
82	1	0	-3.584016	-0.608936	4.020065
83	1	0	-5.868360	-0.230409	4.888986
84	1	0	-7.498030	1.109689	3.570087
85	1	0	-6.817682	2.068279	1.377911
86	1	0	-4.537650	1.679109	0.499240
87	1	0	5.644248	-0.137380	0.916782
88	1	0	7.500241	-0.850860	2.397385
89	1	0	7.157703	-2.702546	4.019186
90	1	0	4.948734	-3.836951	4.156227
91	1	0	3.095862	-3.129773	2.688816
92	1	0	-0.577558	2.574707	-0.960655
93	1	0	-2.967209	2.752552	2.473932
94	1	0	-1.323626	2.383759	2.974180
95	1	0	-1.172450	4.835126	-1.082186
96	1	0	0.130255	5.020760	0.079890
97	1	0	-2.267215	4.891928	3.086023
98	1	0	-0.590228	4.625980	2.648999
99	1	0	-1.729874	6.206801	1.039526
100	1	0	-2.872307	4.927661	0.626940
101	1	0	0.884489	3.467266	1.610646
102	9	0	2.495301	4.931429	0.640336
103	9	0	1.472627	0.625535	-1.102877
104	9	0	4.398570	5.219569	-1.240753
105	9	0	3.371577	0.931519	-2.955631

106	9	0	4.865783	3.230232	-3.051326
107	1	0	1.654700	0.195168	1.636363

(2S,1'S)–**X_LX_L–INT2b**

Center Number	Atomic Number	Atomic Type	Coordinates (Angstroms) X	Y	Z
1	6	0	-4.112644	-2.096493	-2.978954
2	6	0	-4.247807	-1.428355	-1.752459
3	6	0	-5.483819	-0.876415	-1.403475
4	6	0	-6.576284	-0.989702	-2.264956
5	6	0	-6.436074	-1.651918	-3.485154
6	6	0	-5.202347	-2.203952	-3.841135
7	6	0	-3.079188	-1.316082	-0.806804
8	7	0	-1.901232	-0.639806	-1.418846
9	6	0	-0.649015	-1.232495	-0.890065
10	6	0	-1.110513	-2.258255	0.206444
11	6	0	-2.500813	-2.653655	-0.308674
12	6	0	0.153730	-1.856460	-2.026078
13	8	0	-0.427223	-2.477063	-2.913731
14	7	0	-3.336815	-3.227040	0.803621
15	8	0	-3.479240	-2.526575	1.807403
16	6	0	-0.234189	-3.423337	0.611928
17	6	0	0.092901	-3.565643	1.968659
18	6	0	0.817873	-4.670039	2.419848
19	6	0	1.235812	-5.645346	1.513469
20	6	0	0.915246	-5.513758	0.159276
21	6	0	0.177544	-4.418708	-0.288857
22	7	0	1.522963	-1.767463	-2.034849
23	6	0	2.517225	-0.974224	-1.305268
24	6	0	2.565224	0.533227	-1.715505
25	7	0	1.894248	1.232526	-0.583806
26	6	0	1.885544	0.412198	0.653774
27	6	0	2.421229	-0.999669	0.254517
28	6	0	1.313871	2.394958	-0.708283
29	6	0	0.357637	2.927551	0.317398
30	6	0	0.738776	4.361356	0.783959
31	6	0	1.677909	5.077558	-0.203563
32	6	0	1.323742	4.746327	-1.658262
33	6	0	1.528149	3.235189	-1.937125
34	6	0	3.973257	1.027740	-2.030476
35	6	0	4.602986	0.485595	-3.159262
36	6	0	5.893476	0.879579	-3.506835
37	6	0	6.567969	1.827715	-2.732952
38	6	0	5.940562	2.379958	-1.617005
39	6	0	4.646355	1.986143	-1.265987
40	6	0	3.717610	-1.466878	0.901406
41	6	0	3.763684	-2.771152	1.407052
42	6	0	4.936047	-3.273381	1.973746
43	6	0	6.079096	-2.476601	2.045010
44	6	0	6.043688	-1.176530	1.536016
45	6	0	4.873917	-0.677298	0.962373
46	6	0	2.619831	1.136855	1.788150
47	8	0	3.141646	2.227975	1.691583
48	8	0	2.529664	0.406315	2.891547
49	6	0	3.283746	0.874382	4.038143
50	8	0	-3.816325	-4.338118	0.631087
51	6	0	-1.048713	2.887294	-0.422796
52	8	0	-1.038843	1.903692	-1.437727
53	6	0	-2.189834	2.740021	0.560268
54	6	0	-3.181106	3.717644	0.671725
55	6	0	-4.291042	3.555356	1.500744
56	6	0	-4.433055	2.382019	2.241444
57	6	0	-3.452808	1.389921	2.166288
58	6	0	-2.356538	1.595905	1.340131
59	9	0	-5.492141	2.211430	3.024846
60	9	0	-5.217329	4.508053	1.580441
61	9	0	-3.077164	4.856600	-0.028424
62	9	0	-1.412024	0.621013	1.296594
63	9	0	-3.573118	0.260374	2.860773
64	1	0	-1.934759	-0.794212	-2.425686
65	1	0	-0.077745	-0.437886	-0.425346
66	1	0	-1.296272	-1.659283	1.101187
67	1	0	-2.466609	-3.413911	-1.087333

68	1	0	-3.377049	-0.752980	0.082893
69	1	0	1.900558	-2.287946	-2.819589
70	1	0	3.468932	-1.422580	-1.585610
71	1	0	1.927312	0.676004	-2.592082
72	1	0	0.859054	0.306087	1.003477
73	1	0	1.659496	-1.727402	0.531216
74	1	0	3.010228	0.205440	4.852178
75	1	0	4.350850	0.799400	3.815936
76	1	0	3.018894	1.908142	4.268560
77	1	0	-0.243361	-2.815989	2.680896
78	1	0	1.050809	-4.767335	3.476195
79	1	0	1.796724	-6.508784	1.859339
80	1	0	1.224444	-6.277146	-0.548866
81	1	0	-0.093334	-4.351647	-1.337494
82	1	0	2.880584	-3.399495	1.360208
83	1	0	4.950339	-4.287561	2.362655
84	1	0	6.991023	-2.864843	2.489065
85	1	0	6.929854	-0.549289	1.575015
86	1	0	4.877678	0.329211	0.561576
87	1	0	4.081519	-0.245473	-3.773959
88	1	0	6.369393	0.450861	-4.383628
89	1	0	7.573279	2.135946	-3.003515
90	1	0	6.454684	3.120538	-1.011399
91	1	0	4.176056	2.417306	-0.388360
92	1	0	-5.593852	-0.362424	-0.451288
93	1	0	-7.533803	-0.561797	-1.982675
94	1	0	-7.285306	-1.741211	-4.156349
95	1	0	-5.091162	-2.724307	-4.788020
96	1	0	-3.156271	-2.535230	-3.257780
97	1	0	0.306133	2.269655	1.180396
98	1	0	2.535271	3.061900	-2.319606
99	1	0	0.822414	2.873569	-2.695602
100	1	0	1.215543	4.299235	1.765176
101	1	0	-0.190419	4.930566	0.909286
102	1	0	1.957576	5.309069	-2.349827
103	1	0	0.292364	5.044882	-1.873750
104	1	0	1.639345	6.157933	-0.034268
105	1	0	2.712022	4.762128	-0.008575
106	1	0	-1.175992	3.851413	-0.918442
107	1	0	-1.575091	1.076014	-1.259427

XLXL–INT1a·13a

Center Number	Atomic Number	Atomic Type	Coordinates (Angstroms) X	Y	Z
1	6	0	4.933541	0.884318	1.082978
2	6	0	4.754960	0.550813	-0.264063
3	6	0	5.780672	0.868154	-1.170761
4	6	0	6.963473	1.465278	-0.739232
5	6	0	7.137499	1.774415	0.612169
6	6	0	6.116434	1.487727	1.516708
7	6	0	3.476191	-0.054673	-0.834428
8	6	0	2.197364	0.063157	0.027867
9	6	0	2.069937	-1.261863	0.821329
10	7	0	2.677287	-2.280172	-0.131128
11	6	0	3.502042	-1.588084	-1.185033
12	7	0	1.052130	0.223919	-0.873581
13	6	0	-0.188991	0.517537	-0.375863
14	8	0	-0.502451	0.291803	0.790884
15	6	0	2.662213	-1.265591	2.213084
16	6	0	1.916049	-0.593857	3.194018
17	6	0	2.391331	-0.508596	4.500820
18	6	0	3.607303	-1.106036	4.845624
19	6	0	4.345975	-1.780690	3.874630
20	6	0	3.881347	-1.857198	2.558913
21	6	0	4.877174	-2.242803	-1.220287
22	8	0	5.282727	-2.974105	-0.339142
23	6	0	-1.115913	1.247783	-1.366474
24	7	0	-2.520722	1.094545	-1.061131
25	6	0	-3.104965	2.321891	-0.484843
26	6	0	-1.841301	3.134577	-0.142520
27	6	0	-0.870172	2.792140	-1.270205
28	6	0	-3.275800	0.102270	-1.713954
29	6	0	-2.697032	-0.992732	-2.259647

30	6	0	-3.454440	-2.060985	-3.014036
31	6	0	-4.968379	-1.996311	-2.774084
32	6	0	-5.448631	-0.545863	-2.852406
33	6	0	-4.775600	0.316361	-1.775220
34	6	0	0.580216	3.202171	-1.127996
35	6	0	1.178929	3.474947	0.108702
36	6	0	2.555922	3.699788	0.195003
37	6	0	3.354586	3.651609	-0.948233
38	6	0	2.761348	3.414316	-2.191582
39	6	0	1.384637	3.200461	-2.278933
40	7	0	-2.112327	4.613203	-0.080732
41	8	0	-1.709709	5.206868	0.913057
42	6	0	-3.998477	2.113558	0.723618
43	6	0	-5.270238	2.691999	0.758032
44	6	0	-6.087916	2.539781	1.880057
45	6	0	-5.635280	1.808202	2.979273
46	6	0	-4.360172	1.233907	2.953123
47	6	0	-3.544248	1.386907	1.832556
48	8	0	-2.688928	5.120225	-1.039371
49	8	0	5.529505	-1.908767	-2.323203
50	6	0	6.907757	-2.361624	-2.412625
51	6	0	-2.384433	-3.055415	0.106417
52	6	0	-1.650628	-4.112581	-0.320403
53	7	0	-0.298847	-3.936841	-0.699337
54	8	0	0.135638	-2.768130	-0.905285
55	6	0	-3.769354	-3.087853	0.520430
56	6	0	-4.594417	-4.226959	0.401613
57	6	0	-5.928912	-4.164059	0.781644
58	6	0	-6.462562	-2.971777	1.289918
59	6	0	-5.656003	-1.837704	1.418372
60	6	0	-4.321879	-1.897035	1.032158
61	8	0	0.426059	-4.927469	-0.809619
62	1	0	1.863646	-2.763085	-0.576941
63	1	0	3.266131	-2.972621	0.351127
64	1	0	-0.902354	0.902104	-2.386532
65	1	0	-1.279861	3.228440	-2.188610
66	1	0	-1.457172	2.862615	0.837882
67	1	0	-3.657107	2.888763	-1.245333
68	1	0	1.268324	0.660790	-1.761409
69	1	0	2.239170	0.912794	0.713029
70	1	0	1.015947	-1.518821	0.892833
71	1	0	3.004275	-1.750164	-2.142663
72	1	0	3.303989	0.475100	-1.776100
73	1	0	0.577574	3.528516	1.009983
74	1	0	3.005478	3.908874	1.161340
75	1	0	4.426539	3.804711	-0.872492
76	1	0	3.367585	3.409078	-3.093357
77	1	0	0.925765	3.025282	-3.250425
78	1	0	6.954777	-3.443787	-2.279630
79	1	0	7.237892	-2.072397	-3.408793
80	1	0	7.499030	-1.861219	-1.642516
81	1	0	0.967663	-0.138903	2.919028
82	1	0	1.807961	0.015258	5.252377
83	1	0	3.974311	-1.045903	5.865904
84	1	0	5.294301	-2.242884	4.131949
85	1	0	4.506802	-2.344729	1.817261
86	1	0	5.646246	0.652994	-2.226747
87	1	0	7.740270	1.704696	-1.459902
88	1	0	8.054987	2.245551	0.952156
89	1	0	6.231455	1.735003	2.567956
90	1	0	4.157024	0.690913	1.810659
91	1	0	-5.625066	3.262839	-0.096790
92	1	0	-7.074376	2.994112	1.894650
93	1	0	-6.267915	1.691894	3.854806
94	1	0	-4.001511	0.667287	3.808221
95	1	0	-2.554786	0.938899	1.802793
96	1	0	-1.622601	-1.140906	-2.182021
97	1	0	-5.209681	0.092039	-0.792146
98	1	0	-5.000900	1.371165	-1.966738
99	1	0	-3.070221	-3.050512	-2.730875
100	1	0	-3.249990	-1.964881	-4.092483
101	1	0	-6.536448	-0.485671	-2.733667
102	1	0	-5.212565	-0.136909	-3.844106
103	1	0	-5.494164	-2.618813	-3.507173
104	1	0	-5.204225	-2.400155	-1.781964
105	1	0	-1.889404	-2.090151	0.156583
106	1	0	-1.954958	-5.148591	-0.351921

107	1	0	-4.198062	-5.153173	-0.003145
108	1	0	-6.560787	-5.041166	0.680514
109	1	0	-7.508130	-2.930936	1.581413
110	1	0	-6.058361	-0.908427	1.810238
111	1	0	-3.698832	-1.013548	1.107213

(*Re*,*Si*)–**X$_L$X$_L$–TS2a**

Center Number	Atomic Number	Atomic Type	Coordinates (Angstroms) X	Y	Z
1	6	0	-6.074186	-3.299249	1.548907
2	6	0	-5.656586	-4.124316	0.500477
3	6	0	-4.454140	-3.872826	-0.158324
4	6	0	-3.644746	-2.788158	0.220176
5	6	0	-4.070172	-1.978447	1.284069
6	6	0	-5.274638	-2.224012	1.941144
7	6	0	-2.359771	-2.460110	-0.452174
8	6	0	-2.682853	-1.252360	-1.991191
9	6	0	-3.305228	-0.082380	-1.463332
10	6	0	-4.809618	0.001048	-1.445016
11	6	0	-5.425100	-0.563385	-2.740896
12	6	0	-4.938637	-1.987333	-3.003311
13	6	0	-3.407913	-2.028097	-3.089466
14	7	0	-2.586923	0.928226	-0.930016
15	6	0	-1.169796	1.158598	-1.252808
16	6	0	-1.057450	2.719898	-1.255907
17	6	0	-1.969162	3.046402	-0.074473
18	6	0	-3.193783	2.138133	-0.277656
19	6	0	-0.195762	0.563508	-0.219136
20	7	0	1.065769	0.415366	-0.677086
21	6	0	2.198074	0.134233	0.202484
22	6	0	2.063569	-1.296340	0.789581
23	7	0	2.597944	-2.144322	-0.342090
24	6	0	3.494691	-1.334014	-1.233796
25	6	0	3.477164	0.137437	-0.668508
26	6	0	0.366492	3.233188	-1.243999
27	6	0	1.040936	3.577888	-0.065849
28	6	0	2.409842	3.853524	-0.090570
29	6	0	3.121661	3.793262	-1.290287
30	6	0	2.448402	3.490431	-2.477263
31	6	0	1.078958	3.218771	-2.452663
32	7	0	-2.353381	4.504014	-0.073746
33	8	0	-3.077801	4.892778	-0.986403
34	6	0	-3.980923	1.902703	0.995006
35	6	0	-5.296585	2.371853	1.079395
36	6	0	-6.028196	2.220848	2.258141
37	6	0	-5.445510	1.602504	3.365283
38	6	0	-4.126909	1.145067	3.290982
39	6	0	-3.394219	1.296872	2.114015
40	6	0	2.692634	-1.517025	2.145671
41	6	0	2.010595	-0.945834	3.231776
42	6	0	2.524305	-1.057358	4.521676
43	6	0	3.715387	-1.755393	4.743127
44	6	0	4.386325	-2.335588	3.667719
45	6	0	3.882541	-2.215106	2.369621
46	6	0	4.859091	-2.002440	-1.330688
47	8	0	5.553745	-1.492211	-2.340522
48	6	0	6.920410	-1.962962	-2.474490
49	6	0	4.756161	0.655630	-0.020438
50	6	0	5.771811	1.116696	-0.875998
51	6	0	6.963613	1.628799	-0.367541
52	6	0	7.157480	1.706769	1.013948
53	6	0	6.146480	1.278457	1.872169
54	6	0	4.953835	0.760269	1.360957
55	8	0	-0.524493	0.331321	0.943631
56	8	0	-1.882020	5.194228	0.819790
57	8	0	5.227492	-2.888959	-0.588747
58	1	0	1.709862	-2.402731	-0.897848
59	1	0	3.077731	-2.993858	-0.023589
60	1	0	-0.947958	0.763310	-2.246615
61	1	0	-1.564887	3.072644	-2.161930
62	1	0	-1.495930	2.862450	0.886963
63	1	0	-3.834123	2.633059	-1.014583

64	1	0	1.270720	0.746328	-1.609622
65	1	0	2.228822	0.881100	0.998789
66	1	0	1.004906	-1.542168	0.856367
67	1	0	3.041895	-1.344451	-2.228028
68	1	0	3.304068	0.799623	-1.522390
69	1	0	0.508221	3.635747	0.877438
70	1	0	2.921853	4.112181	0.831465
71	1	0	4.189014	3.991147	-1.300543
72	1	0	2.984866	3.475673	-3.421884
73	1	0	0.559040	2.983206	-3.379471
74	1	0	6.935915	-3.052035	-2.545207
75	1	0	7.291585	-1.504408	-3.389563
76	1	0	7.501264	-1.635013	-1.609428
77	1	0	1.078668	-0.414264	3.052700
78	1	0	1.990834	-0.609538	5.355111
79	1	0	4.113488	-1.848458	5.749236
80	1	0	5.312372	-2.878966	3.830425
81	1	0	4.449193	-2.640492	1.548445
82	1	0	5.625502	1.071298	-1.951229
83	1	0	7.733779	1.979054	-1.048988
84	1	0	8.083222	2.108964	1.414816
85	1	0	6.277371	1.344912	2.948252
86	1	0	4.188411	0.444768	2.057222
87	1	0	-5.752230	2.864577	0.223403
88	1	0	-7.048267	2.589373	2.310593
89	1	0	-6.011933	1.486393	4.284798
90	1	0	-3.665668	0.671139	4.152914
91	1	0	-2.372674	0.936255	2.053281
92	1	0	-1.616361	-1.164891	-2.183926
93	1	0	-5.169307	-0.573889	-0.579941
94	1	0	-5.149211	1.025024	-1.293366
95	1	0	-3.040645	-3.061803	-3.083704
96	1	0	-3.092905	-1.605308	-4.054443
97	1	0	-6.516162	-0.531631	-2.650255
98	1	0	-5.155984	0.090211	-3.581750
99	1	0	-5.367442	-2.372404	-3.935254
100	1	0	-5.291618	-2.636728	-2.197077
101	1	0	-1.770051	-1.722790	0.088571
102	6	0	-1.593916	-3.561321	-0.954535
103	1	0	-1.956108	-4.574665	-1.031990
104	1	0	-4.155778	-4.517349	-0.979694
105	1	0	-6.270229	-4.966477	0.193900
106	1	0	-7.013561	-3.497467	2.056749
107	1	0	-5.582774	-1.570722	2.752428
108	1	0	-3.452272	-1.143156	1.592651
109	7	0	-0.295372	-3.416104	-1.293301
110	8	0	0.454348	-4.386239	-1.536831
111	8	0	0.199640	-2.204101	-1.342991

(2R,1'S)–$\mathbf{X_LX_L}$–**INT3a**

Center Number	Atomic Number	Atomic Type	Coordinates (Angstroms) X	Y	Z
1	6	0	4.881939	0.488091	1.659552
2	6	0	4.700894	0.731147	0.293458
3	6	0	5.714467	1.410886	-0.403787
4	6	0	6.888429	1.803189	0.235475
5	6	0	7.065830	1.536283	1.595425
6	6	0	6.056686	0.885674	2.303027
7	6	0	3.450678	0.349562	-0.489283
8	6	0	2.145399	0.145298	0.318931
9	6	0	2.009526	-1.382038	0.566460
10	7	0	2.561290	-1.934322	-0.720143
11	6	0	3.516458	-0.970601	-1.351672
12	7	0	1.039951	0.605027	-0.520302
13	6	0	-0.241011	0.626603	-0.113105
14	8	0	-0.608778	0.256538	1.001234
15	6	0	2.605046	-1.899141	1.855196
16	6	0	1.915766	-1.549385	3.027483
17	6	0	2.397230	-1.947238	4.272324
18	6	0	3.563168	-2.714241	4.358875
19	6	0	4.238793	-3.077359	3.194905
20	6	0	3.766641	-2.670192	1.943878
21	6	0	4.893244	-1.600737	-1.507612

22	8	0	5.235497	-2.630925	-0.968174
23	6	0	-1.207797	1.252973	-1.140601
24	7	0	-2.634647	0.979946	-0.830621
25	6	0	-3.247801	2.155480	-0.097646
26	6	0	-2.033401	3.072091	0.120025
27	6	0	-1.145878	2.814007	-1.096663
28	6	0	-3.329558	-0.030720	-1.319965
29	6	0	-2.662468	-1.193643	-1.961371
30	6	0	-3.445727	-1.769795	-3.165433
31	6	0	-4.970140	-1.803260	-3.015032
32	6	0	-5.487876	-0.447940	-2.539932
33	6	0	-4.825107	-0.043940	-1.202570
34	6	0	0.259075	3.376351	-1.112375
35	6	0	0.934265	3.768594	0.049439
36	6	0	2.286457	4.112655	-0.004132
37	6	0	2.978477	4.071648	-1.216086
38	6	0	2.302590	3.715329	-2.386865
39	6	0	0.949327	3.376707	-2.333940
40	7	0	-2.450576	4.519553	0.194400
41	8	0	-1.962905	5.184428	1.097178
42	6	0	-4.011555	1.846334	1.170935
43	6	0	-5.282630	2.409525	1.340556
44	6	0	-5.997259	2.192849	2.518892
45	6	0	-5.445980	1.409111	3.533974
46	6	0	-4.172796	0.857040	3.372386
47	6	0	-3.451253	1.078829	2.199363
48	8	0	-3.218742	4.923899	-0.674749
49	8	0	5.638129	-0.871332	-2.333772
50	6	0	7.016992	-1.294665	-2.484474
51	6	0	-2.249573	-2.303896	-0.817251
52	6	0	-1.380492	-3.353986	-1.416637
53	7	0	-0.138584	-3.086385	-1.794685
54	8	0	0.254534	-1.805715	-1.802750
55	6	0	-3.420524	-2.891574	-0.056799
56	6	0	-4.169236	-3.977235	-0.533782
57	6	0	-5.261662	-4.461624	0.187149
58	6	0	-5.625595	-3.869518	1.398897
59	6	0	-4.879580	-2.797793	1.891912
60	6	0	-3.782261	-2.323434	1.172187
61	8	0	0.713559	-3.957020	-2.112533
62	1	0	1.652653	-1.992666	-1.358557
63	1	0	2.964593	-2.872401	-0.632610
64	1	0	-0.972173	0.886687	-2.139710
65	1	0	-1.691166	3.179712	-1.975808
66	1	0	-1.536918	2.851865	1.062907
67	1	0	-3.904591	2.663099	-0.810074
68	1	0	1.256100	0.946041	-1.446338
69	1	0	2.134984	0.700468	1.258411
70	1	0	0.949750	-1.637158	0.556708
71	1	0	3.138504	-0.764344	-2.356984
72	1	0	3.287589	1.176515	-1.187085
73	1	0	0.415225	3.815964	1.000846
74	1	0	2.800069	4.410724	0.904850
75	1	0	4.032761	4.329132	-1.249233
76	1	0	2.823260	3.711779	-3.340243
77	1	0	0.426040	3.102905	-3.248345
78	1	0	7.056560	-2.338214	-2.802246
79	1	0	7.436727	-0.636988	-3.244057
80	1	0	7.538981	-1.173508	-1.532489
81	1	0	1.002268	-0.963218	2.952046
82	1	0	1.858788	-1.668366	5.173539
83	1	0	3.937068	-3.029818	5.328473
84	1	0	5.143984	-3.674617	3.252630
85	1	0	4.331890	-2.934573	1.057654
86	1	0	5.582754	1.626263	-1.460317
87	1	0	7.658295	2.326612	-0.324539
88	1	0	7.977990	1.843176	2.098753
89	1	0	6.175450	0.681931	3.363176
90	1	0	4.117139	-0.009551	2.240563
91	1	0	-5.714032	3.023416	0.552779
92	1	0	-6.981372	2.634943	2.641324
93	1	0	-6.002748	1.236203	4.450264
94	1	0	-3.734859	0.256677	4.164841
95	1	0	-2.457890	0.662226	2.076500
96	1	0	-1.667143	-0.918151	-2.310872
97	1	0	-5.078927	-0.787873	-0.434404
98	1	0	-5.220627	0.908069	-0.856684

99	1	0	-3.041224	-2.767184	-3.370353
100	1	0	-3.196734	-1.155024	-4.040730
101	1	0	-6.571804	-0.467170	-2.386344
102	1	0	-5.286366	0.325760	-3.293204
103	1	0	-5.423076	-2.065709	-3.977691
104	1	0	-5.272622	-2.571879	-2.298616
105	1	0	-1.652001	-1.711415	-0.117686
106	1	0	-1.646786	-4.398968	-1.455455
107	1	0	-3.906305	-4.446143	-1.477637
108	1	0	-5.828560	-5.303998	-0.198906
109	1	0	-6.477974	-4.246667	1.956386
110	1	0	-5.145653	-2.329253	2.835393
111	1	0	-3.201568	-1.494848	1.565026

(*Re*,*Re*)–**X_LX_L–TS2a**

Center Number	Atomic Number	Atomic Type	Coordinates (Angstroms) X	Y	Z
1	6	0	5.265201	-0.053866	1.024874
2	6	0	4.405581	0.830406	1.686556
3	6	0	4.875984	1.487409	2.834824
4	6	0	6.176787	1.287746	3.293382
5	6	0	7.031364	0.415831	2.614140
6	6	0	6.568421	-0.257357	1.484413
7	6	0	2.986590	1.134418	1.221810
8	6	0	2.264164	0.006299	0.440412
9	6	0	2.349228	0.357539	-1.077473
10	7	0	2.482827	1.868069	-1.079074
11	6	0	2.844975	2.387349	0.283619
12	7	0	0.871053	-0.087415	0.876360
13	6	0	0.156259	-1.179542	0.513003
14	8	0	0.655477	-2.131313	-0.083588
15	6	0	3.397695	-0.362451	-1.900087
16	6	0	3.077616	-1.663711	-2.316441
17	6	0	3.983122	-2.399295	-3.077779
18	6	0	5.213016	-1.843960	-3.440649
19	6	0	5.534316	-0.550778	-3.029709
20	6	0	4.633679	0.189225	-2.259027
21	6	0	4.086575	3.255227	0.153866
22	8	0	4.780574	3.283742	-0.844201
23	6	0	-1.328172	-1.185483	0.937060
24	7	0	-2.220114	-0.803314	-0.187936
25	6	0	-2.248920	-1.977967	-1.119626
26	6	0	-1.950405	-3.195214	-0.188430
27	6	0	-1.845088	-2.614773	1.234497
28	6	0	-3.306382	-0.013671	-0.017703
29	6	0	-3.525678	0.817792	1.101237
30	6	0	-4.952981	0.879177	1.647865
31	6	0	-6.073705	0.753172	0.603800
32	6	0	-5.739923	-0.277572	-0.479923
33	6	0	-4.362494	0.053801	-1.094010
34	6	0	-1.128746	-3.416780	2.298787
35	6	0	0.024352	-4.175949	2.057205
36	6	0	0.632806	-4.880990	3.096171
37	6	0	0.105593	-4.834983	4.387906
38	6	0	-1.042127	-4.081373	4.638601
39	6	0	-1.654134	-3.382756	3.598960
40	7	0	-3.102293	-4.174732	-0.272604
41	8	0	-2.845729	-5.303980	-0.665798
42	6	0	-1.326498	-1.825427	-2.315598
43	6	0	-0.954390	-2.954455	-3.055286
44	6	0	-0.130683	-2.820744	-4.173037
45	6	0	0.324235	-1.559887	-4.567674
46	6	0	-0.064604	-0.431434	-3.845619
47	6	0	-0.891645	-0.563093	-2.727906
48	8	0	-4.218307	-3.759898	0.045417
49	8	0	4.281393	3.963326	1.257295
50	6	0	5.518087	4.719084	1.320664
51	6	0	-2.949419	2.849488	0.347289
52	6	0	-4.223773	3.567952	0.246903
53	6	0	-4.780236	4.117503	1.418986
54	6	0	-5.982016	4.819969	1.382519
55	6	0	-6.651814	4.992365	0.169233
56	6	0	-6.103580	4.472301	-1.008216

57	6	0	-4.902443	3.771130	-0.972852
58	1	0	1.521893	2.244959	-1.339250
59	1	0	3.182134	2.196032	-1.757217
60	1	0	-1.469609	-0.523261	1.786764
61	1	0	-2.877782	-2.459830	1.568808
62	1	0	-1.069313	-3.745724	-0.492845
63	1	0	-3.270797	-2.100329	-1.470636
64	1	0	0.370324	0.794053	1.023329
65	1	0	2.718678	-0.967640	0.617105
66	1	0	1.377784	0.153674	-1.529030
67	1	0	2.004924	2.996245	0.620779
68	1	0	2.399710	1.348670	2.118568
69	1	0	0.458175	-4.208050	1.065249
70	1	0	1.521935	-5.470820	2.892359
71	1	0	0.581431	-5.388614	5.192201
72	1	0	-1.467818	-4.046537	5.637337
73	1	0	-2.559300	-2.811889	3.797177
74	1	0	5.605620	5.370726	0.449369
75	1	0	5.451626	5.298411	2.240077
76	1	0	6.360740	4.024914	1.357945
77	1	0	2.121398	-2.094282	-2.036835
78	1	0	3.722571	-3.404945	-3.395085
79	1	0	5.915655	-2.416389	-4.039242
80	1	0	6.489795	-0.111402	-3.300809
81	1	0	4.929758	1.178160	-1.921566
82	1	0	4.218509	2.168667	3.368150
83	1	0	6.518262	1.803614	4.186406
84	1	0	8.044728	0.255454	2.970501
85	1	0	7.218268	-0.947159	0.953768
86	1	0	4.933570	-0.595158	0.147863
87	1	0	-1.303179	-3.941840	-2.762019
88	1	0	0.157341	-3.704764	-4.734484
89	1	0	0.975230	-1.460350	-5.430938
90	1	0	0.273032	0.558138	-4.140525
91	1	0	-1.172674	0.323802	-2.172287
92	1	0	-2.779125	0.795272	1.889433
93	1	0	-4.379025	1.087062	-1.462644
94	1	0	-4.142151	-0.569380	-1.959452
95	1	0	-5.092361	1.791836	2.235682
96	1	0	-5.034696	0.047678	2.364942
97	1	0	-6.498538	-0.267443	-1.269836
98	1	0	-5.713306	-1.295100	-0.069886
99	1	0	-7.006262	0.483594	1.111712
100	1	0	-6.244321	1.723422	0.128377
101	1	0	-2.424871	2.968004	1.289117
102	1	0	-4.490651	3.389145	-1.901638
103	1	0	-6.612271	4.621129	-1.956082
104	1	0	-7.588706	5.540585	0.137305
105	1	0	-6.391374	5.237625	2.297512
106	1	0	-4.251576	4.002370	2.361936
107	6	0	-2.096256	2.737858	-0.774177
108	1	0	-2.408800	2.820026	-1.803648
109	7	0	-0.774798	2.532630	-0.621688
110	8	0	-0.046214	2.442360	-1.679272
111	8	0	-0.239508	2.451234	0.538471

(*Re*,*Re*)–$\mathbf{X_LX_L}$**–TS2a**

Center Number	Atomic Number	Atomic Type	Coordinates (Angstroms) X	Y	Z
1	6	0	-5.004353	-0.246278	-1.465156
2	6	0	-4.174847	0.845848	-1.741946
3	6	0	-4.652379	1.847216	-2.603465
4	6	0	-5.934344	1.775276	-3.145670
5	6	0	-6.760971	0.690116	-2.844623
6	6	0	-6.288183	-0.321372	-2.010041
7	6	0	-2.775182	1.014616	-1.161905
8	6	0	-2.048237	-0.281955	-0.722938
9	6	0	-2.223843	-0.400075	0.828700
10	7	0	-2.503825	1.016227	1.290934
11	6	0	-2.688777	1.927316	0.114651
12	7	0	-0.635275	-0.189833	-1.105379
13	6	0	0.185704	-1.256846	-0.907425
14	8	0	-0.214240	-2.381824	-0.631192

15	6	0	-3.251437	-1.399385	1.324341
16	6	0	-2.848530	-2.741884	1.380852
17	6	0	-3.730769	-3.722826	1.829139
18	6	0	-5.022251	-3.376733	2.233915
19	6	0	-5.429615	-2.044154	2.176337
20	6	0	-4.551709	-1.058065	1.719977
21	6	0	-3.929277	2.772635	0.369173
22	8	0	-4.773015	2.468600	1.188648
23	6	0	1.700792	-0.940737	-1.022403
24	7	0	2.217507	-0.664010	0.335863
25	6	0	2.442250	-1.972262	1.038372
26	6	0	2.604150	-2.973500	-0.144986
27	6	0	2.583100	-2.134969	-1.436652
28	6	0	2.551321	0.519718	0.820416
29	6	0	2.937194	1.691410	-0.056967
30	6	0	4.492248	1.813784	0.063588
31	6	0	5.014976	1.937406	1.494112
32	6	0	4.544620	0.748173	2.330110
33	6	0	3.002318	0.626328	2.249737
34	6	0	2.258484	-2.835679	-2.739077
35	6	0	1.263637	-3.815334	-2.863102
36	6	0	1.016534	-4.413481	-4.098907
37	6	0	1.751805	-4.041340	-5.225698
38	6	0	2.744603	-3.067268	-5.111703
39	6	0	2.996778	-2.474278	-3.875140
40	7	0	3.929939	-3.694872	-0.011693
41	8	0	3.894067	-4.912713	0.097520
42	6	0	1.394386	-2.404606	2.058153
43	6	0	1.393567	-3.744767	2.470056
44	6	0	0.498802	-4.183563	3.446032
45	6	0	-0.400605	-3.289988	4.030566
46	6	0	-0.391368	-1.953116	3.635778
47	6	0	0.502914	-1.509070	2.658696
48	8	0	4.944762	-2.999050	-0.009975
49	8	0	-3.951490	3.836586	-0.421059
50	6	0	-5.164782	4.633109	-0.377388
51	6	0	2.102185	3.055855	0.135369
52	6	0	2.928346	4.278225	-0.225121
53	6	0	3.076897	4.620805	-1.576468
54	6	0	3.859458	5.709834	-1.959588
55	6	0	4.503003	6.482292	-0.990548
56	6	0	4.355440	6.158104	0.358639
57	6	0	3.575588	5.064069	0.737487
58	1	0	-1.710172	1.417164	1.881742
59	1	0	-3.362369	1.066589	1.855536
60	1	0	1.851240	-0.088610	-1.681768
61	1	0	3.588242	-1.706368	-1.525801
62	1	0	1.840909	-3.742371	-0.132325
63	1	0	3.409992	-1.880663	1.537144
64	1	0	-0.204082	0.709482	-0.901227
65	1	0	-2.453965	-1.168695	-1.207986
66	1	0	-1.261862	-0.667303	1.252702
67	1	0	-1.811266	2.571697	0.062654
68	1	0	-2.172131	1.497231	-1.936031
69	1	0	0.670561	-4.103047	-2.003682
70	1	0	0.246656	-5.175554	-4.178797
71	1	0	1.557242	-4.512703	-6.184687
72	1	0	3.330298	-2.777415	-5.979235
73	1	0	3.785267	-1.729168	-3.789617
74	1	0	-5.369765	4.946929	0.647671
75	1	0	-4.966569	5.490129	-1.018948
76	1	0	-5.996707	4.038942	-0.762565
77	1	0	-1.844284	-3.011064	1.070115
78	1	0	-3.403873	-4.757738	1.870687
79	1	0	-5.706944	-4.141055	2.589645
80	1	0	-6.434219	-1.764614	2.479536
81	1	0	-4.920079	-0.037737	1.644191
82	1	0	-4.014414	2.692124	-2.847858
83	1	0	-6.280971	2.558011	-3.814511
84	1	0	-7.759031	0.628912	-3.268329
85	1	0	-6.914839	-1.177830	-1.779001
86	1	0	-4.665107	-1.051495	-0.826113
87	1	0	2.091088	-4.455967	2.034755
88	1	0	0.509147	-5.226315	3.749218
89	1	0	-1.100908	-3.632972	4.786000
90	1	0	-1.085862	-1.247104	4.082198
91	1	0	0.482047	-0.467438	2.359033

92	1	0	2.758639	1.433832	-1.101638
93	1	0	2.564818	1.549996	2.648618
94	1	0	2.638483	-0.196566	2.861933
95	1	0	4.807518	2.661762	-0.549246
96	1	0	4.925250	0.913384	-0.395114
97	1	0	4.827950	0.854576	3.382081
98	1	0	5.009777	-0.176671	1.962980
99	1	0	6.109250	1.987960	1.480231
100	1	0	4.660685	2.870616	1.945227
101	1	0	1.338672	2.947139	-0.649630
102	1	0	3.472147	4.829845	1.793140
103	1	0	4.845737	6.757442	1.120336
104	1	0	5.109481	7.333745	-1.284669
105	1	0	3.959843	5.959265	-3.012015
106	1	0	2.572563	4.028551	-2.337799
107	6	0	1.312405	3.242683	1.399800
108	1	0	1.298899	4.146564	1.990202
109	7	0	0.366232	2.366364	1.646185
110	8	0	-0.555630	2.475305	2.538655
111	8	0	0.358384	1.270563	0.913297

X_LX_L–INT1b’·13a

Center Number	Atomic Number	Atomic Type	Coordinates (Angstroms) X	Y	Z
1	6	0	-5.334038	-1.552553	2.029309
2	6	0	-5.536413	-2.075701	0.742576
3	6	0	-6.448276	-3.118719	0.553078
4	6	0	-7.155278	-3.635996	1.639546
5	6	0	-6.946736	-3.118455	2.918437
6	6	0	-6.034039	-2.077596	3.113069
7	6	0	-4.797210	-1.508945	-0.438483
8	7	0	-3.294232	-1.576598	-0.259592
9	6	0	-2.645863	-0.517392	-1.112270
10	6	0	-3.818846	0.371927	-1.641845
11	6	0	-4.983608	-0.003360	-0.711281
12	6	0	-1.709900	0.218789	-0.144202
13	8	0	-2.036599	0.266588	1.049599
14	7	0	-6.312629	0.239030	-1.386430
15	8	0	-7.045303	1.074633	-0.882114
16	6	0	-3.439512	1.833937	-1.764962
17	6	0	-3.009145	2.311122	-3.009016
18	6	0	-2.497308	3.603824	-3.136673
19	6	0	-2.411684	4.433810	-2.017964
20	6	0	-2.858572	3.972710	-0.777547
21	6	0	-3.371655	2.682799	-0.650740
22	7	0	-0.622355	0.784067	-0.664304
23	6	0	0.378815	1.456383	0.167289
24	6	0	1.272647	0.432710	0.923502
25	7	0	2.483118	0.359444	0.110389
26	6	0	2.662118	1.485535	-0.790121
27	6	0	1.309990	2.297621	-0.741101
28	6	0	3.393042	-0.687555	0.264782
29	6	0	3.181657	-1.707096	1.140829
30	6	0	4.222371	-2.726799	1.532967
31	6	0	5.498600	-2.703799	0.675736
32	6	0	5.837471	-1.285095	0.213094
33	6	0	4.664698	-0.667552	-0.564605
34	6	0	1.476483	0.781202	2.401048
35	6	0	0.373293	0.694561	3.262537
36	6	0	0.504834	1.009964	4.614528
37	6	0	1.742423	1.408390	5.125487
38	6	0	2.843768	1.487385	4.273096
39	6	0	2.715338	1.172457	2.917737
40	6	0	1.405558	3.754491	-0.320376
41	6	0	1.160989	4.758148	-1.265335
42	6	0	1.238438	6.105866	-0.912998
43	6	0	1.565036	6.467157	0.395715
44	6	0	1.810593	5.472876	1.345045
45	6	0	1.730470	4.125149	0.991766
46	6	0	3.847645	2.404589	-0.459928
47	8	0	4.476138	2.425552	0.573799
48	8	0	4.075513	3.210005	-1.508530
49	6	0	5.019775	4.278593	-1.278836

50	8	0	-6.541965	-0.421609	-2.397991
51	6	0	1.881632	-3.621224	-1.133941
52	6	0	0.734286	-4.118711	-0.599478
53	7	0	-0.410284	-3.304370	-0.540277
54	8	0	-1.508735	-3.840015	-0.248919
55	6	0	3.092989	-4.377696	-1.367822
56	6	0	3.424432	-5.533260	-0.630996
57	6	0	4.595757	-6.228757	-0.907850
58	6	0	5.443888	-5.797980	-1.934656
59	6	0	5.123928	-4.658786	-2.679290
60	6	0	3.966162	-3.945610	-2.388249
61	8	0	-0.318549	-2.084473	-0.792593
62	1	0	-3.061560	-1.278386	0.709948
63	1	0	-2.098300	-0.993017	-1.922304
64	1	0	-4.088708	-0.011959	-2.631369
65	1	0	-5.009434	0.575758	0.209827
66	1	0	-5.037838	-2.051400	-1.355670
67	1	0	-0.382977	0.564188	-1.621476
68	1	0	-0.169129	2.081665	0.871511
69	1	0	0.766712	-0.540207	0.874568
70	1	0	2.830165	1.140135	-1.817855
71	1	0	0.896161	2.299728	-1.753709
72	1	0	5.090757	4.812242	-2.225978
73	1	0	4.642349	4.935810	-0.491126
74	1	0	5.990437	3.872767	-0.984695
75	1	0	-3.071138	1.667312	-3.883928
76	1	0	-2.170458	3.960455	-4.109244
77	1	0	-2.001425	5.434803	-2.108304
78	1	0	-2.799846	4.615814	0.095289
79	1	0	-3.678045	2.331631	0.329499
80	1	0	0.911115	4.482295	-2.287009
81	1	0	1.047096	6.871620	-1.659860
82	1	0	1.626985	7.515414	0.673753
83	1	0	2.065264	5.742839	2.365908
84	1	0	1.929821	3.365059	1.740104
85	1	0	-0.591187	0.390914	2.866691
86	1	0	-0.359628	0.943287	5.269910
87	1	0	1.846047	1.653163	6.178958
88	1	0	3.811890	1.794607	4.659216
89	1	0	3.571917	1.250777	2.260739
90	1	0	-6.612817	-3.520642	-0.443433
91	1	0	-7.866077	-4.441961	1.485245
92	1	0	-7.495858	-3.522213	3.763739
93	1	0	-5.873464	-1.670924	4.106836
94	1	0	-4.634984	-0.733359	2.191928
95	1	0	2.294499	-1.703196	1.764877
96	1	0	4.501783	-1.233562	-1.494436
97	1	0	4.938062	0.343992	-0.865500
98	1	0	3.790446	-3.737962	1.525167
99	1	0	4.482413	-2.534223	2.585950
100	1	0	6.732746	-1.291778	-0.419651
101	1	0	6.056614	-0.643061	1.075904
102	1	0	6.333040	-3.131033	1.243633
103	1	0	5.366055	-3.337824	-0.205454
104	1	0	1.851177	-2.602317	-1.505356
105	1	0	-2.850223	-2.512576	-0.382100
106	1	0	5.782266	-4.323833	-3.474913
107	1	0	3.717718	-3.055283	-2.959595
108	1	0	2.778829	-5.863324	0.177320
109	1	0	4.853196	-7.106708	-0.323419
110	1	0	6.355820	-6.347267	-2.148928
111	1	0	0.556139	-5.134855	-0.279382

(*Re*,*Si*)–**$X_L$$X_L$–TS2b'**

Center Number	Atomic Number	Atomic Type	Coordinates (Angstroms) X	Y	Z
1	7	0	-3.174345	-1.351446	-0.124784
2	6	0	-2.668265	-0.258208	-1.028416
3	6	0	-3.947900	0.464525	-1.565082
4	6	0	-5.060273	-0.049716	-0.633312
5	6	0	-4.663351	-1.502832	-0.316331
6	6	0	-1.748509	0.575678	-0.126809
7	8	0	-2.049075	0.699339	1.065211

8	7	0	-0.648753	1.107610	-0.689368
9	6	0	0.437642	1.608770	0.161723
10	6	0	1.248342	0.428354	0.772460
11	7	0	2.429399	0.317458	-0.105324
12	6	0	2.671627	1.525672	-0.904291
13	6	0	1.422347	2.459632	-0.679020
14	1	0	-3.000130	-1.001005	0.832517
15	6	0	3.972924	2.250127	-0.535933
16	8	0	4.629478	2.066108	0.463874
17	8	0	4.258380	3.134200	-1.495840
18	6	0	5.325129	4.065244	-1.194722
19	6	0	-3.845628	1.966548	-1.725619
20	6	0	-3.785093	2.509751	-3.014408
21	6	0	-3.668934	3.888032	-3.200691
22	6	0	-3.613337	4.738559	-2.096122
23	6	0	-3.677343	4.205598	-0.806504
24	6	0	-3.794245	2.829219	-0.620444
25	6	0	1.728192	3.823211	-0.079535
26	6	0	1.666082	4.958521	-0.896735
27	6	0	1.969206	6.221474	-0.387969
28	6	0	2.341904	6.364097	0.950255
29	6	0	2.405250	5.237826	1.773037
30	6	0	2.100235	3.975126	1.263260
31	7	0	-6.402012	-0.013834	-1.324061
32	8	0	-7.262344	0.697276	-0.829339
33	8	0	-6.516679	-0.702835	-2.336412
34	6	0	1.563810	0.564379	2.261507
35	6	0	0.487427	0.508075	3.159019
36	6	0	0.707219	0.606974	4.532138
37	6	0	2.004445	0.756655	5.028394
38	6	0	3.077173	0.811298	4.139086
39	6	0	2.861460	0.714333	2.761622
40	6	0	-5.328865	-2.138493	0.874618
41	6	0	-6.058245	-3.320229	0.713178
42	6	0	-6.692373	-3.907569	1.808945
43	6	0	-6.593073	-3.320399	3.070718
44	6	0	-5.862281	-2.140698	3.238151
45	6	0	-5.235749	-1.548161	2.144209
46	1	0	-2.103383	-0.719385	-1.835125
47	1	0	-4.158047	0.024150	-2.545078
48	1	0	-5.177237	0.550408	0.267718
49	1	0	-4.813490	-2.101252	-1.218095
50	1	0	-0.380579	0.724610	-1.588065
51	1	0	-0.028490	2.197982	0.949112
52	1	0	0.647118	-0.472960	0.625131
53	1	0	2.741242	1.262117	-1.963968
54	1	0	0.970294	2.639275	-1.657736
55	1	0	5.435626	4.675956	-2.089586
56	1	0	5.036415	4.679394	-0.338275
57	1	0	6.247494	3.525085	-0.971033
58	1	0	-3.839054	1.852093	-3.879082
59	1	0	-3.631048	4.294638	-4.207117
60	1	0	-3.528591	5.812028	-2.237932
61	1	0	-3.640456	4.862398	0.057654
62	1	0	-3.826440	2.430166	0.388599
63	1	0	1.385782	4.853096	-1.942104
64	1	0	1.912933	7.091791	-1.035618
65	1	0	2.577804	7.346278	1.349622
66	1	0	2.692365	5.337763	2.815782
67	1	0	2.163900	3.110400	1.915251
68	1	0	-0.522995	0.398549	2.776778
69	1	0	-0.136504	0.564379	5.215372
70	1	0	2.175488	0.831841	6.098430
71	1	0	4.090259	0.934793	4.511630
72	1	0	3.701541	0.788955	2.083269
73	1	0	-6.135723	-3.778377	-0.269419
74	1	0	-7.261158	-4.822629	1.675217
75	1	0	-7.085735	-3.778162	3.923324
76	1	0	-5.786920	-1.679577	4.218262
77	1	0	-4.679998	-0.622191	2.285837
78	6	0	3.223472	-0.773655	-0.113737
79	6	0	2.904646	-1.950030	0.597859
80	6	0	4.504741	-0.783789	-0.911652
81	6	0	4.036292	-2.736478	1.243753
82	1	0	2.012150	-1.921781	1.211887
83	6	0	5.670362	-1.257513	-0.018115
84	1	0	4.386629	-1.496335	-1.737333

85	1	0	4.738865	0.180221	-1.359087
86	6	0	5.386721	-2.665251	0.514301
87	1	0	3.737102	-3.780016	1.387630
88	1	0	4.148719	-2.316136	2.255197
89	1	0	6.596295	-1.246331	-0.603463
90	1	0	5.797309	-0.540008	0.801691
91	1	0	6.182022	-2.978191	1.200116
92	1	0	5.398412	-3.370709	-0.321969
93	6	0	1.989977	-3.212939	-1.025228
94	1	0	1.857055	-2.355490	-1.675002
95	6	0	3.056554	-4.145200	-1.412934
96	6	0	3.343440	-5.311137	-0.677985
97	6	0	3.809753	-3.883418	-2.573348
98	6	0	4.373401	-6.161457	-1.070347
99	6	0	4.834602	-4.738969	-2.970323
100	6	0	5.127650	-5.874965	-2.212212
101	1	0	-2.587671	-2.242935	-0.211332
102	1	0	5.400562	-4.522927	-3.871672
103	1	0	3.569721	-3.013709	-3.179775
104	1	0	2.766773	-5.547811	0.211001
105	1	0	4.588373	-7.051229	-0.486072
106	1	0	5.930722	-6.540252	-2.514998
107	6	0	0.789464	-3.711442	-0.475569
108	1	0	0.647783	-4.711169	-0.093958
109	7	0	-0.323339	-2.942973	-0.473168
110	8	0	-1.446249	-3.446522	-0.116367
111	8	0	-0.259499	-1.718352	-0.828156

(2R,1’S)–$\mathbf{X_LX_L}$**–INT3b**

Center Number	Atomic Number	Atomic Type	Coordinates (Angstroms) X	Y	Z
1	6	0	4.367252	-6.834941	-1.228112
2	6	0	3.778129	-6.575535	0.011428
3	6	0	3.013406	-5.424050	0.196301
4	6	0	2.833467	-4.507892	-0.850346
5	6	0	3.408191	-4.792679	-2.095916
6	6	0	4.173957	-5.944057	-2.284875
7	6	0	2.009825	-3.245271	-0.670678
8	6	0	0.625282	-3.604939	-0.216792
9	7	0	-0.357078	-2.794468	-0.494128
10	8	0	-0.220654	-1.639742	-1.014128
11	8	0	-1.617876	-3.248041	-0.220834
12	6	0	2.660551	-2.100572	0.270218
13	6	0	2.908649	-0.862413	-0.547375
14	6	0	3.991848	-0.997493	-1.574603
15	6	0	5.336031	-1.265608	-0.833418
16	6	0	5.225908	-2.514884	0.045557
17	6	0	4.009953	-2.446935	0.977978
18	7	0	2.306314	0.272303	-0.316984
19	6	0	1.248463	0.460387	0.721935
20	6	0	0.461549	1.703572	0.212315
21	6	0	1.433481	2.541020	-0.647631
22	6	0	2.492819	1.491144	-1.150684
23	7	0	-0.676427	1.283987	-0.597177
24	6	0	-1.703732	0.625848	-0.007933
25	8	0	-1.811738	0.538235	1.216495
26	6	0	1.781708	0.537799	2.143091
27	6	0	0.842739	0.409314	3.177538
28	6	0	1.255217	0.473856	4.507742
29	6	0	2.604605	0.661052	4.819195
30	6	0	3.538220	0.791524	3.791215
31	6	0	3.131734	0.731867	2.455474
32	6	0	3.915451	2.044035	-1.080768
33	8	0	4.143411	2.789365	-2.162231
34	6	0	5.373579	3.558974	-2.150705
35	6	0	2.020084	3.782387	0.011986
36	6	0	2.333015	4.876847	-0.808732
37	6	0	2.908012	6.030026	-0.276010
38	6	0	3.172822	6.109052	1.093123
39	6	0	2.851368	5.032180	1.919316
40	6	0	2.278950	3.877026	1.384207
41	6	0	-2.729259	-0.015744	-0.936602
42	7	0	-3.304615	-1.165962	-0.212400

43	6	0	-4.754677	-1.205013	-0.517618
44	6	0	-5.117155	0.296912	-0.620817
45	6	0	-3.926504	0.922217	-1.365240
46	6	0	-3.708809	2.405740	-1.162005
47	6	0	-3.512974	3.231315	-2.275486
48	6	0	-3.257805	4.594967	-2.118269
49	6	0	-3.195899	5.150648	-0.840053
50	6	0	-3.395052	4.336663	0.277886
51	6	0	-3.650669	2.975757	0.118917
52	7	0	-6.406167	0.485247	-1.376719
53	8	0	-6.438099	0.063198	-2.532699
54	6	0	-5.551810	-1.990560	0.493729
55	6	0	-6.346893	-3.062941	0.078638
56	6	0	-7.100628	-3.784887	1.005630
57	6	0	-7.060895	-3.441975	2.357347
58	6	0	-6.268459	-2.371631	2.780502
59	6	0	-5.521132	-1.648106	1.853355
60	8	0	4.697190	1.850898	-0.177728
61	8	0	-7.331200	1.021857	-0.782773
62	1	0	-3.185854	-0.958912	0.784521
63	1	0	-2.214422	-0.378884	-1.829241
64	1	0	-4.103319	0.737067	-2.428718
65	1	0	-5.291063	0.736861	0.360272
66	1	0	-4.882420	-1.638619	-1.514594
67	1	0	-0.541133	1.140253	-1.589014
68	1	0	0.071344	2.268671	1.057970
69	1	0	0.580663	-0.395171	0.633323
70	1	0	2.278338	1.223405	-2.187769
71	1	0	0.891662	2.890401	-1.530559
72	1	0	5.420970	4.036787	-3.127932
73	1	0	5.321268	4.303704	-1.353209
74	1	0	6.228631	2.898933	-1.992824
75	1	0	-3.571350	2.805557	-3.274838
76	1	0	-3.118408	5.222125	-2.994410
77	1	0	-3.003249	6.212244	-0.714275
78	1	0	-3.356324	4.762878	1.276379
79	1	0	-3.781667	2.352861	0.998797
80	1	0	2.128240	4.823679	-1.875036
81	1	0	3.135452	6.869292	-0.927132
82	1	0	3.616338	7.007214	1.512753
83	1	0	3.044405	5.085409	2.986730
84	1	0	2.040830	3.056311	2.049082
85	1	0	-0.205980	0.272049	2.930984
86	1	0	0.520987	0.373400	5.301835
87	1	0	2.923740	0.708287	5.856253
88	1	0	4.587811	0.949528	4.022287
89	1	0	3.862430	0.874949	1.668445
90	1	0	-6.380606	-3.331104	-0.974288
91	1	0	-7.717701	-4.613278	0.670249
92	1	0	-7.647594	-4.002589	3.079272
93	1	0	-6.238732	-2.096718	3.830893
94	1	0	-4.919820	-0.807289	2.195665
95	1	0	1.942381	-1.892882	1.061282
96	1	0	3.767207	-1.870085	-2.197407
97	1	0	4.076234	-0.136922	-2.238487
98	1	0	3.884569	-3.388515	1.517146
99	1	0	4.170505	-1.670975	1.735767
100	1	0	6.118499	-1.384426	-1.590064
101	1	0	5.587975	-0.383767	-0.236829
102	1	0	6.132512	-2.616616	0.652702
103	1	0	5.167790	-3.405545	-0.585727
104	1	0	1.875848	-2.792451	-1.659562
105	1	0	-2.261794	-2.443995	-0.316070
106	1	0	4.610836	-6.147962	-3.258132
107	1	0	3.248685	-4.113574	-2.930956
108	1	0	2.547955	-5.242626	1.162228
109	1	0	3.908844	-7.272746	0.833830
110	1	0	4.961670	-7.732190	-1.372212
111	1	0	0.360867	-4.551958	0.229927

(*Re*,*Re*)–$\mathbf{X_L X_L}$**–TS2b'**

Center Number	Atomic Number	Atomic Type	Coordinates (Angstroms) X	Y	Z

1	6	0	-4.074967	6.645771	-0.814207
2	6	0	-3.332596	6.545190	0.364603
3	6	0	-2.483641	5.459418	0.563001
4	6	0	-2.361946	4.446724	-0.408677
5	6	0	-3.094885	4.580823	-1.606288
6	6	0	-3.945387	5.664352	-1.802131
7	6	0	-1.459122	3.323453	-0.144819
8	6	0	-2.707157	1.909819	1.162326
9	6	0	-3.170007	1.027436	0.171871
10	6	0	-4.410853	1.427928	-0.588029
11	6	0	-5.507296	1.892822	0.389722
12	6	0	-5.005475	3.080280	1.215803
13	6	0	-3.697950	2.746151	1.950154
14	7	0	-2.503140	-0.102076	-0.141881
15	6	0	-1.356149	-0.601149	0.652939
16	6	0	-0.764089	-1.760628	-0.205234
17	6	0	-1.837124	-2.167584	-1.250202
18	6	0	-2.919802	-1.027192	-1.204862
19	7	0	0.467868	-1.375591	-0.888577
20	6	0	1.652041	-1.384640	-0.226670
21	8	0	1.825601	-1.819479	0.906445
22	6	0	-1.694365	-1.003408	2.088934
23	6	0	-0.622382	-1.170559	2.977600
24	6	0	-0.855159	-1.533693	4.303161
25	6	0	-2.160943	-1.729281	4.760049
26	6	0	-3.229077	-1.563909	3.878659
27	6	0	-3.000722	-1.200708	2.548203
28	6	0	-4.321884	-1.613841	-0.992875
29	8	0	-4.743077	-2.143333	-2.144133
30	6	0	-5.943051	-2.949104	-2.062856
31	6	0	-2.369140	-3.579469	-1.065091
32	6	0	-2.451380	-4.432747	-2.171969
33	6	0	-2.954582	-5.727200	-2.039358
34	6	0	-3.386806	-6.183497	-0.792405
35	6	0	-3.309996	-5.338904	0.317395
36	6	0	-2.803726	-4.045289	0.183740
37	6	0	2.792146	-0.690088	-0.976985
38	7	0	3.142219	0.515769	-0.162297
39	6	0	4.352327	1.109376	-0.829357
40	6	0	5.091530	-0.145984	-1.393778
41	6	0	4.170254	-1.374331	-1.147861
42	6	0	4.625634	-2.216458	0.031468
43	6	0	4.384004	-3.594936	0.002998
44	6	0	4.745857	-4.403348	1.078399
45	6	0	5.357519	-3.842638	2.201507
46	6	0	5.609646	-2.471436	2.238168
47	6	0	5.248797	-1.662876	1.159022
48	7	0	5.329736	0.083847	-2.870767
49	8	0	4.357283	-0.013345	-3.616284
50	6	0	5.145082	2.012850	0.089702
51	6	0	6.508611	2.230147	-0.149981
52	6	0	7.233174	3.096180	0.668679
53	6	0	6.605372	3.753526	1.728505
54	6	0	5.243376	3.554839	1.958153
55	6	0	4.511872	2.694039	1.138936
56	8	0	-4.944563	-1.616381	0.045470
57	8	0	6.467428	0.384924	-3.206872
58	1	0	2.290813	1.159901	-0.101357
59	1	0	3.356835	0.200635	0.793924
60	1	0	2.468887	-0.309064	-1.944131
61	1	0	4.137103	-1.993976	-2.046764
62	1	0	6.080371	-0.281780	-0.965604
63	1	0	3.937742	1.675717	-1.670956
64	1	0	0.393432	-0.704337	-1.651857
65	1	0	-0.506608	-2.594793	0.445421
66	1	0	-0.602948	0.190676	0.701943
67	1	0	-2.933361	-0.502930	-2.164466
68	1	0	-1.375386	-2.128192	-2.239634
69	1	0	-6.147982	-3.264189	-3.084917
70	1	0	-5.754358	-3.812417	-1.420094
71	1	0	-6.769009	-2.358915	-1.660179
72	1	0	3.904934	-4.038620	-0.866676
73	1	0	4.554804	-5.471715	1.037066
74	1	0	5.643801	-4.472633	3.038460
75	1	0	6.095164	-2.027848	3.102546
76	1	0	5.482286	-0.600385	1.208257
77	1	0	-2.124397	-4.079814	-3.147264

78	1	0	-3.005518	-6.377922	-2.907852
79	1	0	-3.776153	-7.191992	-0.685375
80	1	0	-3.641388	-5.685927	1.291878
81	1	0	-2.756442	-3.399145	1.055418
82	1	0	0.395195	-1.038272	2.622640
83	1	0	-0.014789	-1.662100	4.979843
84	1	0	-2.342141	-2.009649	5.793790
85	1	0	-4.248813	-1.718442	4.220663
86	1	0	-3.838904	-1.089677	1.871639
87	1	0	7.003538	1.743223	-0.985900
88	1	0	8.289442	3.256426	0.474961
89	1	0	7.172937	4.424379	2.366391
90	1	0	4.744545	4.073655	2.771423
91	1	0	3.444560	2.581651	1.314736
92	1	0	-1.854738	1.586827	1.748507
93	1	0	-4.143619	2.268899	-1.241494
94	1	0	-4.791469	0.640186	-1.234522
95	1	0	-3.219933	3.664983	2.307910
96	1	0	-3.926557	2.161624	2.855081
97	1	0	-6.400472	2.171023	-0.180107
98	1	0	-5.784066	1.049646	1.034514
99	1	0	-5.762620	3.382911	1.947683
100	1	0	-4.852117	3.935733	0.550606
101	1	0	-0.843848	3.416054	0.743123
102	1	0	-4.738872	7.490852	-0.970340
103	1	0	-4.503567	5.749463	-2.729782
104	1	0	-2.996510	3.841284	-2.394874
105	1	0	-1.894804	5.395614	1.473834
106	1	0	-3.410777	7.315341	1.126138
107	6	0	-0.862561	2.609126	-1.202437
108	1	0	-1.225083	2.595905	-2.219103
109	7	0	0.271118	1.900043	-1.032946
110	8	0	0.813346	1.311010	-2.021383
111	8	0	0.816246	1.811112	0.139803

(2R,1'R)–**X_LX_L–INT3b**

Center Number	Atomic Number	Atomic Type	Coordinates (Angstroms) X	Y	Z
1	6	0	4.359183	2.517703	1.085246
2	6	0	5.030911	1.806107	0.079934
3	6	0	6.403795	2.010165	-0.109482
4	6	0	7.101518	2.888737	0.719932
5	6	0	6.436889	3.571879	1.739476
6	6	0	5.064105	3.389918	1.914871
7	6	0	4.256605	0.882439	-0.835214
8	7	0	3.072539	0.259831	-0.153578
9	6	0	2.737142	-0.930879	-0.990211
10	6	0	4.121633	-1.601791	-1.158546
11	6	0	5.017307	-0.357784	-1.411513
12	6	0	1.574206	-1.639940	-0.293438
13	8	0	1.716071	-2.189029	0.792855
14	7	0	5.242202	-0.108374	-2.887794
15	8	0	6.333079	0.349615	-3.203864
16	6	0	4.588154	-2.422067	0.032651
17	6	0	4.353601	-3.802325	0.030882
18	6	0	4.722711	-4.589370	1.119059
19	6	0	5.335162	-4.006210	2.230685
20	6	0	5.581893	-2.634127	2.241208
21	6	0	5.214211	-1.846698	1.148162
22	7	0	0.386530	-1.504016	-0.951866
23	6	0	-0.849379	-1.840257	-0.253225
24	6	0	-1.360155	-0.643792	0.599765
25	7	0	-2.437577	-0.030882	-0.241334
26	6	0	-2.923737	-0.962452	-1.292059
27	6	0	-1.974527	-2.212870	-1.256160
28	6	0	-2.855783	1.200881	-0.120903
29	6	0	-2.256760	2.153357	0.874942
30	6	0	-3.380297	2.710067	1.794352
31	6	0	-4.640892	3.187170	1.059187
32	6	0	-5.136256	2.145883	0.051041
33	6	0	-3.992509	1.743960	-0.924935
34	6	0	-1.808627	-1.002769	2.011439
35	6	0	-0.811482	-1.450775	2.891554

36	6	0	-1.133548	-1.778490	4.206869
37	6	0	-2.450222	-1.661463	4.661152
38	6	0	-3.442656	-1.220784	3.787257
39	6	0	-3.126190	-0.890046	2.465832
40	6	0	-2.670448	-3.532485	-0.962509
41	6	0	-2.862763	-4.447424	-2.005076
42	6	0	-3.529999	-5.652089	-1.782841
43	6	0	-4.018119	-5.955798	-0.510171
44	6	0	-3.829908	-5.050276	0.535781
45	6	0	-3.159941	-3.846493	0.312705
46	6	0	-4.395801	-1.336181	-1.104639
47	8	0	-5.047610	-1.141878	-0.102770
48	8	0	-4.834003	-1.915609	-2.219499
49	6	0	-6.134982	-2.550267	-2.135072
50	8	0	4.307240	-0.335538	-3.651081
51	6	0	-1.329648	3.242610	0.112431
52	6	0	-0.674769	2.619001	-1.083241
53	7	0	0.352710	1.813028	-0.960529
54	8	0	0.869579	1.583005	0.242522
55	6	0	-1.980115	4.584081	-0.181769
56	6	0	-2.019277	5.542564	0.843221
57	6	0	-2.645423	6.774358	0.659089
58	6	0	-3.234536	7.083221	-0.568610
59	6	0	-3.170816	6.158242	-1.610922
60	6	0	-2.544945	4.924947	-1.420238
61	8	0	0.874371	1.195030	-1.965909
62	1	0	2.192113	0.907998	-0.050446
63	1	0	3.321155	-0.065365	0.789765
64	1	0	2.427621	-0.533354	-1.955631
65	1	0	4.101779	-2.229407	-2.052011
66	1	0	6.012665	-0.474764	-0.992797
67	1	0	3.812923	1.441329	-1.667590
68	1	0	0.358786	-0.720876	-1.614251
69	1	0	-0.618861	-2.679812	0.400156
70	1	0	-0.547115	0.082225	0.670291
71	1	0	-2.823854	-0.461965	-2.257631
72	1	0	-1.520788	-2.297571	-2.245734
73	1	0	-6.336176	-2.924358	-3.137455
74	1	0	-6.085789	-3.370794	-1.415450
75	1	0	-6.889681	-1.824413	-1.825862
76	1	0	3.871499	-4.263093	-0.827959
77	1	0	4.535560	-5.659009	1.097978
78	1	0	5.626314	-4.619713	3.078157
79	1	0	6.069540	-2.172256	3.094823
80	1	0	5.447961	-0.783817	1.176855
81	1	0	-2.493915	-4.212848	-3.000650
82	1	0	-3.665743	-6.352375	-2.602009
83	1	0	-4.536615	-6.893765	-0.333851
84	1	0	-4.201650	-5.278711	1.530446
85	1	0	-3.027219	-3.154043	1.137004
86	1	0	0.210247	-1.558669	2.537447
87	1	0	-0.353415	-2.125779	4.878135
88	1	0	-2.698883	-1.917356	5.686936
89	1	0	-4.471933	-1.138138	4.124633
90	1	0	-3.916597	-0.587145	1.790816
91	1	0	6.926562	1.508605	-0.918656
92	1	0	8.165713	3.038783	0.564891
93	1	0	6.983642	4.251291	2.386554
94	1	0	4.536554	3.931953	2.694214
95	1	0	3.283225	2.420036	1.210081
96	1	0	-1.558883	1.613965	1.511659
97	1	0	-3.644160	2.652960	-1.424500
98	1	0	-4.357040	1.061171	-1.691055
99	1	0	-2.959742	3.516904	2.401655
100	1	0	-3.647611	1.902792	2.487410
101	1	0	-5.962517	2.543366	-0.547298
102	1	0	-5.501735	1.243502	0.552285
103	1	0	-5.426888	3.388674	1.795877
104	1	0	-4.444648	4.129980	0.541544
105	1	0	-0.542296	3.421021	0.850811
106	1	0	-3.721361	8.042488	-0.717013
107	1	0	-3.599898	6.397543	-2.579709
108	1	0	-2.490289	4.241350	-2.262120
109	1	0	-1.547922	5.321774	1.797756
110	1	0	-2.664018	7.495968	1.470719
111	1	0	-1.018379	2.743516	-2.099245

X_LX_L–INT1a'·13a

Center Number	Atomic Number	Atomic Type	Coordinates (Angstroms) X	Y	Z
1	6	0	4.776855	0.148865	2.473399
2	6	0	3.622095	0.719869	1.929657
3	6	0	3.450759	2.111597	1.978330
4	6	0	4.428452	2.919967	2.553225
5	6	0	5.578924	2.346563	3.102557
6	6	0	5.747296	0.963059	3.063812
7	6	0	2.515213	-0.053741	1.245445
8	6	0	2.501448	0.053131	-0.301550
9	6	0	3.255095	-1.193498	-0.836485
10	6	0	2.893814	-2.288648	0.232055
11	7	0	2.541270	-1.548931	1.493665
12	7	0	1.134311	0.029078	-0.849613
13	6	0	0.090896	0.798274	-0.399348
14	8	0	-0.006668	1.216922	0.750254
15	6	0	4.734890	-1.018544	-1.167860
16	6	0	5.523081	0.043038	-0.708216
17	6	0	6.870821	0.135849	-1.064419
18	6	0	7.451784	-0.823405	-1.891530
19	6	0	6.668865	-1.871605	-2.381291
20	6	0	5.323548	-1.960453	-2.029749
21	6	0	4.000955	-3.277191	0.569911
22	8	0	4.649304	-3.213216	1.596365
23	6	0	-0.966575	1.163188	-1.454244
24	7	0	-2.307970	0.769260	-1.037527
25	6	0	-3.086400	1.936842	-0.586118
26	6	0	-2.029115	3.058352	-0.489741
27	6	0	-1.003469	2.716953	-1.573001
28	6	0	-2.969174	-0.291922	-1.706756
29	6	0	-4.308226	-0.367433	-1.824389
30	6	0	-5.032692	-1.482013	-2.537235
31	6	0	-4.098813	-2.314098	-3.423690
32	6	0	-2.820599	-2.654797	-2.654188
33	6	0	-2.061220	-1.379890	-2.259134
34	6	0	0.373467	3.352034	-1.474667
35	6	0	0.733169	4.240789	-0.455041
36	6	0	2.048393	4.700761	-0.346870
37	6	0	3.024928	4.274132	-1.247442
38	6	0	2.671286	3.407458	-2.285414
39	6	0	1.354525	2.960555	-2.399639
40	7	0	-2.652892	4.413117	-0.725836
41	8	0	-2.475293	5.264660	0.138862
42	6	0	-3.823658	1.721315	0.724804
43	6	0	-5.194947	1.974079	0.815139
44	6	0	-5.872786	1.802071	2.025904
45	6	0	-5.179451	1.380665	3.161020
46	6	0	-3.802935	1.141509	3.081780
47	6	0	-3.128794	1.309156	1.871929
48	8	0	-3.282332	4.571330	-1.768186
49	8	0	4.137347	-4.185094	-0.384512
50	6	0	5.256527	-5.099860	-0.239066
51	6	0	-3.022579	-2.996055	0.875643
52	6	0	-2.215133	-2.086570	1.468067
53	7	0	-0.808589	-2.248860	1.425925
54	8	0	-0.139518	-1.530730	2.206112
55	6	0	-4.465255	-2.944977	0.808264
56	6	0	-5.204763	-1.800120	1.177764
57	6	0	-6.587398	-1.797173	1.060524
58	6	0	-7.258263	-2.929393	0.580683
59	6	0	-6.539255	-4.069389	0.208908
60	6	0	-5.152568	-4.074465	0.314744
61	8	0	-0.272490	-3.054921	0.648347
62	1	0	1.580529	-1.809144	1.810652
63	1	0	3.216655	-1.813015	2.224378
64	1	0	-0.703664	0.732184	-2.422551
65	1	0	-1.463547	2.952021	-2.540546
66	1	0	-1.585797	3.104486	0.500118
67	1	0	-3.804568	2.224597	-1.366426
68	1	0	1.117989	-0.136760	-1.848372
69	1	0	2.981366	0.985815	-0.614505
70	1	0	1.560541	0.297823	1.629942

71	1	0	1.997831	-2.826958	-0.078857
72	1	0	2.773077	-1.518615	-1.764041
73	1	0	-0.008487	4.592524	0.253918
74	1	0	2.305745	5.397020	0.446545
75	1	0	4.047837	4.627142	-1.154612
76	1	0	3.415494	3.090769	-3.011126
77	1	0	1.084464	2.298195	-3.220215
78	1	0	5.204584	-5.603876	0.727563
79	1	0	5.153017	-5.808712	-1.058792
80	1	0	6.190191	-4.538729	-0.321010
81	1	0	2.553137	2.557704	1.557386
82	1	0	4.286661	3.996444	2.580996
83	1	0	6.337949	2.975601	3.558030
84	1	0	6.641074	0.509065	3.481159
85	1	0	4.969462	-0.918393	2.407323
86	1	0	4.720571	-2.770659	-2.429176
87	1	0	7.100247	-2.610299	-3.050874
88	1	0	8.499808	-0.748497	-2.166130
89	1	0	7.462125	0.966355	-0.690053
90	1	0	5.105236	0.811981	-0.072495
91	1	0	-5.739086	2.310529	-0.064482
92	1	0	-6.938448	2.006451	2.080983
93	1	0	-5.703016	1.249622	4.103612
94	1	0	-3.253603	0.830363	3.966588
95	1	0	-2.059837	1.128118	1.808568
96	1	0	-4.940148	0.391123	-1.378491
97	1	0	-5.841870	-1.053816	-3.143505
98	1	0	-5.527354	-2.134056	-1.801719
99	1	0	-2.161207	-3.302469	-3.243790
100	1	0	-3.092115	-3.215286	-1.752965
101	1	0	-4.602525	-3.228048	-3.758886
102	1	0	-3.838700	-1.741817	-4.324617
103	1	0	-2.536072	-3.852461	0.414784
104	1	0	-1.534956	-1.007079	-3.151303
105	1	0	-1.283965	-1.621273	-1.520414
106	1	0	-2.521298	-1.219452	2.031931
107	1	0	-4.588408	-4.954813	0.017353
108	1	0	-7.058769	-4.944946	-0.167835
109	1	0	-8.340646	-2.919531	0.490431
110	1	0	-7.141496	-0.905765	1.336695
111	1	0	-4.699828	-0.907678	1.525693

(*Si*,*Re*)–**X_LX_L–TS2b**

Center Number	Atomic Number	Atomic Type	Coordinates (Angstroms) X	Y	Z
1	6	0	4.947920	-4.115537	0.366785
2	6	0	4.558231	-2.937231	-0.295534
3	6	0	5.507044	-2.260493	-1.083849
4	6	0	6.802858	-2.750699	-1.213701
5	6	0	7.177263	-3.923851	-0.552744
6	6	0	6.245902	-4.603685	0.236806
7	6	0	3.205113	-2.366106	-0.190134
8	6	0	3.570380	-0.899443	1.356017
9	6	0	2.335593	-0.244143	1.548262
10	6	0	1.455720	-0.918610	2.564391
11	6	0	2.128165	-0.856911	3.965993
12	6	0	3.659730	-1.061595	3.864814
13	6	0	4.035881	-1.747026	2.536140
14	7	0	2.002011	0.940184	0.994990
15	6	0	0.755230	1.654373	1.301875
16	6	0	1.160543	3.144604	1.193927
17	6	0	2.157914	3.102928	0.032499
18	6	0	2.961165	1.799625	0.249603
19	6	0	-0.349382	1.266851	0.302059
20	8	0	-0.230712	1.492312	-0.899233
21	6	0	3.516263	1.264432	-1.055247
22	6	0	4.853727	1.538438	-1.365599
23	6	0	5.381429	1.170015	-2.605026
24	6	0	4.577070	0.511578	-3.537065
25	6	0	3.243454	0.230243	-3.226446
26	6	0	2.708177	0.612606	-1.995915
27	7	0	3.066650	4.307602	0.067634
28	8	0	3.815234	4.417614	1.038058

29	6	0	0.013006	4.130316	1.131358
30	6	0	-0.650008	4.453650	-0.060790
31	6	0	-1.725856	5.341855	-0.049230
32	6	0	-2.156902	5.914636	1.148764
33	6	0	-1.501386	5.600230	2.340313
34	6	0	-0.422336	4.716642	2.327877
35	7	0	-1.436102	0.652516	0.834219
36	6	0	-2.599927	0.264722	0.030095
37	6	0	-3.603789	-0.472575	0.951239
38	6	0	-3.109214	-1.962049	0.864881
39	7	0	-2.400239	-2.100921	-0.444768
40	6	0	-2.186820	-0.746124	-1.084828
41	6	0	-5.087078	-0.245816	0.675372
42	6	0	-5.593014	0.245855	-0.532613
43	6	0	-6.967899	0.417410	-0.711273
44	6	0	-7.858028	0.109273	0.315692
45	6	0	-7.363311	-0.356133	1.536440
46	6	0	-5.991596	-0.523278	1.714843
47	6	0	-2.892908	-0.588074	-2.417014
48	6	0	-2.422510	0.433226	-3.255983
49	6	0	-3.021855	0.658551	-4.493949
50	6	0	-4.087720	-0.140903	-4.915062
51	6	0	-4.557153	-1.158713	-4.085434
52	6	0	-3.968187	-1.380011	-2.838202
53	6	0	-4.215126	-3.008502	0.916487
54	8	0	-4.630295	-3.206100	2.160399
55	6	0	-5.781037	-4.079658	2.311197
56	8	0	-4.640376	-3.560419	-0.077996
57	8	0	2.974902	5.095260	-0.862716
58	1	0	-2.959728	-2.722534	-1.042872
59	1	0	0.438929	1.448834	2.326350
60	1	0	1.753736	3.356022	2.091795
61	1	0	1.679569	3.115956	-0.941858
62	1	0	3.791731	2.022053	0.929621
63	1	0	-1.519411	0.608618	1.839827
64	1	0	-3.030071	1.161637	-0.421281
65	1	0	-1.115445	-0.670690	-1.246487
66	1	0	-2.376001	-2.163366	1.648823
67	1	0	-3.440993	-0.152683	1.986272
68	1	0	-0.335949	4.010557	-0.998605
69	1	0	-2.223206	5.591613	-0.982121
70	1	0	-2.991372	6.609929	1.152140
71	1	0	-1.819907	6.050883	3.275886
72	1	0	0.096916	4.489559	3.256842
73	1	0	-5.575331	-5.050266	1.856347
74	1	0	-5.929893	-4.172420	3.385694
75	1	0	-6.647356	-3.617321	1.832457
76	1	0	-1.588890	1.046488	-2.925756
77	1	0	-2.649091	1.452469	-5.134667
78	1	0	-4.548759	0.029388	-5.883487
79	1	0	-5.390111	-1.780688	-4.399482
80	1	0	-4.396662	-2.145706	-2.196125
81	1	0	-5.616714	-0.873338	2.672447
82	1	0	-8.043157	-0.572912	2.355529
83	1	0	-8.926226	0.244046	0.173959
84	1	0	-7.336461	0.796182	-1.660030
85	1	0	-4.930886	0.502073	-1.348904
86	1	0	5.482347	2.052386	-0.641855
87	1	0	6.418328	1.394380	-2.837532
88	1	0	4.986209	0.218613	-4.499568
89	1	0	2.614229	-0.283721	-3.947629
90	1	0	1.671669	0.403862	-1.759827
91	1	0	4.341926	-0.373889	0.803758
92	1	0	5.115010	-1.903201	2.464081
93	1	0	3.579715	-2.746306	2.507825
94	1	0	1.902769	0.103236	4.443989
95	1	0	1.672062	-1.634571	4.588317
96	1	0	4.021901	-1.650129	4.714405
97	1	0	4.169726	-0.091986	3.912383
98	1	0	2.986310	-1.565289	-0.885733
99	1	0	0.434097	-0.541674	2.588825
100	1	0	1.381784	-1.972362	2.271649
101	6	0	2.098505	-3.177645	0.148605
102	1	0	2.142598	-4.199985	0.490483
103	1	0	4.240656	-4.654473	0.989645
104	1	0	6.531845	-5.516287	0.751544
105	1	0	8.188486	-4.307437	-0.652485

106	1	0	7.520605	-2.218904	-1.831653
107	1	0	5.218754	-1.348387	-1.596620
108	7	0	0.844344	-2.682811	0.010687
109	8	0	-0.170203	-3.439011	0.239923
110	8	0	0.665948	-1.468251	-0.305187
111	1	0	-1.462351	-2.588958	-0.261793

(2S,1’R)–**X_LX_L–INT3a**

Center Number	Atomic Number	Atomic Type	Coordinates (Angstroms) X	Y	Z
1	6	0	3.778647	-1.532258	2.784003
2	6	0	2.728130	-0.705603	2.364341
3	6	0	2.189128	0.225844	3.264132
4	6	0	2.693073	0.326089	4.559942
5	6	0	3.732829	-0.508382	4.977350
6	6	0	4.273283	-1.435381	4.086600
7	6	0	2.124205	-0.747668	0.973072
8	6	0	2.634127	0.329444	-0.041487
9	6	0	3.647587	-0.375084	-0.976057
10	6	0	3.108823	-1.850426	-1.010000
11	7	0	2.357781	-2.060543	0.262885
12	7	0	1.522191	0.832404	-0.854130
13	6	0	0.455925	1.453482	-0.296336
14	8	0	0.386791	1.682420	0.909371
15	6	0	5.124011	-0.219617	-0.627172
16	6	0	5.593348	0.139324	0.640681
17	6	0	6.964031	0.245841	0.888387
18	6	0	7.886365	0.003612	-0.127908
19	6	0	7.429316	-0.330437	-1.405053
20	6	0	6.061763	-0.433152	-1.651791
21	6	0	4.191543	-2.918141	-1.101021
22	8	0	4.575385	-3.543348	-0.133517
23	6	0	-0.711115	1.790529	-1.240935
24	7	0	-1.864257	0.932176	-0.897863
25	6	0	-2.835252	1.659076	-0.018824
26	6	0	-2.159851	3.033866	0.183227
27	6	0	-1.265918	3.218321	-1.048453
28	6	0	-1.988579	-0.335982	-1.331051
29	6	0	-3.326684	-1.057755	-1.145798
30	6	0	-3.886247	-1.469426	-2.531072
31	6	0	-3.615660	-0.375204	-3.571721
32	6	0	-2.095614	-0.213840	-3.821891
33	6	0	-1.278457	-0.728193	-2.611840
34	6	0	-0.240396	4.331882	-1.036288
35	6	0	0.482667	4.689632	0.110139
36	6	0	1.432757	5.709545	0.052390
37	6	0	1.677261	6.381086	-1.146857
38	6	0	0.961193	6.032138	-2.293022
39	6	0	0.007061	5.016726	-2.234044
40	7	0	-3.194292	4.132599	0.251831
41	8	0	-3.104334	4.915869	1.185450
42	6	0	-3.228715	1.012614	1.292470
43	6	0	-4.558673	1.138467	1.712405
44	6	0	-4.960532	0.630635	2.949335
45	6	0	-4.036962	-0.021322	3.768437
46	6	0	-2.709104	-0.149032	3.351640
47	6	0	-2.301330	0.372662	2.124086
48	8	0	-4.033560	4.171816	-0.647754
49	8	0	4.640948	-3.041114	-2.342865
50	6	0	5.775969	-3.930263	-2.518217
51	6	0	-3.242592	-2.256915	-0.114943
52	6	0	-2.079664	-3.155330	-0.385505
53	7	0	-0.885809	-2.631129	-0.361710
54	8	0	-0.783525	-1.317325	-0.144519
55	6	0	-4.569441	-2.986579	-0.012721
56	6	0	-4.908130	-4.060061	-0.847730
57	6	0	-6.153750	-4.680140	-0.739899
58	6	0	-7.081213	-4.231876	0.202060
59	6	0	-6.753955	-3.162086	1.037154
60	6	0	-5.506713	-2.546555	0.931051
61	8	0	0.210614	-3.285291	-0.589019
62	1	0	2.894366	-2.719221	0.841060
63	1	0	-0.425873	1.651925	-2.284573

64	1	0	-1.948153	3.383673	-1.891413
65	1	0	-1.613090	3.078120	1.119309
66	1	0	-3.735209	1.831480	-0.617864
67	1	0	1.550212	0.698254	-1.854364
68	1	0	3.075468	1.169998	0.496789
69	1	0	1.044308	-0.653130	1.060184
70	1	0	2.394901	-1.979304	-1.826784
71	1	0	3.535173	0.024055	-1.989853
72	1	0	0.313212	4.172235	1.047119
73	1	0	1.978465	5.983421	0.950783
74	1	0	2.414171	7.178088	-1.185552
75	1	0	1.134687	6.556815	-3.228104
76	1	0	-0.561874	4.761842	-3.125958
77	1	0	5.533074	-4.924983	-2.140230
78	1	0	5.958923	-3.951260	-3.591266
79	1	0	6.636047	-3.524782	-1.980325
80	1	0	1.382393	0.874171	2.936491
81	1	0	2.266971	1.051842	5.246612
82	1	0	4.119441	-0.435222	5.989561
83	1	0	5.087261	-2.083573	4.397358
84	1	0	4.258365	-2.231592	2.103285
85	1	0	5.716444	-0.682944	-2.651118
86	1	0	8.136116	-0.494804	-2.213439
87	1	0	8.951314	0.088020	0.067121
88	1	0	7.304276	0.522295	1.882009
89	1	0	4.904904	0.341138	1.450783
90	1	0	-5.282454	1.640230	1.073948
91	1	0	-5.993484	0.739524	3.266620
92	1	0	-4.348952	-0.425555	4.726967
93	1	0	-1.985431	-0.652738	3.986362
94	1	0	-1.272595	0.276672	1.804142
95	1	0	-4.040854	-0.360888	-0.703974
96	1	0	-4.957769	-1.654762	-2.425797
97	1	0	-3.440388	-2.417444	-2.856627
98	1	0	-1.855320	0.836111	-4.026580
99	1	0	-1.786008	-0.785026	-4.703607
100	1	0	-4.133800	-0.599382	-4.508944
101	1	0	-4.036318	0.570825	-3.205133
102	1	0	-3.045260	-1.768110	0.845760
103	1	0	-0.245654	-0.382653	-2.633124
104	1	0	-1.228356	-1.818160	-2.645353
105	1	0	-2.132659	-4.217164	-0.577508
106	1	0	-4.202948	-4.417467	-1.593597
107	1	0	-6.398192	-5.513480	-1.392483
108	1	0	-8.050024	-4.715524	0.286739
109	1	0	-7.466730	-2.810350	1.777837
110	1	0	-5.253808	-1.720354	1.590032
111	1	0	1.411875	-2.538289	0.010847

(*Si*,*Si*)–**X_LX_L–TS2a**

Center Number	Atomic Number	Atomic Type	Coordinates (Angstroms) X	Y	Z
1	6	0	-5.480046	0.190247	-0.572576
2	6	0	-4.600893	1.006461	-1.293359
3	6	0	-5.113101	1.749121	-2.370861
4	6	0	-6.468449	1.709091	-2.691970
5	6	0	-7.339841	0.909510	-1.948313
6	6	0	-6.838334	0.145720	-0.895976
7	6	0	-3.108281	1.123670	-1.000413
8	6	0	-2.454526	-0.044176	-0.217390
9	6	0	-2.414656	0.382560	1.279252
10	7	0	-2.298410	1.888422	1.196845
11	6	0	-2.665671	2.384960	-0.172810
12	7	0	-1.123410	-0.293285	-0.768548
13	6	0	-0.452378	-1.423471	-0.400752
14	8	0	-0.868997	-2.206719	0.446012
15	6	0	-3.546796	-0.118320	2.151423
16	6	0	-3.500596	-1.474915	2.508413
17	6	0	-4.511394	-2.029821	3.290515
18	6	0	-5.572511	-1.235907	3.734768
19	6	0	-5.620116	0.112262	3.383036
20	6	0	-4.615122	0.671892	2.589711
21	6	0	-3.707965	3.482092	-0.024464

22	8	0	-4.315212	3.689767	1.007583
23	6	0	0.823239	-1.705711	-1.233583
24	7	0	2.002451	-0.928060	-0.790225
25	6	0	2.762195	-1.726965	0.237895
26	6	0	2.034292	-3.095384	0.242750
27	6	0	1.319399	-3.162937	-1.108045
28	6	0	2.618656	-0.023357	-1.584863
29	6	0	3.846444	0.574858	-1.222053
30	6	0	4.839060	0.960250	-2.317533
31	6	0	4.246603	0.957490	-3.736944
32	6	0	2.779392	1.385345	-3.700575
33	6	0	1.969719	0.356030	-2.903213
34	6	0	0.278634	-4.238647	-1.342797
35	6	0	-0.081679	-5.178995	-0.373143
36	6	0	-1.043487	-6.149428	-0.657933
37	6	0	-1.660402	-6.188007	-1.908718
38	6	0	-1.301142	-5.256117	-2.884518
39	6	0	-0.333409	-4.293324	-2.602514
40	7	0	3.045812	-4.211644	0.391238
41	8	0	2.857638	-5.015728	1.293926
42	6	0	2.883807	-1.101143	1.608195
43	6	0	4.154879	-0.900394	2.159262
44	6	0	4.294493	-0.342089	3.431553
45	6	0	3.160866	0.024360	4.160050
46	6	0	1.889761	-0.191751	3.621699
47	6	0	1.751809	-0.761054	2.357752
48	8	0	3.975677	-4.234193	-0.412251
49	8	0	-3.837662	4.164846	-1.154265
50	6	0	-4.907114	5.145144	-1.186706
51	6	0	3.230406	2.387733	-0.284857
52	6	0	4.543903	2.981669	0.030646
53	6	0	5.028863	4.032626	-0.767665
54	6	0	6.252750	4.636927	-0.481912
55	6	0	7.016660	4.187909	0.596277
56	6	0	6.549556	3.134658	1.390241
57	6	0	5.324760	2.536348	1.112797
58	1	0	-1.261402	2.083866	1.370518
59	1	0	-2.874736	2.369744	1.897690
60	1	0	0.583816	-1.469638	-2.266542
61	1	0	2.108738	-3.267859	-1.865621
62	1	0	1.349359	-3.198099	1.079693
63	1	0	3.759155	-1.893289	-0.177684
64	1	0	-0.552237	0.526263	-0.998729
65	1	0	-3.016079	-0.972860	-0.317247
66	1	0	-1.470790	0.044220	1.706621
67	1	0	-1.754438	2.801153	-0.607930
68	1	0	-2.606725	1.195969	-1.969194
69	1	0	0.389528	-5.173048	0.603481
70	1	0	-1.308190	-6.877863	0.103038
71	1	0	-2.408739	-6.944615	-2.125993
72	1	0	-1.765866	-5.284243	-3.866008
73	1	0	-0.044817	-3.583531	-3.376108
74	1	0	-4.803214	5.842406	-0.353424
75	1	0	-4.797641	5.655292	-2.142345
76	1	0	-5.868231	4.629200	-1.127562
77	1	0	-2.675866	-2.089356	2.157210
78	1	0	-4.466543	-3.081347	3.558688
79	1	0	-6.356990	-1.667542	4.349365
80	1	0	-6.445137	0.735618	3.714901
81	1	0	-4.712560	1.711046	2.290052
82	1	0	-4.442118	2.367106	-2.960577
83	1	0	-6.840265	2.289404	-3.531775
84	1	0	-8.396224	0.872576	-2.197771
85	1	0	-7.500453	-0.493327	-0.319089
86	1	0	-5.124548	-0.422987	0.244813
87	1	0	5.040491	-1.185447	1.595439
88	1	0	5.285642	-0.197254	3.851797
89	1	0	3.266921	0.465093	5.146904
90	1	0	1.003979	0.090424	4.181699
91	1	0	0.760903	-0.936281	1.954416
92	1	0	4.313306	0.205695	-0.316491
93	1	0	5.680984	0.258947	-2.263161
94	1	0	5.267412	1.942414	-2.094422
95	1	0	2.359828	1.456185	-4.709466
96	1	0	2.680916	2.380071	-3.250106
97	1	0	4.832984	1.614782	-4.386739
98	1	0	4.308210	-0.049720	-4.170708

99	1	0	2.736918	2.790834	-1.162614
100	1	0	1.882074	-0.554914	-3.513683
101	1	0	0.955454	0.722317	-2.723458
102	6	0	2.355611	2.098167	0.791378
103	1	0	2.667828	1.997676	1.819280
104	1	0	4.433847	4.385267	-1.606488
105	1	0	6.608863	5.454555	-1.101481
106	1	0	7.972669	4.653160	0.817493
107	1	0	7.144429	2.780986	2.227414
108	1	0	4.974661	1.714665	1.729912
109	7	0	1.031563	1.993287	0.600104
110	8	0	0.279977	1.846388	1.635541
111	8	0	0.510050	2.057400	-0.572904

(*Si*,*Si*)–**X_LX_L–TS2a**

Center Number	Atomic Number	Atomic Type	Coordinates (Angstroms) X	Y	Z
1	6	0	5.431825	2.382244	0.910471
2	6	0	4.586339	2.938759	-0.059073
3	6	0	5.002221	4.092102	-0.734487
4	6	0	6.238514	4.676555	-0.453178
5	6	0	7.075506	4.111884	0.510025
6	6	0	6.667218	2.964002	1.193376
7	6	0	3.245308	2.306399	-0.391325
8	6	0	2.374025	2.199781	0.815721
9	7	0	1.089081	2.014300	0.670680
10	8	0	0.578565	1.866347	-0.536845
11	8	0	0.296857	1.948524	1.704545
12	6	0	3.484341	0.904440	-1.112993
13	6	0	2.237561	0.157949	-1.494767
14	6	0	1.603740	0.491671	-2.830193
15	6	0	2.060901	1.833963	-3.407634
16	6	0	3.601898	1.952082	-3.472763
17	6	0	4.307851	1.075607	-2.416424
18	7	0	1.878111	-0.940129	-0.865433
19	6	0	0.785131	-1.838784	-1.325250
20	6	0	1.410654	-3.235128	-1.148600
21	6	0	2.054768	-3.069793	0.226401
22	6	0	2.689090	-1.658120	0.201618
23	6	0	-0.518652	-1.599037	-0.509902
24	8	0	-1.010748	-2.472921	0.191502
25	6	0	2.762174	-1.014591	1.562209
26	6	0	4.017711	-0.813073	2.148597
27	6	0	4.115302	-0.275240	3.432249
28	6	0	2.957302	0.067459	4.133853
29	6	0	1.703419	-0.151970	3.559330
30	6	0	1.606345	-0.704591	2.284961
31	7	0	3.134033	-4.103002	0.467792
32	8	0	4.113293	-4.067092	-0.274505
33	6	0	0.494986	-4.414293	-1.409005
34	6	0	0.138220	-5.338140	-0.422913
35	6	0	-0.695628	-6.412031	-0.736816
36	6	0	-1.186960	-6.570324	-2.033075
37	6	0	-0.829919	-5.654033	-3.024221
38	6	0	0.011976	-4.587762	-2.712713
39	7	0	-1.088541	-0.381090	-0.734728
40	6	0	-2.415427	-0.104344	-0.186321
41	6	0	-3.047225	1.071546	-0.977323
42	6	0	-2.577346	2.329916	-0.162280
43	7	0	-2.216236	1.840843	1.207862
44	6	0	-2.350217	0.342054	1.303714
45	6	0	-4.543100	0.982862	-1.260721
46	6	0	-5.437417	0.206527	-0.514968
47	6	0	-6.798646	0.188722	-0.828323
48	6	0	-7.288340	0.940623	-1.894761
49	6	0	-6.402678	1.701618	-2.661695
50	6	0	-5.044457	1.714797	-2.350454
51	6	0	-3.474371	-0.146934	2.193459
52	6	0	-3.448177	-1.508325	2.533219
53	6	0	-4.451506	-2.053161	3.331525
54	6	0	-5.486407	-1.243426	3.808312
55	6	0	-5.515806	0.109077	3.471619
56	6	0	-4.517286	0.658590	2.662895

57	6	0	-3.603676	3.442864	-0.025774
58	8	0	-3.715376	4.124265	-1.159785
59	6	0	-4.772167	5.116941	-1.202246
60	8	0	-4.219677	3.661248	0.998622
61	8	0	2.945363	-4.901497	1.373483
62	1	0	-1.152751	2.041825	1.387798
63	1	0	-2.788182	2.332746	1.903613
64	1	0	0.558532	-1.648837	-2.371929
65	1	0	2.239678	-3.279332	-1.869180
66	1	0	1.339173	-3.185120	1.036699
67	1	0	3.692801	-1.768098	-0.215121
68	1	0	-0.454917	0.433414	-0.802074
69	1	0	-2.996413	-1.021174	-0.276515
70	1	0	-1.403046	-0.001201	1.722822
71	1	0	-1.659920	2.724546	-0.605972
72	1	0	-2.546397	1.122366	-1.947584
73	1	0	0.511922	-5.237949	0.589974
74	1	0	-0.959879	-7.126516	0.037268
75	1	0	-1.836332	-7.407254	-2.272599
76	1	0	-1.196965	-5.774431	-4.039409
77	1	0	0.303687	-3.889975	-3.496210
78	1	0	-4.663815	5.817519	-0.372240
79	1	0	-4.652490	5.621043	-2.159969
80	1	0	-5.740188	4.613761	-1.144596
81	1	0	-2.647868	-2.137973	2.151392
82	1	0	-4.422624	-3.108777	3.585400
83	1	0	-6.265652	-1.666766	4.435194
84	1	0	-6.321780	0.743992	3.827492
85	1	0	-4.601258	1.702214	2.374313
86	1	0	-4.362535	2.304502	-2.956529
87	1	0	-6.766182	2.273059	-3.511200
88	1	0	-8.347093	0.924730	-2.136322
89	1	0	-7.472665	-0.419631	-0.232300
90	1	0	-5.091228	-0.395436	0.314803
91	1	0	4.920031	-1.081379	1.603486
92	1	0	5.092377	-0.124043	3.881425
93	1	0	3.031619	0.495575	5.128964
94	1	0	0.800352	0.117162	4.096690
95	1	0	0.629409	-0.886065	1.850283
96	1	0	4.063987	0.310308	-0.406286
97	1	0	4.508792	0.077442	-2.826235
98	1	0	5.279184	1.499578	-2.148413
99	1	0	1.624778	1.946329	-4.405063
100	1	0	1.628492	2.623468	-2.788400
101	1	0	3.889293	2.999341	-3.338170
102	1	0	3.962990	1.661181	-4.464596
103	1	0	2.728493	2.945517	-1.109161
104	1	0	1.901794	-0.322244	-3.512495
105	1	0	0.515045	0.463388	-2.762392
106	1	0	2.732492	2.289071	1.830415
107	1	0	4.351007	4.543868	-1.479114
108	1	0	6.544330	5.573305	-0.984324
109	1	0	8.037653	4.564868	0.730349
110	1	0	7.310338	2.523247	1.950008
111	1	0	5.117099	1.497790	1.456542

X_LX_L–INT1b·13a

Center Number	Atomic Number	Atomic Type	Coordinates (Angstroms) X	Y	Z
1	6	0	4.061335	-2.449263	-0.007680
2	6	0	3.293462	-1.863057	-1.025138
3	6	0	2.745890	-2.679890	-2.020569
4	6	0	2.954095	-4.058358	-2.002783
5	6	0	3.712456	-4.634781	-0.985131
6	6	0	4.266095	-3.827456	0.011024
7	6	0	3.058896	-0.370698	-1.095514
8	6	0	2.112998	0.256231	-0.026384
9	7	0	2.555551	1.699767	0.075171
10	6	0	3.771021	1.937249	-0.821171
11	6	0	4.313571	0.511559	-0.988362
12	6	0	2.255197	-0.345555	1.365443
13	8	0	3.239375	-0.025596	2.038428
14	6	0	4.708211	2.957043	-0.235475

15	6	0	5.292719	2.745255	1.023883
16	6	0	6.168389	3.692352	1.550734
17	6	0	6.474509	4.845869	0.822702
18	6	0	5.900035	5.054664	-0.431858
19	6	0	5.013861	4.114125	-0.959069
20	7	0	5.134695	0.410817	-2.251638
21	8	0	6.242038	-0.092196	-2.152507
22	7	0	1.330489	-1.241711	1.778835
23	6	0	-0.091170	-1.331805	1.423697
24	6	0	-0.862359	-0.002719	1.663619
25	7	0	-2.081073	-0.212458	0.884887
26	6	0	-2.017387	-1.389064	0.038611
27	6	0	-0.505886	-1.782078	0.014229
28	6	0	-3.226892	0.584605	0.919126
29	6	0	-4.374233	0.222969	0.290693
30	6	0	-5.653484	1.016986	0.352518
31	6	0	-5.612983	2.126162	1.411055
32	6	0	-4.271156	2.858536	1.344918
33	6	0	-3.116277	1.900892	1.664761
34	6	0	-1.021869	0.255933	3.149718
35	6	0	-0.239410	1.236578	3.765342
36	6	0	-0.323785	1.450649	5.142513
37	6	0	-1.201929	0.685954	5.911337
38	6	0	-1.987255	-0.296145	5.299396
39	6	0	-1.896757	-0.516650	3.925775
40	6	0	-0.258287	-3.227254	-0.372229
41	6	0	-0.604133	-3.632364	-1.670571
42	6	0	-0.465866	-4.959726	-2.067924
43	6	0	0.035892	-5.907392	-1.171445
44	6	0	0.399607	-5.512183	0.115085
45	6	0	0.249701	-4.181533	0.513201
46	6	0	-2.839929	-2.561379	0.591552
47	8	0	-2.825609	-2.914688	1.751985
48	8	0	-3.514359	-3.181755	-0.385131
49	6	0	-4.175807	-4.408812	-0.003001
50	8	0	4.609742	0.825210	-3.284277
51	6	0	-2.843749	1.873787	-2.193086
52	6	0	-1.965266	2.620554	-1.472952
53	7	0	-0.674743	2.136198	-1.207174
54	8	0	0.010460	2.780305	-0.362314
55	6	0	-4.178994	2.253089	-2.590449
56	6	0	-4.981038	1.288588	-3.236906
57	6	0	-6.280318	1.590867	-3.628586
58	6	0	-6.800834	2.865564	-3.386989
59	6	0	-6.016395	3.836821	-2.752959
60	6	0	-4.718522	3.537741	-2.359286
61	8	0	-0.229216	1.128083	-1.788350
62	1	0	2.833784	1.880542	1.048060
63	1	0	1.097722	0.268329	-0.397583
64	1	0	2.603863	-0.133673	-2.064098
65	1	0	4.973462	0.218129	-0.175024
66	1	0	3.381150	2.262660	-1.787943
67	1	0	1.551777	-1.590887	2.705942
68	1	0	-0.507628	-2.060543	2.121160
69	1	0	-0.282077	0.834459	1.239143
70	1	0	-2.370447	-1.158332	-0.972310
71	1	0	-0.053260	-1.140756	-0.745901
72	1	0	-4.678628	-4.760457	-0.903163
73	1	0	-3.432983	-5.135248	0.335986
74	1	0	-4.893842	-4.218756	0.797992
75	1	0	2.152203	-2.235763	-2.815728
76	1	0	2.511605	-4.679045	-2.774346
77	1	0	3.873740	-5.708547	-0.968327
78	1	0	4.864662	-4.269729	0.802084
79	1	0	4.496710	-1.836409	0.775428
80	1	0	-0.999326	-2.899699	-2.370668
81	1	0	-0.748342	-5.255076	-3.074610
82	1	0	0.147041	-6.943839	-1.477019
83	1	0	0.800790	-6.238433	0.816013
84	1	0	0.541478	-3.899509	1.518917
85	1	0	0.431848	1.846349	3.163067
86	1	0	0.287761	2.216872	5.610450
87	1	0	-1.276592	0.854605	6.981759
88	1	0	-2.672316	-0.892917	5.895046
89	1	0	-2.501899	-1.280161	3.445790
90	1	0	4.568499	4.275226	-1.937614
91	1	0	6.139284	5.947969	-1.000668

92	1	0	7.163077	5.578575	1.232881
93	1	0	6.618808	3.526118	2.524605
94	1	0	5.067681	1.843359	1.590707
95	1	0	-4.432655	-0.720183	-0.244090
96	1	0	-3.095525	1.706900	2.742622
97	1	0	-2.154996	2.375802	1.428749
98	1	0	-6.488950	0.332953	0.554752
99	1	0	-5.870350	1.456761	-0.633153
100	1	0	-4.244389	3.701601	2.044390
101	1	0	-4.145039	3.274665	0.337678
102	1	0	-6.445402	2.823055	1.261902
103	1	0	-5.734366	1.688380	2.410805
104	1	0	-2.490380	0.902759	-2.528377
105	1	0	1.740277	2.303894	-0.168823
106	1	0	-4.577521	0.295078	-3.413676
107	1	0	-6.887318	0.837793	-4.121399
108	1	0	-4.116058	4.305784	-1.884969
109	1	0	-6.419932	4.828368	-2.572608
110	1	0	-7.814828	3.104583	-3.693480
111	1	0	-2.161867	3.568410	-0.996080

Si,Re)–**X_LX_L–TS2b**

Center Number	Atomic Number	Atomic Type	Coordinates (Angstroms) X	Y	Z
1	6	0	4.955918	-1.612591	-3.092185
2	6	0	4.236041	-2.399126	-2.174879
3	6	0	4.631687	-3.733381	-1.979624
4	6	0	5.708341	-4.264077	-2.689057
5	6	0	6.411020	-3.471589	-3.599664
6	6	0	6.030764	-2.141606	-3.800555
7	6	0	3.107694	-1.777099	-1.446239
8	6	0	4.037552	-0.642700	-0.051608
9	6	0	2.999900	-0.587626	0.939671
10	6	0	2.898139	-1.706402	1.945824
11	6	0	4.059069	-2.705849	1.875378
12	6	0	5.376053	-1.945697	1.723611
13	6	0	5.396571	-1.207477	0.375441
14	7	0	2.042872	0.335101	0.916990
15	6	0	0.794330	0.287675	1.718289
16	6	0	0.052911	1.588546	1.298870
17	6	0	0.507099	1.873744	-0.139323
18	6	0	1.995733	1.412893	-0.093803
19	7	0	-1.379087	1.523149	1.603247
20	6	0	-2.256427	0.526463	1.287176
21	8	0	-3.203048	0.221117	2.011856
22	6	0	0.937997	0.220062	3.223570
23	6	0	0.087928	-0.623030	3.946283
24	6	0	0.130050	-0.638580	5.341654
25	6	0	1.033308	0.180823	6.020252
26	6	0	1.885871	1.023355	5.300167
27	6	0	1.835033	1.053256	3.906901
28	6	0	2.905587	2.568410	0.316442
29	8	0	3.546909	3.060744	-0.744615
30	6	0	4.282239	4.288665	-0.519639
31	6	0	0.337703	3.275372	-0.690883
32	6	0	0.504472	3.452166	-2.072941
33	6	0	0.419505	4.717439	-2.648380
34	6	0	0.155842	5.830988	-1.845349
35	6	0	-0.019831	5.664735	-0.471576
36	6	0	0.075937	4.394769	0.103779
37	6	0	-2.101503	-0.177129	-0.052508
38	7	0	-2.430579	-1.633234	0.145354
39	6	0	-3.571431	-2.025999	-0.775337
40	6	0	-4.268601	-0.675094	-0.997579
41	6	0	-3.114489	0.330808	-1.129976
42	6	0	-4.403582	-3.134324	-0.189408
43	6	0	-5.057491	-2.958501	1.040423
44	6	0	-5.818564	-3.993827	1.579312
45	6	0	-5.939253	-5.205019	0.891856
46	6	0	-5.295254	-5.380415	-0.333611
47	6	0	-4.525064	-4.348444	-0.871969
48	6	0	-3.471403	1.799702	-1.060444
49	6	0	-2.847764	2.685714	-1.945981

50	6	0	-3.104465	4.055011	-1.883832
51	6	0	-4.001192	4.552451	-0.938754
52	6	0	-4.638805	3.674955	-0.058757
53	6	0	-4.375130	2.307268	-0.115440
54	7	0	-5.094152	-0.709389	-2.260201
55	8	0	-4.522488	-1.083613	-3.282752
56	8	0	-6.257792	-0.349311	-2.171066
57	8	0	2.979391	3.003546	1.446448
58	6	0	2.056775	-2.596889	-0.888785
59	7	0	0.822868	-2.103247	-0.755366
60	8	0	0.454441	-1.018335	-1.327779
61	8	0	-0.023436	-2.740685	0.005909
62	1	0	-2.736297	-1.771536	1.115150
63	1	0	-1.092666	-0.164850	-0.449489
64	1	0	-2.645966	0.128574	-2.099603
65	1	0	-4.960587	-0.431557	-0.194446
66	1	0	-3.116745	-2.330585	-1.720781
67	1	0	-1.631728	1.951885	2.487951
68	1	0	0.444399	2.386402	1.932473
69	1	0	0.235124	-0.589027	1.365183
70	1	0	2.279402	1.032854	-1.071205
71	1	0	0.026823	1.181943	-0.827136
72	1	0	4.751214	4.522477	-1.474190
73	1	0	3.587090	5.077538	-0.222582
74	1	0	5.031036	4.142741	0.261922
75	1	0	-2.149783	2.304260	-2.686586
76	1	0	-2.595650	4.727688	-2.566223
77	1	0	-4.207313	5.617803	-0.890192
78	1	0	-5.345553	4.054099	0.673759
79	1	0	-4.863321	1.643793	0.590825
80	1	0	0.705623	2.586478	-2.701016
81	1	0	0.553917	4.834536	-3.720047
82	1	0	0.083880	6.819587	-2.289548
83	1	0	-0.232982	6.523453	0.158438
84	1	0	-0.064484	4.292978	1.174872
85	1	0	-0.610256	-1.269006	3.418597
86	1	0	-0.537651	-1.292690	5.894852
87	1	0	1.072692	0.165231	7.105471
88	1	0	2.588650	1.664035	5.825209
89	1	0	2.484738	1.718409	3.345472
90	1	0	-4.021738	-4.485416	-1.825588
91	1	0	-5.388803	-6.319481	-0.870594
92	1	0	-6.536625	-6.009117	1.311310
93	1	0	-6.322751	-3.853748	2.530764
94	1	0	-4.975208	-2.014819	1.577954
95	1	0	4.149080	0.249582	-0.660977
96	1	0	2.865316	-1.259616	2.944853
97	1	0	1.928851	-2.205332	1.807562
98	1	0	6.109254	-0.374917	0.414902
99	1	0	5.765877	-1.878890	-0.405974
100	1	0	4.048152	-3.317932	2.782769
101	1	0	3.932021	-3.390651	1.029896
102	1	0	6.232650	-2.623916	1.788847
103	1	0	5.472923	-1.230300	2.551641
104	1	0	2.685405	-0.931696	-1.980687
105	1	0	-1.521963	-2.193080	-0.018526
106	1	0	4.662833	-0.576643	-3.249187
107	1	0	6.568745	-1.521021	-4.511304
108	1	0	4.092322	-4.367386	-1.282998
109	1	0	5.996475	-5.299643	-2.533742
110	1	0	7.248318	-3.888015	-4.151857
111	1	0	2.209057	-3.546638	-0.401260

(2S,1'S)–**X_LX_L–INT3b**

Center Number	Atomic Number	Atomic Type	Coordinates (Angstroms) X	Y	Z
1	6	0	-3.996137	2.416284	-0.434178
2	6	0	-3.152247	1.707951	-1.302927
3	6	0	-2.520513	2.395416	-2.345777
4	6	0	-2.716816	3.765563	-2.517291
5	6	0	-3.550417	4.463737	-1.645036

6	6	0	-4.191109	3.785539	-0.605343
7	6	0	-2.911264	0.222817	-1.156969
8	6	0	-2.056123	-0.242791	0.057616
9	7	0	-2.439875	-1.685644	0.258592
10	6	0	-3.598203	-2.048348	-0.661254
11	6	0	-4.157739	-0.666575	-1.033730
12	6	0	-2.317859	0.518590	1.345581
13	8	0	-3.329915	0.277060	2.002588
14	6	0	-4.560173	-3.006809	-0.012220
15	6	0	-5.215693	-2.659209	1.180002
16	6	0	-6.113087	-3.550102	1.765548
17	6	0	-6.369721	-4.784803	1.162560
18	6	0	-5.723663	-5.129969	-0.025219
19	6	0	-4.816805	-4.244597	-0.609901
20	7	0	-4.894762	-0.727235	-2.348756
21	8	0	-6.018539	-0.249335	-2.376009
22	7	0	-1.428763	1.488304	1.711131
23	6	0	-0.002198	1.589750	1.397199
24	6	0	0.766248	0.304662	1.810221
25	7	0	1.952428	0.289569	0.895471
26	6	0	1.866742	1.380132	-0.119140
27	6	0	0.403598	1.906195	-0.053368
28	6	0	2.845733	-0.666233	0.866145
29	6	0	3.852056	-0.763950	-0.252603
30	6	0	5.268150	-1.179654	0.247237
31	6	0	5.314445	-1.898245	1.609913
32	6	0	4.030607	-2.690704	1.852795
33	6	0	2.851549	-1.715904	1.948595
34	6	0	1.033576	0.275880	3.299067
35	6	0	0.288310	-0.601242	4.094401
36	6	0	0.452501	-0.608507	5.480356
37	6	0	1.373174	0.254458	6.076606
38	6	0	2.119596	1.131718	5.284439
39	6	0	1.947272	1.152525	3.900581
40	6	0	0.255549	3.350663	-0.503877
41	6	0	0.748080	3.710337	-1.768449
42	6	0	0.642996	5.020464	-2.230774
43	6	0	0.023953	5.992630	-1.441181
44	6	0	-0.490095	5.639682	-0.194447
45	6	0	-0.372901	4.328575	0.271689
46	6	0	2.864689	2.478830	0.245001
47	8	0	2.940879	2.980753	1.345818
48	8	0	3.606514	2.817525	-0.811891
49	6	0	4.474145	3.964736	-0.629829
50	8	0	-4.294650	-1.232934	-3.295756
51	6	0	3.136349	-1.723191	-1.373036
52	6	0	2.047476	-2.564315	-0.770473
53	7	0	0.824008	-2.089292	-0.651241
54	8	0	-0.032797	-2.726175	0.123033
55	6	0	4.151938	-2.477152	-2.204136
56	6	0	4.792817	-1.800641	-3.252657
57	6	0	5.758976	-2.434755	-4.031967
58	6	0	6.102346	-3.763662	-3.773220
59	6	0	5.472434	-4.448462	-2.733330
60	6	0	4.507048	-3.809514	-1.953089
61	8	0	0.458303	-0.982082	-1.207220
62	1	0	-2.736842	-1.831851	1.228211
63	1	0	-1.011076	-0.271043	-0.237965
64	1	0	-2.369399	-0.131972	-2.041511
65	1	0	-4.874863	-0.296930	-0.303765
66	1	0	-3.154158	-2.483707	-1.559086
67	1	0	-1.714090	1.945404	2.570698
68	1	0	0.377680	2.399733	2.022822
69	1	0	0.194365	-0.585020	1.531477
70	1	0	2.068671	0.973916	-1.104149
71	1	0	-0.158602	1.282994	-0.746285
72	1	0	5.022573	4.061407	-1.565511
73	1	0	3.864989	4.851594	-0.440435
74	1	0	5.152298	3.795559	0.209104
75	1	0	-1.872358	1.853490	-3.030545
76	1	0	-2.210620	4.285543	-3.323887
77	1	0	-3.703628	5.530813	-1.775744
78	1	0	-4.850276	4.321987	0.071113
79	1	0	-4.500277	1.903944	0.379526
80	1	0	1.221123	2.960277	-2.396881
81	1	0	1.036963	5.279794	-3.209432
82	1	0	-0.063641	7.014017	-1.799885

83	1	0	-0.986777	6.383601	0.421461
84	1	0	-0.798698	4.078909	1.237144
85	1	0	-0.417928	-1.285463	3.629329
86	1	0	-0.132094	-1.291169	6.089852
87	1	0	1.508635	0.245078	7.154058
88	1	0	2.834923	1.806364	5.745817
89	1	0	2.517756	1.842458	3.286423
90	1	0	-4.313685	-4.513406	-1.535210
91	1	0	-5.922629	-6.087369	-0.497204
92	1	0	-7.074643	-5.474411	1.617482
93	1	0	-6.618726	-3.277484	2.687123
94	1	0	-5.027464	-1.694690	1.648476
95	1	0	3.967707	0.201541	-0.744070
96	1	0	2.913245	-1.189644	2.908037
97	1	0	1.880511	-2.228640	1.948814
98	1	0	5.886230	-0.276903	0.291114
99	1	0	5.713002	-1.815609	-0.520987
100	1	0	4.083427	-3.258906	2.786780
101	1	0	3.882406	-3.418384	1.049338
102	1	0	6.191028	-2.552133	1.650204
103	1	0	5.432506	-1.166681	2.421132
104	1	0	2.626342	-1.018147	-2.032859
105	1	0	-1.526947	-2.252359	0.080342
106	1	0	4.525563	-0.766931	-3.463062
107	1	0	6.237274	-1.895573	-4.844657
108	1	0	4.027797	-4.365729	-1.153604
109	1	0	5.728816	-5.483983	-2.528672
110	1	0	6.851916	-4.262475	-4.380469
111	1	0	2.204519	-3.517029	-0.291679

(*Si*,*Si*)–$\mathbf{X_LX_L}$–TS2b

Center Number	Atomic Number	Atomic Type	Coordinates (Angstroms) X	Y	Z
1	6	0	-6.355821	3.170686	-3.752127
2	6	0	-5.833399	4.081612	-2.833284
3	6	0	-4.768067	3.711666	-2.011920
4	6	0	-4.200213	2.431063	-2.109316
5	6	0	-4.740860	1.519832	-3.033022
6	6	0	-5.807940	1.887055	-3.847195
7	6	0	-3.053097	2.079977	-1.239821
8	6	0	-4.019470	0.801084	0.126416
9	6	0	-2.955982	0.698888	1.057320
10	6	0	-2.781584	1.786397	2.092848
11	6	0	-3.894141	2.843672	2.064960
12	6	0	-5.251807	2.154519	1.925186
13	6	0	-5.336547	1.431770	0.571638
14	7	0	-2.039019	-0.278003	0.988985
15	6	0	-0.774683	-0.285676	1.758310
16	6	0	-0.083431	-1.596449	1.297546
17	6	0	-0.592088	-1.825061	-0.137241
18	6	0	-2.080383	-1.375020	-0.000879
19	7	0	1.356754	-1.583037	1.587800
20	6	0	2.235112	-0.578800	1.292833
21	8	0	3.160235	-0.245789	2.030864
22	6	0	-0.863768	-0.225744	3.269495
23	6	0	0.038300	0.584762	3.966189
24	6	0	0.038004	0.607307	5.361749
25	6	0	-0.874793	-0.175597	6.069754
26	6	0	-1.776218	-0.989263	5.377320
27	6	0	-1.768410	-1.023847	3.983035
28	6	0	-2.955449	-2.534024	0.472805
29	8	0	-3.618825	-3.060395	-0.558099
30	6	0	-4.318760	-4.299561	-0.290011
31	6	0	-0.416603	-3.194960	-0.756269
32	6	0	-0.449830	-3.288757	-2.154919
33	6	0	-0.342287	-4.523804	-2.791011
34	6	0	-0.193163	-5.687550	-2.031743
35	6	0	-0.154320	-5.603738	-0.639352
36	6	0	-0.269937	-4.365100	-0.004029
37	6	0	2.085242	0.093500	-0.056992
38	7	0	2.268784	1.580682	0.112076
39	6	0	3.423183	2.044523	-0.768479
40	6	0	4.232566	0.746383	-0.921787

41	6	0	3.162831	-0.339269	-1.095227
42	6	0	3.587866	-1.787271	-1.002753
43	6	0	2.949297	-2.717873	-1.830892
44	6	0	3.239407	-4.077688	-1.730715
45	6	0	4.189265	-4.519308	-0.808978
46	6	0	4.842163	-3.596442	0.011161
47	6	0	4.540577	-2.237732	-0.078884
48	7	0	5.123029	0.819723	-2.137257
49	8	0	4.596535	1.182080	-3.187126
50	6	0	4.144071	3.220998	-0.170777
51	6	0	4.232127	4.419642	-0.885185
52	6	0	4.915513	5.508912	-0.342235
53	6	0	5.504341	5.406410	0.918834
54	6	0	5.416050	4.211031	1.638264
55	6	0	4.743359	3.118447	1.095013
56	8	0	-2.981472	-2.953936	1.611043
57	8	0	6.290191	0.492362	-1.985650
58	1	0	2.504286	1.782701	1.089764
59	1	0	1.114272	-0.031587	-0.502506
60	1	0	2.706483	-0.162959	-2.075609
61	1	0	4.893125	0.570329	-0.075589
62	1	0	2.982662	2.286285	-1.737804
63	1	0	1.591107	-2.011843	2.477982
64	1	0	-0.488032	-2.399294	1.915758
65	1	0	-0.202058	0.577336	1.394241
66	1	0	-2.437602	-1.023916	-0.964135
67	1	0	-0.156540	-1.099973	-0.825327
68	1	0	-4.816809	-4.556954	-1.223561
69	1	0	-3.596285	-5.069638	-0.008858
70	1	0	-5.041658	-4.159071	0.516449
71	1	0	2.211693	-2.377950	-2.552383
72	1	0	2.718378	-4.785287	-2.367689
73	1	0	4.424591	-5.576986	-0.732352
74	1	0	5.588761	-3.933148	0.724548
75	1	0	5.039977	-1.538251	0.583289
76	1	0	-0.554678	-2.381416	-2.746984
77	1	0	-0.369281	-4.577952	-3.875568
78	1	0	-0.103891	-6.652175	-2.522975
79	1	0	-0.029881	-6.502638	-0.042470
80	1	0	-0.239561	-4.325990	1.079966
81	1	0	0.748772	1.199843	3.418258
82	1	0	0.744952	1.238513	5.892089
83	1	0	-0.883169	-0.154169	7.155540
84	1	0	-2.485667	-1.603200	5.924780
85	1	0	-2.458990	-1.664429	3.443829
86	1	0	3.775339	4.497591	-1.868362
87	1	0	4.986565	6.435043	-0.904483
88	1	0	6.035594	6.254497	1.340538
89	1	0	5.879673	4.127158	2.616584
90	1	0	4.692944	2.185062	1.654578
91	1	0	-4.158834	-0.032917	-0.554026
92	1	0	-2.758556	1.318264	3.081643
93	1	0	-1.796778	2.244572	1.944381
94	1	0	-6.088166	0.633800	0.618076
95	1	0	-5.689938	2.122907	-0.200221
96	1	0	-3.837804	3.432275	2.986241
97	1	0	-3.746595	3.547230	1.236434
98	1	0	-6.071910	2.873854	2.015411
99	1	0	-5.368958	1.434256	2.746195
100	1	0	-2.763484	2.853887	-0.536048
101	1	0	1.343102	2.035061	-0.128359
102	1	0	-4.363167	4.423475	-1.296897
103	1	0	-6.254819	5.079250	-2.752859
104	1	0	-7.188190	3.455171	-4.388861
105	1	0	-6.216383	1.172817	-4.556135
106	1	0	-4.332379	0.514887	-3.105755
107	6	0	-1.948310	1.427564	-1.856990
108	1	0	-2.026503	0.891626	-2.791606
109	7	0	-0.684222	1.513892	-1.399184
110	8	0	0.259431	0.978534	-2.066420
111	8	0	-0.403651	2.135429	-0.294354

(2S,1'S)–**X_LX_L–INT3a**

Center	Atomic	Atomic	Coordinates (Angstroms)

Number	Number	Type	X	Y	Z
1	6	0	4.472783	3.289565	1.450452
2	6	0	3.836399	3.394605	0.203776
3	6	0	3.773040	4.635082	-0.438165
4	6	0	4.342385	5.761754	0.157431
5	6	0	4.968896	5.654505	1.399767
6	6	0	5.032158	4.417371	2.047464
7	6	0	3.236128	2.182161	-0.452591
8	7	0	2.162041	1.541830	0.405637
9	6	0	2.068754	0.083696	0.049867
10	6	0	3.226479	-0.183216	-0.965914
11	6	0	4.177403	0.990110	-0.693251
12	6	0	2.149555	-0.742267	1.315876
13	8	0	2.976487	-0.449244	2.176633
14	7	0	5.067090	1.252228	-1.882775
15	8	0	6.266059	1.075818	-1.725492
16	6	0	3.797406	-1.583079	-0.944385
17	6	0	3.330200	-2.504666	-1.888984
18	6	0	3.764400	-3.829257	-1.867634
19	6	0	4.688471	-4.242750	-0.907116
20	6	0	5.170411	-3.327028	0.031010
21	6	0	4.724689	-2.005477	0.018010
22	7	0	1.328079	-1.837754	1.409898
23	6	0	-0.103653	-1.878304	1.081476
24	6	0	-0.868375	-0.733111	1.800025
25	7	0	-2.083488	-0.521703	0.956083
26	6	0	-2.069700	-1.431763	-0.224830
27	6	0	-0.564783	-1.792484	-0.385852
28	6	0	-2.860955	0.525173	1.088203
29	6	0	-3.863636	0.901984	0.054602
30	6	0	-5.228026	1.333607	0.680988
31	6	0	-5.156233	1.744004	2.161195
32	6	0	-3.829946	2.447617	2.448844
33	6	0	-2.681948	1.442736	2.268490
34	6	0	-1.072931	-1.011042	3.273119
35	6	0	-0.111056	-0.525570	4.168969
36	6	0	-0.205768	-0.818033	5.530080
37	6	0	-1.270209	-1.584992	6.006758
38	6	0	-2.230452	-2.069720	5.115351
39	6	0	-2.131235	-1.794243	3.751235
40	6	0	-0.292815	-2.965370	-1.299810
41	6	0	-0.094618	-2.694841	-2.661050
42	6	0	0.135732	-3.731517	-3.564631
43	6	0	0.174915	-5.053291	-3.113938
44	6	0	-0.023213	-5.330291	-1.759747
45	6	0	-0.260289	-4.292594	-0.856188
46	6	0	-2.919738	-2.674847	0.012344
47	8	0	-2.933290	-3.309521	1.046749
48	8	0	-3.573490	-2.995787	-1.102743
49	6	0	-4.255313	-4.275137	-1.090178
50	8	0	4.511513	1.608631	-2.919270
51	6	0	-3.154677	2.062828	-0.839274
52	6	0	-1.971049	1.568586	-1.608629
53	7	0	-0.734589	1.583481	-1.146781
54	8	0	-0.477648	1.976269	0.090512
55	6	0	-4.175797	2.655734	-1.795451
56	6	0	-4.511005	4.011795	-1.710923
57	6	0	-5.437130	4.571910	-2.593216
58	6	0	-6.042500	3.778943	-3.568584
59	6	0	-5.716528	2.423252	-3.658995
60	6	0	-4.790934	1.866580	-2.777744
61	8	0	0.254820	1.190036	-1.869768
62	1	0	2.425860	1.629797	1.393328
63	1	0	1.137759	-0.008612	-0.481047
64	1	0	2.789651	0.007270	-1.951996
65	1	0	4.848319	0.817455	0.146351
66	1	0	2.748559	2.429455	-1.398259
67	1	0	1.547062	-2.365026	2.250531
68	1	0	-0.465107	-2.821520	1.493210
69	1	0	-0.298344	0.193742	1.672640
70	1	0	-2.409327	-0.912052	-1.113206
71	1	0	-0.152173	-0.911317	-0.871456
72	1	0	-4.758542	-4.341374	-2.053375
73	1	0	-3.519030	-5.074686	-0.979149
74	1	0	-4.971397	-4.313285	-0.266685
75	1	0	2.613307	-2.187665	-2.640121

76	1	0	3.378359	-4.531453	-2.600435
77	1	0	5.036814	-5.271536	-0.891440
78	1	0	5.895838	-3.640568	0.776128
79	1	0	5.090320	-1.314326	0.770504
80	1	0	-0.107733	-1.661916	-3.005109
81	1	0	0.291297	-3.507380	-4.615997
82	1	0	0.360244	-5.862866	-3.813948
83	1	0	0.012241	-6.355488	-1.402549
84	1	0	-0.416577	-4.529602	0.191697
85	1	0	0.722288	0.070007	3.803547
86	1	0	0.547414	-0.440698	6.215401
87	1	0	-1.350426	-1.805665	7.067033
88	1	0	-3.057457	-2.671613	5.480707
89	1	0	-2.862868	-2.194487	3.058874
90	1	0	3.286397	4.716820	-1.406500
91	1	0	4.295811	6.720779	-0.349585
92	1	0	5.411810	6.531498	1.862433
93	1	0	5.525271	4.330059	3.010964
94	1	0	4.542906	2.326489	1.955018
95	1	0	-4.054276	0.075858	-0.629799
96	1	0	-2.587879	0.830697	3.169876
97	1	0	-1.718166	1.947370	2.127368
98	1	0	-5.935823	0.507976	0.556357
99	1	0	-5.617406	2.163934	0.087337
100	1	0	-3.793484	2.834872	3.471711
101	1	0	-3.709465	3.309449	1.782491
102	1	0	-6.007584	2.386794	2.405453
103	1	0	-5.233638	0.859285	2.808952
104	1	0	-2.815498	2.839541	-0.149511
105	1	0	1.182187	1.951589	0.246488
106	1	0	-4.039964	4.635414	-0.954761
107	1	0	-5.683487	5.627055	-2.517104
108	1	0	-6.763777	4.212893	-4.254735
109	1	0	-6.182996	1.800106	-4.416667
110	1	0	-4.544733	0.809903	-2.860328
111	1	0	-2.056244	1.248597	-2.637549

10 References

[1] E. Conde, D. Bello, A. de Cózar, M. Sanchez, M. A. Vazquez, F. P. Cossío, *Chem. Sci.* **2012**, *3*, 1486–1491.

[2] (a) M. Ayerbe, A. Arrieta, F. P. Cossío, A. Linden, *J. Org. Chem.* **1999,** *63*, 1795-1805. (b) S. Vivanco, B. Lecea, A. Arrieta, P. Prieto, I. Morao, ALinden, F. P. Cossío, *J. Am. Chem. Soc.* **2000,** *122*, 6078-6092. (c) G. Revial, S. Lim, B. Viossat, P. Lemoine, A. Tomas, A. F. Duprat, M. Pfau, *J. Org. Chem.* **2000**, *65*, 4593-4600.

[3] (a) M. d. G. Retamosa, A. de Cozar, M. Sanchez, J. I. Miranda, J. M. Sansano, L. M. Castello, C. Najera, A. I. Jimenez, F. J. Sayago, C. Cativiela, F. P. Cossio, *Eur. J. Org. Chem.* **2015**, 2503–2516. (b) A. Ruiz-Olalla, M. d. G. Retamosa, F. P. Cossio, *J. Org. Chem.* **2015**, *80*, 5588–5599.

[4] (a) T. Llamas, R. Gómez Arrayás, J. C. Carretero, *Org. Lett.* **2006**, *8*, 1795-1798 (b) S. E. Denmark, H. Matsuhashi, *J. Org. Chem.*, **2002**, *67*, 3479-3486 (c) K. V. Kudryavtsev, M. Y. Tsentalovich, A. S. Yegorov, E. L. Kolychev, J. *Het. Chem.* **2006**, *43*, 1461-1466.

[5] M. Gruttadauria, F. Giacalone, A. M. Marculescu, P. Lo Meo, S. Riela, R. Noto, *Eur. J. Org. Chem.* **2007**, 4688-4698.

[6] N. Mase, K. Watanabe, H. Yoda, K. Takabe, F. Tanaka, C. F. Barbas III, *J. Am. Chem. Soc.* **2006**, *128*, 4966-4967.

[7] A. Quintard, A. Alexakis, *Chem. Eur. J.* **2009**, *15*, 11109-11113.

[8] V. K. Vishnumaya, Singh, *Org. Lett*. **2007**, *9*, 1117-1119.

[9] L. Gu, Y. Wu, Y. Zhang, G. Zhao, *J Mol Catal A Chem.* **2007**, *263*, 186-194.

[10] M. d. G. Retamosa, A. Ruiz-Olalla, T. Bello, A. de Cózar, F. P. Cossío, *Angew. Chem. Int. Ed.* **2018**, *57*, 668–672.

[11] D. C. Harris, *Experimental Error in Quantitative Chemical Analysis*, 8th ed.; W. H. Freeman: New York, 2010; pp 51-67.
